# Symbiont‐Derived Key Innovations Across the Diversity and Diets of Herbivorous Insects

**DOI:** 10.1111/mec.70615

**Published:** 2026-11-07

**Authors:** Jacob A. Russell, Hannah M. Smith, Benoit Bechade, Christian Cabuslay, Melissa Carpenter, Daniel Freeman, Allison Hansen, Clesson Higashi, Yi Hu, Linyao Peng, Abigail Williams, Patrick Abbot

**Affiliations:** ^1^ Department of Biology Drexel University Philadelphia Pennsylvania USA; ^2^ Department of Biodiversity, Earth, and Environmental Sciences Drexel University Philadelphia Pennsylvania USA; ^3^ Department of Entomology University of California Riverside Riverside California USA; ^4^ College of Life Sciences Beijing Normal University Beijing China; ^5^ Department of Biological Sciences Vanderbilt University Nashville Tennessee USA; ^6^ National Science Foundation Alexandria Virginia USA

**Keywords:** herbivory, insect, key innovation, nutrient, plant tissue, symbiosis

## Abstract

With prodigious biomass and diversity, herbivorous insects are among the world's most successful animals. It is widely believed, however, that some plant‐based diets do not meet the needs of insect metabolism. Microbial symbionts may balance such deficits through diverse biochemistry. Here, we consider whether obligate nutritional symbioses between insects and microbes support large portions of insect herbivore diversity. In doing so, we assess the evidence that these highly integrated relationships are key innovations, circumventing nutritive shortfalls within particular plant tissues. Toward these ends, we compile nutrient data for 20 plant‐ and non‐plant foods, plus data on diet and symbiosis across 874 families of insects—both herbivorous and non‐herbivorous. Our results reinforce widely believed, but often uncited evidence that several insect taxa have long‐consumed plant tissues with low concentrations of B‐vitamins, nitrogen, or accessible carbon. Emerging as symbiont‐enriched are seed‐feeders, which face insufficient quantities of several B‐vitamins in their food. Experimental and genome assessments show how symbionts surmount these B‐vitamin shortcomings, showing also where this same challenge, and those of high fibre or low amino acid content are surmounted through symbiosis on other diets. We thus show where symbiont services are key innovations. Over half of the world's insect herbivores persist without nutritive assistance from obligate symbionts. This is largely because many consume leaves, which are reasonably good sources of amino acids and B‐vitamins. Lateral transfer of microbial genes encoding plant cell‐wall degrading enzymes has helped to overcome high fibre content, defusing the most challenging nutritive aspect of leaf‐chewing diets.

## 1 Introduction

For nearly 500 million years, terrestrial ecosystems have been revolutionized by plants and the turnover in their diversity, form, and function (Bateman et al. 1998; Raven and Edwards 2001; Clarke et al. 2011; Labandeira 2014; Gibling and Davies 2012; Bowman et al. 2017; Lutzoni et al. 2018; Benton et al. 2022). While diverse themselves (Christenhusz and Byng 2016), plants support even more biodiversity, insects perhaps most impressively. Insects comprise more than half of all described species on Earth; and nearly half of all described insects are thought to be herbivorous (Zhang 2013; Rainford and Mayhew 2015; Stork 2018; but see Jezkova and Wiens 2017; Forbes et al. 2018). While much diversity awaits discovery (Scheffers et al. 2012; Costello and Chaudhary 2017), the half million plant‐feeding insect species comprise ~33% of all currently described animals, nearly equaling the described diversity of plants and fungi combined (Enquist et al. 2019; Benton et al. 2022; Bhunjun et al. 2022). To explain insect diversity is to, thus, explain a substantial portion of life on Earth.

The story of how so many insect species have radiated across plant‐based feeding niches is itself a story of diverse adaptations enabling ingestion, digestion, and assimilation of plant biomass (Cummins and Klug 1979; Karasov 1982; Wiegert and Petersen 1983; Slansky 1985; Simpson and Simpson 1990). Were adaptations mere adjustments to pre‐existing characters? Or were more drastic changes required to insect body plans and physiology? In addressing these questions we can test the fit of the key innovation model for explaining insect herbivory. Under this hypothesis, adaptive novelties enable the use of a previously inaccessible niche (Miller et al. 2023).

## 2 Origins of Diverse, Herbivorous Diets

Colonizing land ~480 MYA—just as plants were towelling off from their 500 MYA water‐to‐land transition (Rota‐Stabelli et al. 2013; Lozano‐Fernandez et al. 2016; Schwentner et al. 2017; Morris et al. 2018; Schreiber et al. 2022)—the hexapods began their terrestrial lives eating detritus and microbes (Rainford and Mayhew 2015). Since this time the diets of hexapods, including insects, have undergone repeated origins of herbivory, predation, scavenging, and parasitism, and reversions to ancestral foods (Weaver III and Morse 1986; Peters et al. 2014, 2017; Rainford and Mayhew 2015; Nelsen et al. 2018; Johnson et al. 2018; McKenna et al. 2019; Weirauch et al. 2019).

Plant diets are seen as challenging compared to other insect foods (Simpson and Simpson 1990; Berenbaum 1995; Behmer 2009; McNeil and Southwood 1978; Akin 1988; Mitter et al. 1988; Kaspari et al. 2008; Filipiak et al. 2017). This stems from (1) defensive phytochemistry (Dyer et al. 2018), (2) low nitrogen content compared to insect tissue (Fagan et al. 2002), (3) limiting quantities of B‐vitamins and amino acids in light of animals' lost biosynthetic capacities (Payne and Loomis 2006; Helliwell et al. 2013), and (4) recalcitrant lignocellulose barriers comprising plant cell walls (but see Shelomi et al. 2020). The prodigious diversity of insect herbivores shows these challenges are not insurmountable. Have different insects converged upon similar solutions to these nutritive dilemmas? Do these adaptations fit the definition of key innovations?

Addressing these questions requires fine‐scale study, focused on consumption of distinct plant tissues (Janzen 1971; Weisser and Siemann 2008; Novotny et al. 2010; Vidal and Murphy 2018). Plant tissues vary biophysically and biochemically (Mattson 1980; Robbins 1993; Felton 1996; Lucas 2000; Roulston et al. 2000; Sanson 2006; Berenbaum and Johnson 2015), and immature insects often specialize on one such part of plant architecture (Farrell and Sequeira 2004; Mitter and Winkler 2008). Each plant species, thus, supports several dietary niches. So while studies of host plant switching have been integral in understanding insect diversity (Janz et al. 2006; Hardy and Otto 2014; Forbes et al. 2017), studies on the mechanisms allowing consumption of different plant tissues must be a further priority in insect diversity research (Novotny et al. 2012; Ding et al. 2015).

## 3 Herbivore Adaptations—Endogenous and Outsourced

Evolving quite literally on the leading edge of insect‐plant tissue interactions are insect mouthparts, preserved well within the fossil record (Labandeira and Sepkoski Jr. 1993). Many of the extant insect buccal forms arose ~150–250 million years ago (MYA), during and after Permian and Triassic diversification of non‐angiosperm seed plants (Labandeira 2013, 2019, 2014). These early modifications to the maxillae, labium, labrum, hypopharynx, and mandibles paved the way for modern, specialized consumption of the tissues and fluids of seed‐bearing plants (Nel et al. 2018). Insect mouthparts, thus, help to tell the story of radiations onto plants and their partitioning into tissue‐specific diets.

Acting alongside insect mouthpart adaptation have been changes to other traits, less amenable to fossil preservation. Alterations to feeding behaviour (Bernays 1998), behavioural physiology (Goldman‐Huertas et al. 2015), and abilities to manipulate host plants (Nabity et al. 2013) have played important roles. Of further importance have been the many anatomical (King 1991; Miguel‐Aliaga et al. 2018) and physiological changes to the digestive tract (O'Brien et al. 1979; Berenbaum and Isman 1989; Appel 1994; Johnson and Felton 1996; Bernays 1998; Clark 1999; Coy et al. 2010; Ke et al. 2012; Sabbadin et al. 2018; Gloss et al. 2019; Seppey et al. 2019; Terra and Ferreira 2020), with their resulting effects on detoxification and nutrient extraction.

Emerging as a major theme alongside such discoveries is a trend of outsourcing, where plant‐feeding insects rely upon symbiotic microbes. Broad, though mostly indirect evidence for symbiont‐supported plant use by insects was synthesized by Paul Buchner in his 1965 book. This came two years before the seminal Lynn Margulis paper on the symbiotic origins of plastids and mitochondria (Sagan 1967). Since this time, a moderate body of evidence has shown how the ancient microbial inventions of nutrient scavenging, biosynthesis, and assimilation (Strope et al. 2011; Barruetabeña et al. 2019; Pi et al. 2022) were co‐opted through symbiosis to benefit plant‐feeding insects.

Given the broad distrubutions of symbiosis across insects (Douglas 2021), one might ask ‘Why re‐invent the wheel?’ Shouldn't prokaryotic, protist, or fungal symbionts prove sufficient in solving herbivorous nutritive dilemmas? Support and speculation for this have grown in recent years with the growing ease of molecular and experimental study (Douglas 2009; Feldhaar 2011; Frago et al. 2012; Vavre and Kremer 2014; Hammer and Bowers 2015; Sudakaran et al. 2017; Mason et al. 2019). But unlike clear structure/function relationships for mouthpart morphologies, the impacts of symbionts on host physiology, ecology, and fitness (i.e., their ‘functions’) are sometimes undefined. In looking across the insect phylogeny, just where do symbioses equate to novel insect traits and where do they compensate for shortcomings in the diet? In addressing this, systematically, we can further ask: what fraction of insect herbivory requires nutritional symbiosis? With growing evidence for symbiont‐independent plant‐exploitation (Buchner 1965; Shelomi et al. 2015; Hammer et al. 2017; Sanders et al. 2017; Rubin et al. 2019), a synthesis of these topics would seem timely.

## 4 Study Objectives

In a systematic study across the insect phylogeny, we identify where nutritive services of highly integrated, obligate symbionts meet unique needs of plant tissue diets. We then estimate the levels of diversity sustained by such symbiotic key innovations. In our first objective we consult the literature to define tissue‐specific diets. Adults from several insect groups do not feed, and many adult insects are short‐lived. But diverse nutrient intake is always pivotal for growth and development in immature insects. We, thus, placed much of our emphasis on the diets of larvae and nymphs. With data across 807 out of 1110 insect families (Rainford et al. 2014), we provide insight into diet across a large portion of insect diversity. We then use previous estimates of insect species richness and a review of the symbiosis literature to investigate whether most insect herbivores harbour derived, highly integrated, obligate microbial symbionts. Through identifying the plant tissue diets most tightly correlated with such symbioses, we add nuance and novelty to the conventional wisdom in this area.

For our second objective, we assess whether obligate symbiont‐encoded nutritive services fit the original definition of key innovations—novel traits enabling the use of a previously inaccessible niche (Figure 1; Miller et al. 2023). Extracting data on nitrogen, B‐vitamin, and fibre quantities from 163 plant families, and for several non‐plant foods, we define multiple dimensions of hospitality for each dietary niche. We also review in vivo evidence for obligate symbiont function, while drawing further inference from genome‐encoded metabolism across 23 origins of obligate symbiosis. By combining these efforts, we assess where symbiont services match specific nutritional shortcomings of plant tissue diets.

**FIGURE 1:**
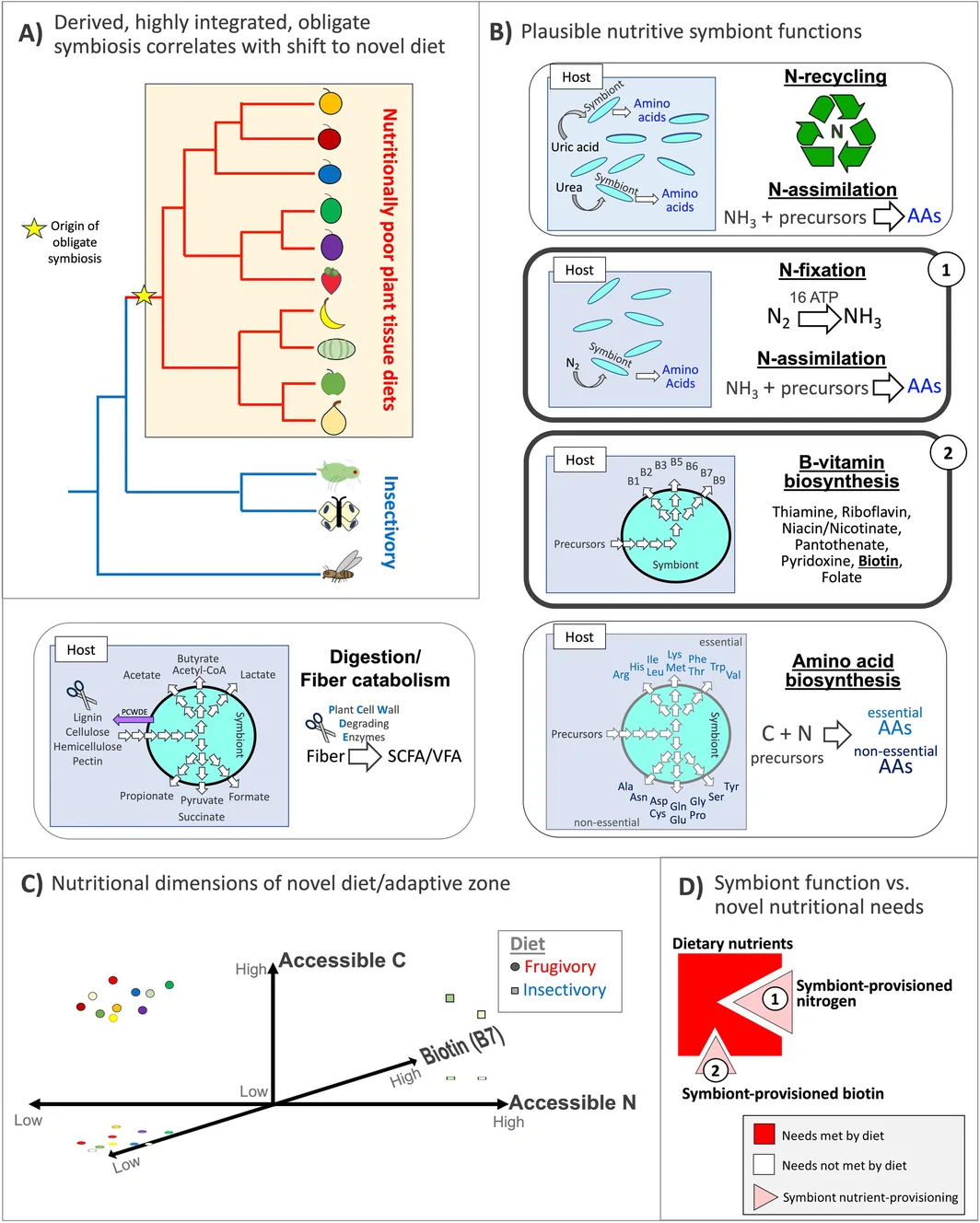
Symbiont‐driven, nutritional key innovations. To classify an obligate symbiosis as a nutrient‐themed key innovation, one must fulfil the following. (A) Demonstrate that a dietary shift evolves in association with highly integrated obligate symbiosis. (B) Characterize symbiont metabolism and directly show that the host insect acquires symbiont‐generated nutrients. (C) Identify nutritive deficiencies that are unique to the novel diet compared to the ancestral diet. And (D) reveal how symbiont metabolism/nutrient provisioning matches these unique shortcomings of the novel diet. This unified approach has rarely been applied across insect taxa. Achieving this in the study of insect herbivory requires study of both plant (i.e., fruit) and non‐plant diets (i.e., other insects). Key figure abbreviations include: AA, amino acid; SCFA, short chain fatty acid; VFA, volatile fatty acid. Nutrient values in panel C are hypothetical and used to illustrate relative levels in the novel (fruit) versus ancestral (insect tissue) diets.

We conclude by connecting knowledge on symbiont function, dietary nutrition, and insect herbivore diversity. Obligate symbionts may shape diversity through several means. We do not view their nutritive services as diversifying traits (Janson et al. 2008; Hansen and Moran 2014). Instead, we envision these as membership cards enabling entry and persistence within a nutrient‐limited niche. Where do such membership cards sustain diverse taxa, meeting unique needs of specialized, nutrient‐poor diets? And in which clades can we, thus, define them as key innovations? We discuss these topics in light of insect species richness, exploring the symbiotic dimension of the insect‐plant‐diversity nexus.

### 4.1 Objective 1A—What Do Insects Eat?

To delineate diet, we reviewed 2285 peer‐review articles and 98 separate websites. We obtained data for 874 insect families (Class Insecta) spanning 25 orders, and for three insect groups studied only at the order level (Archaeognatha, Strepsiptera, and Zoraptera). Methodologies, raw data, and summarized results for each of these—and all subsequent—efforts can be found in Data S1, in our Supplementary Tables, and in the Zenodo data repository (Zenodo materials enumerated in Data S2).

While prior studies have summarized diet across insects, we aimed for improved precision, moving beyond broad categories (i.e., ‘herbivory’, ‘omnivory’) to include 14 distinct plant tissues and six other non‐herbivorous foods (Figure 2; Box 1). Inclusion of separate plant tissues seemed crucial due to prior findings of varying nutritive content across plant anatomy (Mattson 1980; Robbins 1993). Inclusion of non‐plant foods clarified whether insect families are predominantly herbivorous or non‐herbivorous, and helped to later contexualize plant nutritive content within a broader dietary perspective.

**FIGURE 2:**
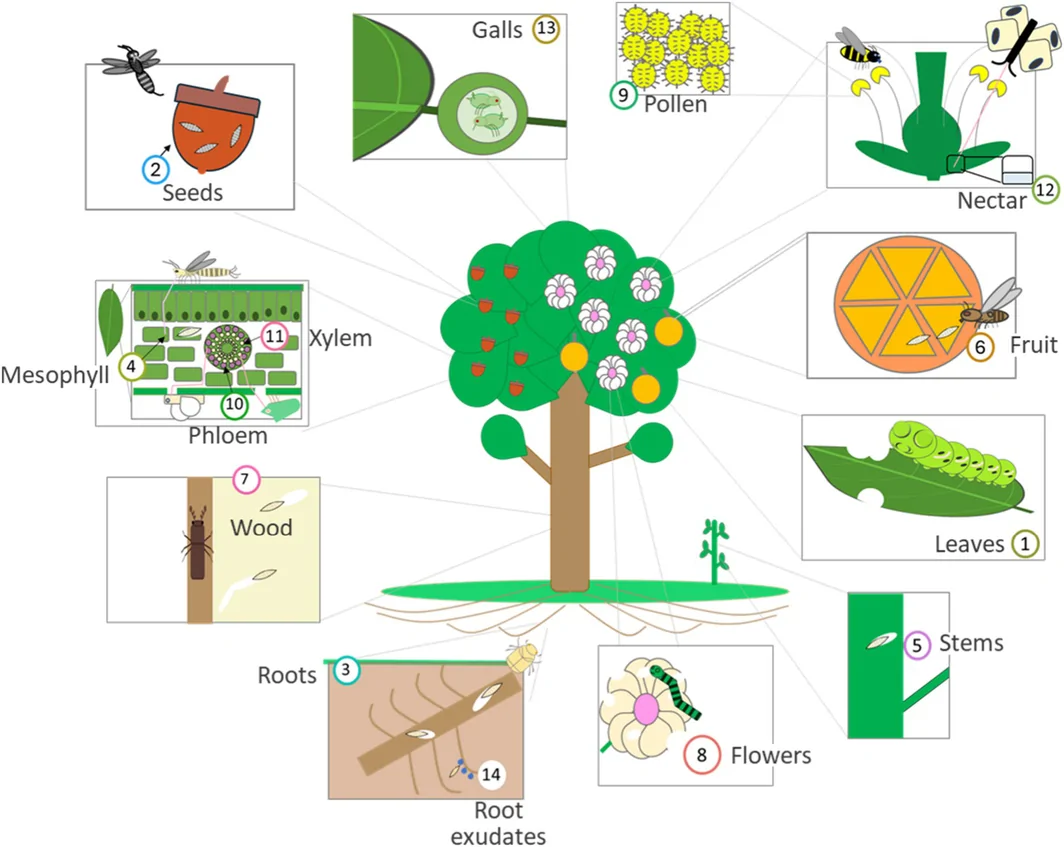
Vascular plant tissues utilized by insect herbivores. While insects consume a range of non‐plant‐based foods (animal tissue, blood, detritus, microbes, algae, and ‘other’), we focus here on 14 plant‐tissue‐specific food items. Using diversity‐weighted measures of juvenile stage consumption for each tissue across insect families, we illustrate the prevalence of tissue consumption with circle‐highlighted numerical rankings. Leaf‐chewing was the most prevalent herbivorous feeding habit (Figure S3). Least prevalent was root exudate consumption. Diets of galling insects were classified as phloem, mesophyll/parenchyma, or microbes when such precision was available. When unclear, we classified these diets as ‘gall’ tissue.

BOX 1 **Refined measures of insect diet.**

Building on the work of prior comprehensive studies (Labandeira 2014; Rainford and Mayhew 2015; Cornwallis et al. 2023), we aimed to improve precision of insect diet classification, while expanding the covered taxonomic breadth (Figure B1). Our literature review spanned 874 insect families—a large portion of their taxonomic diversity. Some insect families are highly specialized on a single food source, but many consume a mixture due to species‐level polyphagy or dietary evolution among monophagous insects.

Prior insect‐wide studies have used broad dietary bins, including an ‘herbivory’/‘herbivore’ category based on consumption of any plant tissue. Since nutrition varies between leaves, stems, flowers, roots, seeds, fruit, wood, pollen, and phloem/xylem sap, we focused on specific tissues. This enhanced our study on the links between symbiont function and dietary nutrition.

To illustrate our expanded and refined dietary summaries, we show data from the first 20 alphabetically sorted beetle families with GBIF database species richness data (Figure B1). Included are previously published family‐level diet classifications along with our assessments—that is, 1–3 ordinal scores for any food consumed at the juvenile stage. By summing these scores across plant‐based and non‐plant‐based foods, we estimate whether families are predominantly herbivorous. When combined with family‐level species counts, these scores reveal insect‐wide prevalence of diverse feeding habits.

**FIGURE B1:**
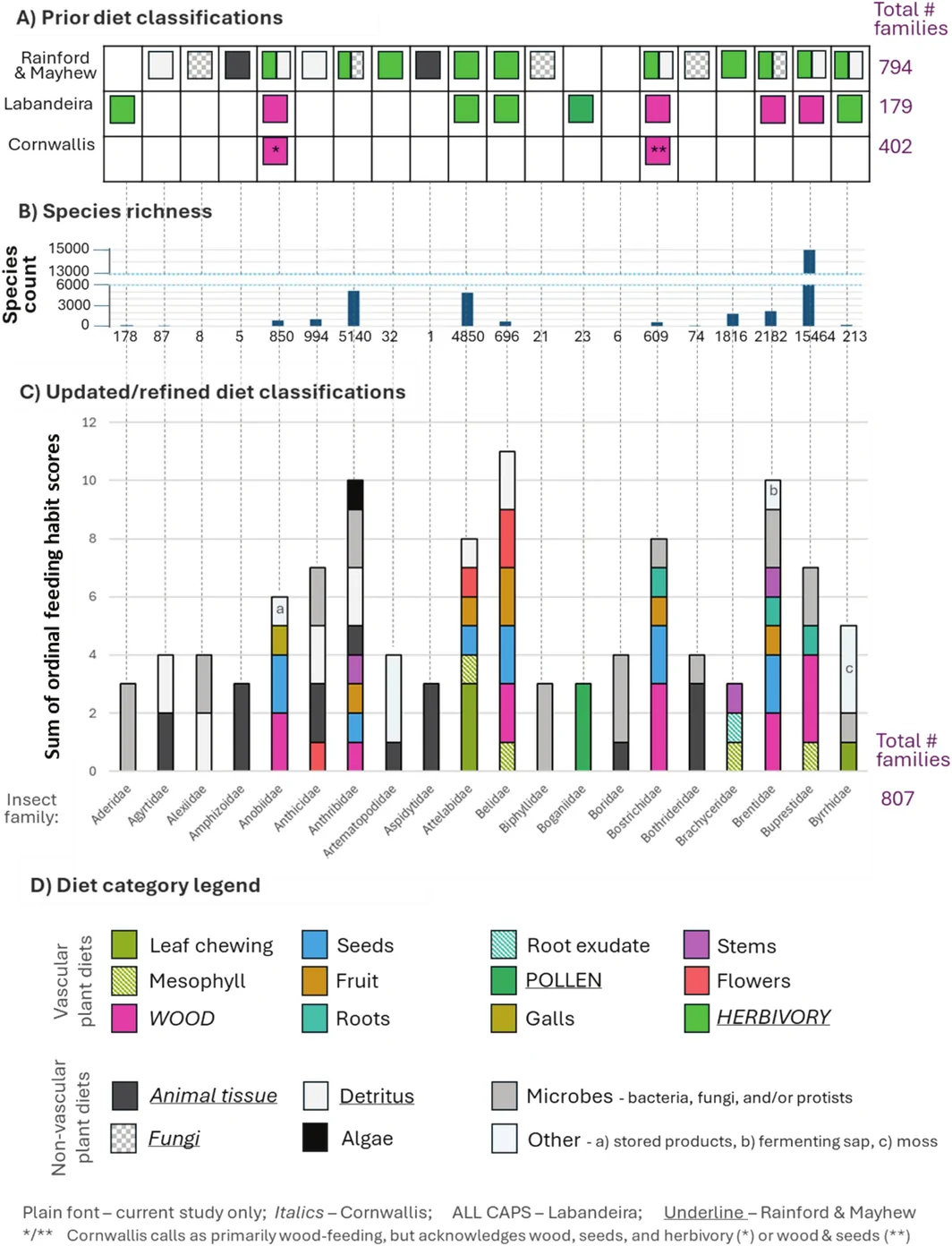
Comparing prior (A) and current (C) understanding of diet across insect families. Panel B shows insect family species richness, while panel D shows the figure legend.

For each insect family, we used ordinal scoring for each of 20 food categories, with ‘1’ denoting rare consumption, and ‘2’ and ‘3’ denoting moderate and prevalent consumption, respectively. Dietary data were successfully obtained for immature stages across 807 of 874 surveyed insect families (Figure S1a). Families lacking such data comprised a small fraction of our covered species richness (e.g., black bars in Figure S1b). To summarize diet, we reported the average use of each food by averaging ordinal scores for the given food across all families within each insect order (Figure S2a). In these analyses, we used ‘0’ to indicate no consumption. We also provided diversity‐weighted measures of feeding prevalence across these varying foods. To achieve this, we multiplied the ordinal scores for each consumed food within a family by the family's species richness value—that is, the sum of all accepted and provisional species in the Global Biodiversity Information Facility (GBIF) database as of August 2023 (https://www.gbif.org). We then summed these products within each food category. Since insect families vary drastically in species richness, this approach provided a clearer measure of dietary prevalence across the insect phylogeny (Figure S2b).

Our literature review revealed that immature insects from 15 out of 27 orders consume vascular plant tissues to at least some extent (Figure S2). Plant tissue consumption was seen within 379 out of 807 insect families with available immature‐stage dietary data. Leaf‐chewing was the most common herbivorous habit according to unweighted and species‐weighted measures (Figure 2; Figure S3). Seed‐feeding and root chewing were the next most prevalent for herbivores under our species‐weighted measures, followed by mesophyll consumption by leaf‐mining, leaf‐skeletonizing, and leaf‐piercing/sucking insects. We found a lower prevalence of stem‐boring, and wood‐, fruit‐, and flower‐feeding at the immature stage. Rarer, still, was consumption of pollen, phloem sap, and xylem sap.

Looking beyond herbivorous diets, insects from 24 of 27 studied orders were reported to eat animal flesh. This ‘predator/scavenger/parasite’ lifestyle was the most widespread feeding habit at immature stages according to both species‐weighted and unweighted measures (Figure S3). Consumption of microbial biomass was the next most common non‐herbivorous habit, followed by feeding on algae, detritus, foods from the ‘other’ category, and blood.

To understand the predominance of herbivory within and among insect families, we summed our 1–3 prevalence scores across all vascular plant tissue foods within each family, comparing these sums to those across non‐herbivorous foods. Nonwithstanding bias due to the larger number herbivorous versus non‐herbivorous categories (14 vs. 6), and other sources of imprecision (Data S1—discussed in results section), 264 of 807 families had a higher prevalence of herbivorous feeding at the juvenile stage. These represented 10 of the 28 studied insect orders, and an estimated 539,454 species, exceeding the 495,974 species from predominantly non‐herbivorous families.

These estimates are comparable to previous suggestions of ~500,000 herbivorous insect species (Rainford and Mayhew 2015; Hardy et al. 2020). Among the most diverse insect families found to be primarily non‐herbivorous were Crabronidae (wasps), Staphylinidae (beetles), Ichneumonidae (wasps), Braconidae (wasps), and Tachinidae (flies). Immature insects from each of these families are predominantly seen as predators and parasitoids (Table S1). Ants (Formicidae) comprised another diverse predominantly non‐herbivorous family, matching current perceptions (Nelsen et al. 2018). Imperfection in our binning was plausibly evidenced by the grouping of Arctiidae (moths) into the predominantly non‐herbivorous group. This was driven by moderate larval consumption of both microbes and algae in addition to leaves—their most common diet.

Among the predominant herbivores, Curculionidae (beetles), Scarabaeidae (beetles), Cerambycidae (beetles), Geometridae (moths), and Cicadellidae (leafhoppers) were the most diverse. Also grouped here was the similarly diverse Carabidae (beetles) family, comprising another possible mis‐classification. This stemmed from carabids' moderate consumption of seeds and rare consumption of fruit, in addition to their common predatory lifestyles. Another possible caveat was that while our GBIF estimate for the Chrysomelidae equaled 13,560 accepted and provisional species, some species counts for this beetle family are much higher (~32,500). Reliance on these would have placed this family as the fifth‐most‐diverse herbivore group (Slipinski et al. 2011).

Ranges of plant‐based foods were broad for the Curculionidae, Scarabaeidae, and Cerambycidae. More specialized diets were seen among the Geometridae and Cicadellidae, which had immature‐stage feeding habits of leaf‐chewing and phloem‐feeding, respectively. In general, while we found just one juvenile feeding habit for a high percentage of herbivorous insect families (Figure S4a), these families were often non‐diverse (Figure S4b). As a result, most herbivorous insect species belonged to families with multiple juvenile feeding habits.

### 4.2 Objective 1B—Where Do We Find Obligate Symbioses?

#### 4.2.1 Symbionts Across the Insect Phylogeny

To relate diet and insect diversity to symbiosis, we next reviewed the incidence of obligate symbioses across our studied insect families. Criteria for determining an insect family as host to a derived, highly integrated, and (likely) required symbiont are described in Box 2 (see also Table S2). In short, we looked for symbioses showing some combination of the following: housing within elaborated gut regions or specialized cells/organs; transmission or acquisition through derived host behaviours or physiologies; and ubiquity across host species or broader host clades. Using these criteria in a literature search spanning 879 insect families (Data S1), we found no evidence for highly integrated, obligate symbiosis in 722 such taxa. Searches returned for 157 families suggested a need for closer examination. To identify those meeting some of the above criteria, we reviewed 343 published articles. Likely or unequivocal cases of highly integrated, obligate symbiosis were seen for 115 families. These spanned the orders Blattodea, Psocoptera, Hymenoptera, Diptera, Coleoptera, and Hemiptera, comprising 13.1% of all surveyed families. Among the remaining families with possible cases, two had ambiguous evidence, while 40 had little‐to‐no supporting evidence. The rarity—and possible absence—of highly integrated, obligate symbioses among the frequently herbivorous Phasmatodea, Orthoptera, and Lepidoptera supports conclusions from prior papers (Buchner 1965; Shelomi et al. 2013; Paniagua Voirol et al. 2018; Frago et al. 2020).

BOX 2 **Defining highly integrated, ‘obligate’ symbioses as novel insect traits.**

Diverse microbial interactions have a broad capacity to shape insect nutrition. This is sometimes true for low‐density gut microbiota or heritable, facultative symbionts (Bailly‐Bechet et al. 2017; Sanders et al. 2017; Moran et al. 2019). These run‐of‐the‐mill interactions are often quite labile, making them poor candidates for macroevolutionary study. With such interactions appearing to be the baseline across the insects, they do not fit the bill of ‘novel traits’ envisioned by the key innovation hypothesis.

To align with this definition, we focused on ubiquitous, highly integrated, and derived symbioses (Kucuk 2020; Table S2). When members of a host family used novel anatomies or behaviours for housing or transmitting symbionts, or when there was evidence for tight physiological integration, long‐term conservation, and/or ubiquity across species, we considered symbioses to be novel traits. We refer to these as obligate because extremely prevalent symbionts, integrated through derived host features, *are* often required by their hosts. Despite this, there remains a need for in‐depth fitness measures across a decent fraction of these insects (Fisher et al. 2017).

We searched for these criteria across the literature focusing, first, on ubiquity—cases where symbionts are found in all, or nearly all, individuals within a species. Ubiquity can also extend across entire insect clades when symbiosis predates host diversification (Bennett and Moran 2015). When the literature suggested ~100% prevalence of a symbiotic microbe across one or more host species within the studied insect family, we took this as partial evidence for reliance on symbiosis. Ubiquity is not a fail‐proof means for identifying highly derived and long‐impactful symbioses. It is sometimes achieved by symbionts that manipulate insect reproduction (i.e., *Wolbachia, Rickettsia, Spiroplasma*, or *Cardinium*—e.g., Perlman et al. 2008; Behar et al. 2010; Turelli et al. 2022). As such, short of meeting criteria spelled out below, we did not take ubiquity for known reproductive manipulating taxa as strong evidence of a derived, obligate symbiosis.

The second of our researched criteria concerned symbiont housing. Novel structures and physiologies have evolved to maintain and transmit symbionts in a limited range of insects. While microbes may sometimes rapidly evolve the capacity to be symbiotic (Medina Munoz et al. 2020; Koga et al. 2022), symbiont‐supportive housing structures have likely required a complex series of genomic, developmental, and physiological changes (Hansen and Moran 2011; Wilson and Duncan 2015; Skidmore and Hansen 2017; Alarcón et al. 2022). The origin and conservation of such structures may, thus, reflect stable and long‐term benefits of symbiosis. As such, when an insect group was found to concentrate microbes within specialized organs like mycangia, bacteriocytes/mycetocytes, or derived gut anatomies (Ebert and Brune 1997; van Borm et al. 2002; Bution and Caetano 2008; Zheng et al. 2012; Engel and Moran 2013; Ohbayashi et al. 2015), we took this as evidence for tight integration into host biology.

For our third criterion, we considered some portion of an insect family to harbour a derived, highly integrated, obligate symbiosis if their microbes were found to be transferred through host‐controlled behaviours/physiologies, or acquired through partner choice mechanisms (Table S4; Koga et al. 2012; Nalepa 2015; Salem et al. 2015; Lanan et al. 2016; Luan et al. 2018; Ohbayashi et al. 2020). Last was our ‘by extension’ criterion, where we declared the presence of a derived, obligate symbiosis when an insect family harboured symbionts deemed obligate in a closely related family.

We did not use cospeciation as a requirement for declaring an obligate symbiosis, nor did we require evidence that symbionts rely solely on an insect host (Cornwallis et al. 2023). While co‐dependence is common in obligate symbioses, several persistent associations between heteropteran insects and non‐host‐dependent microbes are crucial for host viability (Kikuchi et al. 2007; Ohbayashi et al. 2019; Hunter et al. 2022; Kho et al. 2024). Despite this unidirectional dependence, such symbioses are often ancient and specific, with long‐standing recruitment of non‐cospeciating, environmental microbes (Gordon et al. 2016; Stillson et al. 2022). With hosts harbouring large symbiont biomass within derived gut anatomies (Kikuchi et al. 2011), such symbioses are clearly derived, highly integrated, and impactful novelties.

What do less integrated, non‐obligate symbioses look like? Beyond the earlier‐mentioned reproductive manipulator symbioses, one case of non‐obligacy is exemplified by *Drosophila*‐gut microbe interactions. These fly hosts lack derived symbiont‐housing structures, and microbe‐free flies can be easily reared on diets containing yeast, a common food source (Broderick and Lemaitre 2012; Ridley et al. 2012; Consuegra et al. 2020). Like *Drosophila*, moth and butterfly larvae harbour transient bacteria in non‐specialized tubular guts. Most do not seem reliant upon gut bacteria (Hammer et al. 2017; see also Shelomi et al. 2013), despite possible exceptions (Mason et al. 2022). Mosquitoes present an additional case. Despite larval reliance on microbe‐provisioned riboflavin in some species (Trager and Subbarow 1938; Golberg et al. 1945; Wang et al. 2021), the involved microbes are transient and non‐specific, while hosts lack derived mechanisms for symbiont housing and acquisition (Coon et al. 2016).

Using this summarized knowledge and the aid of several published phylogenies (Rainford et al. 2014; Johnson et al. 2018; de Moya et al. 2019; McKenna et al. 2019; Weirauch et al. 2019), we estimate a minimum of 44 separate origins of these conspicuous, derived symbioses (Table S3; and see yellow circles on Figure S5). Importantly, this total does not include symbiont replacements after reliance was first evolved. In all but eight of these cases, symbionts were housed in specialized structures—that is, mycangia, bacteriocytes, or modified portions of the gut. Where this was not true, we found evidence for high conservation of specialized microbial taxa across host species (across‐clade ubiquity), tight integration into host physiology, and/or strong impacts on fitness, supporting an ‘obligate’ designation. Aside from the bacteriocyte symbiosis of *Bactrocera oleae* olive flies (Estes et al. 2009; Estes et al. 2018), all of our classified obligate symbioses appeared distributed across multiple species.

These varying symbioses were comprised by the following origins: one in the ancestor of cockroaches and termites; up to four within the Psocodea (chewing and sucking lice); at least four within the Hemiptera (ancestral; bedbugs; kissing bugs; Pentatomomorpha heteropterans); 12 within the Hymenoptera; over 18 within the Coleoptera; and five within the Diptera. Finer‐scale assessment of symbiont distributions with phylogeny‐based statistics will improve these efforts, as will functional and screening research within understudied insects (Klug and Kotarski 1980; Kaufman and Klug 1991).

#### 4.2.2 Obligate Symbiosis vs. Overall Herbivory

Considering diet as a correlate (Buchner 1965), we next studied the relationship between obligate symbiosis and plant‐feeding. Our literature assessment suggested that 27.3% (72/264) of predominantly herbivorous insect families harbour an obligate symbiont. The same was true for only 6.8% (37/543) of predominantly non‐herbivorous families, comprising a highly significant difference (Fisher's exact test, *p* < 0.0001). To estimate the diversity of symbiont reliant versus non‐reliant insects across diets, we used the species richness values from the GBIF database for all families in our dietary and symbiont assessments. The 264 herbivorous insect families comprise 539,063 species. Species counts from the 72 families with symbiont reliance comprise 47.8% of this total. In predominantly non‐herbivorous families, 10.2% of 495,974 species belong to a family with symbiont reliance.

Combined, this shows that highly integrated, obligate symbioses are much more common in herbivores, but that a small majority of herbivores may not require them. Some caveats arise as we consider these estimates. First, obligate symbiosis is not ubiquitous in several insect families. As such, if the number of undiscovered symbioses is low among poorly studied insects, then symbiont reliance may be less prevalent than suggested above. In addition, family‐level summaries are inherently inferior to those at the species level. The lack of diet and symbiosis studies at this finer‐grained level precludes more precise insights.

#### 4.2.3 Obligate Symbiosis vs. Specific Diets

Recognizing that plant‐based diets can vary drastically, we moved next to our tissue‐specific assessment of symbiosis. One obstacle, however, was consumption of multiple food types (e.g., distinct plant tissues) by several insect families. To navigate this, we analysed symbiont data for all families with an immature stage diet score of ‘3’ in any particular feeding category. We took such classifications as evidence of a ‘primary’ diet for the given insect taxa. Six hundred and twenty‐seven insect families met this criterion, and their contained diversity comprised 54.2% of the ~1,039,712 family‐assigned insect species in our dataset. Among this subset, families consuming phloem sap as their primary food were the most symbiotic, with 94.3% harbouring a known obligate symbiont (Figure 3). Highly integrated, obligate symbiosis was also common across families with primary diets of blood (86.7%), xylem sap (85.7%), fruit (66.7%), seeds (46.2%), and wood (33.3%). In Table S4 we highlight families from these feeding guilds without clear cases of obligate symbiosis, noting that many have received little study. We further highlight detritivorous, microbivorous, and carnivorous families that *did* have obligate symbionts as exceptions to the norm.

**FIGURE 3:**
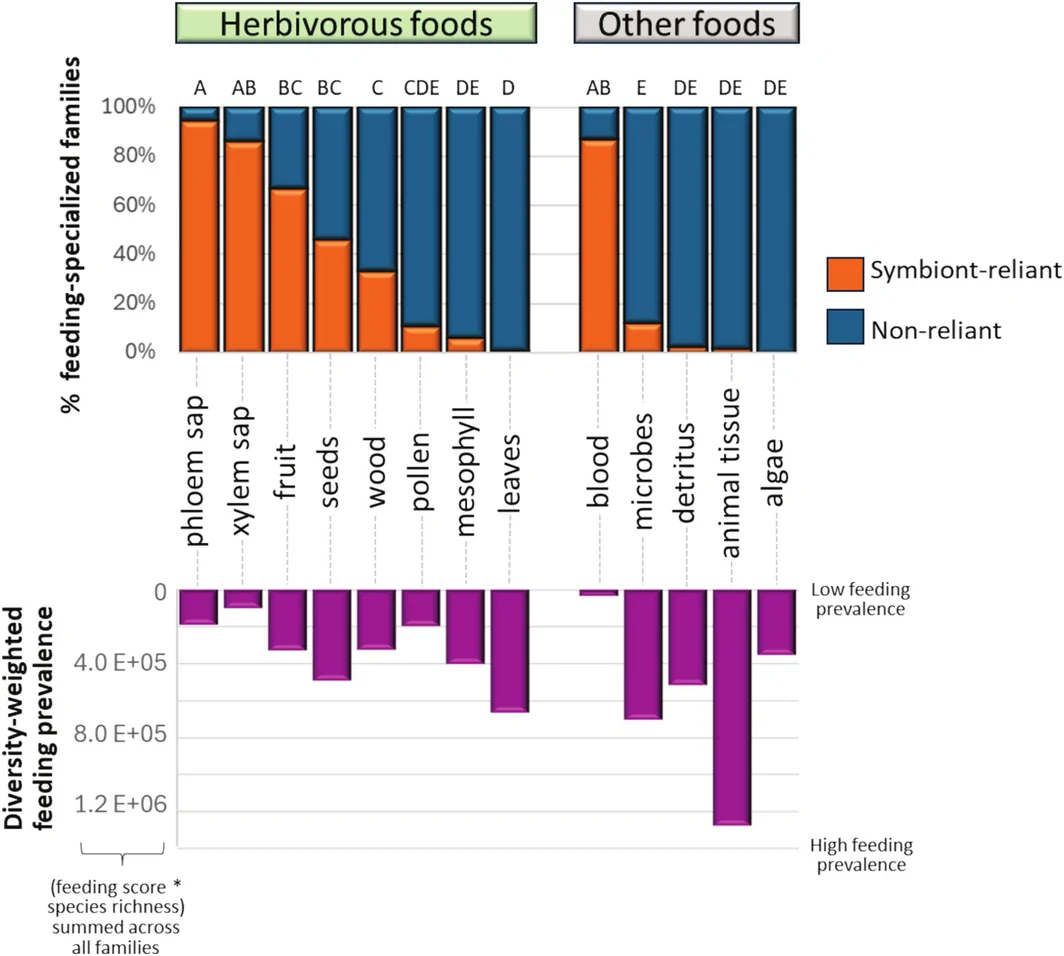
Obligate symbiosis versus juvenile insect feeding prevalence. Diversity‐weighted prevalence of feeding on plant tissues and other common foods is indicated with purple bars. Stacked orange and blue bars in the graph above reveal counts of insect families—with the given foods as primary diets—harbouring obligate symbionts. Animal tissue is the most commonly used food. Leaf tissue is the most commonly consumed herbivorous food. Letters above stacked symbiosis bars show results of pairwise Fisher's Exact tests. Primary diets with non‐overlapping letters had significantly different counts of families harbouring versus not harbouring obligate symbionts.

Links between diet and symbiosis have been recognized for decades. Early‐stage patterns were summarized in Buchner's treatise (Buchner 1965), which noted the prevalence of obligate symbiosis among insects specialized on phloem sap, xylem sap, blood, and wood. Our analyses show that seed‐ and fruit‐feeding guilds are also enriched for highly integrated, obligate symbioses, a pattern discussed in later sections. Buchner (1965) also noted some seed‐symbiosis links in beetles, but thought these were relics from times of wood‐feeding ancestry. Seed‐feeding heteroptera also harbour obligate symbionts, but do not have wood‐feeding ancestry (Table S5; Ye et al. 2022). It is possible, then, that symbioses within this guild are not historical artefacts. Their enrichment may, instead, reflect the needs for symbiosis within the seed‐feeding niche.

Bolstering the pattern of diet‐symbiosis links are symbiont losses after dietary shifts (Sudakaran et al. 2017; Bell‐Roberts et al. 2019; Hansen et al. 2024) in the phylloxera and Typhlocybinae leafhoppers. Since changing from phloem or xylem sap to parenchyma/mesophyll‐based diets (Backus et al. 2005, 2021; Powell et al. 2013; Saguez et al. 2015), these hemipterans have lost their reliance on obligate symbionts (Buchner 1965; Medina et al. 2011; Gordon 2017). Mesophyll feeding has been further identified in *Matsucoccus* scale insects (Michalik et al. 2019), the hemipteran family Miridae (Backus et al. 2007), and families from the Lepidoptera, Thysanoptera, Diptera, and Coleoptera. These insects also lack highly integrated, obligate symbioses.

Despite symbiont loss after some dietary shifts, some obligate symbioses persist. Flies from the Tephritinae have evolved distinct larval diets including flowerheads, galls, and shoot tips, without loss of their cospeciating *Stammerula* symbionts (Viale et al. 2015). Moving across the insect phylogeny to the weevils (Curculionidae), shifts to seed‐feeding led to symbiont replacements (Lefèvre et al. 2004; Heddi and Nardon 2005; Toju et al. 2010), but other weevils have otherwise kept their obligate *Nardonella* symbionts amidst shifts between leaf, stem, root, dead wood, and flower consumption (Hosokawa and Fukatsu 2010; Hosokawa et al. 2015). Nutrition should vary drastically across such diets, raising questions of whether dietary deficiencies are the only factors behind sustained maintenance of some ancient symbioses.

### 4.3 Objective 2—Obligate Nutritional Symbioses as Key Innovations?

Our review of the published literature does not suggest that highly integrated, obligate symbionts sustain most insect herbivore diversity. But they are clearly widespread across numerous taxa and enriched in particular feeding guilds. This raises two questions. First, what are the nutritive qualities of the diets eaten by insects with and without symbiont reliance? Second, where do nutritive services of obligate symbionts match specific shortcomings of derived host diets? In addressing these questions, we summarize support for the hypothesis that symbiont services are key innovations. This is the first comprehensive, insect‐wide test of this hypothesis, and a formal realization of ideas proposed in earlier studies (Moran and Telang 1998).

#### 4.3.1 Objective 2a—Nutrient Content of Insect Food Sources

Plant tissue quality is characterized by defensive secondary metabolites, anti‐feeding/anti‐digestive proteins (Broadway and Duffey 1988; Li et al. 2007; Zhu‐Salzman and Zeng 2015; War et al. 2017), and titers of accessible nutrients (Felton 1996; Wu et al. 2021). Nutrient shortfalls for some plant tissues (Jennings 1976; Dadd 1985; Fagan et al. 2002) have favoured diverse adaptations for their sufficient acquisition (Mattson 1980; Boggs and Dau 2004; Kaspari et al. 2008). Numbering among these are symbiont‐encoded solutions. If just under half (~47.8%) of all herbivorous insect species rely on highly integrated, obligate symbionts, nutritional symbiosis could be a far‐reaching fix. Which plant tissues and nutrient shortfalls favour such a remedy?

We obtained 4293 nutrient measurements across plant tissues for total nitrogen, neutral detergent fibre (cellulose, hemicelluloses, and lignin), and seven B‐vitamins, choosing this focus based on prior symbiont nutritive precedent. Beyond three heavily consulted databases (USDA: 1159 datapoints; feedipedia: 96 datapoints; voederbomen: 236 datapoints), 2802 datapoints came from 131 other sources—mostly from the peer‐reviewed literature. To eliminate redundancy, we computed species‐level averages for each tissue, yielding 3125 nutrient entries from ~1209 plant species hailing from 163 families and 53 orders. For comparative purposes, we gathered a smaller sample of B‐vitamin and nitrogen data from non‐plant foods. Summary statistics and plant‐ versus non‐plant food comparisons are presented as Supporting Information (Table S6).

Our synthesis of nutritive data provides a unified picture of insect dietary nutrition (Figure 4A; Figure S6). Poor in nitrogen were the expected foods of floral nectar, wood, xylem sap, and phloem sap, all with under 1% nitrogen as a function of dry weight. Fruit and root tissues were also fairly low in nitrogen, falling on average between 1% and 2%, while nitrogen in stems just exceeded 2%. Leaves (2.67%) and flowers (3.07%) had intermediate levels, while seeds (3.8%) and pollen had higher quantities (4.44%). Statistics on the full dataset revealed several significant pairwise differences between these tissues (Data S1).

**FIGURE 4:**
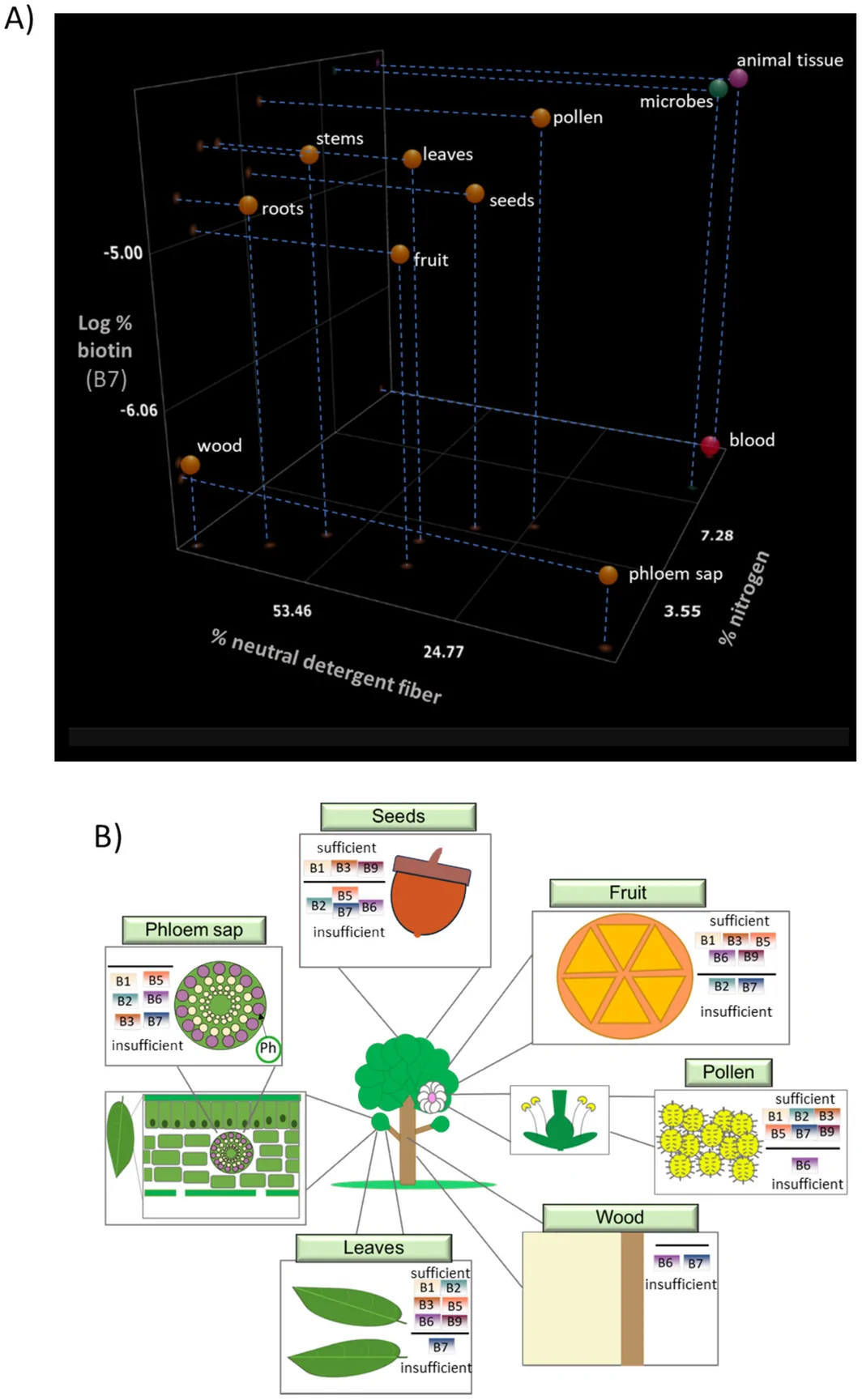
Multi‐dimensional nutritional space of several insect foods. (A) 3D scatterplot constructed on the MiaBella website (https://miabellaai.net/index.html) shows the total % dry weight averages for biotin (vitamin B7), nitrogen, and neutral detergent fibre. Food category binning methodologies are enumerated in the Data S1. Note that some foods, including xylem sap, were excluded from this graph due to a lack of one or more of the three focal nutrients. (B) Quantities of B‐vitamins in plant tissues relative to insect needs. We indicate whether averages for each tissue are above or below the average insect diet requirement threshold (‘sufficient’ vs. ‘insufficient’). Some B‐vitamin data were absent for particular tissues (e.g., none for xylem sap, only B6 and B7 for wood).

Nitrogen levels in common non‐plant foods were often higher than those seen in vascular plant tissues (Table S6), reaching 9%–13% for animal tissue and 9.8%–10.5% for blood. Bacteria had a midpoint value of 10%, while that for microalgae was 5.5%. Fungi had a mid‐point of 7.8%, displaying high variability. Ovelapping somewhat with vascular plant tissues were the nitrogen levels in detritus (1.5%–6%), diatoms (2.1%–3.2%), and lichen (0.2%–3.5%). Soil (0.1%–0.9%) and non‐vascular plants (0.5%–1.7%) were poor sources of nitrogen.

Through its digestion‐resistant and nutrient‐shielding properties, fibre presents a different challenge to plant‐feeding animals (Wertz and Béchade 2020). We summarized prior measures of neutral detergent fibre, comprising the sum of cellulose, hemicellulose, and lignin. Comparisons of this measure showed drastic variation across plant tissues (Figure 4A; Figure S6; Data S1). Among those with data across five or more species, wood was most fibre‐rich at 78.2% dry weight. Herbaceous plant stems were also fibrous (58%) compared to other tissues, which ranged from 24% (pollen) to 41.6% (leaves). With such high levels of their dietary carbon being locked up in fibre, wood feeders may be severely challenged in their quest for carbon‐based biomass and energy. Challenged along a similar axis, due instead to a scarcity of carbon, are xylem sap feeders (Andersen et al. 1995).

Like fibre and nitrogen, quantities of seven B‐vitamins differed among plant tissues (Figure 4A; Data S1). Excluded from these analyses was the eighth B‐vitamin, cobalamin (B12), which is not required by—and thus rare within—plants, some bacteria, and some insects (Wakayama et al. 1984; Halarnkar et al. 1987; Roth et al. 1996; McCutcheon et al. 2009; Jörgensen and Joergensen 2024).

#### 4.3.2 Objective 2a—B‐Vitamin Sufficiency Across Insect Diets

Nutrient levels in insect foods are best interpreted when knowing what insects need for successful development. We found data on such needs for B‐vitamins across nine insects spanning the Coleoptera, Diptera, Lepidoptera, and Orthoptera (Dadd 1985; Figure 4B; Table S7). By comparing average requirements for each vitamin to the levels in varying foods, we found thiamine (B1), niacin/nicotinate (B3), and folate (B9) to be the most generally sufficient (Table S6). In stark contrast was biotin (B7), which fell below the average insect requirement in all foods with data except pollen, prokaryotes, fungi, microalgae, and insect tissue.

Across plant tissues (Figure 4B), pollen, stems, and leaves exceeded the average requirements for six B‐vitamins. Fruit was also somewhat nutritious, having sufficient quantities for five. Standing in contrast were wood and phloem sap, which lacked sufficient quantities of every vitamin measured (*n* = 2 and *n* = 6, respectively). Seeds were also vitamin‐poor, with insufficient riboflavin (B2), pantothenate (B5), pyridoxine (B6), and B7 titers. Might this explain the above‐mentioned seed feeder/symbiosis link?

Non‐plant foods also varied in B‐vitamin sufficiency (Table S6). Blood was the poorest source, falling below the required content in all seven cases. Animal tissues were quite variable. Those from vertebrates possessed inadequate quantities of B2, B7, and B9, while crustacean tissues lacked required titers for B1, B2, B5, and B7. Insect tissues had no insufficiencies, while those from molluscs were lacking only in B7. Beyond this latter vitamin‐rich foods, some of the best sources were fungi (including yeast), eubacteria, and microalgae.

#### 4.3.3 Objective 2b—Symbiont Enrichment vs. Diet/Nutrition

The above results suggest dietary nutrition is often related to obligate symbiosis. Most likely to harbour an obligate symbiont were insects from families with (A) phloem sap, (B) xylem sap, (C) fruit, (D) seeds, or (E) wood as primary diets. These foods had low or relatively low titers of (A) nitrogen/all six measured B‐vitamins; (B) nitrogen (with unknown B‐vitamin content); (C) nitrogen/vitamins B2, B7; (D) vitamins B2, B5, B6, and B7; and (E) nitrogen/vitamins B6 and B7 (with no measures for other vitamins). Carbon accessibility is also limited for wood diets (E), due to high fibre content.

In their 2023 study, Cornwallis et al. found insects with low dietary levels of B5 and B9 were more likely to harbour an obligate symbiont. Our analyses—across more insect families and with more precise plant tissue diet definitions—showed similar, significant trends for all seven B‐vitamins (Figure S7; Data S1). This trend vanished for vitamin B9 when data from blood were excluded, due possibly to the absence of B9 measures for phloem sap and wood. In a further set of these GLM analyses, we found significantly negative correlations between obligate symbiosis versus nitrogen, but no correlation for fibre (Figure S8; Data S1).

#### 4.3.4 Objective 2c—Symbiont Metabolism/Function vs. Diet/Nutrition—Overview

Obligate symbionts vary in their in vivo impacts on host physiology and ecology. So in assessing roles of symbiosis in key innovations, one must relate symbiont function, not just presence, to insects' dietary shortcomings. To do this, we studied genome‐inferred obligate symbiont metabolism (Tables S8–S11), complementing this focus on predicted function with results from direct in vivo study (Data S1). Methods and rationale behind this work are outlined in Box 3.

BOX 3 **Metabolism/function of obligate microbiomes (methods & rationale).**

Symbiont functions considered in this review centred on the capacities for fibre catabolism, nitrogen fixation, nitrogen recycling, amino acid (AA) and B‐vitamin synthesis (Figure 1B), and the abilities of such metabolism to benefit the host. In an emphasis on in vivo study, we performed a Google Scholar‐based literature search using key words described in the methods section of Data S1. We ignored papers on cultivated microbes unless they clearly came from a known, non‐transient symbiont. Notes were taken on each paper and summaries were made at the insect species level.

Across 277 primary research articles we found in vivo evidence for possible symbiont function in 158 insect species. These belonged to 44 families from eight orders. Results from insect species with direct evidence for symbiont function were summarized in the main manuscript. These direct approaches tested host nutritional phenotypes and included nutrient quantification, isotope labelling studies, and ‘nutrient sparing’ tests of symbionts' abilities to eliminate dietary needs. Many species had only indirect evidence (e.g., enzymatic assays, transcriptomics, proteomics). These were not considered in our tallies.

In a parallel effort, we inferred symbiont capacities for the above‐described metabolism by searching for 400 involved KEGG orthologs (KOs) across the genomes of obligate symbionts (Figure S9). After identifying the relevant genes/proteins from each symbiont, and considering the entirety of each obligate microbiome, we expressed results from fibre degradation, nitrogen fixation, and nitrogen recycling searches as the number of unique KOs encoded. For the biosynthesis of 10 essential AAs, cuticle‐enriched chorismate and tyrosine, and seven B‐vitamins, we instead assessed ‘pathway completeness’—that is, the proportions of each pathway encoded (Data S1).

Symbiont genomes were identified within the IMG and NCBI databases and were used when the literature from our obligate symbiont review suggested we had full coverage of a host's obligate microbiome. In many cases, these symbiotic systems are simple and analysis of one or a few isolate genomes reveals the metabolic potential of the obligate microbiome. For highly diverse, highly integrated gut microbiomes, we used metagenomes for such inference.

Through these efforts, we obtained 102 (meta)genomic datasets each equating to a complete symbiont genome or deeply sequenced metagenome. These datasets spanned obligate microbiomes across 77 insect species representing 23 of 44 plausibly separate origins of highly integrated symbiont dependence (Table S3; Table S8). To minimize pseudoreplication in our statistical analyses, we averaged gene/protein search results across hosts with the same diet and shared ancestral symbiosis. When symbionts were replaced after the origins of an ancestral obligate symbiosis, we treated such reinvented symbioses as separate replicates due to their unique potential to retain, lose, or acquire genes/functions. In most of our analyses on symbiont metabolism versus dietary nutrition, we focused on the genomes of ancient endosymbionts, deemed to be those where the average 16S rRNA identity versus the closest free‐living microbe was < 95%. This minimized effects of superfluous gene content in young symbionts with less streamlined genomes (Box 4; Figure S10). It also minimized effects from environmentally acquired symbiotic microbes, whose genomes are further shaped by life outside of insect hosts.

To study the match between nutrient‐poor diets and symbiont metabolism, we focused several analyses on older endosymbionts. Genomic streamlining has jettisoned thousands of genes in specialized, obligate symbionts (McCutcheon and Moran 2011), with a steady drop in genome size for some time post‐domestication (Figure S10). If less integral genes are lost gradually, then the genomes of younger symbionts may not yet reflect the core needs for life under their symbioses. Supporting this was our finding that genes for essential amino acid (AA) synthesis are more abundant in young versus old symbionts among insects with nitrogen‐rich seed and blood diets (Table S12; Box 4). This trend was not seen for symbionts of insects on nitrogen‐poor phloem sap diets, reflecting the persistent need to make AAs on diets that lack them. We took all microbiomes with an average of < 95% 16S rRNA identity versus free‐living microbes to be ‘ancient’, using genomes from these symbioses for most of our analyses below.

BOX 4 **Pitfalls of testing function vs. diet using genome‐inferred symbiont metabolism.**

Genome‐encoded metabolism reveals potential for host‐level symbiotic function. But superfluous genome content of recently domesticated symbionts could obscure expected matches between symbiont metabolism and dietary nutrient shortfalls. Host‐dependent, obligate symbionts from low‐diversity microbiomes undergo genome reduction over time, losing non‐essential genes due to mutation, drift, and altered selection in their specialized habitat (McCutcheon and Moran 2011; Figure B2). When symbionts are environmentally acquired or recently domesticated microbes, their gene content may not (yet) be streamlined for function under symbiosis. Statistical analyses on genome‐encoded metabolism versus dietary nutrition might, thus, be more insightful if limited to older, host‐specialized symbionts.

To test this, we examined the relationship between time since symbiont domestication versus biosynthetic pathway completeness, confining our focus to the 10 essential amino acid (AA) pathways. We used percent 16S rRNA identity to the closest non‐symbiotic relative as a rough proxy for time since symbiont domestication (Moran et al. 1993; Degnan et al. 2004). Percent identity was assessed using BLASTn searches against the nr/nt database for one representative, NCBI‐derived 16S rRNA sequence from each symbiont species in our genomic dataset. While computing a single 16S rRNA divergence score was impractical for highly diverse metagenome‐sampled symbioses, we included data from low‐diversity, multispecies microbiomes by taking the average 16S rRNA divergence across the 2–3 obligate symbionts of the relevant host insects.

**FIGURE B2:**
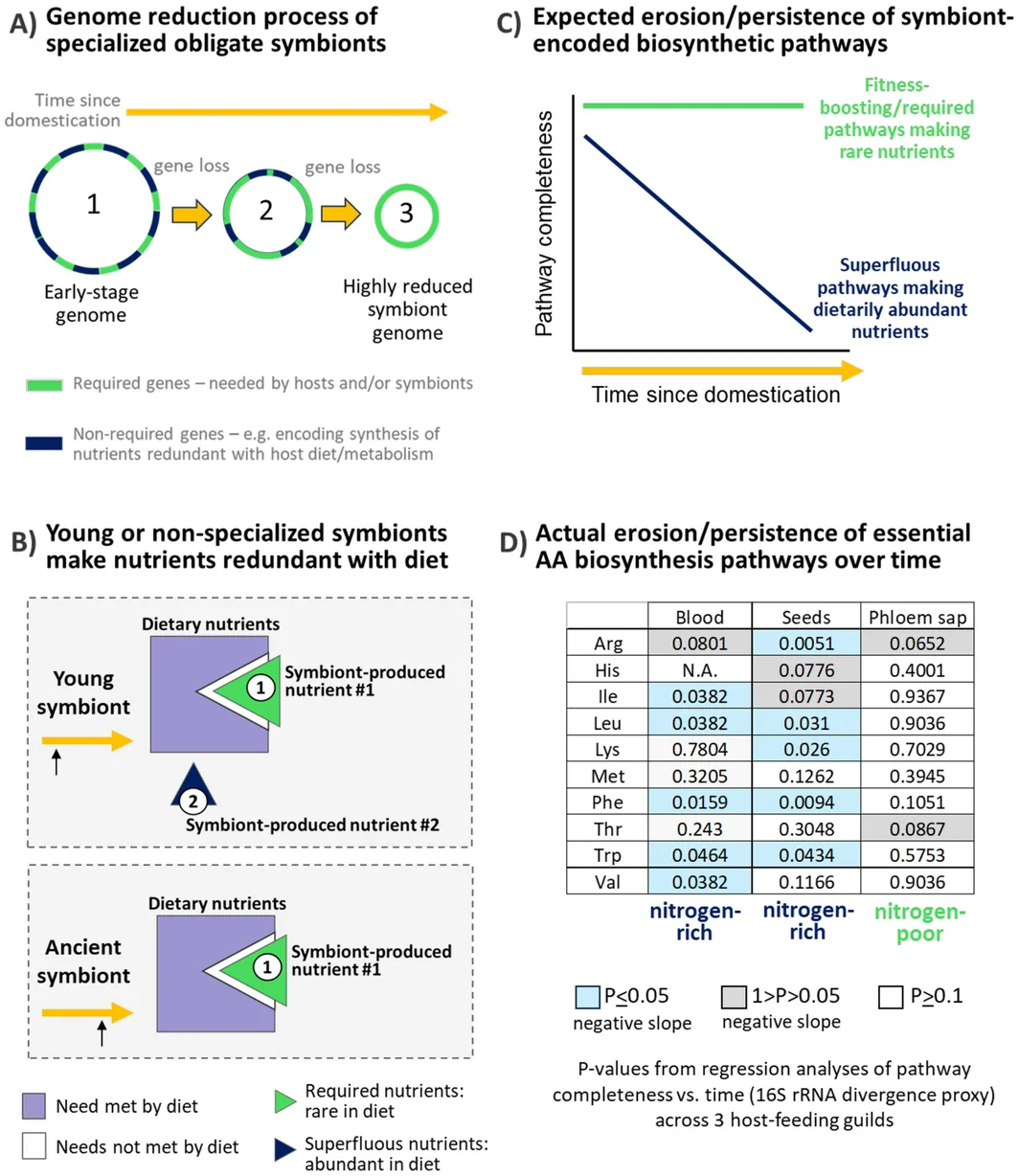
Genome content of ancient, obligate microbiomes may best predict persistent selective pressures unique to each diet. As a case study, we illustrate how obligate symbionts of insects with nitrogen‐rich diets typically lose amino acid biosynthetic genes over time (approximated by % 16S rRNA gene divergence from free‐living bacteria). This is not seen for symbionts in insects with nitrogen‐poor diets.

Due to their relatively robust sample sizes, we focused our analyses on the obligate microbiomes of phloem‐, blood‐, and seed‐feeders. Diets for insects in the former guild are very poor in nitrogen and AAs. Those from the latter two are relatively rich. For symbionts of these latter hosts, linear regression statistics showed that increasing time since symbiont domestication predicts decreasing pathway completeness for several essential AAs (Table S12; Figure B2). Among obligate symbionts of seed feeders, this pattern was significant for the lysine, arginine, leucine, phenylalanine, and tryptophan pathways. Symbionts of blood feeders showed similar, significant trends across the latter three pathways, and those for isoleucine and valine.

Eventual loss of most AA pathways suggests the perils of inferring integral symbiont function with genomes from young or environmentally acquired symbionts—these may retain capacities for functions not mandated by host diet. In contrast to these findings, AA pathway completeness did not change with increasing 16S rRNA divergence for symbionts of insects consuming nitrogen‐poor phloem sap. Retention of AA synthesis genes in some of the world's most streamlined bacterial genomes reveals strong, persistent selection for this capacity in the symbiotic habitat, supporting expectations that symbionts have long helped hosts to overcome dietary shortfalls.

#### 4.3.5 Objective 2c—Genomic B‐Vitamin Results

We first tested the relationship between the completeness of symbiont‐encoded B‐vitamin synthesis pathways versus log‐transformed dietary B‐vitamin levels (Table S9). Among regression analyses for all seven pathways, B5 and B7 showed the expected negative relationship for the ancient symbiont‐only dataset (Figure 5A; Figure S11; B5: linear regression equation: *Y* = −0.2938 × *X* − 0.5592; *R*‐square = 0.274; *p*‐value = 0.0072; B7: linear regression equation: *Y* = −0.1555 × *X* − 0.3765; *R*‐square = 0.1789; *p*‐value = 0.0395).

**FIGURE 5:**
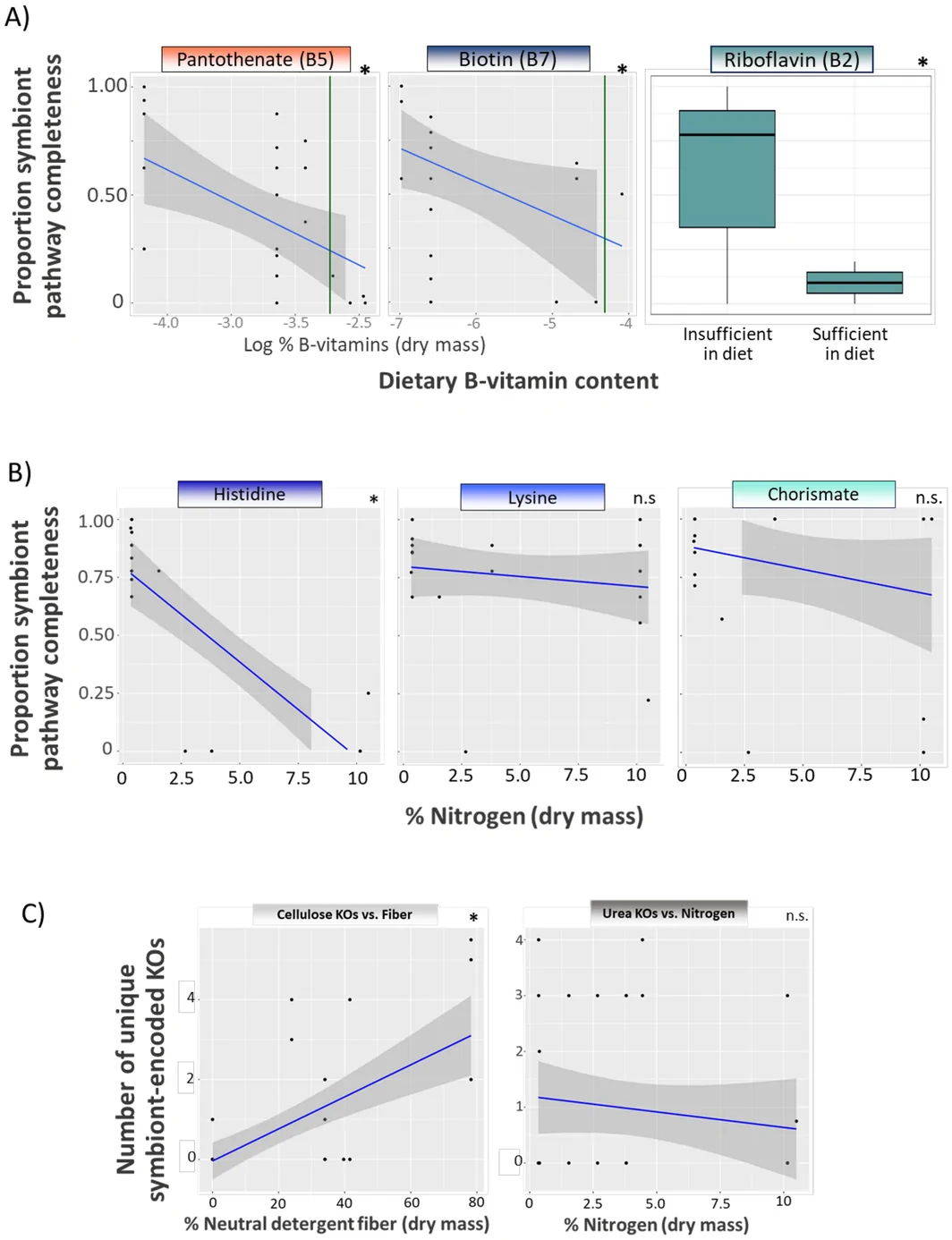
Nutritional quality of insect diet predicts symbiont metabolism. Analyses here focus on nutrient content of individual insect foods and the genomic capacities of obligate symbionts from insects feeding primarily on these foods. (A) Obligate microbiomes encode more of the vitamin B5 and B7 biosynthesis pathways with decreasing amounts of these vitamins in their diet—according to linear regression. All other regressions tests were non‐significant. Right side of panel A—*t*‐test revealed greater vitamin B2 pathway completeness in symbionts of insects with diets lacking sufficient B2 quantities, suggesting dietary B2 (riboflavin) also predicts symbiont metabolism. Not shown, but also significant, were *t*‐test results for B5 and B6. Green vertical lines in the first 2 panels reveal two of these dietary thresholds. (B) Obligate microbiomes encode more of the histidine biosynthesis pathway with decreasing amounts of dietary nitrogen, according to linear regression (a finding significant for many other essential AAs). The same relationship did not exist for lysine or for tyrosine. The latter is a precursor and building block of the insect cuticle (Kramer and Hopkins 1987). (C) Obligate microbiomes encode more unique cellulose‐degrading proteins (KEGG orthologs, or KOs) with increasing amounts of dietary fibre, but do not show shifts in urease‐encoding KOs across varying dietary nitrogen. Asterisks to the upper right of each panel indicate significance, while n.s. indicates non‐significant relationships between the *x*‐ and *y*‐variables. Note that panels A and B focused only on datasets without metagenomes and without symbionts having > 95% 16S rRNA sequence identity to a free‐living bacterium. This smaller dataset excluded many extracellular gut symbioses. With these being important in digestion, and possibly nitrogen recycling, we show analyses for the full dataset (i.e., including metagenomes and recently derived symbionts) in panel C. For all panels with regression lines, the position of each datapoint on the *y*‐axis represents the pathway completeness for each analysed microbiome from each insect with the given dietary input (*x*‐axis). Grey shaded regions surrounding each regression line represent 95% confidence intervals.

These findings support the expectation that B‐vitamin synthesis by obligate symbionts is favoured by vitamin‐poor diets (Douglas 2017). We tested this further through 1‐tailed *t*‐tests. For these analyses we coded B‐vitamin levels for each food as being above (1) or below (0) the average dietary requirement, relating this to pathway completeness of ancient, obligate microbiomes from insects specialized on such foods (Figure 5A; Figure S12; Table S10). We saw the expected, significant relationships for B2, B5, and B6. Ancient obligate microbiomes from insects with insufficient dietary B2 encoded 62.0% of the corresponding biosynthetic pathway, compared to only 9.7% for those of insects with sufficient dietary access (*p* = 0.017). For B5, these averages were 50.0% and 3.9%, respectively (*p* = 2.26E‐06). For B6, they equaled 48.4% and 8.0% (*p* = 0.002).

Datapoints from insects with insufficient juvenile dietary B‐vitamins came mainly from symbioses of seed‐, phloem sap‐, and blood‐feeders. Moving beyond our pathway completeness calculations, we averaged gene presence/absence at each step of the seven B‐vitamin pathways across separately derived, obligate symbioses within these feeding guilds (Figure 6; Table S13). Out of 140 pathway‐by‐symbiosis combinations, only 18 showed pathway completeness. In 31 cases, pathways were missing genes for just one or two steps. For partially encoded pathways, there was occasional convergence in the identities of missing genes. This was seen for those encoding step 4 in the B2 pathway; steps 1–3 for that of B3; step 1 for B6 synthesis; steps 1 and 10–14 for the B7 pathway; and steps 2′, 4, and 5 for B9.

**FIGURE 6:**
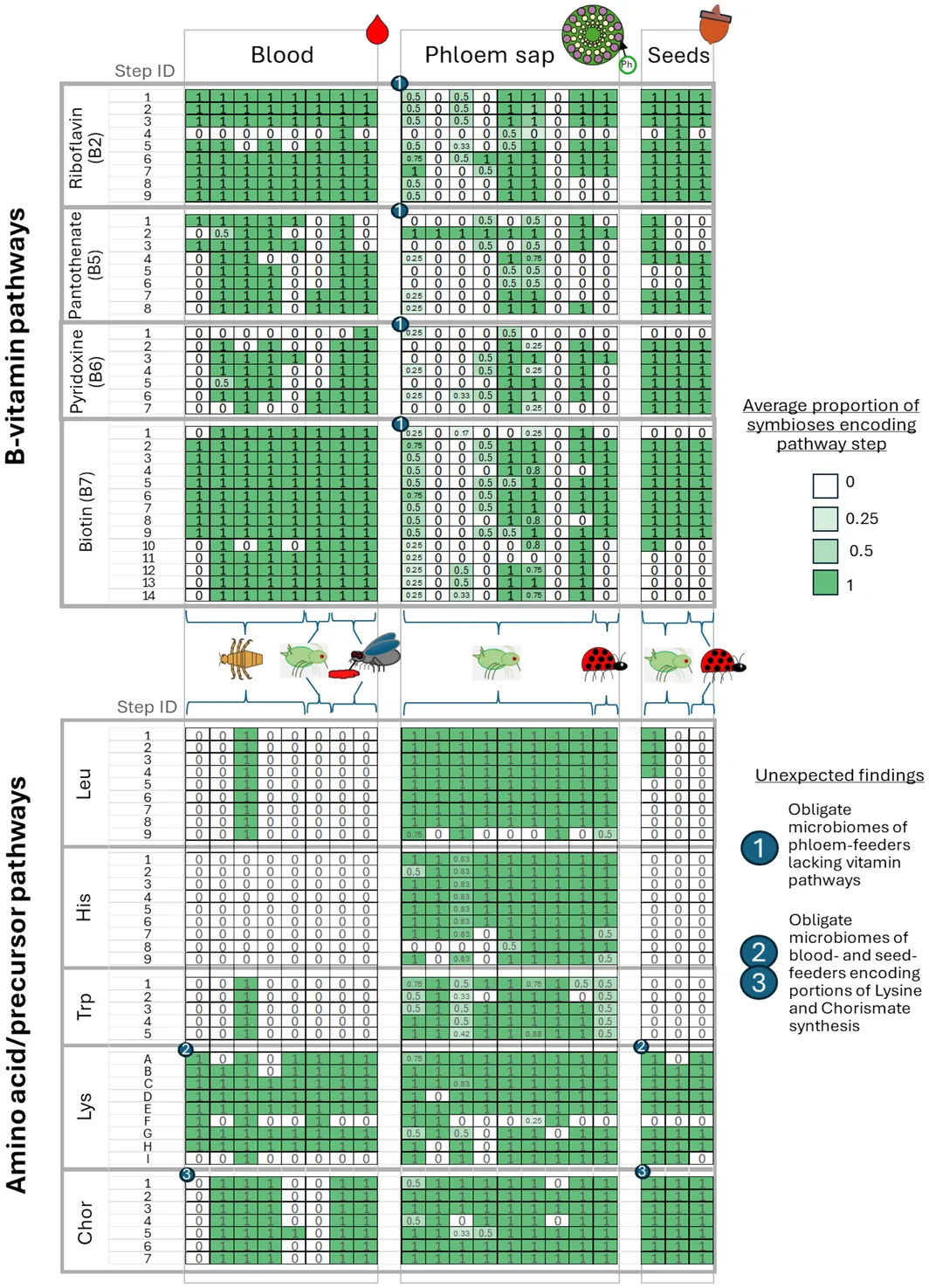
Variable capacities for B‐vitamin and amino acid synthesis across ancient, obligate microbiomes—expected versus unexpected trends in light of diet. Each column corresponds to a uniquely evolved or reinvented symbiosis. Information linking these to host species names and to names of symbionts can be found in Table S13. Rows correspond to steps in biosynthetic pathways. When there were multiple sub‐pathway alternatives, we chose the most commonly encoded pathway. Numbers in cells indicate the average gene presence (‘1’) versus absence (‘0’) per step, across distinct, non‐pseudoreplicate obligate microbiomes. For example, one column averages results from all obligate aphid microbiomes with a *Buchnera* symbiont and any co‐obligate symbionts. Insect cartoons refer to symbioses in lice (Psocoptera), varying Hemiptera, Diptera (Hippoboscidae), and Coleoptera. Three patterns that violate expectations from dietary nutrient content include the typical lack of B‐vitamin pathway encoding by obligate microbiomes of phloem feeders, tendencies to encode most of the lysine pathway in blood‐ and seed‐feeders, and similar tendencies toward chorismate synthesis for symbionts in hosts from these same two feeding guilds.

Most symbioses from blood‐ and seed‐feeders encoded large majorities of B2, B5, B6, and B7 pathways. Among the phloem‐feeders, this was true for only *Ishikawaella* symbionts (from *Megacopta* stink bugs) and two reinvented symbioses from aphids. Vitamin‐synthesis is, thus, under‐represented for six of the nine separately evolved obligate microbiomes in this feeding guild. This raises intriguing questions. First, phloem is more vitamin‐poor than seed tissue (Table S6). Why is B‐vitamin synthesis more enriched among obligate symbionts of seed‐feeders? Second, if we view obligate symbiont nutritive services as niche‐accessing membership cards, has one of the original access‐granting mechanisms (B‐vitamin provisioning) often expired in the phloem‐feeding guild? If so, what has replaced them?

#### 4.3.6 Objective 2c—Experimental B‐Vitamin Results

Because symbionts require B‐vitamins for their own metabolism (Serbus et al. 2017), support beyond genomics is required to prove B‐vitamin provisioning. Such evidence is lacking for most symbioses. But experiments have shown some symbionts can boost host B‐vitamin economies (Serrato‐Salas and Gendrin 2023). From our synthesis of the experimental literature, we found evidence from the predominantly phloem‐feeding Auchennoryncha/Sternorrhyncha lineage to be most extensive. Positive results were obtained from planthoppers (B7), aphids (B2, B7), and whiteflies (B2, B3, B6, B7, B9; Figure S13a) across their varying reinventions of a shared ancestral symbiosis (Dadd et al. 1967; Ehrhardt 1968; Nakabachi and Ishikawa 1999; Blow et al. 2020; Ju et al. 2020; Wang, Ren, et al. 2020). In several of these cases, facultative symbionts contribute some of the documented B‐vitamin provisioning or precursor biosynthesis (Figure S14). This may partially explain the variable retention of B‐vitamin pathways across obligate symbioses within the phloem‐feeding guild.

Beyond these examples we found no published experimental B‐vitamin studies across other insects with separately evolved phloem‐feeding diets. Future effort is certainly warranted, given genomic findings of modestly complete B2, B5, B6, B7, and B9 pathways in symbionts of *Megacopta* stinkbugs (Hemiptera: Heteroptera: Pentatomomorpha), and near‐complete B2 and B7 pathways in symbionts of reed beetles (Table S13).

Among the seed‐feeding insects, obligate symbionts provide B‐vitamins in at least three lineages (Figure S13b). Experimental studies in the first lineage—the Ptinidae family (formerly Anobiidae)—come from two beetles consuming grains, spices, flour, and several stored products (Ponce et al. 2023). These hosts receive five‐to‐seven B‐vitamins from intracellular, yeast‐like *Symbiotaphrina* symbionts (Pant and Fraenkel 1950, 1954; Jurzitza 1969, 1970; Martinson 2020). In the second lineage (Salem et al. 2014), findings of higher pooled B‐vitamin titers in the presence of obligate gut symbionts support direct contributions of bacteria to Pyrrhocoridae fire bug hosts (Hemiptera: Heteroptera: Pentatomomorpha). Full community metagenome analysis was lacking for this symbiosis. But B‐vitamin synthesis is encoded across several reinvented symbioses in other seed‐feeding Pentatomomorpha (75.5%–100% pathway completeness for B2‐B9).

The third B‐vitamin provisioning example from a symbiont of seed‐feeders comes from the grain weevil *Sitophilus oryzae* . Harbouring a reinvented symbiosis with a *Sodalis* species taking over for ancestral *Nardonella* (Lefèvre et al. 2004), this insect's symbiont exhibits 57.1%–100% pathway completeness for all seven B‐vitamins versus 0% in *Nardonella* from other weevils. Direct experimental evidence suggests *Sodalis* provides hosts with vitamins B1, B2, B5, and B9 (Wicker 1983). *Curculioniphilus*—another usurper of the ancestral *Nardonella* symbiosis (Conord et al. 2008; Toju et al. 2013)—lives with a different group of seed‐feeding weevils, encoding 30.0%–88.9% of the seven B‐vitamin pathways. Diets of *Nardonella‐*harbouring weevils are diverse, but include foods such as stems and microbe‐enriched wood (Lefèvre et al. 2004). With stems exceeding insects' B‐vitamin requirements for all but B7, and with microbes being an especially rich source of these nutrients (Table S6), at least some *Nardonella* hosts should not lack for B‐vitamins. The corresponding absence of B‐vitamin pathways in *Nardonella* genomes, thus, likely mandated new symbioses when hosts shifted to seed‐feeding.

Given the dietary shortcomings of seeds shown through our literature summary, obligate symbiont B‐vitamin provisioning is likely a key innovation for several insect seed feeders. This same service is likely a key innovation across blood feeders, as well (Manzano‐Marín et al. 2015; Duron and Gottlieb 2020; Cornwallis et al. 2023). Adding to remarkably conserved biosynthetic capacities for all but B1 and B3 across obligate blood feeders' microbiomes (Figure 6), experimental evidence directly demonstrates B‐vitamin provisioning within the Cimicidae (bedbugs; Hemiptera), Glossinidae (tsetse flies; Diptera), Pediculidae (lice; Psocodea), and Reduviidae (kissing bugs; Hemiptera) (Puchta 1955; Eichler and Schaub 1998; Michalkova et al. 2014; Nikoh et al. 2014; Snyder and Rio 2015; Gilliland et al. 2023).

Several curiosities were found in our review of B‐vitamin provisioning research. For example, while neither *Drosophila* fruit flies nor *Aedes* mosquitoes have evolved highly integrated symbioses like the ones considered here, both acquire B‐vitamins from transient gut bacteria (Trager and Subbarow 1938; Golberg et al. 1945; Sang 1956; Coon et al. 2016; Sannino et al. 2018; Consuegra et al. 2020; Wang et al. 2021). Given the typical vitamin‐enriched, yeast‐based diets of *Drosophila* larvae (Table S6), B‐vitamin provisioning by their gut bacteria may be important under nutrient‐poor conditions (Storelli et al. 2018). Like *Drosophila*, *Camponotus* ‘carpenter’ ants may acquire B‐vitamins from facultative, gut‐dwelling acetobacteria, with some assistance from obligate *Blochmannia* (Mankowski and Morrell 2014; Brown and Wernegreen 2019). Long‐thought to be omnivores, some research suggests these ants feed lower on the food chain (Davidson et al. 2003). Shifting paradigms in presumed diet have also unfolded for cockroaches. While they are often described as omnivores, plant‐based detritus may comprise most of their food (Nalepa et al. 2001; Bell et al. 2007). Precise measures of cockroaches' dietary B‐vitamins remain important in explaining their symbiont‐derived B‐vitamin acquisition (Noland et al. 1949). The abundance of nutrient‐rich microbes on most detritus, and the rarity of obligate symbiosis in many detritus feeders (Figure 3) raises questions on whether or why this service could be a niche‐required key innovation.

#### 4.3.7 Objective 2c—Genomic Amino Acid Results

Like B‐vitamins, pathways for biosynthesis of 9–10 essential AAs are absent from animal genomes. In light of strong correlations between AA versus nitrogen content in insect foods (Table S14; Figure S15), might links between low nitrogen and obligate symbiosis (Figure S8) suggest frequent AA‐provisioning by symbionts of some herbivorous insects? In assessing metabolic potential, we note that average pathway completeness for the 10 essential AAs, plus cuticle‐enriched tyrosine and chorismate, ranged from 47.6% to 89.0% across all obligate microbiomes. Tryptophan and arginine pathways were the least encoded, while those for chorismate and lysine were the most highly represented.

Using nitrogen as a proxy for AA quantities, linear regression revealed nine significantly negative correlations across the 12 analysed pathways (Table S11). So, as nitrogen rises in insect diets, we see a decline in the completeness of AA biosynthesis pathways encoded by obligate microbiomes. Results were strongest for ancient symbiont‐only datasets excluding higher diversity metagenome‐sampled gut microbiomes (Figure 5B; Figure S16). Lacking significant correlations with nitrogen in this pruned dataset were the frequently encoded tyrosine, chorismate, and lysine pathways. Ancient, specialized symbionts from hosts with two fairly nitrogen‐rich diets—seeds and blood—frequently encoded portions of these latter two pathways, much like their counterparts from phloem feeders (Table S13; Figure 6).

Similarities among symbionts from the seed, blood, and phloem sap feeding guilds were lacking across the remaining nine essential AA pathways (Table S13; Figure 6). Among phloem feeders, all but arginine trended toward 50%–100% completeness across ancient, obligate microbiomes. This was rarely true for the obligate symbionts of blood and seed feeders. Exceptions were seen for short threonine and methionine pathways, where the first two steps overlap with those from the lysine pathway.

Failure of tyrosine and chorismate pathway completeness to decline with increasing dietary nitrogen (Wierz et al. 2024; Figure S16) is made all the more intriguing by our finding that nitrogen predicts tyrosine levels in the diet (Figure S15; Table S14). Given the importance of these molecules, and their derivatives, in the insect cuticle (Arakane et al. 2016), there is clearly high demand. But we cannot yet link dietary shortcomings to the known or suspected cases of symbiont‐supported cuticle building (Box 5). Lysine synthesis showed a similar pattern, with symbionts encoding much of this pathway, even on lysine‐rich diets (Figure 6). Aside from possible exceptions among pollen‐feeding social insects, our review of symbiont function revealed there is little precedent for symbiont lysine provisioning on lysine‐/nitrogen‐rich diets. It is of further curiosity that most symbionts from insects consuming lysine‐rich blood lack the final step of lysine biosynthesis, in which meso‐2,6,‐diaminopimelate (mDAP) is converted to lysine (Figure 6). By stopping short, these symbionts may be feeding not the insect lysine economy but, instead, their own needs for peptidoglycan (Garde et al. 2021; Figure S9).

BOX 5 **Defined vs. undefined symbiotic key innovations.**

*Context‐dependence of obligate symbiosis*

Like other animals, insects lack abilities to make diet‐limiting nutrients, including B‐vitamins and essential AAs. Due to the multi‐gene nature of the involved biosynthetic pathways, most of these losses appear irreversible, being beyond repair by lateral gene transfer (Husnik and McCutcheon 2018; but see Craig et al. 2008). Despite variable nutrition across plant taxa and variable insect needs (Dadd 1985; Fagan et al. 2002; Woods et al. 2004; Chown et al. 2007; Clavé et al. 2022; Naug 2024), the rare adoption of xylem and phloem sap diets—with their remarkable scarcity in B‐vitamins and/or essential amino acids—has undoubtedly required provisioning of these nutrients by highly integrated symbioses (Bell‐Roberts et al. 2019). Here we show that the genome content and functions of the oldest, most specialized symbionts from these relationships are well matched to meet at least the AA needs of these insects.

Similar findings suggest services of obligate symbionts in insects consuming seeds, wood, and fruit are key innovations, given the nutrient‐poor status of these tissues. But what should we make of possible symbiont‐independence within such feeding guilds? And how should we view reliance on symbionts among insects with nutrient‐rich diets, like pollen?

While hard to quantify (but see Bernays and Klein 2002; Akman Gündüz and Douglas 2009), the ‘need’ for obligate symbionts may be context‐dependent. Factors shaping these contexts possibly include:

1. Variable possession of insect‐encoded genes

Thanks to ancestral retention and lateral gene transfer, many insects encode one or more fibre‐degrading genes. These aid insects in breaching or digesting the plant cell wall (Treves and Martin 1994; Calderón‐Cortés et al. 2012; Wybouw et al. 2016; Shin et al. 2026). In some lineages, the presence or absence of these genes may dictate the necessity for obligate symbiosis (McKenna et al. 2019; Ebert et al. 2021; Kirsch et al. 2025). Did a long history of parasitoidism and predation in groups like the Hymenoptera enable loss of such genes? And did a reduced genomic repertoire make symbiosis the quickest way to exploit pollen, leaves, and detritus within descendant Hymenoptera, including some bees and ants?

2 Developmental pre‐adaptations

Do other variable genes shape the likelihood of evolving symbiosis? For example, are some insects predisposed to evolve bacteriocytes due to flexible embryonic cells with a capacity for convergent re‐wiring (Rafiqi et al. 2020; Alarcón et al. 2022)? Might this make it easier to domesticate symbionts? Could this, in turn, enable more frequent outsourcing for non‐insurmountable challenges?

3 Behaviour

By increasing partner fidelity, coprophagy may lessen selection favouring selfish strategies among faeces‐acquired microbes. Did this facilitate the origins of some cockroach symbioses (Nalepa et al. 2001)? Transmission behaviours enabled by eusociality may similarly achieve faster alignment of host and microbe interests (Foster and Wenseleers 2006; Salem et al. 2015; Onchuru et al. 2018; Rose et al. 2023). Do such behaviours make ‘outsourcing’ more likely for challenges quite often addressed through insect‐encoded solutions?

4 Size

Symbiont‐mediated plant fibre breakdown often functions as an anaerobic, fermentative process. Certain types of digestive symbioses may thus require the presence of a large gut chamber, with anoxic luminal regions sufficiently beyond the reaches of oxygen diffusion (Brune et al. 1995; Lucey and Leadbetter 2014; Zheng et al. 2017). If gut size matters, this could hinder the origins of digestive symbioses below particular body size thresholds. A mitigating factor in larger insects could be trade‐offs between host mobility versus large gut biomass required to sustain digestive symbioses (Katzin and Kirby 1939; Ebert et al. 2021).

*Undefined niches among insects with cuticle‐building symbioses?*

Insects with very diverse and sometimes rich diets harbour cuticle‐building symbioses, characterized through symbiont provisioning of tyrosine, phenylalanine, or chorismate (Hosokawa et al. 2015; Morera‐Margarit et al. 2019; Duplais et al. 2021; Wierz et al. 2024). This service by obligate symbionts may be among the most common across the insects. For several symbioses it seems to be the *raison d'être*, with symbionts having lost most other types of metabolism with potential host utility (Anbutsu et al. 2017; Engl et al. 2020; Jackson et al. 2022).

The diversity of diets consumed by insects with cuticle‐building symbioses, and the seemingly high abundance of phenylalanine/tyrosine in the diets of some affected insects (Figure S18) raise questions on the drivers of such symbioses. Well‐developed cuticles protect against predators, pathogens, and desiccation, suggesting ecological perks of these symbioses (Engl et al. 2018; Kanyile et al. 2022, 2023; Lv et al. 2025). But are symbiont‐supported cuticles more effective or economical than those produced entirely by hosts? Is symbiont cuticle‐enhancement a more evolvable strategy than endogenous enhancement? If so, why are obligate cuticle‐building symbioses only found in some insect orders? The answers to these questions have bearing on the drivers of symbiosis among thousands—if not tens of thousands—of ant and beetle species, and beyond.

*Undefined niches within detritivores and herbivorous social insects?*

Symbioses supporting fibre catabolism, nitrogen fixation, nitrogen recycling, B‐vitamin provisioning, or essential AA provisioning have been reported in some detritivores and in eusocial insects with omnivorous or pollen‐based juvenile diets (Bignell 1977; Breznak and Switzer 1986; Bayon and Mathelin 1980; Mankowski and Morrell 2014; Ayayee et al. 2016; Kešnerová et al. 2017; Hu et al. 2018; Zheng et al. 2019; Hansen et al. 2020). In light of the general trends shown in this review, these symbioses initially seem superfluous. Do we require a more precise understanding of diet to make sense of these patterns (Cummins and Klug 1979; Sinsabaugh et al. 1985; Génissel et al. 2002; Davidson et al. 2003; Weiner et al. 2010; France 2011; Šustr et al. 2014)? Or are the above speculations on eusociality and its potential to increase symbiotic evolvability more pertinent (Nalepa 2020; Mee and Barribeau 2023)? Another question emerges for eusocial insects—are symbioses there simply to meet the demands of the insect body plan? Or are there emergent colony‐level phenotypes amenable to symbiont support, suggesting niches unique to this lifestyle (Anderson and McShea 2001)?

#### 4.3.8 Objective 2c—Experimental Amino Acid Results

Provisioning of synthesized AAs is a well‐documented symbiont service across insect lineages. Our literature search found direct evidence in support of this across 28 insect species compared to 20 species for B‐vitamins. Retention of AA pathways by ancient obligate symbionts may, thus, often signify some host‐level benefit (Box 4).

Species with direct evidence of symbiont AA provisioning represented ~11 separate origins of obligate symbiosis, and two instances of facultative symbiosis (see Kwak et al. 2025 for a more recent example of the latter). In three separate insect lineages, direct evidence supported only chorismate, tyrosine (non‐essential), or phenylalanine (essential) provisioning, suggestive of cuticle‐building symbioses (Wicker and Nardon 1982; Vigneron et al. 2014; Anbutsu et al. 2017; Kiefer et al. 2021). In other instances, varying essential AAs are provided by symbionts of seed‐, stored product‐, and wood‐feeding beetles from the family Ptinidae (Pant et al. 1960; Jurzitza 1969, 1970; Bismanis 1976), and omnivorous or pollen‐feeding eusocial insects (i.e., ants and corbiculate bees—Feldhaar et al. 2007; Hu et al. 2018; Zheng et al. 2019; Duplais et al. 2021). Essential AA provisioning has been, further, demonstrated in omnivorous/detritivorous cockroaches (Ayayee et al. 2016). AA provisioning in insects with separately evolved habits of wood‐feeding (Ayayee et al. 2014; Tokuda et al. 2014), phloem‐feeding (Sasaki et al. 1991; Febvay et al. 1999; Bernays and Klein 2002; Akman Gündüz and Douglas 2009; Chong and Moran 2018; Moriyama and Fukatsu 2022), and fruit‐feeding (Kashima et al. 2006; Ben‐Yosef et al. 2014) suggests a link between this service and low dietary nitrogen. While fruit is more nitrogen‐rich than the former two foods, it is possible that anti‐nutritional, defensive compounds make AAs less accessible than otherwise expected (Ben‐Yosef et al. 2015).

#### 4.3.9 Objective 2c—Experimental and Genomic Results for Nitrogen Recycling, Nitrogen Fixation, and Fibre Catabolism

For the remaining functions of nitrogen recycling, nitrogen fixation, and fibre catabolism, we found a rich body of experimental literature and genome‐encoded capacities (Data S1; Figure 5C; Figure S17). Dietary nitrogen did not predict the counts of genes encoding urea‐based nitrogen recycling. Nitrogen fixation was encoded by few of our studied obligate microbiomes, preventing genome versus nutrient analysis. Symbioses with in vivo evidence for nitrogen fixation were concentrated in wood and detritus feeders. These were found across 65 species that spanned eight origins of obligate symbiosis. Direct evidence for nitrogen recycling was rarer (14 insect species; 9 origins of obligate symbiosis) and found sporadically across diets.

In contrast to the above nitrogen‐focused genome results, genome‐encoded obligate symbiont capacities for lignin, pectin, hemicellulose, and cellulose catabolism were predicted by fibre content of host diets. Although intuitive, these findings require follow‐up research since there are many plant cell wall degrading enzymes without a KO or EC number designation. Our reliance on such KO‐ and EC‐defined enzymes, and their mapping onto a KEGG pathway step, provides only a partial picture.

Experimental support for in vivo fibre catabolism was found across 76 insect species representing 12 separate origins of obligate symbiosis. Comprising the breakdown of cellulose, hemicellulose, pectin, or lignin, this suite of functions was most common in wood‐ and detritus‐feeding insects—resembling the trend for nitrogen fixation. Other symbioses in these guilds may metabolize hard‐to‐digest sugars or starches, suggesting further ways in which they help to access recalcitrant carbon.

Overall, nitrogen‐fixation and fibre catabolism were concentrated in insects with extracellular symbioses. Since AA‐ and B‐vitamin provisioning were seen across most modes of integration (e.g., also in intracellular symbionts), these have a broader reach as niche‐accessing innovations. We focus the rest of our manuscript on these latter functions.

### 4.4 Symbiotic, Diversity‐Sustaining Key Innovations

#### 4.4.1 Symbiotic Key Innovations

Proliferation of vascular plants, including seed plants and angiosperms, created numerous niches and demand for novel insect adaptations (Labandeira 2013; Benton et al. 2022). Drastic changes to mouthparts were an important part of insect responses, facilitating specialized use of different tissues (Blanke et al. 2015; Kawahara et al. 2019; Labandeira 2019; Wang, Brożek, and Dai 2020). Beyond the demands for precise tissue ingestion, specialized requirements for nutrient acquisition were also integral to insect‐plant interactions (Felton and Gatehouse 1996; Chen et al. 2005; Behmer 2009).

Are many of the resulting adaptations properly defined as key innovations—that is, novel, revolutionary traits enabling niche exploitation? Or were changes more often due to tinkering with ancestral traits? Across the eukaryotic phylogeny, obligate symbioses may often fit the former model. Indeed, their evolution has brought about both radical changes to host morphology/physiology and major shifts into novel niches, including oxygen‐rich environments and terrestrial habitats (Margulis 1971; Gray 1993; Brundrett 2002; Wang and Qiu 2006). So our tested hypothesis has prior precedent.

From an insect's perspective, diverse microbial metabolism harnessed through obligate symbiosis can overcome plant nutritive limits. But while bacteria, protists, and fungi can be commonly symbiotic with insects, many symbioses are costly (Caragata et al. 2014; Allman et al. 2020; Engl et al. 2020; Ma et al. 2021; Zytynska et al. 2021; Clavé et al. 2022). Obligate symbioses may be all the moreso, as they are harnessed by major changes to host morphology, physiology, and behaviour (Login et al. 2011; Six 2012; Kaltenpoth et al. 2014; Vigneron et al. 2014; Lanan et al. 2016; Ankrah and Douglas 2018; Ohbayashi et al. 2020; Wilson 2020; Jang et al. 2021; Alarcón et al. 2022; Fukumori et al. 2022; Hu et al. 2023; Koga et al. 2022; Duncan et al. 2023; Luan 2024; Oguchi et al. 2024). Such costs and such complex integration may explain why obligate symbioses are the exception, not the rule. Their persistence implies stable net benefits in the face of unique or unusually strong environmental pressures (Six and Klepzig 2021).

Such symbioses could serve as key innovations, but systematic efforts relating obligate symbiont function to host nutritional ecology have been lacking. To be clear, there is little doubt that the metabolism of some obligate symbionts has been co‐opted to meet dietary challenges. But where have these challenges and the corresponding symbiont services been measured and linked? And is a large fraction of herbivorous insect diversity supported by symbionts' nutritive key innovations?

In our study we identified 627 families with primary reliance on a single food source at the juvenile stage, comprising 563,524 insect species. From data in the published literature, we showed that obligate symbionts are enriched in those specialized on a subset of these diets, including xylem sap, phloem sap, wood, seeds, and fruit. The low levels of nitrogen and/or certain B‐vitamins characterizing some of these tissues were predictive of both the presence and metabolism of obligate symbionts. Extreme levels of fibre in wood similarly predicted symbioses and their encoded cellulose, hemicellulose, lignin, and pectin metabolism. A good volume of experimental data connects this genome‐predicted metabolism with insect nutritional phenotypes.

Shifts to some plant tissue‐feeding niches have, thus, unfolded through evolution of intricate symbioses fulfilling dietary needs. Insects with shared plant tissue diets may consume disparate plant taxa and come from groups with somewhat variable needs for growth and reproduction (Dadd 1985; Fagan et al. 2002). In the face of this variation, functional symbiotic convergence and long‐standing retention of symbiont function are quite amazing (McCutcheon et al. 2009; Bittleston et al. 2016).

Due to extremely poor nutritive content and scarce examples of symbiont‐free exploitation, the phloem and xylem sap feeding niches may be inaccessible without symbionts. Need‐matching obligate symbiont services seem to also enable seed‐, wood‐, and fruit‐feeding. Obligate symbiont‐free use of these latter foods suggests that non‐essential symbionts, insect‐encoded solutions, or food‐enriching environmental microbes may sometimes be sufficient (Table S4). It is also possible that varying reliance on obligate symbiosis across these guilds stems from varying evolvability of symbiosis or cryptic nutritional differences due to sub‐tissue heterogeneity (e.g., bark vs. cambium vs. sapwood vs. heartwood; Merrill and Cowling 1966; Ito 1983). Variable feeding across plant decay/development (Stamp and Bowers 1990; Ulyshen 2016), or supplementary feeding on more nutritious foods (Molleman 2010) could also play a role (Box 5).

#### 4.4.2 Why Aren't Nutritional Symbiont Key Innovations More Widespread?

Our analyses back prior estimates that over half of the world's million‐plus insect species are herbivorous. With herbivores being more frequently engaged in obligate symbioses we asked whether symbiotic nutritive key innovations sustain the bulk of insect herbivore diversity by allowing continued use of poor diets. With < 47.8% of species from herbivorous families relying on a highly integrated, obligate symbiont (i.e., < 257,762 species), and with some symbionts' of herbivores supporting only diet‐decoupled cuticular needs (Anbutsu et al. 2017), the reach of these key innovations falls short of a majority.

Beyond the costs of symbioses and the complexity of integration, a likely driver of obligate symbiont scarcity extends from three patterns demonstrated here. First, more herbivores consume leaves than any other plant tissue. Second, leaves are relatively good sources of vitamins and amino acids. And third, while the pectin and lignocellulose content from cell walls complicates leaf digestion, many insects possess plant cell wall‐degrading genes within their genomes (Calderón‐Cortés et al. 2012; McKenna et al. 2019).

#### 4.4.3 Nutritional Key Innovations Supporting Highly Diverse Hemipteran Taxa

So where have well‐defined symbiont key innovations sustained large swaths of diversity (Figure 7A)? The strongest case with far‐reaching impacts presently comes from the Hemiptera. Primarily herbivorous hemipteran families with reliance on obligate symbionts comprise 68,148 species—a sizeable ~26% portion of the aforementioned 257,762 species figure. Following two origins of reliance on highly integrated, obligate symbiosis within hemipteran herbivorous lineages (Figure 7B), many descendants have retained their symbionts, despite an impressive exception within the ~6000 species‐strong, mesophyll‐feeding Typhlocybinae (Hemiptera: Auchenorrhyncha: Cicadellidae) (Dietrich 2013; Li et al. 2017; Johnson et al. 2018). Across plant‐feeding hemipterans, functions/metabolism of obligate microbiomes trend strongly toward B‐vitamin provisioning across diets of seeds and, to some extent, those of phloem and xylem sap. Tendencies toward AA provisioning are, similarly, evident in insects consuming these plant sap fluids (Bell‐Roberts et al. 2019; Sudakaran et al. 2017; Weirauch et al. 2019) and, possibly, fruit (Kashima et al. 2006; Sabree et al. 2013). It is notable that symbiont functions seem to match deficiencies in these tissues. Combining this with the 300 million years of symbiotic history within this group (Li et al. 2017), the mechanisms behind symbiont‐enabled plant feeding and the reach of these symbioses across time and diversity are both clear and staggering.

**FIGURE 7:**
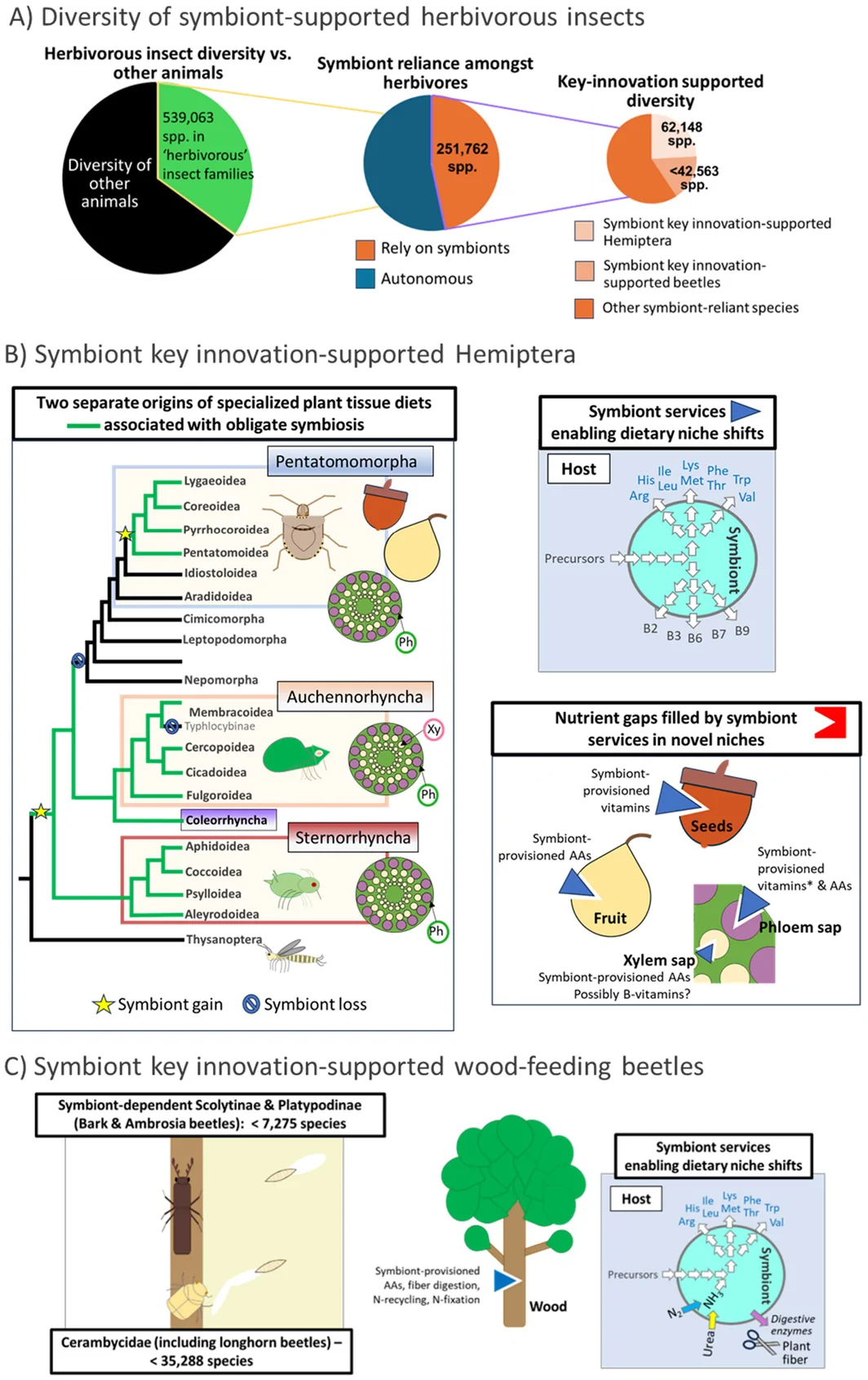
The reach of nutritional symbiotic key innovations in sustaining insect diversity. (A) Described insect herbivore diversity is substantial compared to that of other animals. Among this fraction, just under half of insect herbivores come from a family with an obligate symbiont. And from this subset, symbiont‐supported Hemiptera make up ~26% (24% if we account for the obligate symbiont‐free state among diverse Typhlocybinae leafhoppers). Note, the left‐most pie chart is not quantitative due to a rough approximation that herbivorous insects make up around one‐third of animal species. The middle and right‐most pie charts use numbers from the GBIF database when possible, with sums from the symbiont‐reliant herbivorous Hemiptera being corrected for the non‐reliant, but herbivorous Typhlocybinae (~6000 species). Some Cerambycidae and some bark beetles appear symbiont non‐reliant, hence the use of our ‘<’ symbol in those species counts. (B) Separate origins of obligate symbiosis in herbivorous Hemiptera are associated with specialized feeding on phloem sap, xylem sap, fruit, and seeds. Host‐level nutritional phenotypes (for all but xylem sap feeders) and symbiont genomics reveal definitive or likely AA and/or B‐vitamin provisioning matching nutrient scarcities in these diets. However, obligate microbiomes of some phloem‐feeding Sternorrhyncha have lost expected B‐vitamin synthesis pathways, raising questions in need of further study (denoted by asterisk). (C) Wood‐feeding by many Cerambycidae and bark/ambrosia beetles is associated with symbiosis. Experimental evidence from the Cerambycidae suggests that the challenges of a wood diet are met by symbionts' amino acid provisioning, nitrogen recycling/fixation, fibre digestion or other aspects of carbohydrate metabolism. B‐vitamin provisioning is plausible, but largely untested.

## 5 Conclusions

Key innovations are a central focus of evolutionary biology (Miller et al. 2023), supporting some of the world's most species‐rich taxa (Blaimer et al. 2023). In a few cases, outsourced services achieved by symbioses have been convincingly shown to comprise such key innovations. This is true for the ancient symbiosis that evolved into our mitochondria (Mills et al. 2022), and for the mycorrhizal symbioses allowing plant terrestrialization (Wang et al. 2010).

Here we formally test the symbiotic key innovation hypothesis for herbivorous insects, addressing whether obligate, nutritional symbioses opened plant feeding niches (Moran and Telang 1998). Two general expectations were met. First, insects evolved obligate symbioses across three convergent cases of phloem‐sap diets. Despite different types of symbionts and despite subsequent symbiont turnover within two of these host lineages, symbiont function has also converged. As such, symbiont‐mediated essential AA‐ and (sometimes) B‐vitamin provisioning services have come to match the deficiencies of phloem sap. Such need versus function matches may also exist for xylem‐feeders, although data are currently lacking. For the second set of a priori expectations, convergently evolved wood feeding is similarly linked to separately evolved obligate symbioses. These convergently express AA‐provisioning, nitrogen recycling, nitrogen fixation, and fibre catabolic services. Given the scarcity of nitrogen, amino acids, and accessible carbon in woody tissues, symbiont function seems to often match host need. B‐vitamin provisioning may also be important in this niche, but data on these nutrients are mostly lacking for woody tissue.

In two newly reported discoveries, we see parallel matches between symbiont services and plausible dietary shortcomings in insects consuming fruit and seeds. The fruit feeding guild has not previously been implicated as symbiosis‐enriched. A combination of modest nitrogen content (shown here) and reduced AA availability in some pre‐ripened fruits (Ben‐Yosef et al. 2015) could explain this trend. Studies on *Drosophila suzukii* appear illustrative. These flies eat ripe or ripening fruit, not the microbe‐rich slurries of post‐ripened or rotting fruit consumed by other *Drosophila*. Work from this system has revealed protein starvation in symbiont‐free *D. suzukii* consuming natural diets and a reliance on non‐specific gut microbes to rectify this (Bing et al. 2018). While this is not a highly integrated, obligate symbiosis, these findings fit with patterns of essential AA biosynthesis and provisioning by convergently evolved obligate symbioses of fruit feeders. Symbiont‐enabled fruit‐feeding seems evident in armoured scale insects, varying tephritid flies, and many Pentatomomorpha (Table S5), suggesting useful systems for further study. Studies on convergently evolved symbioses will be further insightful for seed feeders, given common B‐vitamin provisioning in the face of B‐vitamin scarcity within seed tissue. Such efforts may focus on a range of species across the Pentatomomorpha with distinct sets of obligate symbionts (Table S5). They will be further useful in at least three beetle taxa, where seed feeding and symbiosis evolved independently.

Symbiont‐encoded, nutritional key innovations have thus been crucial for insects within four tissue‐specific feeding guilds. But many—if not most—insect herbivores exist without nutritive, obligate symbioses. The prevalence of leaf tissue diets helps to explain this, as leaves are reasonably good sources of AAs and most B‐vitamins. Laterally transferred, plant cell wall degrading genes help to overcome the fibre‐richness of leaves, limiting the need for obligate symbiosis (Shelomi et al. 2020; Kirsch et al. 2025). Given the costs and complexity of obligate symbioses, and the moderate richness of some plant‐based diets, it is not surprising to see so many herbivorous taxa without them. Indeed, endogenous solutions and those supported through lateral gene transfer may be less costly or easier to evolve. Such solutions may not be accessible in solving B‐vitamin and essential AA shortages. We discuss this topic and exceptions to the above ‘rules’ in Box 5, addressing the context‐dependence of pressures to evolve symbiosis.

Returning to our focus on diversity, we note that prior studies have asked the questions of whether symbionts promote diversification (Janson et al. 2008) and whether symbiont‐reliant organisms diversify faster than relatives (Richardson 2001; Joy and Crespi 2007; LaJeunesse et al. 2018; Dorchin et al. 2019; Cornwallis et al. 2023). Our paper considers a different aspect of insect diversity. We show, simply, just how and why 10s of thousands of insects would not exist without symbiosis. Symbiotic key innovations support much diversity within the Hemiptera. A case can also be made that symbioses in some wood‐feeding Coleoptera comprise nutritive key innovations (Data S1—discussion section; Figure 7C). Given the hyper‐diversity of symbiont‐reliant bark and ambrosia beetles, and of the Cerambycidae, it seems that obligate symbionts have joined the list of those displaying ‘an inordinate fondness for beetles’ (Farrell 1998).

## Author Contributions

The study, design, and scope of the manuscript were crafted by Patrick Abbot and Jacob A. Russell. The design and implementation of the symbiont genome versus nutrient analyses section was overseen by Hannah M. Smith, with assistance from Allison Hansen and Jacob A. Russell. Jacob A. Russell and Patrick Abbot wrote the manuscript, with assistance on the genomics sections from Hannah M. Smith. Benoit Bechade, Christian Cabuslay, Allison Hansen, Clesson Higashi, Yi Hu, Hannah M. Smith, and Abigail Williams helped with editing the manuscript. Research on insect diet was performed by Patrick Abbot, Daniel Freeman, Jacob A. Russell, and Abigail Williams. Research on plant tissue nutrition was performed by Benoit Bechade, Christian Cabuslay, Melissa Carpenter, Clesson Higashi, Yi Hu, Linyao Peng, and Jacob A. Russell. Research on the occurrence of obligate symbiosis was performed by Patrick Abbot and Jacob A. Russell, who also performed all research on in vivo studies of symbiont function. Statistical analyses were performed by Yi Hu, Jacob A. Russell, Hannah M. Smith, and Abigail Williams. Patrick Abbot, Yi Hu, Jacob A. Russell, and Hannah M. Smith designed figures and tables.

## Funding

This study was supported by the National Science Foundation (2240393, 1442144).

## Ethics Statement

This article is an Invited Review and does not report new experiments involving humans, animals, field sampling, or the collection of biological material. Therefore, ethical approval was not required.

## Conflicts of Interest

The authors declare no conflicts of interest.

## Supporting information


**Data S1:** Methods, Results, and Discussion.

**Data S2:** Description of Zenodo materials for Russell et al.

**Figure S1:** Diversity of juvenile‐stage food consumption across each insect family.
**Figure S2:** Juvenile feeding habit prevalence, summarized at the order level.
**Figure S3:** Feeding habit prevalence, summarized across the insects.
**Figure S4:** Diversity of foods consumed across herbivorous insect families.
**Figure S5:** Insect phylogeny with mapped feeding habits, diversity, and symbiosis (see separate file).
**Figure S6:** Box plots and outcomes of statistical comparisons in nitrogen, fibre, and B‐vitamin content among plant tissues. See separate file.
**Figure S7:** B‐vitamin quantities in insect diets predict obligate symbiosis.
**Figure S8:** Nitrogen quantities in insect diets predict obligate symbiosis; fibre quantities do not.
**Figure S9:** Metabolic pathways used in assessments of symbiont nutrient biosynthesis/pathway completeness and unique KO counts.—See separate file.
**Figure S10:** Genetic divergence of symbionts versus free‐living bacteria predicts genome size.
**Figure S11:** Dietary B‐vitamin content predicts symbiont capacities for synthesizing pantothenate (B5) and biotin (B7).
**Figure S12:** In insects with diets below the required B‐vitamin thresholds, symbionts encode more complete riboflavin (B2), pantothenate (B5), and pyridoxine (B6) pathways.
**Figure S13:** Separate origins of nutrient‐poor diets are associated with separate origins of symbioses addressing nutrient needs.
**Figure S14:** Facultative symbionts of some symbiont‐reliant insects expand the portions of encoded B‐vitamin synthesis pathways in phloem‐feeding insects and beyond.
**Figure S15:** Dietary nitrogen levels predict dietary amino acid levels.
**Figure S16:** Dietary nitrogen content predicts symbiont capacities for synthesizing all essential amino acids except lysine.
**Figure S17:** Dietary nutrient content predicts symbiont fibre—but not urea ‐metabolism.
**Figure S18:** Cuticle‐enriched tyrosine content is highly variable across insect food sources.

**Table S1:** (Zenodo—Table S2): Ten most speciose herbivorous and non‐herbivorous insect families. Rankings are based on data summaries from Table S1. Included here, also, are data on obligate symbioses obtained from Table S3.
**Table S2:** (Zenodo—Table S4): Modes of symbiont integration and host‐level investment. Aspects of symbiont housing, specialization, and transmission/acquisition are discussed. Those defined as obligate symbioses have pink highlighting in column B. Examples are provided for each mode of integration, including known symbiont function in such instances, and typical diet of these symbiont‐housing hosts.
**Table S3:** (Zenodo—Table S5): Separate origins of obligate symbiosis across the insect phylogeny. Parsimonious assessments of symbiont origins were made using the phylogeny from Rainford et al. 2014 (doi:10.1371/journal.pone.0109085) and obligate symbiont distributions in Table S4. In some cases, literature on symbiont integration helped to distinguish single versus multiple origins among closely related taxa. See column G for further discussion of/justification for our approach. The estimate of 44 origins is likely lower than the actual number due to the plausibility of unidentified obligate symbioses in some taxa, and polyphyletic origins of some obligacy among fairly close relatives (e.g., ambrosia beetle/fungus farming within the Curculionidae—DOI: 10.1111/mec.14394).
**Table S4:** (Zenodo—Table S6): Surprising exceptions to symbiont presence/absence in several insect families. Shown here are: (1) plausibly obligate symbiont‐free (or understudied) insect taxa with diets normally associated with obligate symbiosis; and (2) obligate symbiont‐harbouring insect taxa with diets rarely associated with obligate symbiosis. Additional notes are provided, based on our literature review.
**Table S5:** (Zenodo—Table S7): Symbiosis across the Pentatomomorpha (Hemiptera: Heteroptera). Diet and symbioses have evolved within the heteropteran infraorder, Pentatomomorpha. We provide information on these two attributes across separate families from this taxon, highlighting dietary correlations and changes in symbiosis across this group's history. Notable here is the common presence of obligate symbionts within seed‐feeding families. *Caballeronia* symbioses are highlighted here in blue. These bacteria are acquired from the environment (e.g., the soil) and were formerly referred to as *Burkholderia* and *Paraburkholderia* before taxonomic revision. Housing mechanisms for *Cabelleronia* symbionts—and some others, as well—typically involve crypts in the M4 midgut region. We highlight these in green. This symbiotic syndrome is widespread across this insect infraorder. A question arises due to the variable symbioses across this group—ranging from maternally inherited to environmentally acquired—what are the pros and cons of obtaining larger‐genomed, free‐living symbionts each generation from the environment?
**Table S6:** (Zenodo—Table S21): Comparing nutrition across plant tissues versus non‐vascular plant foods. Foods were ranked by their averages or mid‐range values. Values encompassing the average insect requirement for each of the seven studied B‐vitamins are shaded in grey.
**Table S7:** (Zenodo—Table S27): Dietary B‐vitamin requirements for nine insect species spanning five orders. Data computed from Chapter 8 of Dadd, R. H. 1985. Nutrition: organisms, pp. 313–390. In Kerkut G. A. and Gilbert L. I. (eds.), Comprehensive insect physiology, biochemistry, and pharmacology, vol. 4. Chapter 8. Pergamon Press Inc., New York.
**Table S8:** (Zenodo—Table S29): Metadata on the (meta)genomes used in our analysis of obligate symbiont genome metabolism. Included here are accession numbers, database sources, host species and symbiont names, and information on each symbiosis. We also indicated which symbiont origin number (out of the 44 estimated) each symbiont was derived from (column F), using information from Table S3. For some origin number identifiers we added subdividing letters, upper‐case roman numerals, and/or lower case roman numerals to differentiate symbioses showing partner exchange after a single presumed ancestral origin. For instance, within the weevil lineage evolving symbioses with bacteriocyte‐dwelling/cuticle‐building bacteria (origin number 33), those hosts harbouring *Sodalis* were given the designation of 33.a, while those harbouring *Curculioniphilus* or the ancestral *Nardonella* were designated as 33.b and 33.c. In a separate ID assignment, we used a replicate numbering system to designate nucleic acid accessions belonging to the obligate microbiome of a single host species (column G). To illustrate this, consider the insect host *Cinara cedri*. This aphid possesses two obligate symbionts— *Buchnera aphidicola* and *Serratia symbiotica* . *Buchnera* genome data were found under two NCBI accession numbers, while the *Serratia* genome data were deposited under one IMG genome ID. Because these three accessions comprise the entirety of one obligate microbiome, they were given the same analysis replicate ID (i.e., 18). Facultative symbionts included in pathway analyses were not given such IDs, nor were some microbes with unclear status as obligate symbionts.
**Table S9:** (Zenodo—Table S74): Linear regression statistics on symbiont B‐vitamin completeness versus log‐transformed dietary B‐vitamin data. Statistics were run on the GraphPad website (https://www.graphpad.com/quickcalcs/linear1/) on log‐transformed dietary B‐vitamin data (x‐variable) and untransformed completeness estimates (y‐variable). Slopes of best‐fit regression lines significantly different from zero were given blue highlighting if the slope was negative (as expected), and yellow highlighting if the slope was positive.
**Table S10:** (Zenodo—Table S75): *T*‐tests on symbiont B‐vitamin completeness versus dietary B‐vitamin levels above or below average insect requirements. One‐tailed *t*‐tests (with unequal variance) were run in Microsoft Excel. Dietary B‐vitamin requirement thresholds were the averages from Table S7. Data analyses were only run after excluding all metagenome data and data from hosts where obligate symbionts had > 95% 16S rRNA gene sequence identity to free‐living bacteria, on average. *p*‐values from *t*‐tests are in row 36. Significant values were colour‐coded, with those in blue matching the expected direction (i.e., higher symbiont B‐vitamin pathway completeness for insects with diets falling below insect B‐vitamin requirements).
**Table S11:** (Zenodo—Table S79): Linear regression stats for non‐vitamin symbiont functions—AA synthesis, N‐recycling, N‐fixation, fibre digestion. Statistics were run on the GraphPad website (https://www.graphpad.com/quickcalcs/linear1/) on and pathway completeness/KO count estimates (y‐variables) versus select dietary nutrient data (x‐variables). Slopes of best‐fit regression lines significantly different from zero were given blue highlighting if the slope was negative (as expected for amino acid completeness versus % nitrogen data). For significant slope results in our KO count data (all but AA synthesis), where we expected positive correlations in analyses with % fibre as the x‐variable, highlighting for positive slopes was in yellow. Note that due to nearly all nitrogen fixation proteins being detected in metagenomes (outside of one instance), we did not run statistics on that function versus dietary nutrient relationship.
**Table S12:** (Zenodo—Table S81): Symbiont amino acid pathway completeness versus time since domestication for insects on blood, phloem, and seed diets. For all analyses we used linear regression; y‐variable = pathway completeness for AA in question; x‐variable = 16S rRNA identity to most closely related free‐living bacterium (a proxy for time since domestication). Significant and borderline‐significant *p*‐values are given yellow and beige highlighting. For each tissue‐specific analysis, we included all relevant obligate microbiomes (young and ancient) except for those sampled through metagenomics. For multi‐species microbiomes we used the average 16S rRNA identity across co‐obligate symbionts.
**Table S13:** (Zenodo—Table S82): Encoded steps of B‐vitamin and AA biosynthetic pathways across obligate microbiomes of blood‐, seed‐, and phloem‐feeders. Pathway steps with varying numbers of ‘*’ in column Z are those that overlap across multiple pathways (e.g., the same enzymes used for steps 2, 3, 4, and 5 of the Ile pathway are used to fulfil steps 1, 2, 3, and 4 of the Leu pathway). This helps to show partial non‐independence of some pathway completeness calculations.
**Table S14:** (Zenodo—Table S86): Linear regression examining the relationship between % amino acid content versus % nitrogen of varying insect foods. Excluded blood & root_storage tissue because of *n* = 1 for amino acids and nitrogen, respectively. Inputs for % amino acid data are in Table S84. Those for % nitrogen are in column B.

## Data Availability

Raw and analysed data (plus details on sources) from our literature review on insect food consumption, insect diversity, obligate symbioses, plant nutrition, symbiont function, and symbiont genomic capacity are presented in Tables within the Supporting Information section and in those uploaded to the Zenodo data repository at: https://doi.org/10.5281/zenodo.19607748. We describe protocols and results of several statistical analyses in the Data S2 document.
