# Supplementary material for: Symbiont‐Derived Key Innovations Across the Diversity and Diets of Herbivorous Insects
Source: Mol Ecol. 2026 Nov 7;35(21):e70615. doi: 10.1111/mec.70615 (PMC13743980; doi:10.1111/mec.70615)
Supplement: Supplementary file 1 [file MEC-35-e70615-s001.docx]

**Contents of Russell et al. Zenodo data repository submission**

https://doi.org/10.5281/zenodo.19607748

**Zenodo SUPPLEMENTARY FIGURES**

Same as Molecular Ecology supplementary materials.

**Zenodo SUPPORTING INFORMATION**

Document on methods, including statistical results and some discussion – same as Molecular Ecology supplementary materials.

**Zenodo SUPPLEMENTARY TABLES**

87 Tables grouped into 11 Microsoft Excel files. Numbering is distinct from that of the smaller # of supplementary materials contained within the supplementary materials on the Molecular Ecology website.

**Zenodo SUPPLEMENTARY TABLE LEGENDS**

**Table S1: Diets and species richness across insect families and other hexapod taxa.** Numerical scoring system (1-3) indicates dietary predominance across taxa, based on a literature review (see sources in column AY). “Predominant larval herbivory” column (BA) assesses whether the sum of scores across 14 herbivorous categories exceeds the sum from the 6 remaining foods. Diet is classified separately for insects at juvenile/larval vs. adult stages, and asterisks next to numerical scores indicate the tentative nature of some classifications. We indicate species counts for all families, when available, in column BB based on searches of the GBIF database (https://www.gbif.org). Findings on diet from three other studies are summarized here, as well (Labandeira 2014 - DOI: 10.1007/978-3-319-07623-2_13; Rainford & Mayhew 2015 - DOI: 10.1086/683461; Cornwallis et al. 2023 – DOI: 10.1038/s41559-023-02058-0).

**Table S2: Ten most speciose herbivorous and non-herbivorous insect families.** Rankings are based on data summaries from Table S1. Included here, also, are data on obligate symbioses obtained from Table S3.

**Table S3: Insect families and their known associations with obligate symbioses (or lack thereof).** We include notes and citations from our literature reviews for taxa with varying degrees of evidence for obligate symbiosis. Provided, also, are species richness estimates from column BB of Table S1.

**Table S4: Modes of symbiont integration and host-level investment.** Aspects of symbiont housing, specialization, and transmission/acquisition are discussed. Those defined as obligate symbioses have pink highlighting in column B. Examples are provided for each mode of integration, including known symbiont function in such instances, and typical diet of these symbiont-housing hosts.

**Table S5: Separate origins of obligate symbiosis across the insect phylogeny.** Parsimonious assessments of symbiont origins were made using the phylogeny from Rainford et al. 2014 (doi: 10.1371/journal.pone.0109085) and obligate symbiont distributions in Table S4. In some cases, literature on symbiont integration helped to distinguish single vs. multiple origins amongst closely related taxa. See column G for further discussion of/justification for our approach. The estimate of 44 origins is likely lower than the actual number due to the plausibility of unidentified obligate symbioses in some taxa, and polyphyletic origins of some obligacy among fairly close relatives [e.g. ambrosia beetle/fungus farming within the Curculionidae – Vanderpool et al. 2018 (DOI: 10.1111/mec.14394)].

**Table S6: Exceptions to symbiont presence/absence in several insect families.** Shown here are: 1) plausibly obligate symbiont-free (or understudied) insect taxa with diets normally associated with obligate symbiosis; and 2) obligate symbiont-harboring insect taxa with diets rarely associated with obligate symbiosis. Additional notes are provided, based on our literature review.

**Table S7: Symbiosis across the Pentatomomorpha (Hemiptera: Heteroptera).** Diet and symbioses have evolved within the heteropteran infraorder, Pentatomomorpha. We provide information on these two attributes across separate families from this taxon, highlighting dietary correlations and changes in symbiosis across this group’s history. Notable here is the common presence of obligate symbionts within seed-feeding families. *Caballeronia* symbioses are highlighted here in blue. These bacteria are acquired from the environment (e.g. the soil) and were formerly referred to as *Burkholderia* and *Paraburkholderia* before taxonomic revision. Housing mechanisms for *Cabelleronia* symbionts – and some others, as well – typically involve crypts in the M4 midgut region. We highlight these in green. This symbiotic syndrome is widespread across this insect infraorder. A question arises due to the variable symbioses across this group – ranging from maternally inherited to environmentally acquired - what are the pros and cons of obtaining larger-genomed, free-living symbionts each generation from the environment?

**Table S8: Percent nitrogen as a function of dry weight across varying plant tissues.** Data were obtained from peer-review research and review literature and from online databases. Taxonomic information is provided for each plant species. Due to replication within some species, average % nitrogen levels were computed at the plant species level, for each tissue (see columns N-R).

**Table S9: Percent neutral detergent fiber (cellulose, hemicellulose, lignin) as a function of dry weight across varying plant tissues.** Data were obtained from peer-review research and review literature and from online databases. Taxonomic information is provided for each plant species. Due to replication within species, average % fiber levels were computed at the plant species level, for each tissue (see columns N-R).

**Table S10: Percent thiamine (vitamin B1) as a function of dry weight across varying plant tissues.** Data were obtained from peer-review research and review literature and from online databases, with most data coming from USDA’s food data central database (https://fdc.nal.usda.gov). Taxonomic information is provided for each plant species. Due to replication within species, average % thiamine levels were computed at the plant species level, for each tissue (see columns N-R).

**Table S11: Percent riboflavin (vitamin B2) as a function of dry weight across varying plant tissues.** Data were obtained from peer-review research and review literature and from online databases, with most data coming from USDA’s food data central database (https://fdc.nal.usda.gov). Taxonomic information is provided for each plant species. Due to replication within species, average % thiamine levels were computed at the plant species level, for each tissue (see columns N-R).

**Table S12: Percent niacin/nicotinate (vitamin B3) as a function of dry weight across varying plant tissues.** Data were obtained from peer-review research and review literature and from online databases, with most data coming from USDA’s food data central database (https://fdc.nal.usda.gov). Taxonomic information is provided for each plant species. Due to replication within species, average % thiamine levels were computed at the plant species level, for each tissue (see columns N-R).

**Table S13: Percent pantothenate (vitamin B5) as a function of dry weight across varying plant tissues.** Data were obtained from peer-review research and review literature and from online databases, with most data coming from USDA’s food data central database (https://fdc.nal.usda.gov). Taxonomic information is provided for each plant species. Due to replication within species, average % thiamine levels were computed at the plant species level, for each tissue (see columns N-R).

**Table S14: Percent pyridoxine (vitamin B6) as a function of dry weight across varying plant tissues.** Data were obtained from peer-review research and review literature and from online databases, with most data coming from USDA’s food data central database (https://fdc.nal.usda.gov). Taxonomic information is provided for each plant species. Due to replication within species, average % thiamine levels were computed at the plant species level, for each tissue (see columns N-R).

**Table S15: Percent biotin (vitamin B7) as a function of dry weight across varying plant tissues.** Data were obtained from peer-review research and review literature and from online databases, with most data coming from USDA’s food data central database (https://fdc.nal.usda.gov). Taxonomic information is provided for each plant species. Due to replication within species, average % thiamine levels were computed at the plant species level, for each tissue (see columns N-R).

**Table S16: Percent folate (vitamin B9) as a function of dry weight across varying plant tissues.** Data were obtained from peer-review research and review literature and from online databases, with most data coming from USDA’s food data central database (https://fdc.nal.usda.gov). Taxonomic information is provided for each plant species. Due to replication within species, average % thiamine levels were computed at the plant species level, for each tissue (see columns N-R).

**Table S17: Percent B-vitamin content as a function of dry weight across varying non-vascular plant foods.** Data were obtained from the published research and review literature and from online databases. Genus and species are provided for each food. Average % B-vitamin levels were computed at the for each tissue and species (see columns R-Z). Included amongst these alternative foods were data from archaeal, eubacterial, protist, and fungal cells (yeast and others) – classified jointly as “microbes”; vertebrate blood; vertebrate muscle, insect, crustacean, and mollusk tissue – collectively representing carnivorous or animal tissue diets (“predator/scavenger/parasite”); and, finally, macroalgal and microalgal cells – classified as “algae”.

**Table S18: Percent nitrogen as a function of dry weight across varying non-vascular plant foods.** Data were obtained from the published research literature and are presented as ranges when multiple datapoints and/or sources were obtained. In addition to several of the foods referenced in the legend for Table S18, we also obtained %nitrogen data for slide molds (classified as microbes), soil, varying forms of detritus, lichen, mosses and liverworts, phloem-feeding insect honeydew (i.e. liquid excrement eaten by some ants), and diatom algae.

**Table S19: Summary stats for nutrients across non-vascular plant foods.** Data from Tables S17 were used to calculate averages, sample sizes, standard deviations, variances, and min/max ranges.

**Table S20: Summary stats for nutrients across plant tissue foods.** Data from Tables S8-S16 were used to calculate averages, sample sizes, standard deviations, variances, and min/max ranges.

**Table S21: Comparing nutrition across plant tissues vs. non-vascular plant foods.** Foods were ranked by their averages or mid-range values. Values encompassing the average insect requirement for each of the seven studied B-vitamins are shaded in gray.

**Table S22: Raw and utilized data for vitamins B6 and B7 in wood.** B-vitamin data were recorded across varying growth rings and stem/root wood or bark tissues. Table shows source/citation and raw data, while outlining the logic behind datapoints selected for analysis. In short, we chose the highest B-vitamin values across wood or bark, after combining the totals for free and bound B-vitamin estimates.

**Table S23: Calculations used to obtain percent dry weight content for nitrogen based on moles/liter data obtained for xylem sap.** Xylem sap is mostly water, with ~3% solute concentration. Using this value, an approximated density of 1 mg/ml (value for water), % nitrogen concentrations in molarity, and the molecular masses for the most abundant nitrogen sources in xylem sap (Arginine and GABA, assuming equal quantities of each), we yielded a rough estimate of % nitrogen in dry weight. Table shows logic and calculations in this conversion process, with citations to support assumptions/approximations along with solute concentrations and % nitrogen values (moles/liter).

**Table S24: Calculations used to obtain percent dry weight content for nitrogen based on fresh weight data obtained for nectar.** Density estimates of nectar were obtained from three separate studies, then averaged. Using this density, the average solute concentration for the nectar of insect-pollinated plants, and % nitrogen as a function of fresh weight, we yielded a rough estimate of % nitrogen in dry weight. Table shows logic and calculations in this conversion process, with citations to support assumptions/approximations, and our sources of data on density/solute concentration/fresh weight nitrogen values.

**Table S25: Calculations used to obtain percent dry weight content for nitrogen and B-vitamins based on fresh weight data obtained for phloem sap.** Sources and values for typical phloem sap solute concentration and density are provided here. Using these values, and fresh weight measures of nitrogen and several B-vitamins (all but folate), we obtained rough estimates of % dry weight content. Table shows all necessary calculations.

**Table S26: Calculations used to obtain percent dry weight content for nitrogen and B-vitamins based on fresh weight data obtained for vertebrate blood.** Sources and values for typical blood solute concentration and density are provided here. Using these values, and fresh weight measures of nitrogen and several B-vitamins, we obtained rough estimates of % dry weight content. Table shows all necessary calculations.

**Table S27: Dietary B-vitamin requirements for nine insect species spanning five orders.** add Raw data , with averages and ranges, based on Chapter 8 of Dadd, R. H. 1985. Nutrition: organisms, pp. 313–390. InKerkut G. A. and Gilbert L. I. (eds.), Comprehensive insect physiology, biochemistry, and pharmacology, vol. 4. Chapter 8. Pergamon Press Inc, New York.

**Table S28: Average, minimum, and maximum dietary B-vitamin requirements in percent dry weight.** Used data from Table S27, and converted to % dry weight.

**Table S29: Metadata on the (meta)genomes used in our analysis of obligate symbiont genome metabolism.** Included here are accession numbers, database sources, host species and symbiont names, and information on each symbiosis. We also indicated which symbiont origin number (out of the 44 estimated) each symbiont was derived from (column F), using information from Table S5. For some origin number identifiers we added subdividing letters, upper-case roman numerals, and/or lower case roman numerals to differentiate symbioses showing partner exchange after a single presumed ancestral origin. For instance, within the weevil lineage evolving symbioses with bacteriocyte-dwelling/cuticle-building bacteria (origin number 33), those hosts harboring *Sodalis* were given the designation of 33.a, while those harboring *Curculioniphilus* or the ancestral *Nardonella* were designated as 33.b and 33.c. In a separate ID assignment, we used a replicate numbering system to designate nucleic acid accessions belonging to the obligate microbiome of a single host species (column G). To illustrate this, consider the insect host *Cinara cedri*. This aphid possesses two obligate symbionts – *Buchnera aphidicola* and *Serratia symbiotica*. *Buchnera* genome data were found under two NCBI accession numbers, while the *Serratia* genome data were deposited under one IMG genome ID. Because these three accessions comprise the entirety of one obligate microbiome, they were given the same analysis replicate ID (i.e. 18). Facultative symbionts included in pathway analyses were not given such IDs, nor were some microbes with unclear status as obligate symbionts.

**Table S30 - % 16S rRNA gene sequence identity vs. the closest free-living relative of each studied obligate symbiont.** We used one NCBI-derived 16S rRNA gene sequence as a representative for each obligate symbiont in our genome study. Each was used as a query in a BLASTn search against the nr/nt database to identify the % sequence similarity vs. the closest free-living bacterium. Ignored here are the symbionts comprising complex communities typically sampled through metagenomics.

**Table S31 - Genome sizes (and % 16S rRNA identity vs. free-living bacteria) for the obligate symbionts in this study.** Genome sizes represent the numbers of summed base pairs from all plasmids, chromosomes, or scaffolds belonging to a single obligate symbiont isolate. Ignored here are metagenomes.

**Table S32 - Web and literature resources used to define the proteins/genes involved in pertinent symbiont metabolism.** For 25 types of distinct metabolism, we indicate the relevant literature and database (KEGG and MetaCyc) resources used in defining relevant enzymes/enzyme encoding genes.

**Table S33: BLAST-KOALA annotation of all FastA formatted proteins encoded by NCBI-derived symbiont genomes.** KEGG Orthology IDs (KOs) are indicated in column F, while pseudogene status – identified through text searches of NCBI protein annotations - is indicated in column A. Names of symbionts and host insects, along with accession numbers, can be linked with those in Table S29.

**Table S34: IMG (meta)genome annotations of encoded protein, as organized by the Abundance Profile tool.** KEGG Orthology IDs (KOs) are indicated in column D. Names of symbionts and host insects, along with accession/genome ID numbers, can be linked with those in Table S29.

**Table S35: EC number annotations of encoded proteins from *Stammera capleta* NCBI-derived genome (accession #: CP024013.1).** No FastA formatted protein files were available for this genome, preventing BLAST-KOALA annotation and generation of KO numbers. We, thus, used EC numbers for the encoded proteins to find associated KOs, searching for those linked to any on our list of 400 (i.e. those executing some portion of our focal symbiont metabolism).

**Table S36: Numbers of unique nitrogen-fixation proteins (KOs) encoded by symbiont genomes.** Searches against the data in Tables S33-S35 recovered proteins involved in nitrogen fixation. Metadata on genomes are provided in rows 3-6 across Columns K-ES. Presence/absence data for each protein/KO are found in these same columns across rows 7-15, while the sum of unique proteins (KOs) identified in each genome is indicated in row 17. An additional noteworthy point includes columns combining KO data across separate obligate symbionts from the same insect host. Columns with these full obligate microbiome calculations are labeled with the term “oblholobiont” row 4. All further statistical analyses were performed on datasets with a numerical value in row 5, which provide analysis replicate IDs. Abbreviations in row 4 are as follows: oblsym (the metabolism of a single obligate symbiont), oblholobiont (typically the combined metabolism across separate obligate symbionts in the same host, although *Cryptocercus* cockroach data given this same label comprised data pooled across a gut metagenome and an obligate symbiont), mbiome (the combined metabolism across a complex microbiome as sampled through metagenomics), facsym (metabolism from a non-obligate, facultative symbiont), and facholobiont (the combined metabolism of an obligate symbionts and facultative symbionts from a single host).

**Table S37: Numbers of unique nitrogen-recycling proteins (KOs) encoded by symbiont genomes.** For this analysis we focused on catabolism of urea, a process not encoded by insects (unlike uric acid catabolism). The generation of ammonia from urea creates molecules that insects can assimilate. Searches against the data in Tables S33-S35 recovered proteins involved in this process. Metadata on genomes are provided in rows 3-6 across Columns K-ES. Presence/absence data for each protein/KO are found in these same columns across rows 7-10. The sum of unique proteins (KOs) identified in each genome is indicated in row 12. An additional noteworthy point in this table includes columns combining KO data across separate obligate symbionts from the same insect host. Columns with these full obligate microbiome calculations are labeled with the term “oblholobiont” row 4. All further statistical analyses were performed on datasets with a numerical value in row 5, which provide analysis replicate IDs. Abbreviations in row 4 are as follows: oblsym (the metabolism of a single obligate symbiont), oblholobiont (typically the combined metabolism across separate obligate symbionts in the same host, although *Cryptocercus* cockroach data given this same label comprised data pooled across a gut metagenome and an obligate symbiont), mbiome (the combined metabolism across a complex microbiome as sampled through metagenomics), facsym (metabolism from a non-obligate, facultative symbiont), and facholobiont (the combined metabolism of an obligate symbionts and facultative symbionts from a single host).

**Table S38: Numbers of unique cellulose-catabolizing proteins (KOs) encoded by symbiont genomes.** Searches against the data in Tables S33-S35 recovered proteins involved in cellulose catabolism. Metadata on genomes are provided in rows 3-6 across Columns K-ES. Data for each protein/KO are found in these same columns across rows 7-14. Because of the way we extracted IMG data, ‘counts’ for (meta)genomes from IMG are capped at 1 per KO (despite higher gene copy numbers, in some instances). But NCBI KO counts occasionally exceeded 1. We thus coded KO presence/absence in rows 17-24, while the sum of unique cellulose-catabolizing proteins (KOs) identified in each genome is indicated in row 26. One additional point of interest is our combination of KO data across separate obligate symbionts from the same insect host. Columns with these full obligate microbiome calculations are labeled with the term “oblholobiont” row 4. All further statistical analyses were performed on datasets with a numerical value in row 5, which provide analysis replicate IDs. Abbreviations in row 4 are as follows: oblsym (the metabolism of a single obligate symbiont), oblholobiont (typically the combined metabolism across separate obligate symbionts in the same host, although *Cryptocercus* cockroach data given this same label comprised data pooled across a gut metagenome and an obligate symbiont), mbiome (the combined metabolism across a complex microbiome as sampled through metagenomics), facsym (metabolism from a non-obligate, facultative symbiont), and facholobiont (the combined metabolism of an obligate symbionts and facultative symbionts from a single host).

**Table S39: Numbers of unique hemicellulose-catabolizing proteins (KOs) encoded by symbiont genomes.** Searches against the data in Tables S33-S35 recovered proteins involved in hemicellulose catabolism. Metadata on genomes are provided in rows 3-6 across Columns K-ES. Presence/absence data for each protein/KO are found in these same columns across rows 7-18. Total number of unique proteins (KOs) identified in each genome is indicated in row 34. One additional point of interest is our combination of KO data across separate obligate symbionts from the same insect host. Columns with these full obligate microbiome calculations are labeled with the term “oblholobiont” row 4. All further statistical analyses were performed on datasets with a numerical value in row 5, which provide analysis replicate IDs. Abbreviations in row 4 are as follows: oblsym (the metabolism of a single obligate symbiont), oblholobiont (typically the combined metabolism across separate obligate symbionts in the same host, although *Cryptocercus* cockroach data given this same label comprised data pooled across a gut metagenome and an obligate symbiont), mbiome (the combined metabolism across a complex microbiome as sampled through metagenomics), facsym (metabolism from a non-obligate, facultative symbiont), and facholobiont (the combined metabolism of an obligate symbionts and facultative symbionts from a single host).

**Table S40: Numbers of unique lignin-catabolizing proteins (KOs) encoded by symbiont genomes.** Searches against the data in Tables S33-S35 recovered proteins involved in lignin catabolism. Metadata on genomes are provided in rows 3-6 across Columns K-ES. Data for each protein/KO are found in these same columns across rows 7-18. Because of the way we extracted IMG data, ‘counts’ for (meta)genomes from IMG are capped at 1 per KO (despite higher gene copy numbers, in some instances). But NCBI KO counts occasionally exceeded 1. We thus coded KO presence/absence rows 17-24, while the sum of unique proteins (KOs) identified in each genome is indicated in row 26. One additional point of interest is our combination of KO data across separate obligate symbionts from the same insect host. Columns with these full obligate microbiome calculations are labeled with the term “oblholobiont” row 4. All further statistical analyses were performed on datasets with a numerical value in row 5, which provide analysis replicate IDs. Abbreviations in row 4 are as follows: oblsym (the metabolism of a single obligate symbiont), oblholobiont (typically the combined metabolism across separate obligate symbionts in the same host, although *Cryptocercus* cockroach data given this same label comprised data pooled across a gut metagenome and an obligate symbiont), mbiome (the combined metabolism across a complex microbiome as sampled through metagenomics), facsym (metabolism from a non-obligate, facultative symbiont), and facholobiont (the combined metabolism of an obligate symbionts and facultative symbionts from a single host).

**Table S41: Numbers of unique pectin-catabolizing proteins (KOs) encoded by symbiont genomes.** Searches against the data in Tables S33-S35 recovered proteins involved in pectin catabolism. Metadata on genomes are provided in rows 3-6 across Columns K-ES. Data for each protein/KO are found in these same columns across rows 7-23. Because of the way we extracted IMG data, ‘counts’ for (meta)genomes from IMG are capped at 1 per KO (despite higher gene copy numbers, in some instances). But NCBI KO counts occasionally exceeded 1. We thus coded KO presence/absence in rows 26-42, while the sum of unique proteins (KOs) identified in each genome is indicated in row 44. One additional point of interest is our combination of KO data across separate obligate symbionts from the same insect host. Columns with these full obligate microbiome calculations are labeled with the term “oblholobiont” row 4. All further statistical analyses were performed on datasets with a numerical value in row 5, which provide analysis replicate IDs. Abbreviations in row 4 are as follows: oblsym (the metabolism of a single obligate symbiont), oblholobiont (typically the combined metabolism across separate obligate symbionts in the same host, although *Cryptocercus* cockroach data given this same label comprised data pooled across a gut metagenome and an obligate symbiont), mbiome (the combined metabolism across a complex microbiome as sampled through metagenomics), facsym (metabolism from a non-obligate, facultative symbiont), and facholobiont (the combined metabolism of an obligate symbionts and facultative symbionts from a single host).

**Table S42: Pathway completeness for thiamine biosynthesis as encoded by symbiont genomes.** Searches against the data in Tables S33-S35 recovered proteins involved in thiamine synthesis. Metadata on genomes are provided in rows 3-6 across Columns K-ES. Data for each protein/KO are found in these same columns across rows 7-39. We consolidated data into a single row per biosynthetic step in rows 43-54, coalescing information for each step encoded by multiple KOs. Non-zero values for steps occasionally exceeded 1. Given our goal of assessing step presence/absence, we coded this information in rows 58-69. Pathway completeness was then calculated across four biosynthetic routes, as shown in rows 72-75. These calculations took the total number of represented steps, dividing that by the total number required per route (i.e. specific pathway). Steps are numbered in accordance to the numbering in Figure S9, and those included in each route are enumerated. Note that the asterisk starred and double asterisk starred pathways were the main foci of our data analyses. One additional point of interest is our combination of KO data across separate obligate symbionts from the same insect host. Columns with these full obligate microbiome calculations are labeled with the term “oblholobiont” row 4. All further statistical analyses were performed on datasets with a numerical value in row 5, which provide analysis replicate IDs. Abbreviations in row 4 are as follows: oblsym (the metabolism of a single obligate symbiont), oblholobiont (typically the combined metabolism across separate obligate symbionts in the same host, although *Cryptocercus* cockroach data given this same label comprised data pooled across a gut metagenome and an obligate symbiont), mbiome (the combined metabolism across a complex microbiome as sampled through metagenomics), facsym (metabolism from a non-obligate, facultative symbiont), and facholobiont (the combined metabolism of an obligate symbionts and facultative symbionts from a single host).

**Table S43. Pathway completeness for riboflavin biosynthesis as encoded by symbiont genomes.** Searches against the data in Tables S33-S35 recovered proteins involved in riboflavin synthesis. Metadata on genomes are provided in rows 3-6 across Columns K-ES. Data for each protein/KO are found in these same columns across rows 7-28. We consolidated data into a single row per biosynthetic step in rows 32-40, coalescing information for each step encoded by multiple KOs. Non-zero values for steps occasionally exceeded 1. Given our goal of assessing step presence/absence, we coded this as binary data in rows 43-51. Pathway completeness was then calculated across three biosynthetic routes, as shown in rows 53-55. These calculations took the total number of represented steps, dividing that by the total number required per route (i.e. specific pathway). Steps are numbered in accordance to the numbering in Figure S9, and those included in each route are enumerated. Note that the asterisk starred and double asterisk starred pathways were the main foci of our data analyses. One additional point of interest is our combination of KO data across separate obligate symbionts from the same insect host. Columns with these full obligate microbiome calculations are labeled with the term “oblholobiont” row 4. All further statistical analyses were performed on datasets with a numerical value in row 5, which provide analysis replicate IDs. Abbreviations in row 4 are as follows: oblsym (the metabolism of a single obligate symbiont), oblholobiont (typically the combined metabolism across separate obligate symbionts in the same host, although *Cryptocercus* cockroach data given this same label comprised data pooled across a gut metagenome and an obligate symbiont), mbiome (the combined metabolism across a complex microbiome as sampled through metagenomics), facsym (metabolism from a non-obligate, facultative symbiont), and facholobiont (the combined metabolism of an obligate symbionts and facultative symbionts from a single host).

**Table S44: Pathway completeness for niacin/nicotinate biosynthesis as encoded by symbiont genomes.** Searches against the data in Tables S33-S35 recovered proteins involved in niacin/nicotinate synthesis. Metadata on genomes are provided in rows 3-6 across Columns K-ES. Data for each protein/KO are found in these same columns across rows 7-48. We consolidated data into a single row per biosynthetic step in rows 52-67, coalescing information for each step encoded by multiple KOs. Non-zero values for steps occasionally exceeded 1. Given our goal of assessing step presence/absence, we coded this as binary data in rows 71-86. Pathway completeness was then calculated across fifteen biosynthetic routes, as shown in rows 88-102. These calculations took the total number of represented steps, dividing that by the total number required per route (i.e. specific pathway). Steps are numbered in accordance to the numbering in Figure S9, and those included in each route are enumerated. Note that the asterisk starred and double asterisk starred pathways were the main foci of our data analyses. One additional point of interest is our combination of KO data across separate obligate symbionts from the same insect host. Columns with these full obligate microbiome calculations are labeled with the term “oblholobiont” row 4. All further statistical analyses were performed on datasets with a numerical value in row 5, which provide analysis replicate IDs. Abbreviations in row 4 are as follows: oblsym (the metabolism of a single obligate symbiont), oblholobiont (typically the combined metabolism across separate obligate symbionts in the same host, although *Cryptocercus* cockroach data given this same label comprised data pooled across a gut metagenome and an obligate symbiont), mbiome (the combined metabolism across a complex microbiome as sampled through metagenomics), facsym (metabolism from a non-obligate, facultative symbiont), and facholobiont (the combined metabolism of an obligate symbionts and facultative symbionts from a single host).

**Table S45: Pathway completeness for pantothenate biosynthesis as encoded by symbiont genomes.** Searches against the data in Tables S33-S35 recovered proteins involved in pantothenate synthesis. Metadata on genomes are provided in rows 3-6 across Columns K-ES. Data for each protein/KO are found in these same columns across rows 7-28. We consolidated data into a single row per biosynthetic step in rows 32-39, coalescing information for each step encoded by multiple KOs. Non-zero values for steps occasionally exceeded 1. Given our goal of assessing step presence/absence, we coded this as binary data in rows 42-49. Pathway completeness was then calculated across three biosynthetic routes, as shown in rows 52-54. These calculations took the total number of represented steps, dividing that by the total number required per route (i.e. specific pathway). Steps are numbered in accordance to the numbering in Figure S9, and those included in each route are enumerated. Note that the asterisk starred and double asterisk starred pathways were the main foci of our data analyses. One additional point of interest is our combination of KO data across separate obligate symbionts from the same insect host. Columns with these full obligate microbiome calculations are labeled with the term “oblholobiont” row 4. All further statistical analyses were performed on datasets with a numerical value in row 5, which provide analysis replicate IDs. Abbreviations in row 4 are as follows: oblsym (the metabolism of a single obligate symbiont), oblholobiont (typically the combined metabolism across separate obligate symbionts in the same host, although *Cryptocercus* cockroach data given this same label comprised data pooled across a gut metagenome and an obligate symbiont), mbiome (the combined metabolism across a complex microbiome as sampled through metagenomics), facsym (metabolism from a non-obligate, facultative symbiont), and facholobiont (the combined metabolism of an obligate symbionts and facultative symbionts from a single host).

**Table S46: Pathway completeness for pyridoxine biosynthesis as encoded by symbiont genomes.** Searches against the data in Tables S33-S35 recovered proteins involved in pyridoxine synthesis. Metadata on genomes are provided in rows 3-6 across Columns K-ES. Data for each protein/KO are found in these same columns across rows 7-16. We consolidated data into a single row per biosynthetic step in rows 20-27, coalescing information for each step encoded by multiple KOs. Non-zero values for steps occasionally exceeded 1. To aid our goal of assessing step presence/absence, we coded this as binary data in rows 30-37. Pathway completeness was then calculated across three biosynthetic routes, as shown in rows 39-41. These calculations took the total number of represented steps, dividing that by the total number required per route (i.e. specific pathway). Steps are numbered in accordance to the numbering in Figure S9, and those included in each route are enumerated. Note that the asterisk starred and double asterisk starred pathways were the main foci of our data analyses. One additional point of interest is our combination of KO data across separate obligate symbionts from the same insect host. Columns with these full obligate microbiome calculations are labeled with the term “oblholobiont” row 4. All further statistical analyses were performed on datasets with a numerical value in row 5, which provide analysis replicate IDs. Abbreviations in row 4 are as follows: oblsym (the metabolism of a single obligate symbiont), oblholobiont (typically the combined metabolism across separate obligate symbionts in the same host, although *Cryptocercus* cockroach data given this same label comprised data pooled across a gut metagenome and an obligate symbiont), mbiome (the combined metabolism across a complex microbiome as sampled through metagenomics), facsym (metabolism from a non-obligate, facultative symbiont), and facholobiont (the combined metabolism of an obligate symbionts and facultative symbionts from a single host).

**Table S47: Pathway completeness for biotin biosynthesis as encoded by symbiont genomes.** Searches against the data in Tables S33-S35 recovered proteins involved in biotin synthesis. Metadata on genomes are provided in rows 3-6 across Columns K-ES. Data for each protein/KO are found in these same columns across rows 7-22 . We consolidated data into a single row per biosynthetic step in rows 26-39, coalescing information for each step encoded by multiple KOs. Non-zero values for steps occasionally exceeded 1. To aid our goal of assessing step presence/absence, we coded this as binary data in rows 43-56. Pathway completeness was then calculated across two biosynthetic routes, as shown in rows 58-59. These calculations took the total number of represented steps, dividing that by the total number required per route (i.e. specific pathway). Steps are numbered in accordance to the numbering in Figure S9, and those included in each route are enumerated. Note that the asterisk starred and double asterisk starred pathways were the main foci of our data analyses. One additional point of interest is our combination of KO data across separate obligate symbionts from the same insect host. Columns with these full obligate microbiome calculations are labeled with the term “oblholobiont” row 4. All further statistical analyses were performed on datasets with a numerical value in row 5, which provide analysis replicate IDs. Abbreviations in row 4 are as follows: oblsym (the metabolism of a single obligate symbiont), oblholobiont (typically the combined metabolism across separate obligate symbionts in the same host, although *Cryptocercus* cockroach data given this same label comprised data pooled across a gut metagenome and an obligate symbiont), mbiome (the combined metabolism across a complex microbiome as sampled through metagenomics), facsym (metabolism from a non-obligate, facultative symbiont), and facholobiont (the combined metabolism of an obligate symbionts and facultative symbionts from a single host).

**Table S48: Pathway completeness for folate biosynthesis as encoded by symbiont genomes.** Searches against the data in Tables S33-S35 recovered proteins involved in folate synthesis. Metadata on genomes are provided in rows 3-6 across Columns K-ES. Data for each protein/KO are found in these same columns across rows 7-48. We consolidated data into a single row per biosynthetic step in rows 52-66, coalescing information for each step encoded by multiple KOs. Non-zero values for steps occasionally exceeded 1. To aid our goal of assessing step presence/absence, we coded this as binary data in rows 82-94 with the exception of step 4. With this holoenzyme being encoded by 2 subunits, we coded the genome as 0.5 if one of two subunits was present, and 1 if both were present, using a series of calculations to infer which – if either of the subunits was present (rows 68-row79). Pathway completeness was then calculated across eight biosynthetic routes, as shown in rows 96-100 and 106-107. These calculations took the total number of represented steps (or half-steps), dividing that by the total number required per route (i.e. specific pathway). Steps are numbered in accordance to the numbering in Figure S9, and those included in each route are enumerated. Note that the asterisk starred and double asterisk starred pathways were the main foci of our data analyses. For folate, these corresponded to the pathways with the maximum level of completeness. One additional point of interest is our combination of KO data across separate obligate symbionts from the same insect host. Columns with these full obligate microbiome calculations are labeled with the term “oblholobiont” row 4. All further statistical analyses were performed on datasets with a numerical value in row 5, which provide analysis replicate IDs. Abbreviations in row 4 are as follows: oblsym (the metabolism of a single obligate symbiont), oblholobiont (typically the combined metabolism across separate obligate symbionts in the same host, although *Cryptocercus* cockroach data given this same label comprised data pooled across a gut metagenome and an obligate symbiont), mbiome (the combined metabolism across a complex microbiome as sampled through metagenomics), facsym (metabolism from a non-obligate, facultative symbiont), and facholobiont (the combined metabolism of an obligate symbionts and facultative symbionts from a single host).

**Table S49: Pathway completeness for histidine biosynthesis as encoded by symbiont genomes.** Searches against the data in Tables S33-S35 recovered proteins involved in histidine synthesis. Metadata on genomes are provided in rows 3-6 across Columns K-ES. Data for each protein/KO were found in these same columns across rows 7-22. We consolidated data into a single row per biosynthetic step in rows 25-34, coalescing information for each step encoded by multiple KOs. Non-zero values for steps occasionally exceeded 1. To aid our goal of assessing step presence/absence, we coded this as binary data in rows 36-44, with the exception of step 5. With some versions of the step 5-encoding enzyme being encoded by 2 subunits, we coded the genome as 0.5 if one of two subunits was present, and 1 if both were present. Pathway completeness was then calculated across one biosynthetic route, as shown in row 46. These calculations took the total number of represented steps (or half-steps), dividing that by the total number required for the whole pathway (i.e. 9). Steps are numbered in accordance to the numbering in Figure S9. One additional point of interest is our combination of KO data across separate obligate symbionts from the same insect host. Columns with these full obligate microbiome calculations are labeled with the term “oblholobiont” row 4. All further statistical analyses were performed on datasets with a numerical value in row 5, which provide analysis replicate IDs. Abbreviations in row 4 are as follows: oblsym (the metabolism of a single obligate symbiont), oblholobiont (typically the combined metabolism across separate obligate symbionts in the same host, although *Cryptocercus* cockroach data given this same label comprised data pooled across a gut metagenome and an obligate symbiont), mbiome (the combined metabolism across a complex microbiome as sampled through metagenomics), facsym (metabolism from a non-obligate, facultative symbiont), and facholobiont (the combined metabolism of an obligate symbionts and facultative symbionts from a single host).

**Table S50: Pathway completeness for chorismate biosynthesis as encoded by symbiont genomes.** Searches against the data in Tables S33-S35 recovered proteins involved in chorismate synthesis. Metadata on genomes are provided in rows 3-6 across Columns K-ES. Data for each protein/KO are found in these same columns across rows 7-24. We consolidated data into a single row per biosynthetic step in rows 27-33, coalescing information for each step encoded by multiple KOs. Non-zero values for steps occasionally exceeded 1. To aid our goal of assessing step presence/absence, we coded this as binary data in rows 35-41. Pathway completeness was then calculated across one biosynthetic route, as shown in row 43. These calculations took the total number of represented steps, dividing that by the total number required for the whole pathway (i.e. 7). Steps are numbered in accordance to the numbering in Figure S9. One additional point of interest is our combination of KO data across separate obligate symbionts from the same insect host. Columns with these full obligate microbiome calculations are labeled with the term “oblholobiont” row 4. All further statistical analyses were performed on datasets with a numerical value in row 5, which provide analysis replicate IDs. Abbreviations in row 4 are as follows: oblsym (the metabolism of a single obligate symbiont), oblholobiont (typically the combined metabolism across separate obligate symbionts in the same host, although *Cryptocercus* cockroach data given this same label comprised data pooled across a gut metagenome and an obligate symbiont), mbiome (the combined metabolism across a complex microbiome as sampled through metagenomics), facsym (metabolism from a non-obligate, facultative symbiont), and facholobiont (the combined metabolism of an obligate symbionts and facultative symbionts from a single host).

**Table S51: Pathway completeness for tyrosine biosynthesis as encoded by symbiont genomes.** Searches against the data in Tables S33-S35 recovered proteins involved in tyrosine synthesis. Metadata on genomes are provided in rows 3-6 across Columns K-ES. Data for each protein/KO are found in these same columns across rows 7-56. We consolidated data into a single row per biosynthetic step in rows 59-68, coalescing information for each step encoded by multiple KOs. Non-zero values for steps occasionally exceeded 1. To aid our goal of assessing step presence/absence, we coded this as binary data in rows 70-79. Pathway completeness was then calculated across five biosynthetic routes, as shown in rows 82-86. These calculations took the total number of represented steps, dividing that by the total number required for the whole pathway. We took the maximum value across these pathways (row 88) as our estimate of tyrosine pathway completeness. Steps are numbered in accordance to the numbering in Figure S9. One additional point of interest is our combination of KO data across separate obligate symbionts from the same insect host. Columns with these full obligate microbiome calculations are labeled with the term “oblholobiont” row 4. All further statistical analyses were performed on datasets with a numerical value in row 5, which provide analysis replicate IDs. Abbreviations in row 4 are as follows: oblsym (the metabolism of a single obligate symbiont), oblholobiont (typically the combined metabolism across separate obligate symbionts in the same host, although *Cryptocercus* cockroach data given this same label comprised data pooled across a gut metagenome and an obligate symbiont), mbiome (the combined metabolism across a complex microbiome as sampled through metagenomics), facsym (metabolism from a non-obligate, facultative symbiont), and facholobiont (the combined metabolism of an obligate symbionts and facultative symbionts from a single host).

**Table S52: Pathway completeness for tryptophan biosynthesis as encoded by symbiont genomes.** Searches against the data in Tables S33-S35 recovered proteins involved in tryptophan synthesis. Metadata on genomes are provided in rows 3-6 across Columns K-ES. Data for each protein/KO are found in these same columns across rows 7-21. We consolidated data into a single row per biosynthetic step in rows 24-38, coalescing information for each step encoded by multiple KOs. Non-zero values for steps occasionally exceeded 1. To aid our goal of assessing step presence/absence, we coded this as binary data in rows 38-42, with the exceptions of steps 1 and 5. With some versions of the step 1- and step 5-encoding enzymes being encoded by 2 subunits, we coded the genome as 0.5 if one of two subunits was present, and 1 if both were present. Pathway completeness was then calculated across one biosynthetic route, as shown in row 44. These calculations took the total number of represented steps (or half-steps), dividing that by the total number required for the whole pathway . Steps are numbered in accordance to the numbering in Figure S9. One additional point of interest is our combination of KO data across separate obligate symbionts from the same insect host. Columns with these full obligate microbiome calculations are labeled with the term “oblholobiont” row 4. All further statistical analyses were performed on datasets with a numerical value in row 5, which provide analysis replicate IDs. Abbreviations in row 4 are as follows: oblsym (the metabolism of a single obligate symbiont), oblholobiont (typically the combined metabolism across separate obligate symbionts in the same host, although *Cryptocercus* cockroach data given this same label comprised data pooled across a gut metagenome and an obligate symbiont), mbiome (the combined metabolism across a complex microbiome as sampled through metagenomics), facsym (metabolism from a non-obligate, facultative symbiont), and facholobiont (the combined metabolism of an obligate symbionts and facultative symbionts from a single host).

**Table S53: Pathway completeness for phenylalanine biosynthesis as encoded by symbiont genomes.** Searches against the data in Tables S33-S35 recovered proteins involved in phenylalanine synthesis. Metadata on genomes are provided in rows 3-6 across Columns K-ES. Data for each protein/KO are found in these same columns across rows 7-32. We consolidated data into a single row per biosynthetic step in rows 35-40, coalescing information for each step encoded by multiple KOs. Non-zero values for steps occasionally exceeded 1. To aid our goal of assessing step presence/absence, we coded this as binary data in rows 42-47. Pathway completeness was then calculated across three biosynthetic routes, as shown in rows 49-51. These calculations took the total number of represented steps, dividing that by the total number required for the whole pathway. We took the maximum value across these pathways (row 53) as our estimate of phenylalanine pathway completeness. Steps are numbered in accordance to the numbering in Figure S9. One additional point of interest is our combination of KO data across separate obligate symbionts from the same insect host. Columns with these full obligate microbiome calculations are labeled with the term “oblholobiont” row 4. All further statistical analyses were performed on datasets with a numerical value in row 5, which provide analysis replicate IDs. Abbreviations in row 4 are as follows: oblsym (the metabolism of a single obligate symbiont), oblholobiont (typically the combined metabolism across separate obligate symbionts in the same host, although *Cryptocercus* cockroach data given this same label comprised data pooled across a gut metagenome and an obligate symbiont), mbiome (the combined metabolism across a complex microbiome as sampled through metagenomics), facsym (metabolism from a non-obligate, facultative symbiont), and facholobiont (the combined metabolism of an obligate symbionts and facultative symbionts from a single host).

**Table S54: Pathway completeness for arginine biosynthesis as encoded by symbiont genomes** Searches against the data in Tables S33-S35 recovered proteins involved in arginine synthesis. Metadata on genomes are provided in rows 3-6 across Columns K-ES. Data for each protein/KO are found in these same columns across rows 7-34. We consolidated data into a single row per biosynthetic step in rows 36-52, coalescing information for each step encoded by multiple KOs. Non-zero values for steps occasionally exceeded 1. To aid our goal of assessing step presence/absence, we coded this as binary data in rows 54-69. Pathway completeness was then calculated across four biosynthetic routes, as shown in rows 71-74. These calculations took the total number of represented steps, dividing that by the total number required for the whole pathway. We took the maximum value across these pathways (row 76) as our estimate of arginine pathway completeness. Steps are numbered in accordance to the numbering in Figure S9. One additional point of interest is our combination of KO data across separate obligate symbionts from the same insect host. Columns with these full obligate microbiome calculations are labeled with the term “oblholobiont” row 4. All further statistical analyses were performed on datasets with a numerical value in row 5, which provide analysis replicate IDs. Abbreviations in row 4 are as follows: oblsym (the metabolism of a single obligate symbiont), oblholobiont (typically the combined metabolism across separate obligate symbionts in the same host, although *Cryptocercus* cockroach data given this same label comprised data pooled across a gut metagenome and an obligate symbiont), mbiome (the combined metabolism across a complex microbiome as sampled through metagenomics), facsym (metabolism from a non-obligate, facultative symbiont), and facholobiont (the combined metabolism of an obligate symbionts and facultative symbionts from a single host).

**Table S55: Pathway completeness for lysine biosynthesis as encoded by symbiont genomes.** Searches against the data in Tables S33-S35 recovered proteins involved in lysine synthesis. Metadata on genomes are provided in rows 3-6 across Columns K-ES. Data for each protein/KO are found in these same columns across rows 7-46. We consolidated data into a single row per biosynthetic step in rows 49-77, coalescing information for each step encoded by multiple KOs. Non-zero values for steps occasionally exceeded 1. To aid our goal of assessing step presence/absence, we coded this as binary data in rows 79-107. Pathway completeness was then calculated across six biosynthetic routes, as shown in rows 109-114. These calculations took the total number of represented steps, dividing that by the total number required for the whole pathway. We took the maximum value across these pathways (row 116) as our estimate of lysine pathway completeness. Steps are numbered in accordance to the numbering in Figure S9. One additional point of interest is our combination of KO data across separate obligate symbionts from the same insect host. Columns with these full obligate microbiome calculations are labeled with the term “oblholobiont” row 4. All further statistical analyses were performed on datasets with a numerical value in row 5, which provide analysis replicate IDs. Abbreviations in row 4 are as follows: oblsym (the metabolism of a single obligate symbiont), oblholobiont (typically the combined metabolism across separate obligate symbionts in the same host, although *Cryptocercus* cockroach data given this same label comprised data pooled across a gut metagenome and an obligate symbiont), mbiome (the combined metabolism across a complex microbiome as sampled through metagenomics), facsym (metabolism from a non-obligate, facultative symbiont), and facholobiont (the combined metabolism of an obligate symbionts and facultative symbionts from a single host).

**Table S56: Pathway completeness for threonine biosynthesis as encoded by symbiont genomes.** Searches against the data in Tables S33-S35 recovered proteins involved in threonine synthesis. Metadata on genomes are provided in rows 3-6 across Columns K-ES. Data for each protein/KO are found in these same columns across rows 7-16. We consolidated data into a single row per biosynthetic step in rows 19-23, coalescing information for each step encoded by multiple KOs. Non-zero values for steps occasionally exceeded 1. To aid our goal of assessing step presence/absence, we coded this as binary data in rows 25-29. Pathway completeness was then calculated across one biosynthetic routes, as shown in row 31. These calculations took the total number of represented steps, dividing that by the total number required for the whole pathway. Steps are numbered in accordance to the numbering in Figure S9. One additional point of interest is our combination of KO data across separate obligate symbionts from the same insect host. Columns with these full obligate microbiome calculations are labeled with the term “oblholobiont” row 4. All further statistical analyses were performed on datasets with a numerical value in row 5, which provide analysis replicate IDs. Abbreviations in row 4 are as follows: oblsym (the metabolism of a single obligate symbiont), oblholobiont (typically the combined metabolism across separate obligate symbionts in the same host, although *Cryptocercus* cockroach data given this same label comprised data pooled across a gut metagenome and an obligate symbiont), mbiome (the combined metabolism across a complex microbiome as sampled through metagenomics), facsym (metabolism from a non-obligate, facultative symbiont), and facholobiont (the combined metabolism of an obligate symbionts and facultative symbionts from a single host).

**Table S57: Pathway completeness for methionine biosynthesis as encoded by symbiont genomes.** Searches against the data in Tables S33-S35 recovered proteins involved in methionine synthesis. Metadata on genomes are provided in rows 3-6 across Columns K-ES. Data for each protein/KO are found in these same columns across rows 7-29. We consolidated data into a single row per biosynthetic step in rows 31-43, coalescing information for each step encoded by multiple KOs. Non-zero values for steps occasionally exceeded 1. To aid our goal of assessing step presence/absence, we coded this as binary data in rows 45-56. Pathway completeness was then calculated across five biosynthetic routes, as shown in rows 58-62. These calculations took the total number of represented steps, dividing that by the total number required for the whole pathway. We took the maximum value across these pathways (row 64) as our estimate of methionine pathway completeness. Steps are numbered in accordance to the numbering in Figure S9. One additional point of interest is our combination of KO data across separate obligate symbionts from the same insect host. Columns with these full obligate microbiome calculations are labeled with the term “oblholobiont” row 4. All further statistical analyses were performed on datasets with a numerical value in row 5, which provide analysis replicate IDs. Abbreviations in row 4 are as follows: oblsym (the metabolism of a single obligate symbiont), oblholobiont (typically the combined metabolism across separate obligate symbionts in the same host, although *Cryptocercus* cockroach data given this same label comprised data pooled across a gut metagenome and an obligate symbiont), mbiome (the combined metabolism across a complex microbiome as sampled through metagenomics), facsym (metabolism from a non-obligate, facultative symbiont), and facholobiont (the combined metabolism of an obligate symbionts and facultative symbionts from a single host).

**Table S58: Pathway completeness for valine biosynthesis as encoded by symbiont genomes.** Searches against the data in Tables S33-S35 recovered proteins involved in valine synthesis. Metadata on genomes are provided in rows 3-6 across Columns K-ES. Data for each protein/KO are found in these same columns across rows 7-12. We consolidated data into a single row per biosynthetic step in rows 19-24, coalescing information for each step encoded by multiple KOs. Non-zero values for steps occasionally exceeded 1. To aid our goal of assessing step presence/absence, we coded this as binary data in rows 26-31. Pathway completeness was then calculated across two biosynthetic routes, as shown in rows 33-34. These calculations took the total number of represented steps, dividing that by the total number required for the whole pathway. We took the maximum value across these pathways (row 36) as our estimate of valine pathway completeness. Steps are numbered in accordance to the numbering in Figure S9. One additional point of interest is our combination of KO data across separate obligate symbionts from the same insect host. Columns with these full obligate microbiome calculations are labeled with the term “oblholobiont” row 4. All further statistical analyses were performed on datasets with a numerical value in row 5, which provide analysis replicate IDs. Abbreviations in row 4 are as follows: oblsym (the metabolism of a single obligate symbiont), oblholobiont (typically the combined metabolism across separate obligate symbionts in the same host, although *Cryptocercus* cockroach data given this same label comprised data pooled across a gut metagenome and an obligate symbiont), mbiome (the combined metabolism across a complex microbiome as sampled through metagenomics), facsym (metabolism from a non-obligate, facultative symbiont), and facholobiont (the combined metabolism of an obligate symbionts and facultative symbionts from a single host).

**Table S59: Pathway completeness for isoleucine biosynthesis as encoded by symbiont genomes.** Searches against the data in Tables S33-S35 recovered proteins involved in isoleucine synthesis. Metadata on genomes are provided in rows 3-6 across Columns K-ES. Data for each protein/KO are found in these same columns across rows 7-20. We consolidated data into a single row per biosynthetic step in rows 23-32, coalescing information for each step encoded by multiple KOs. Non-zero values for steps occasionally exceeded 1. To aid our goal of assessing step presence/absence, we coded this as binary data in rows 38-47, with the exceptions of steps B and C. With some versions of the enzyme encoding these consecutive steps being encoded by 2 subunits, we coded the genome as 0.5 if one of two subunits was present, and 1 if both were present. Pathway completeness was then calculated across two biosynthetic routes, as shown in rows 49-50. These calculations took the total number of represented steps (or half-steps), dividing that by the total number required for the whole pathway. The maximum of these two values was used to represent isoleucine pathway completeness (row 52). Steps are numbered in accordance to the numbering in Figure S9. One additional point of interest is our combination of KO data across separate obligate symbionts from the same insect host. Columns with these full obligate microbiome calculations are labeled with the term “oblholobiont” row 4. All further statistical analyses were performed on datasets with a numerical value in row 5, which provide analysis replicate IDs. Abbreviations in row 4 are as follows: oblsym (the metabolism of a single obligate symbiont), oblholobiont (typically the combined metabolism across separate obligate symbionts in the same host, although *Cryptocercus* cockroach data given this same label comprised data pooled across a gut metagenome and an obligate symbiont), mbiome (the combined metabolism across a complex microbiome as sampled through metagenomics), facsym (metabolism from a non-obligate, facultative symbiont), and facholobiont (the combined metabolism of an obligate symbionts and facultative symbionts from a single host).

**Table S60: Pathway completeness for leucine biosynthesis as encoded by symbiont genomes.** Searches against the data in Tables S33-S35 recovered proteins involved in leucine synthesis. Metadata on genomes are provided in rows 3-6 across Columns K-ES. Data for each protein/KO are found in these same columns across rows 7-21. We consolidated data into a single row per biosynthetic step in rows 25-33, coalescing information for each step encoded by multiple KOs. Non-zero values for steps occasionally exceeded 1. To aid our goal of assessing step presence/absence, we coded this as binary data in rows 39-47, with the exceptions of steps 6 and 7. With some versions of the enzyme encoding these consecutive steps being encoded by 2 subunits, we coded the genome as 0.5 if one of two subunits was present, and 1 if both were present. Pathway completeness was then calculated across one biosynthetic route, as shown in row 49. These calculations took the total number of represented steps (or half-steps), dividing that by the total number required for the whole pathway. Steps are numbered in accordance to the numbering in Figure S9. One additional point of interest is our combination of KO data across separate obligate symbionts from the same insect host. Columns with these full obligate microbiome calculations are labeled with the term “oblholobiont” row 4. All further statistical analyses were performed on datasets with a numerical value in row 5, which provide analysis replicate IDs. Abbreviations in row 4 are as follows: oblsym (the metabolism of a single obligate symbiont), oblholobiont (typically the combined metabolism across separate obligate symbionts in the same host, although *Cryptocercus* cockroach data given this same label comprised data pooled across a gut metagenome and an obligate symbiont), mbiome (the combined metabolism across a complex microbiome as sampled through metagenomics), facsym (metabolism from a non-obligate, facultative symbiont), and facholobiont (the combined metabolism of an obligate symbionts and facultative symbionts from a single host).

**Table S61: Notes on amino acid provisioning by symbionts, with an emphasis on *in vivo* studies.** Table contains descriptions of insects, symbionts, and experimental or ‘omic evidence for synthesis/provisioning of individual amino acids. Information in columns AN-AQ typically contains direct quotes from the cited research articles. Rows represent data from different insect species studied across separate publications.

**Table S62: Notes on B-vitamin provisioning by symbionts, with an emphasis on *in vivo* studies.** Table contains descriptions of insects, symbionts, and experimental or ‘omic evidence for synthesis/provisioning of individual B-vitamins. Rows represent data from different insect species studied across separate publications.

**Table S63: Notes on fiber and fermentation (e.g. SCFA) metabolism of symbionts, with an emphasis on *in vivo* studies.** Table contains descriptions of insects, symbionts, and experimental or ‘omic evidence for catabolism of cellulose, hemicellulose, lignin, and pectin. Also emphasized is symbiont production of short chain fatty acids (SCFA) through fermentative processes. Rows represent data from different insect species studied across separate publications.

**Table S64: Notes on nitrogen recycling metabolism by symbionts, with an emphasis on *in vivo* studies.** Table contains descriptions of insects, symbionts, and experimental or ‘omic evidence for catabolism of compounds in the uricolytic pathway. Focus was primarily on uric acid and urea. Rows represent data from different insect species studied across separate publications.

**Table S65: Notes on nitrogen fixation metabolism by symbionts, with an emphasis on *in vivo* studies.** Table contains descriptions of insects, symbionts, and experimental or ‘omic evidence for fixation of atmospheric di-nitrogen. Rows represent data from different insect species studied across separate publications.

**Table S66: *In vivo* amino acid metabolism by symbionts with known or plausible benefits to hosts – summarized at host species level.** Types of supporting evidence for distinct symbioses are emphasized in this table. We indicate symbiont lineage origin numbers when (plausible) symbiont benefit can be assigned to a known obligate symbiosis.

**Table S67: *In vivo* B-vitamin metabolism by symbionts with known or plausible benefits to hosts – summarized at host species level.** Types of supporting evidence for distinct symbioses are emphasized in this table. We indicate symbiont lineage origin numbers when (plausible) symbiont benefit can be assigned to a known obligate symbiosis.

**Table S68: *In vivo* fiber and fermentation metabolism by symbionts with known or plausible benefits to hosts – summarized at host species level.** Types of supporting evidence for distinct symbioses are emphasized in this table. We indicate symbiont lineage origin numbers when (plausible) symbiont benefit can be assigned to a known obligate symbiosis.

**Table S69: *In vivo* nitrogen recycling metabolism by symbionts with known or plausible benefits to hosts – summarized at host species level.** Types of supporting evidence for distinct symbioses are emphasized in this table. We indicate symbiont lineage origin numbers when (plausible) symbiont benefit can be assigned to a known obligate symbiosis.

**Table S70: *In vivo* nitrogen fixation metabolism by symbionts with known or plausible benefits to hosts – summarized at host species level.** Types of supporting evidence for distinct symbioses are emphasized in this table. We indicate symbiont lineage origin numbers when (plausible) symbiont benefit can be assigned to a known obligate symbiosis.

**Table S71: Pathway completeness for B-vitamin biosynthesis across each possible pathway.** The proportions of fulfilled biosynthetic steps for seven B-vitamins, based on protein/KO counts and completeness calculations in Tables S42-S48.

**Table S72: Pathway completeness for B-vitamin biosynthesis across focal pathways, with pseudoreplicate data combined.** Completeness values for pathways with * and ** designations in Table S71 are shown here. ** refers to pathways that are not redundant with host-encoded genes. Metadata in columns A-L are in bold if they combine results from multiple analysis replicates (column C). In these cases, completeness values represent averages across the symbioses of several related host species. This approach minimized impacts of pseudoreplication. In short, data were combined among hosts when they harbored the same symbiont from an ancestral acquisition. This would pertain, for instance, to the obligate holobionts of aphids with a *Buchnera* symbiont regardless of whether this bacterium lives alongside a co-obligate symbiont. Treated separately from these aphids were those from the *Geopemphigus* genus where *Buchnera* was replaced by a symbiont from the Bacteroidetes. Also separate were symbioses from psyllids, whiteflies, scale insects, and varying auchennorhynchans despite these symbioses likely descending from the presence of bacteriocyte-dwelling bacteria in their common ancestor. These other insects harbor distinct types of obligate symbiotic microbes, whose independent acquisition has enabled separate trajectories for genome evolution. Included here in column AA are the average genome sizes across all obligate symbionts/obligate holobionts in the hosts of interest (data from Table S31). Also included are average 16S rRNA gene sequence identities of obligate symbionts vs. free-living bacteria (Table S30). We note that the total numbers of base pairs in column AA for metagenomes reveal total assembly size. For metagenomes we, further, did not compute 16S rRNA sequence identities due to their high diversity.

**Table S73: B-vitamin pathway completeness versus dietary nutrition – input for linear regression analysis.** Included here are B-vitamin pathway completeness values from Table S72, along with B-vitamin quantities of particular dietary items consumed by our focal insects (see averages in **Tables S19-S20**). Complementing the full dataset (rows 3-54) was a secondary dataset (rows 57-90), which excluded all metagenome data and data from hosts where obligate symbionts had >95% 16S rRNA gene sequence identity to free-living bacteria. For multi-species obligate microbiomes, we took the average of each sequence identity to assess this criterion. Genomes of recently domesticated symbionts, or those with alternative lifestyles in free-living habitats, are less likely to have lost genes superfluous to a symbiotic lifestyle. Symbionts across diverse gut microbiomes are also unlikely to have undergone the same genomic streamlining seen in many low-diversity obligate symbioses. Additional details warrant further explanation. First, for animal tissue diets (the predatory/scavenging/parasitic lifestyle), we obtained B-vitamin values by averaging estimated values across crustacean, vertebrate, insect, and mollusk tissues. Second, B-vitamin values were obtained for sweet potato roots from the USDA database, as one included insect was argued to feed upon this food (treated as a type of root in other analyses). Third, for reed beetles (rows 27 & 82), we coded diet as phloem (the larval diet), despite a leaf chewing diet in adulthood. Similarly, for soft-winged flower beetles (rows 18 & 72), we coded diet as animal tissue (the larval diet), despite pollen consumption at the adult stage. A range of insects in this analysis were argued to be omnivorous or to have ambiguous diets – no nutrient data were included for these. Within this group were a number of insects eating dead wood tissue, which was surmised likely to contain a heavy microbial biomass.

**Table S74: Linear regression statistics on symbiont B-vitamin completeness vs. log-transformed dietary B-vitamin data.** Statistics were run on the GraphPad website (<https://www.graphpad.com/quickcalcs/linear1/>) on log-transformed dietary B-vitamin data (x-variable) and untransformed completeness estimates (y-variable). Slopes of best-fit regression lines significantly different from zero were given blue highlighting if the slope was negative (as expected), and yellow highlighting if the slope was positive.

**Table S75: T-tests on symbiont B-vitamin completeness vs. dietary B-vitamin levels above or below average insect requirements.** One-tailed t-tests (with unequal variance) were run in Microsoft Excel. Dietary B-vitamin thresholds were the averages from Table S28. Data analyses were only run after excluding all metagenome data and data from hosts where obligate symbionts had >95% 16S rRNA gene sequence identity to free-living bacteria, on average. See TableS73 legend for explanation. P-values from t-tests are in row 36. Significant values were color-coded, with those in blue matching the expected direction (i.e. higher symbiont B-vitamin pathway completeness for insects with diets falling below insect B-vitamin requirements).

**Table S76: Pathway completeness for chorismate biosynthesis and amino acid biosynthesis; protein/KO counts from fiber catabolism, nitrogen recycling, and nitrogen fixation.** The proportions of fulfilled biosynthetic steps for chorismate and several amino acids are presented in columns Q-AB. These were based on protein/KO counts and completeness calculations in Tables S49-S60. Also presented are numbers of unique KOs encoded by symbiont genomes shaping catabolism of plant cell wall fibers cellulose, hemicellulose, pectin, and lignin, or those impacting the recycling or fixation of nitrogen (columns K-P). Data for these latter functions were obtained from protein/KO counts in Tables S36-S41.

**Table S77: Non-vitamin pathway completeness/KO counts, and average % 16S rRNA identity of obligate symbionts vs. free-living bacteria after combining pseudoreplicates.** Completeness or KO count values for pathways from Table S76 are shown after combining pseudoreplicates. In short, metadata in columns A-L are in bold if they combine results from multiple analysis replicates (see column C). In these cases, completeness/KO count values represent averages across the symbioses of several related host species. This averaging was only done for symbioses of hosts harboring the same symbiont from an ancestral acquisition. This would pertain, for instance, to the obligate holobionts of aphids with a *Buchnera* symbiont regardless of whether this bacterium lived alongside a co-obligate symbiont. Treated separately from these aphids were those from the *Geopemphigus* genus where *Buchnera* was replaced by a symbiont from the Bacteroidetes. Also separate were symbioses from psyllids, whiteflies, scale insects, and varying auchennorhynchans despite these symbioses likely descending from the presence of bacteriocyte-dwelling bacteria in their common ancestor. These latter insects harbor distinct types of obligate symbiotic microbes, whose independent acquisition enabled separate trajectories for genome evolution. In column AE we include the percent identity between 16S rRNA genes of symbionts vs. their closest free-living relative (Table S30). These were first averaged across all members of each low diversity (i.e. n=1 to n=3 species) obligate microbiome, before averaging across pseudoreplicates.

**Table S78: Linear regression inputs for non-vitamin symbiont functions referenced in Table S76.** Included here are non-vitamin KO counts and pathway completeness values from **Table S77**, along with nutrient quantities of particular dietary items consumed by our focal insects (see averages in **Tables S19-S20**). We used % dietary nitrogen as a predictor of symbionts’ chorismate and amino acid biosynthesis, and for the numbers of symbiont-encoded KOs shaping the recycling and fixation of nitrogen. For an x-variable predictor of symbionts’ fiber catabolizing abilities, we used the percent neutral detergent fiber from the diet. Complementing the full dataset (rows 3-50) was a secondary dataset (rows 53-83), which excluded all metagenome data and data from hosts where obligate symbionts had >95% 16S rRNA gene sequence identity to free-living bacteria. Justification for these omissions is provided in the legend for Table S73. Additional details warrant further explanation. First, for many nitrogen values we took the mid-range values from our literature searches. When there was more than one food category with %nitrogen for a broader category, we averaged the mid-range values. Roots, for example, combined data from the categories of ‘roots’, ‘roots_rhizome’, and ‘roots_storage. Animal tissue combined data from insect and vertebrate tissue. The microbes food category combined data from slime molds, fungi, fungi_yeast, Archaea, protists, lichen, and (Eu)bacteria. Algae combined diatoms, microalgae and macroalgae. Note that fruit did not include unripened fruit. Third, for reed beetles (rows 27 & 82), we coded diet as phloem (the larval diet) when modeling nitrogen as our x-variable, and as leaves (the adult diet), when our x-variable was fiber. As for the B-vitamin analyses, above, we used the larval diet for soft-winged flower beetles (animal tissue). A range of insects in this analysis were argued to be omnivorous or to have ambiguous diets – no nutrient data were included for these. Within this group were a number of insects eating dead wood tissue, which was surmised likely to contain a heavy microbial biomass.

**Table S79: Linear regression stats for non-vitamin symbiont functions referenced in Table S76.** Statistics were run on the GraphPad website (<https://www.graphpad.com/quickcalcs/linear1/>) on and pathway completeness/KO count estimates (y-variables) vs. select dietary nutrient data (x-variables). Slopes of best-fit regression lines significantly different from zero were given blue highlighting if the slope was negative (as expected for amino acid completeness vs. % nitrogen data). For significant slope results in our KO count data, where we expected positive correlations in analyses with % fiber as the x-variable, highlighting for positive slopes was in yellow. Note that due to nearly all nitrogen fixation proteins being detected in metagenomes (outside of one instance), we did not run statistics on that function vs. dietary nutrient relationship.

**Table S80: Summaries of stats from Table S79.**

**Table S81: Symbiont amino acid pathway completeness vs. time since domestication for insects on blood, phloem, and seed diets.** For all analyses we used linear regression; y-variable = pathway completeness for AA in question; x-variable = 16S rRNA identity to most closely related free-living bacterium (a proxy for time since domestication). X- and Y-variable data come from Table S77. Significant and borderline-significant p-values are given yellow and beige highlighting. For each tissue-specific analysis, we included all relevant obligate microbiomes (young and ancient) except for those sampled through metagenomics. For multi-species microbiomes we used the average 16s rRNA identity across co-obligate symbionts.

**Table S82: Encoded steps of B-vitamin and AA biosynthetic pathways across obligate microbiomes of blood-, seed-, and phloem-feeders.** Pathway steps with varying numbers of "*" in column Z are those that overlap across multiple pathways (e.g. the same enzymes used for steps 2, 3, 4, and 5 of the Ile pathway are used to fulfill steps 1, 2, 3, and 4 of the Leu pathway). This helps to show partial non-independence of some pathway completeness calculations.

**Table S83: Raw data for % dry weight amino acid content from varying insect foods.** Table includes database and publication sources for each datapoint.

**Table S84: Species-level averages of % dry weight amino acid content from varying insect foods.** Species averaged from multiple datapoints in Table S82 are in bold font.

**Table S85: Summary statistics for % dry weight amino acid content from varying insect foods.** Includes averages, sample sizes, minimum and maximum values, and standard deviation for each potential food.

**Table S86: Linear regression examining the relationship between % amino acid content vs. % nitrogen of varying insect foods.** Excluded blood & root_storage tissue because of n=1 for amino acids and nitrogen, respectively. Inputs for % amino acid data are in Table S84. Those for %nitrogen are in column B.

**Table S87 - Amino acid composition of insects averaged at the order level.** Data for percent amino acid composition (dry weight) in Table S83 were averaged at the order level for this table. Relative composition of each amino acid was then computed for each order.
