# Supplementary material for: Symbiont‐Derived Key Innovations Across the Diversity and Diets of Herbivorous Insects
Source: Mol Ecol. 2026 Nov 7;35(21):e70615. doi: 10.1111/mec.70615 (PMC13743980; doi:10.1111/mec.70615)
Supplement: Supplementary file 2 [file MEC-35-e70615-s002.zip › supplementary figure S5 - phylogeny.pdf]

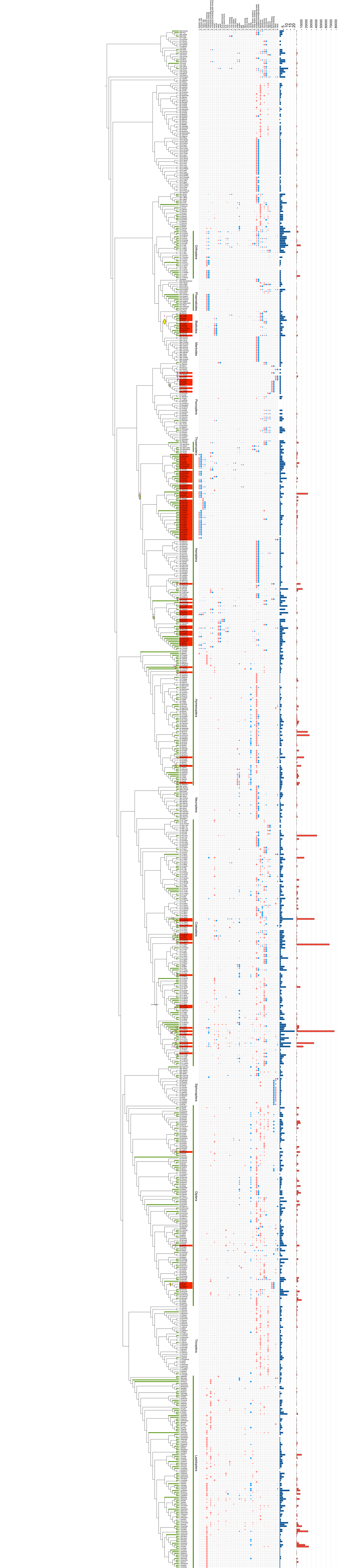

Orthoptera
Phasmatodea
Blattodea
Mantodea
Plecoptera
Thysanoptera
Hemiptera
Hymenoptera
Neuroptera
Coleoptera
Siphonaptera
Diptera
Trichoptera
Lepidoptera
