# Supplementary material for: Symbiont‐Derived Key Innovations Across the Diversity and Diets of Herbivorous Insects
Source: Mol Ecol. 2026 Nov 7;35(21):e70615. doi: 10.1111/mec.70615 (PMC13743980; doi:10.1111/mec.70615)
Supplement: Supplementary file 2 [file MEC-35-e70615-s002.zip › Supplementary Figure S6 boxplot_nutrients_no-log-transformed-20240707.pdf]

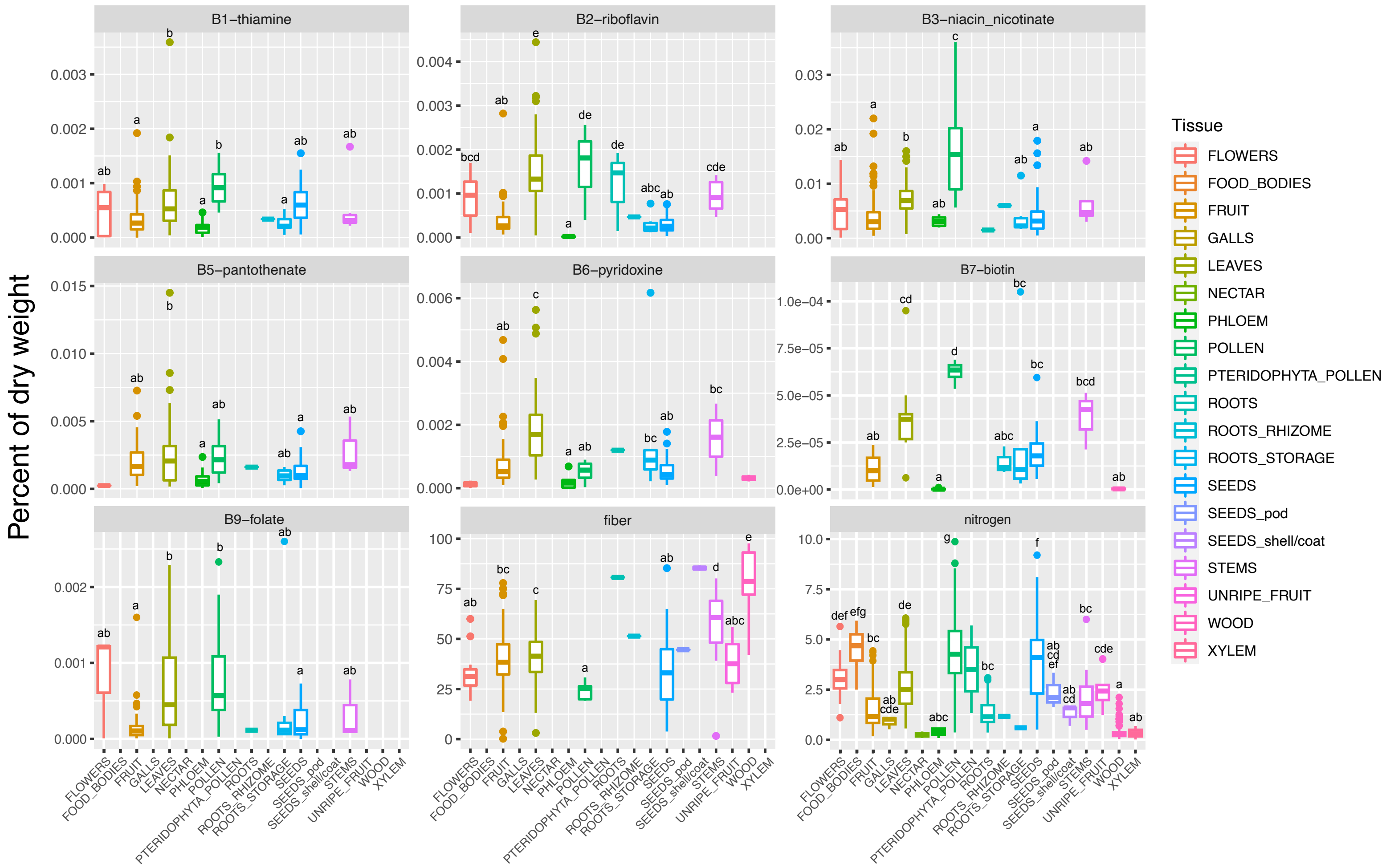

B1−thiamine
B2−riboflavin
B3−niacin_nicotinate
B5−pantothenate
B6−pyridoxine
B7−biotin
B9−folate
fiber
nitrogen
Percent of dry weight
Tissue
FLOWERS
FOOD_BODIES
FRUIT
GALLS
LEAVES
NECTAR
PHLOEM
POLLEN
PTERIDOPHYTA_POLLEN
ROOTS
ROOTS_RHIZOME
ROOTS_STORAGE
SEEDS
SEEDS_pod
SEEDS_shell/coat
STEMS
UNRIPE_FRUIT
WOOD
XYLEM
