# Supplementary material for: Symbiont‐Derived Key Innovations Across the Diversity and Diets of Herbivorous Insects
Source: Mol Ecol. 2026 Nov 7;35(21):e70615. doi: 10.1111/mec.70615 (PMC13743980; doi:10.1111/mec.70615)
Supplement: Supplementary file 3 [file MEC-35-e70615-s003.docx]

**Supporting Information Methods, Results, and Discussion for:**

**Title:** Symbiont-derived key innovations across the diversity and diets of herbivorous insects

**Authors:** Jacob A. Russell*, Hannah Smith*, Benoit Bechade, Christian Cabuslay, Melissa Carpenter, Daniel Freeman, Allison Hansen, Clesson Higashi, Yi Hu, Linyao Peng, Abigail Williams, and Patrick Abbot

Note: Most supplementary table citations refer to table numbering used for our Zenodo data repository submission (<https://doi.org/10.5281/zenodo.19607748>). Those not explicitly linked to this resource are citations of Supplementary Tables submitted to the Molecular Ecology website.

**Table of Contents:**

Supporting Information Methods

| I. Objective 1 – insect diet vs. obligate symbiosis |  |
| --- | --- |
| I.a. Insect diversity counts | **p.6** |
| I.b. Research on insect diets - goals and general purposes | **pp.6-7** |
| I.c. Research on insect diets - caveats and special  considerations | **pp.7-9** |
| I.d. Research on obligate symbioses | **pp.9-10** |
|  |  |
| II. Objective 2 – part 1 – plant nutrition |  |
| II.a. Plant nutrition search strategy and data extraction –  general overview | **pp.10-12** |
| II.b. Plant nutrition search strategy and data extraction –  assumptions made for wood | **p.12** |
| II.c. Plant nutrition search strategy and data extraction – % dry  weight estimates for phloem | **p.13** |
| II.d. Plant nutrition search strategy and data extraction –  assumptions made for nectar and xylem | **p.14** |
| II.e. Nutrition search strategy and data extraction –  assumptions made for blood | **p.14** |
| II.f. Plant nutrition search strategy and data extraction – QC  assessment | **pp.14-15** |
| II.g. Plant nutrition statistics | **p.15** |
| III. Objective 2 - part 2 - symbiont vs. nutrient statistics | **pp.15-16** |
| IV. Objective 2 - part 3 - symbiont metabolism & function |  |
| IV.a. Symbiont function – *in vivo* study | **pp.16-17** |
| IV.b. Symbiont metabolism – genomic study | **pp.17-19** |

Supporting Information Results

| V. Limits to our insect diet assessment | **p.19** |
| --- | --- |
| VI. Research on obligate symbiosis |  |
| VI.a. Differences among specialized feeding guilds | **p.20** |
| VI.b. Non-ubiquity within host families | **p.21** |
| VII. Nutrient comparisons among plant tissues - STATISTICS |  |
| VII.a.i. Nitrogen | **pp.22-23** |
| VII.a.ii. B7-biotin | **p.23** |
| VII.a.iii. B9-folate | **pp.23-24** |
| VII.a.iv. B6 (pyridoxine) | **p.24** |
| VII.a.v. B5 (pantothenate) | **p.25** |
| VII.a.vi. B3 (niacin/nicotinate) | **pp.25-26** |
| VII.a.vii. B2 (riboflavin) | **p.26** |
| VII.a.viii. B1 (thiamine) | **pp.26-27** |
| VII.a.ix. Fiber | **pp.27-28** |
| VII.b. Examining plant taxonomy and plant tissue effects on  nutrient quantities |  |
| VII.b.i. Nitrogen in leaves, pollen, roots, wood across select  plant families | **p.28** |
| VII.b.ii. Nitrogen quantities in leaves, pollen, wood across  select plant families | **pp.28-29** |
| VII.b.iii. Fiber quantities in fruit, leaves for select plant  families | **p.29** |
| VII.c. Examining effects of plant taxonomy on B-vitamin  content of seeds | **pp.29-30** |
| VIII. Obligate symbionts vs. dietary nutrition - STATISTICS |  |
| VIII.a.i. B1 (thiamine) with Blood | **p.31** |
| VIII.a.ii. B1 (thiamine) without Blood | **p.32** |
| VIII.b.i. B2 (riboflavin) with Blood | **pp.32-33** |
| VIII.b.ii. B2 (riboflavin) without Blood | **p.33** |
| VIII.c.i. B3 (niacin/nicotinate) with Blood | **pp.33-34** |
| VIII.c.ii. B3 (niacin/nicotinate) without Blood | **pp.34-35** |
| VIII.d.i. B5 (pantothenate) with Blood | **p.35** |
| VIII.d.ii. B5 (pantothenate) without Blood | **pp.35-36** |
| VIII.e.i. B6 (pyridoxine) with Blood | **pp.36-37** |
| VIII.e.ii. B6 (pyridoxine) without Blood | **p.37** |
| VIII.f.i. B7 (biotin) with Blood | **pp.37-38** |
| VIII.f.ii. B7 (biotin) without Blood | **pp.38-39** |
| VIII.g.i. B9 (folate) with Blood | **p.39** |
| VIII.g.ii. B9 (folate) without Blood | **pp.39-40** |
| VIII.h. Fiber | **pp.40-41** |
| VIII.i. Nitrogen | **p.41** |
| IX. Symbiont metabolism/function vs. dietary nutrition - additional  results and discussion |  |
| IX.a. Nitrogen recycling/fixation results - metabolism | **pp.41-42** |
| IX.b. Nitrogen recycling/fixation results - function | **pp.42-43** |
| IX.c. Fiber breakdown results - metabolism | **pp.43-44** |
| IX.d. Fiber breakdown results - function | **p.44** |
| X. Amino acid content in varying insect foods - STATISTICS |  |
| X.a. Plant tissues only |  |
| X.a.i. Alanine | **p.45** |
| X.a.ii. Arginine | **pp.45-46** |
| X.a.iii. Asparagine | **p.46** |
| X.a.iv. Aspartate | **p.47** |
| X.a.v. Cysteine | **pp.47-48** |
| X.a.vi. Glutamine | **p.48** |
| X.a.vii. Glutamate | **pp.48-49** |
| X.a.viii. Glycine | **pp.49-50** |
| X.a.ix. Histidine | **pp.50-51** |
| X.a.x. Isoleucine | **p.51** |
| X.a.xi. Leucine | **p.52** |
| X.a.xii. Lysine | **pp.52-53** |
| X.a.xiii. Methionine | **pp.53-54** |
| X.a.xiv. Phenylalanine | **p.54** |
| X.a.xv. Proline | **p.55** |
| X.a.xvi. Serine | **pp.55-56** |
| X.a.xvii. Threonine | **pp.56-57** |
| X.a.xviii. Tryptophan | **p.57** |
| X.a.ix. Tyrosine | **pp.57-58** |
| X.a.xx. Valine | **pp.58-59** |
| X.b. All tissues/insect foods |  |
| X.b.i. Alanine | **pp.59-60** |
| X.b.ii. Arginine | **pp.60-61** |
| X.b.iii. Asparagine | **p.61** |
| X.b.iv. Aspartate | **pp.61-62** |
| X.b.v. Cysteine | **pp.62-63** |
| X.b.vi. Glutamine | **p.63** |
| X.b.vii. Glutamate | **pp.63-64** |
| X.b.viii. Glycine | **pp.64-65** |
| X.b.ix. Histidine | **pp.65-66** |
| X.b.x. Isoleucine | **pp.66-67** |
| X.b.xi. Leucine | **p.67** |
| X.b.xii. Lysine | **pp.67-68** |
| X.b.xiii. Methionine | **pp.68-69** |
| X.b.xiv. Phenylalanine | **pp.69-70** |
| X.b.xv. Proline | **pp.70-71** |
| X.b.xvi. Serine | **p.71** |
| X.b.xvii. Threonine | **pp.71-72** |
| X.b.xviii. Tryptophan | **pp.72-73** |
| X.b.ix. Tyrosine | **pp.73-74** |
| X.b.xx. Valine | **p.74** |

Supporting Information Discussion

| XI. Supplementary Discussion – An inordinate fondness for beetles? |  |
| --- | --- |
| XI.a. Diversity sustained by plausible symbiotic key innovations  in curculionids | **p.75** |
| XI.b. Diversity-sustaining symbiotic key innovations in other  beetles? | **pp.75-77** |
| XI.c. Diversity-sustaining nutritional services in small subsets of  the world’s palinivores and detritivores – outlines of  symbiont-based key innovations? | **pp.77-79** |

Supporting Information Comparisons vs. Cornwallis et al. 2023

| XII. Comparisons of study design and sample size vs. a recently publish paper with overlap. | **pp. 79-80** |
| --- | --- |

Supporting Information Bibliography

| XII. References Cited | **pp. 81-101** |
| --- | --- |

Supplementary Figures – please see .pdf and .pptx files

Supplementary Tables – please see Microsoft Excel files

**SUPPORTING INFORMATION METHODS**

**I. Objective 1 – insect diet vs. obligate symbiosis**

**I.a. Insect diversity counts**

Species counts for 874 families of insect, identified below, were retrieved from the GBIF database (https://www.gbif.org). The GBIF API was queried in August 2023 for each species using the species-specific GBIF usage key for each species. We limited counts of species to include only those with either an ‘accepted’ or ‘provisional’ status. Results were deposited into **Table S1** of the supplementary materials from our [Zenodo data repository submission](https://doi.org/10.5281/zenodo.19607748).

**I.b. Research on insect diets – goals and general approaches**

To better delineate insect diet, we reviewed the literature for the above-mentioned insect families (Class Insecta), which spanned 25 orders. Also targeted were three groups studied only at the order level (Archaeognatha, Strepsiptera, and Zoraptera), and 27 families from six orders of non-insect hexapods (Class Entognatha). This taxonomic list came from two prior publications (Rainford et al. 2014; Ranford & Mayhew 2015), but also included a subset of additional taxa identified in two further studies (Labandeira 2014; Cornwallis et al. 2023). The Rainford papers were dually important in their compilation of both dietary and phylogenetic framework for these insects. We used the dietary information from their 2015 study (https://doi.org/10.5061/dryad.6f75v) to begin populating a larger database, adding to it the additional insect families from Labandeira and Cornwallis. We return later to talk about the phylogeny from Rainford et al. 2014, which we use as a framework for understanding insect-diet-symbiont function associations.

Despite dietary classifications by Rainford, Labandeira, and Cornwallis, **we saw a benefit of generating separate diet estimations, and did so through an extensive literature search** (e.g. see **Box 1** in the main manuscript). Our motivations were two-fold. First, many of the prior dietary classifications did not parse out the types of plant tissues we focus on in this review. Having obtained nutrient data for such tissues, and knowing some insects consume them – sometimes exclusively – we aimed for a dataset meeting our objective of comparing symbiont function vs. dietary shortcomings. We identified a total of 20 food categories/feeding habits for our classification, including 14 from distinct plant tissues (**Figure 2**; **Table S1**). Second, prior taxonomically broad studies have sometimes minimized or not encountered evidence of rarer feeding habits when summarizing for their final analyses/data presentations. From our early explorations we saw that this missed out on some key opportunities. Toward this end, we coded food consumption across the 202 categories using an ordinal system, with“3” denoting our best estimate that most species within the family consume the item in question; “2” denoting that a modest fraction consume the given food (or that it makes up a modest percentage of omnivores’ diets); and “1” denoting that the food is eaten by members of the family to a much lower level (i.e. rare occurrence among dietary specialists, or a small fraction of omnivorous diets). While undoubtedly crude, this system provides a good starting snapshot into the diversity and prevalence of food consumption in many insect families.

Our general approach for identifying diet was to include insect family name (e.g. Apidae) in Google Scholar search queries, and then to search for terms including “feeding” and “diet”. When such searches provided minimal insight, we used common names (e.g. honeybee) or genus names (e.g. *Apis*) from these families alongside these aforementioned food terms. When this second strategy continued to yield little data we adopted a third approach, running Google Scholar queries with the diets suggested by websites including Wikipedia or BugGuide in addition to the above taxonomic or common name labels.

When possible, we focused on papers with broad taxonomic assessment of diet. For most families such papers were not discovered. In these cases we read papers spanning a diverse suite of subfamilies and genera when available for a family. Our goal was to always obtain multiple sources for each family, and to thus gain generalizable conclusions. This proved easier for some families than others. We cite all sources behind our decision making – both literature and web-/database-based – in **Table S1**.

In total, our dietary assessments were compiled from a review of 2383 sources covering our 874 insect families. All but 98 of these sources were published research articles and most were from the peer-reviewed, primary research literature. Several sources were cited more than once within our dataset, with the most commonly referenced being bugguide.net (comprising 75 of our 2604 citations), the website for the Center for Freshwater Ecosystems (https://www.mdfrc.org.au – 15 citations), and three websites with 10 citations each (https://www.macroinvertebrates.org/; https://www.ukbeetles.co.uk; and http://www.faculty.ucr.edu/~legneref/).

**I.c. Research on insect diets – caveats and special considerations**

We added asterisks next to ordinal scores in **Table S1** when feeding habits were somewhat uncertain or when there was nuance requiring explanation. Types of uncertainty often flagged in this manner included floral visitation by adult insects (as a basis for tentatively arguing for nectar feeding, especially when other authors had done so); uncertain claims made on websites but not found in the peer-reviewed literature; inferences on particular types of sap-feeding made for several hemipterans when no direct evidence was available (based on dietary conservation within the surrounding lineages); highly conserved habits of larval predation within the Odonata (dragonflies & damselflies) being applied to Odonata families without direct observation; assumed blood-feeding by various lice and adult fleas based on dietary conservation within the surrounding lineages); assumed non-blood-feeding by most flea larvae; rare claims in published papers that disagreed with patterns from other sources; and ‘singleton’ observations based on data from just one insect species within the family.

When we could find no evidence for the feeding habits of adults from a family, or when we found evidence that adults of a family do not feed, we left adult diet categories blank. When we found any designation of a food for an adult, we scored it as a 1-3. But the unique recurrence of non-feeding habits in adults (unlike larvae) renders some 1-3 scorings as misleading for this stage. For example, scoring of ‘3’ for nectar means that among those species with feeding adults (within the family), nectar was the predominant food source identified. It does not mean that most adults in the family feed on nectar. Since all juvenile insects must eat, the 1-3 scoring is more intuitive at this younger stage. And given the high importance of feeding at immature stages to support growth and development, we placed a high emphasis on identifying diet for this stage – i.e. when symbioses are often most important.

For several hemimetabolous insects (including orthopterans, phasmids, varying hemipterans, collembolans, etc.), we assumed diets were similar between mature (adults) and immature (nymphs/larvae) stages, unless otherwise stated. We avoided this assumption for hemimetabolous insects with drastic habitat shifts across development (e.g. aquatic juveniles vs. terrestrial adults, as in Ephemeroptera), leaving diet blank for a stage if not explicitly discovered. For holometabolous insects, we similarly never assumed that larval diets resemble those of adults.

A series of additional considerations and caveats are described as follows:

-For diets of rotting wood, it is often not clear how much of the digestate is comprised by the remaining plant tissue vs. microbial by-catch or even selectively consumed microbes. These diets were, thus, typically given designations of wood consumption and microbivory.

-Diets made up mostly of decaying non-woody plant tissue, across a range of decomposed states, were classified as detritivory. Claims of ‘detritus’ feeding were found in several sources that did not refer to dead plant remains, and these were classified based on further specifics in those descriptions (e.g. sometimes ‘detritus’ meant carrion, in which case this was categorized as predation/scavenging – i.e. animal flesh consumption; sometimes it meant bird feathers or skin, in which case the classification was ‘other’). Like decaying wood, non-wood plant tissue detritus is frequently comprised of high microbial loads, especially during later-stage decay. But given the uncertainty of most detrital classifications we specified microbial designations only when mentioned in our sources. Further classified as detritus in our scheme was dung, based on the reasoning that the most abundant sources of dung are from mammalian herbivores, and that some fraction consists of undigested plant matter. Dung certainly contains some microbial biomass, so once can see the difficulties of precise classification here.

-We differentiated leaf chewing habits from diets in which mostly mesophyll (leaf cell content) was consumed. The latter diet has a lower fraction of fiber, and more concentrated quantities of vitamins and nitrogen. For those insects feeding on cell content (generically called parenchyma) inside of galls, we also gave a mesophyll designation, for similar reasons. In cases where insects feed within galls on unknown food content, we left the feeding designation as simply ‘galls’. In addition to mesophyll feeders – including 1) some galling insects, 2) insects with piercing/sucking mouthparts that consume cell contents (e.g. thrips, some hemipterans), and 3) leaf-miners – we applied the mesophyll feeding designation to leaf skeletonizers, insects which minimize consumption of fibrous tissues when eating leaves.

-We assigned insects characterized as leaf and shoot chewers exclusively to the leaf/shoot-chewing category.

-For insects feeding inside of herbaceous stems, the tendencies to chew fibrous tissues or to selectively consume cell contents were rarely provided. We, thus, referred to these as stem borers.

-The phloem sap and xylem sap categories were assigned if the insects were known to consume sap from these vascular elements as their primary food source. Insects feeding on plant wound exudates, or sap flows, or unidentified root exudates, etc. were not assigned to either of these categories. Insects with these feeding habits were given ‘other’ designations, though sap flow feeders in some cases earned classifications as microbivores when described as consuming the fermenting microbial biomass within this resource, and since several such insects have specialized means for ingesting sap-dwelling microbes.

-We did not group bryophyte- or moss-feeding insects with those consuming leaves or stems, having less available data for the nutritive value of these tissues. Instead, those feeding on these non-vascular plants were placed in the ‘other’ category. We similarly did not classify piercing and sucking ‘moss bugs’ (order Coleorhyncha) as mesophyll feeders or as phloem/xylem sap feeders due, in the latter case, to the non-vascular status of moss.

-We treat blood separately from the animal tissue consumption category, although animal tissue consumption includes some amount of blood in many cases.

**I.d. Research on obligate symbioses**

Using similar Google Scholar queries to those used for diet, we investigated the incidence of obligate symbiosis across insect families. We first searched for family names alongside the terms “symbiont” and “symbiosis”. For families related to symbiont-reliant insect clades, we followed initially unsuccessful searches with follow-up queries using family names alongside “bacteria”, “fungi”, “protist”, or the genus names of symbionts found in their relatives (e.g. “Sulcia” when examining families within the Auchennorhyncha). When needing further data, we queried the NCBI database with these same insect family and symbiont genus names, searching in these cases for 16S rRNA sequences supporting the presence of a conserved bacterial symbiont, found in relatives.

Ideal criteria for assessing obligacy should center around the magnitude of fitness impacts (e.g. Fisher et al. 2017). In this vein, few would argue against the ‘obligate’ label for symbionts that a host cannot live or reproduce without. But one might further extend the definition to those providing large boosts to the survival, growth, or reproduction of hosts. Under this scenario, insects need not have fitness = 0 upon symbiont removal in the lab. Fitness loss must, instead, approach a magnitude implying likely inviability under real-world conditions.

Difficulties arise, however, in knowing whether obligacy stems from evolutionary addiction or from indispensable and novel symbiont services (Hammer 2024). Given this, and the lack of fitness data in many host taxa, we used other traits more commonly in our classification (**Box 2**). In doing so, upon review of the returned literature, we scored each insect family as either harboring an obligate symbiont (“1”) or lacking evidence (“0”) for obligate symbiosis (Tables S3-S4 – [Zenodo](https://doi.org/10.5281/zenodo.19607748) repository). Gray-area examples include the bean bug-*Caballeronia* symbiosis, treated as obligate here because of high host integration and symbiont conservation (despite a complete loss of fitness upon symbiont removal).

**II. Objective 2 – part 1 – plant nutrition**

**II.a. Plant nutrition search strategy and data extraction – general overview**

We performed a comprehensive search for relevant peer-reviewed publications on plant tissue nutritive data between February 2021 through August 2022. At the onset of our literature review, there were few published syntheses on dietary nutrients spanning: 1) multiple plant tissues; 2) multiple plant taxa; and 3) multiple nutrients that are often limiting for insects. For comparative purposes, we also mined the literature and online databases for nutrient data from other diets, including tissues of non-vascular plants, algae, lichen, fungi, protists, prokaryotes, decaying plant matter (i.e. detritus), vertebrate blood, and other animal-based tissue diets.

To identify relevant datapoints, we performed keyword searches against the Web of Science database, and a series of Google Scholar queries that included forward-backward citation searches of identified papers. We also sought out data within the FoodData Central database of the United States Department of Agriculture (https://fdc.nal.usda.gov/index.html), and from two livestock feed databases (https://www.feedipedia.org/; https://www.voederbomen.nl). We initially recorded data on a broad range of nutrients, but narrowed our dataset down to include only % dry weight measures (or conversions) for total Nitrogen (N), fiber (neutral detergent fiber = cellulose + hemicelluloses + lignin), or B-vitamins (all but B12). Our rationale for this stemmed from knowledge that symbionts shape the metabolism of each such nutrient class across several insect hosts (Douglas 2021). Symbionts also synthesize and provision amino acids. We used nitrogen as a proxy for amino acids here, discussing dietary amino acid data in later sections.

Searches for plant tissue nutritive data were initiated using keyword queries in the Web of Science database. Additional Google and Google Scholar searches were implemented when the original searches returned few relevant studies. Due to their rich abundance of data, we obtained several data measures from the livestock fodder databases Feedipedia (<https://www.feedipedia.org/>) and Voederbomen.nl (<https://www.voederbomen.nl/>). And for the same reason, we used USDA’s FoodCentral database (https://fdc.nal.usda.gov/), primarily as a source for B-vitamins and amino acids.

For keywords, we specifically targeted plant tissues known to be the main staples in herbivorous insects’ diets including food bodies, fruit, gall tissue, leaves, nectar, seeds, pollen, phloem sap, xylem sap, roots, and wood (including sapwood, heartwood, and bark). While we initially sought out a broad range of nutrient data, we experienced difficulty in knowing whether some measures were uniform across studies (e.g. total sugars, total carbohydrates). Due to this and to known involvement of insect symbionts in nitrogen, fiber, and B-vitamin metabolism, we prioritized total N, neutral detergent fiber (or the sum of cellulose, hemicelluloses, and lignin), and all B-vitamins but cobalamin (B12), using these phrases, or more specific phrasing (e.g. full names for B-vitamins), as the remaining portions of our tissue/nutrient-specific keyword searches.

We prioritized returned articles for close reading after abstract review. Following review of the main text, we identified articles for data extraction. For some tissue-nutrient combinations we found more relevant papers than we were able to process, given imposed time limits for each tissue and nutrient. In such cases, screening was considered sufficient once the relevant nutrient data were collected from a large taxonomic diversity of plants. Helping to further narrow these large publication lists, we gave top priority to studies with titles or abstracts suggesting nutrient quantification in wild and unmanipulated plants (e.g. unfertilized, un-attacked, un-stressed). Despite this emphasis, a good portion of datapoints came from plants in an agricultural or other human-impacted setting.

Data were recorded for the aforementioned nutrients in a large spreadsheet. When not available in the text or associated tables we used WebPlotDigitizer (https://automeris.io/WebPlotDigitizer/) to extract quantitative values from graphs. In some cases we entered single averages for each tissue, nutrient, and plant species when replicates were taken within a single study. For others, we included each raw measure, using these to later compute averages for our statistical analyses.

To enable comparability across measures, we settled on % dry weight as our focal unit. Defined as the percentage of overall dry weight made up by the nutrient in question, this focus overcomes variation in nutrients created due to varying states of humidity or tissue dehydration. When not obtained directly, conversions to % dry weight were performed for measures obtained as % fresh weight or concentrations when % water was also measured. Data conversions proceeded according to the formulae in **Figure A.**

**Figure A**: Equations used to convert extracted nutrient data to a common set of units – i.e. % dry weight.. Not shown was the following common conversion: % dry weight = 100*(% nutrient fresh weight/(100% – % water weight)). MW = molecular weight.

We applied a further conversion to obtain %nitrogen from %protein when it was clear that protein quantities were obtained from an initial measure of nitrogen, as was often the case. One indication that this derivation had been applied was a reference to the micro-Kjeldahl method as the basis for the protein measure. Since this method quantifies nitrogen, and since protein concentrations are almost always then obtained by multiplying %N by 6.25, we divided most such protein measures by 6.25 to get % nitrogen. In a few cases, individual studies had multiplied by a different conversion factor. We used these unique quotients, in place of 6.25, in such instances.

**II.b. Plant nutrition search strategy and data extraction – assumptions made for wood**

For wood, all B-vitamin data were derived from Zimmerman 1964 (https://doi.org/10.1016/C2013-0-12178-2), which reported values for only biotin (B7) and pyridoxine (B6). Since values provided in this resource consisted of measures across growth rings for two of the three studied tree species (i.e. *Pinus strobus* and *Robinia pseudoacacia*, but not *Tilia vulgaris*), we took the highest B-vitamin quantities across the summed ‘free’ and ‘bound’ fractions to give high end estimates of biotin and pyridoxine in wood. For the third species – which lacked growth ring sampling – we combined estimates of biotin from stem wood, root wood, and bark wood to produce a single estimate used in later graphing. Data and calculations used here can be found in Table S22 from our [Zenodo](https://doi.org/10.5281/zenodo.19607748) data repository submission.

**II.c-e. Plant nutrition data assumptions for wood, phloem sap, xylem sap, nectar, and blood.**

Researchers rarely estimated nutrient content of phloem sap, xylem sap, nectar, or blood in % dry weight. To achieve this, we found studies estimating typical water content for each liquid food. We did the same for estimates of density. With those metrics in hand, we converted nutrient measures from these liquids into our standard % dry weight measures as described in Tables S23-S26 from our [Zenodo](https://doi.org/10.5281/zenodo.19607748) submission.

**II.c. Plant nutrition search strategy and data extraction – % dry weight estimates for phloem**

For phloem sap we found little-to-no %dry weight unit data for %nitrogen and B-vitamins. Measures were, instead, often in the form of weight by volume. But the lack of % water weight and density estimates for the measured sap hindered our conversions to % dry weight. To remedy this, we examined estimates of phloem sap water weight from a small range of separate studies. The first (Lintunen et al. 2016) involved numerous measurements of phloem sap from four different species: *Betula pendula* (birch), *Picea abies* (spruce), *Pinus sylvestris* (Scotch pine), and *Populus tremula* (aspen). Very few datapoints fell below the estimate of 0.5g water/g dry mass material within phloem sap, suggesting 33.3% water weight as a low-end estimate. The center of the data distribution approached 1.25g water/g dry mass, suggesting 55.6% as an intermediate value. We further observed just a modest number of data points (all from *P. silvestris*) beyond 2.0g water/g dry mass, hence, suggesting 66.7% water weight as an upper estimate.

In a separate study (Gall et al. 2002), the authors suggested water weight content of phloem sap ranged from 1.4-2.6 mg water/mg dry mass for *Picea abies* (a.k.a. Norway spruce), hence spanning 58.3-72.2% of the total mass.

In a further study across 55 plant species, Jensen and colleagues (2013) estimated that the fractions of phloem sap weight comprised by solutes ranged from range of 5-59%. They argued that a typical measure was 20% solute concentration, with the rest implied to be water.

Given the more comprehensive nature of the Jensen study, we used their 80% water and 20% solute weight values as reasonable point estimates for phloem. Using this same study, we obtained a density estimate of 1.079 g/ml for phloem sap.

Based on these conversion factors, we divided estimates of total nitrogen, amino acids, or B-vitamins expressed in weight per volume (g/100 ml) by the density estimate of 1.079 g/ml. This produced values with units of g/100g. We then divided by 20 (20 g dry solute/100 g fresh weight) before multiplying by 100 to get % dry weight. Clearly these are imperfect generalizations, and should, thus, be adopted with caution.

**II.d. Plant nutrition search strategy and data extraction – assumptions made for nectar and xylem**

We used a similar approach to that used above for phloem to convert some fresh weight or concentration-based nutrient data from xylem sap and nectar into % dry weight units. For the former, several studies have indicated that the highly dilute nature of xylem sap puts its density close to 1 g/ml (Paljakka et al. 2020). Using data from another study (Gollan et al. 1992) we estimated that xylem sap is comprised of 97% water and 3% solute. As per phloem, then, we hence divided weight by volume (g/100 ml) estimates by the 1 g/ml density value, subsequently dividing this by 3, and multiplying by 100 to get % dry weight.

For nectar, we used data from a study of 15 pollinator-visited plant species (Chalcoff et al. 2006). This suggested an average nectar density of 1.14 g/ml and that the % water weight of floral nectar is 68.3, on average. We converted data to % dry weight by dividing fresh weight measures of nutrients by 1.14, then dividing the quotient by the solute % (31.7), before finally multiplying by 100.

**II.e. Nutrition search strategy and data extraction – assumptions made for blood**

To compare nutrition of vascular plant-based diets to that of other insect food sources, we sought out measures for B-vitamins, amino acids, and nitrogen from blood, animal tissue, fungi, lichen, algae, and prokaryotes. With blood being a liquid diet, published nutrient values were rarely expressed in terms of % dry weight. We adopted the approach used above for phloem, xylem, and nectar to convert weight/volume measures into our common (% dry weight) unit currency.

First, 55% of human blood is made up of plasma, which has a water content of 92% (e.g. varying web sources, including Wikipedia). Most of the remainder of human blood (just under 45%) is comprised of erythrocytes. We computed the average % water content for erythrocytes from Table V of Beilin et al. 1966, with this value coming out to be 65.42%. By multiplying 0.55 * 92% and 0.45 * 65.42%, and adding these products, we obtained a human blood water content of 70.84%, a value we apply to blood in general. We note separately that in a 2015 article, Vitello and colleagues estimated that the density of whole human blood to be 0.994 g/ml.

As such, we divided estimates of total nutrients (B-vitamins, amino acids, nitrogen) expressed in weight per volume (g/100 ml) by 0.994 to obtain measures with units of g/100g. We then divided by the % solute concentration of 29.16 (100-70.84), before multiplying by 100 to get % dry weight.

**II.f. Plant nutrition search strategy and data extraction – QC assessment**

We inspected initial graphs of our data, identifying outliers and double-checking that the data had been accurately extracted, converted, and recorded. In doing so, we found a series of B-vitamin entries from leaves and galls that were 10-100 fold greater than nearly all other data points for the same vitamins in our dataset, including those from leaf tissue within the USDA database. All came from a single, 44-year-old study that had just one citation (Rao & Mallikarjuna 1979). Suspecting these estimates to be mistaken, we removed these data from our study.

**II.g. Plant nutrition statistics**

We compared nutrient quantities (Tables S8-S16 – [Zenodo](https://doi.org/10.5281/zenodo.19607748) repository) among tissues using linear mixed models in the R-statistical package (2021). Tissue was included as a fixed effect, while plant family was modeled as a random effect. Pairwise, post-hoc comparisons were conducted using the emmeans function. Values used for statistics were species-level averages for each nutrient obtained from a given plant tissue.

To complement these analyses, we considered whether plant taxonomy has a larger effect on nutrient quantity than does plant tissue. Toward this end, we performed a small number of analyses with a more balanced design. For these we included each species-level average for a nutrient only when there was a modest sample size (n ≥ 2 species) for the given tissue within a plant family. We modeled plant family, tissue, and their interaction terms as fixed effects, using a linear regression approach due to the absence of a random factor. Due to limited within-family sampling for many nutrients, these analyses focused only on nitrogen and fiber. We focused our statistics on cases where the nutrient in question had factorial representation (across all families of relevance) across two or more tissues. This enabled the following comparisons: 1) nitrogen across leaves, pollen, roots, and wood for the Fabaceae vs. Pinaceae; 2) nitrogen across leaves, pollen, and wood for the Fabaceae vs. Pinaceae vs. Fagaceae vs. Rosaceae vs. Salicaceae vs. Betulaceae vs. Myrtaceae; and 3) fiber across fruit and leaves for the Moraceae vs. Rubiceae vs. Rosaceae vs. Euphorbiaceae vs. Salicaceae vs. Phyllanthaceae.

Being inspired by arguments from Salem et al. 2013, that B-vitamin titers of Malvales seeds could explain reliance on symbionts in cotton stainers (Pyrrhocoridae), we looked further at effects of plant taxonomy within seed diets. We identified all plant families with data for any one B-vitamin across n≥5 species. We then performed pairwise t-tests to assess differences in each such B-vitamin with two or more taxa meeting these criteria. P-values were corrected using the Benjamini-Hochberg and Bonferroni methods.

**III. Objective 2 – part 2 – symbiont vs. nutrient statistics**

We implemented generalized linear models in R to ascertain whether nutritive quantities in primary diets of an insect family (Table S21 – [Zenodo](https://doi.org/10.5281/zenodo.19607748) repository) predicted propensities to harbor obligate symbionts (Table S3 **–** [Zenodo](https://doi.org/10.5281/zenodo.19607748) repository). Response variables were binary (‘1’ = family had obligate symbiont; ‘0’ = family had no known obligate symbiont). Among our X-variables (% dry weight of fiber, nitrogen, or B-vitamins in the predominant food of the given insect families), we log-transformed B-vitamin data due to strong skew. Analyses for all B-vitamins were performed with and without data for blood feeding, helping to assess pattern robustness.

To obtain single point estimates of nutrients, and to synch diet categories with those used for assessing symbiont prevalence, we proceeded as follows:

First, when extracting values for nitrogen, we used the midpoint values of ranges presented in Table S21 ([Zenodo repository](https://doi.org/10.5281/zenodo.19607748)) – sometimes as our final values for analysis and sometimes for use in computing averages used in analysis. Second, the food category “roots” combined data categorized as roots (more traditional fibrous and root tissue) roots_rhizome, and roots_storage (i.e. tubers), through averaging when multiple such sub-tissues had extracted data. Third, for the category “animal tissue” we took the average of crustacean, vertebrate tissue, insect tissue, and mollusk tissue B-vitamin values when available. With a more limited range of animal tissues censused for our summaries of nitrogen, we used the midpoint value of vertebrate tissue for this analysis. Fourth, for the category “microbes” we used the average of amoebozoan slime molds, fungi, fungi_yeast, Archaea, protists, lichen, and (Eu)bacteria. Fifth, the category “algae” combined data from diatoms, microalgae and macroalgae. A sixth point worth consideration is that our category “fruit” excluded unripened fruit.

**IV. Objective 2 – part 3 – symbiont metabolism & function**

Prior *in vivo* studies have linked genetically encoded symbiont metabolism with emergent changes to insect physiology, morphology, behavior, and fitness (e.g. Ayayee et al. 2014; Nikoh et al. 2014; Snyder & Rio 2015; Anbutsu et al. 2017; Consuegra et al. 2020a;b; Duplais et al. 2021; Wang et al. 2020; Scharf & Peterson 2021). Drawing from this functional work, we focused on symbiont metabolism across 25 pathways/processes spanning the realms of fiber degradation (pectin, lignin, cellulose, and hemicellulose), chorismate and tyrosine biosynthesis, essential amino acid biosynthesis, B-vitamin biosynthesis, urease-based nitrogen recycling, and nitrogen fixation. Our research on the *in vivo* literature centered around this metabolism as well.

**IV.a. Symbiont function – *in vivo* study**

Through an extensive literature search we summarized *in vivo* studies of B-vitamins, nitrogen, chorismate/amino acid, and fiber metabolism. After initial discoveries with Google Scholar-based literature searches – where we found novel papers through use of search terms including “insect”, “(endo)symbiont”, “digestion”, “nitrogen”, “carbon”, “amino acid”, “fiber” “vitamin”, etc. – we used the identified articles and those found through forward-backward citation searches for abstract and methodology screening.

Recognizing that insects acquire ephemeral environmental bacteria, and that many specialized symbionts are hard to cultivate, we ignored most studies whose abstracts suggested a focus on cultured microbes. Exceptions were granted if they were clearly part of a specialized symbiosis. Our broader set of notes is contained within Tables S61-S65 ([Zenodo](https://doi.org/10.5281/zenodo.19607748) repository), while summaries at the host species level – for all deemed to have some *in vivo* support for symbiont function – are in Tables S66-S70 ([Zenodo](https://doi.org/10.5281/zenodo.19607748) repository). The summarized data from this narrowed down dataset came from 277 total papers. Most contained evidence from *in vivo* study. But a subset exclusively contained genomic or in vitro datasets when these supported in vivo research from the same insect hosts.

**IV.b. Symbiont metabolism – genomic study**

Expansion of genome sequencing across obligate symbionts of insects presented opportunities to infer likely symbiont function through their encoded metabolism. We achieved this with a careful mapping of proteins encoded by each obligate microbiome against KEGG-defined metabolic steps and pathway.

We used the KEGG database to understand the diversity of enzymes fulfilling our focal metabolism, and the diversity of sub-paths across each pathway. We assessed start- and end-points for multi-step pathways based on the literature and occasional consultation of the MetaCyc database (**Figure S9**; Table S32 - [Zenodo](https://doi.org/10.5281/zenodo.19607748) repository). Pathway topology and the diversity of associated KOs were largely determined on the KEGG website, but additional information was obtained for B-vitamin synthesis from the MetaCyc website, from a B-vitamin focused paper by Serbus et al. (2017), and by additional findings in the literature.

Through analysis of all EC (Enzyme Commission) numbers linked to each KEGG-defined reaction in our pathways, we obtained a list of 400 unique orthologous proteins (KOs) shaping the above processes.

We next explored microbial (meta)genomes in the NCBI and IMG databases, searching for insect hosts for which all known obligate symbionts had available sequences. For each identified NCBI dataset we downloaded the full set of encoded proteins. We searched for the 400 relevant KOs across BLAST-KOALA-generated KO lists for each (meta)genome, removing all instances of KOs encoded by a pseudogene (Table S33 **–** [Zenodo](https://doi.org/10.5281/zenodo.19607748) repository). For IMG-derived (meta)genomes, we identified all encoded KOs using the Abundance Profile tool. IMG results were then exported in matrix format, allowing similar searches for the 400 KOs of interest (Table S34; see also Table S35 - [Zenodo](https://doi.org/10.5281/zenodo.19607748) repository).

Proteins were not available for the genome of *Stammera capleta* from *Cassida rubiginosa*. We could thus not use BLAST-KOALA for assessing this genome. Due to the absence of KO identifiers in this genome’s annotation, we instead cross-referenced all EC numbers assigned to each of encoded protein against the list of proteins encoding steps of the varying biosynthetic/reduction/catabolic pathways alluded to above.

With indications of presence/absence (or partial completeness) for each KO-linked step in our focal metabolic pathways, we proceeded to quantify unique KO counts (nitrogen recycling, nitrogen fixation, fiber breakdown), and pathway completeness (chorismate, tyrosine, essential amino acid, and B-vitamin pathways) across full microbiomes. Some steps were encoded by a number of different individual KOs. When individual steps required multiple KOs – often due to multi-subfactor units of a holoenzyme – we coded steps as absent (0), complete (1), or as fractions (e.g. 1/2 present required KOs = 0.5) depending on the required KO count and total number of required KOs.

For nitrogen fixation, nitrogen recycling, and fiber catabolism we represented findings as counts of unique KOs encoded. When distinct KOs encoded the same step in an amino acid or B-vitamin pathway, we did not double count, due to our focus on a metric of completeness for these pathways/functions. The presence of several genes in a pathway is likely to reflect either autonomous or collaborative biosynthetic abilities. Due to the potential for collaboration or to co-option of novel or promiscuous enzymes not yet recognized to have involvement, we would argue that this continuous – ‘completeness’ – metric holds value beyond that of a binary ‘complete’ vs. ‘incomplete’ classification.

To calculate pathway completeness, we divided the number of steps encoded by each obligate symbiont, and – separately – each multi-symbiont obligate microbiome by the total number of required steps for that pathway. Some biosynthetic pathways were comprised of separate sub-pathways that could be utilized in making a nutrient. In these cases, we considered each of the possible direct routes to go from the starting substrate(s) to the end product nutrient. Counting up the numbers of complete (or partially complete) steps as described above, we estimated which of the sub-pathways had the greatest completeness, using this maximum value for our analyses. In our vitamin analyses, we also considered steps that are vs. are not encoded by some insect hosts, to determine if symbionts might specialize on metabolisms hosts cannot encode.

We used the feeding habits for the hosts of these symbionts (often inferred at the host family, but sometimes from direct reporting on species) to obtain dietary estimates of nutrient content for our analyses (e.g. see Table S21 – [Zenodo](https://doi.org/10.5281/zenodo.19607748) repository) and section **III. Symbiont vs. nutrient statistics**, above, where we discuss how we computed point estimates for nutritive values). Using these point estimates for B-vitamin, nitrogen, and fiber content, we explored the relationship between symbiont metabolism vs. dietary nutrition with a series of linear regressions (Tables S73-74, S78-79 – [Zenodo](https://doi.org/10.5281/zenodo.19607748) repository). For B-vitamins, we followed these up with t-tests (Table S75 – [Zenodo](https://doi.org/10.5281/zenodo.19607748) repository), with a binary x-variable representing whether the host’s diet fell above or below the required B-vitamin dietary threshold (Dadd 1985), and continuous y-variables based on symbiont metabolic results (pathway completeness or unique KO counts).

In these analyses we varied inclusion of full datasets – i.e. pathway completeness/unique KO counts for all obligate microbiomes – or whether our analyses focused only on specialized, ancient obligate symbionts sampled with isolate genome analysis. Such latter analyses excluded free-living bacteria (sometimes found in metagenomes, and also found among a set of insects relying on environmentally acquired microbes). And by excluding all obligate symbionts with 16S rRNA sequences >95% 16S rRNA identity to that of a free-living microbe, we excluded those likely still in the process of losing genes less integral to the symbiosis. These smaller analyses captured the results of ancient reciprocal specialization, where gene retention is a clear indication of symbiont need and a more likely indicator of host-level metabolic benefit.

**SUPPORTING INFORMATION RESULTS**

**V. Limits to our insect diet assessment**

Our calculations of feeding habit/food source prevalence exhibit potential bias. This is partially because our data were not quantitative. For example, when families used more than one diet we could not precisely calculate the numbers of monophagous species using each food source or the relative use of multiple foods, in more polyphagous families. Related challenges arise from ‘artifacts’ of our 1-3 coding system. For example, when estimating which families were predominantly herbivorous, juveniles of the Arctiidae moth family fell within the non-herbivorous category due to assessments of moderately common lichen and free-living algae feeding (dietary scores = 2 + 2 for moderate feeding on each). Despite the higher sum for non-herbivorous foods (microbes + algae) than for their single, primary plant-based diet of leaf-chewing (i.e. 2+2 > 3), most would recognize this family to be predominantly herbivorous. Another counter-intuitive call involved the highly diverse Carabidae. Immatures from many species in this beetle family are likely predatory. But our literature-based assignment of ‘moderate’ predation during the larval stage did not outweigh the summed scores from somewhat common seed feeding and rare fruit feeding (i.e. 2 < 1+2). A third caveat for our heuristic placement of families into predominant herbivory vs. non-herbivory bins was the discrepancy between the numbers of categories for each habit: 6 under non-herbivory vs. 14 for herbivory. Since there is a higher potential sum in the herbivorous category, and since some insect herbivore families eat more than one plant tissue, our finer-grained classifications of plant-feeding have possibly inflated the estimated number of predominantly herbivorous families.

**VI. Research on obligate symbiosis –**

**VI.a. Differences among specialized feeding guilds**

Counts of families with vs. without an obligate symbiont among those with a primary diet. Shown here are results for such feeding guilds with n≥5 families.

| Primary diet | Symbiont negative families | Symbiont positive families | Proportion families with an obligate symbiont |
| --- | --- | --- | --- |
| phloem | 2 | 33 | 0.943 |
| xylem | 1 | 6 | 0.857 |
| fruit | 2 | 4 | 0.667 |
| seeds | 7 | 6 | 0.462 |
| wood | 12 | 6 | 0.333 |
| pollen | 8 | 1 | 0.111 |
| mesophyll | 29 | 2 | 0.065 |
| leaf/shoot chewing | 71 | 1 | 0.014 |
| blood | 2 | 13 | 0.867 |
| microbes | 50 | 7 | 0.123 |
| detritus | 67 | 2 | 0.029 |
| animal flesh | 238 | 5 | 0.021 |
| algae | 23 | 0 | 0.000 |

P-values from Fisher’s Exact tests, making pairwise comparisons on the incidence of obligate symbionts in families with a primary diet. Shown here are results for such feeding guilds with n≥5 families. P-values in bold were still significant after a Benjamini-Hochberg correction (with a 0.01 False Discovery Rate). P-values ≤0.05 that are underlined were not significant after this correction.

**VI.b. Non-ubiquity within host families**

It is important to note that obligate symbionts were not ubiquitous within some hosting insect families. Within the hyper-diverse Chrysomelidae beetles, for instance, are several lineages that lack the separately derived symbionts from the Cassidinae, Eumolpinae, Spilopyrinae, Sagrinae, and Donaciinae subfamilies (Reis et al. 2020; Brunetti et al. 2021; Ma et al. 2021; Fukumori et al. 2022; An et al. 2023; García-Lozano et al. 2024; Kirsch et al. 2025). With no described alternatives, many chrysomelids may be symbiont non-dependent.

Similar cases of non-ubiquitous obligacy are evident within the diverse Formicidae (ants), Tephritidae, and Apidae (bees) families (Mazzon et al. 2008; Kwong et al. 2017; Russell et al. 2017; De Cock et al 2020; Mason et al. 2023). They are also found in the Geocoridae and Pentatomidae of the Pentatomomorpha (true bugs) (Gordon et al. 2016), as well as the highly diverse Curculionidae (weevils). Obligate symbioses across this latter taxon range from those with widespread, bacteriocyte-dwelling bacteria (Lefèvre et al. 2004; Conord et al. 2008; Hosokawa & Fukatsu 2010; Huang et al. 2016; Morera-Margarit et al. 2019), to the mycangium-harbored, fungal symbioses of bark and ambrosia beetles (Ayres et al. 2000; Kostovcik et al. 2015; Vanderpool et al. 2018; Hulcr et al. 2020). The apparent gains and/or losses of these symbioses (Lefèvre et al. 2004; Six 2012; Toju et al. 2013; White et al. 2015; Zhang et al. 2017) suggest that symbiont-dependency has occasionally waxed and waned within the Curculionidae. With their high diversity, habitat and diet variability, weevils are promising candidates in the broader quest to understand ecological drivers of obligate symbiosis. Systematic screening for each type of symbiont, further research on extracellular gut microbes (Shelef et al. 2013; Jing et al. 2020), and further functional studies will be central in this endeavor.

**VII. *Nutrient comparisons among plant tissues***

**VII.a.**

We examined differences in the % dry weight quantities for specific nutrients among plant tissues using linear mixed-effects models. In these analyses, plant tissue was modeled as a fixed effect and plant family was modeled as a random effect. We used post hoc tests, with the emmeans function, for pairwise comparisons among individual tissues.

Before launching into these results, we first note that our gathered data represented an expansion of the work by Cornwallis et al. 2023. First, in comparison to their % protein measures (n=138 datapoints), our dataset of n=1183 species*tissue measures for the analogous %nitrogen was a large expansion. Our B1, B2, B3, B5, B6, B7, and B9 datapoints totaled n=1418 unique species * tissue measures, compared to n=600 for Cornwallis et al. (section XII for graphical comparison).

**VII.a.i. Nitrogen**

Model: lmer(Overall_N_Perc~Tissue+(1|Faimily),data=nitrogen)

Note: We removed data for any tissue with n<3 species sampled for nitrogen (i.e. corresponding to “ROOTS_RHIZOME”, “ROOTS_STORAGE”, “PTERIDOPHYTA_POLLEN”, “NECTAR”).

Table a. ANOVA summary of linear mixed-effects model examining the effects of plant tissues on overall nitrogen percentage.

|  | Sum Sq | Mean Sq | NumDF | DenDF | F value | Pr(>F) |
| --- | --- | --- | --- | --- | --- | --- |
| Tissue | 1950 | 139.28 | 14 | 1183 | 132.76 | < 2.2e-16 |

Table b. Pairwise comparison evaluated with the emmeans function. Letters in ‘group’ column denote significantly different tissues.

| Tissue | emmean | SE | df | lower.CL | upper.CL | .group |
| --- | --- | --- | --- | --- | --- | --- |
| XYLEM | 0.357 | 0.4088 | 1227 | -0.4452 | 1.159 | AB |
| WOOD | 0.365 | 0.0946 | 291 | 0.1786 | 0.551 | A |
| SEEDS_shell/coat | 0.752 | 0.6053 | 1156 | -0.4352 | 1.94 | ABCD |
| PHLOEM | 1.045 | 0.5273 | 1172 | 0.0104 | 2.08 | ABCD |
| GALLS | 1.176 | 0.6074 | 1152 | -0.0153 | 2.368 | ABCDE |
| FRUIT | 1.384 | 0.1122 | 495 | 1.1634 | 1.604 | BC |
| ROOTS | 1.495 | 0.1558 | 895 | 1.1893 | 1.801 | BC |
| STEMS | 1.622 | 0.1898 | 1110 | 1.2498 | 1.995 | BC |
| SEEDS_pod | 1.837 | 0.6048 | 1146 | 0.6504 | 3.024 | ABCDEF |
| UNRIPE_FRUIT | 2.203 | 0.3636 | 1226 | 1.4891 | 2.916 | CDE |
| LEAVES | 2.668 | 0.0866 | 210 | 2.4973 | 2.839 | DE |
| FLOWERS | 2.706 | 0.2459 | 1216 | 2.2237 | 3.189 | DEF |
| SEEDS | 3.557 | 0.1552 | 938 | 3.2521 | 3.861 | F |
| FOOD_BODIES | 3.627 | 0.4362 | 1226 | 2.771 | 4.482 | EFG |
| POLLEN | 4.532 | 0.1045 | 368 | 4.3265 | 4.737 | G |

**VII.a.ii. B7-biotin**

Model: lmer(Overall_B7_Perc~Tissue+(1|Faimily),data=B7)

Note: We removed data for any tissue with n<3 species sampled for biotin

Table c. ANOVA summary of linear mixed-effects model examining the effects of plant tissues on overall B7 percentage.

|  | Sum Sq | Mean Sq | NumDF | DenDF | F value | Pr(>F) |
| --- | --- | --- | --- | --- | --- | --- |
| Tissue | 2.18e-08 | 2.72e-09 | 8 | 641.62 | 9.6638 | 1.063e-12 |

Table d. Pairwise comparison evaluated with the emmeans function. Letters in ‘group’ column denote significantly different tissues.

| Tissue | emmean | SE | df | lower.CL | upper.CL | .group |
| --- | --- | --- | --- | --- | --- | --- |
| PHLOEM-SAP | 2.55E-07 | 4.34E-06 | 22.1 | -8.75E-06 | 9.26E-06 | A |
| WOOD | 3.40E-07 | 1.00E-05 | 75.7 | -1.96E-05 | 2.03E-05 | AB |
| FRUIT | 1.13E-05 | 4.30E-06 | 53.5 | 2.66E-06 | 1.99E-05 | AB |
| ROOTS_RHIZOME | 1.46E-05 | 9.89E-06 | 75.7 | -5.09E-06 | 3.43E-05 | ABC |
| SEEDS | 2.45E-05 | 4.38E-06 | 16.9 | 1.53E-05 | 3.38E-05 | BC |
| ROOTS_STORAGE | 2.48E-05 | 6.63E-06 | 52.3 | 1.15E-05 | 3.81E-05 | ABC |
| LEAVES | 3.73E-05 | 4.72E-06 | 65.8 | 2.78E-05 | 4.67E-05 | CD |
| STEMS | 3.84E-05 | 9.88E-06 | 75.7 | 1.87E-05 | 5.81E-05 | BCD |
| POLLEN | 6.24E-05 | 8.72E-06 | 64.6 | 4.50E-05 | 7.98E-05 | D |

__

**VII.a.iii. B9-folate**

Model: lmer(Overall_B9_Perc~Tissue+(1|Faimily),data=B9)

Note: We removed data for any tissue with n<3 species sampled for vitamin B9 (i.e. corresponding to “ROOTS”).

Table e. ANOVA summary of linear mixed-effects model examining the effects of plant tissues on overall B9 percentage.

|  | Sum Sq | Mean Sq | NumDF | DenDF | F value | Pr(>F) |
| --- | --- | --- | --- | --- | --- | --- |
| Tissue | 8.88e-06 | 1.48e-06 | 6 | 151.75 | 8.3962 | 7.197e-08 |

Table f. Pairwise comparison evaluated with the emmeans function. Letters in ‘group’ column denote significantly different tissues.

| Tissue | emmean | SE | df | lower.CL | upper.CL | .group |
| --- | --- | --- | --- | --- | --- | --- |
| FRUIT | 0.000162 | 6.35E-05 | 53 | 3.47E-05 | 0.000289 | A |
| SEEDS | 0.000162 | 7.34E-05 | 56.9 | 1.51E-05 | 0.000309 | A |
| STEMS | 0.00039 | 2.54E-04 | 186 | -1.10E-04 | 0.000891 | AB |
| ROOTS_STORAGE | 0.000409 | 1.66E-04 | 185.9 | 8.12E-05 | 0.000736 | AB |
| LEAVES | 0.000647 | 7.74E-05 | 102.2 | 4.93E-04 | 0.0008 | B |
| POLLEN | 0.000757 | 1.19E-04 | 148.8 | 5.21E-04 | 0.000993 | B |
| FLOWERS | 0.000766 | 2.50E-04 | 174.3 | 2.72E-04 | 0.00126 | AB |

__

**VII.a.iv. B6 (pyridoxine)**

Model: lmer(Overall_B6_Perc~Tissue+(1|Faimily),data=B6)

Note: We removed data for any tissue with n<3 species sampled for vitamin B6 (i.e. corresponding to “ROOTS”, “FLOWERS”, and ”WOOD”).

Table g. ANOVA summary of linear mixed-effects model examining the effects of plant tissues on overall B6 percentage.

|  | Sum Sq | Mean Sq | NumDF | DenDF | F value | Pr(>F) |
| --- | --- | --- | --- | --- | --- | --- |
| Tissue | 2.71e-05 | 4.52e-06 | 6 | 191.35 | 9.5617 | 3.422e-09 |

Table h. Pairwise comparison evaluated with the emmeans function. Letters in ‘group’ column denote significantly different tissues.

| Tissue | emmean | SE | df | lower.CL | upper.CL | .group |
| --- | --- | --- | --- | --- | --- | --- |
| PHLOEM | 9.19E-05 | 0.000299 | 191.2 | -0.000498 | 0.000681 | A |
| POLLEN | 5.76E-04 | 0.00023 | 168.1 | 0.000122 | 0.001029 | AB |
| SEEDS | 7.60E-04 | 0.000151 | 116.3 | 0.000461 | 0.001059 | AB |
| FRUIT | 8.29E-04 | 0.000142 | 91.5 | 0.000547 | 0.001111 | AB |
| ROOTS_STORAGE | 1.61E-03 | 0.000305 | 195.9 | 0.001006 | 0.002209 | BC |
| LEAVES | 1.79E-03 | 0.000158 | 131 | 0.001479 | 0.002102 | C |
| STEMS | 1.87E-03 | 0.000465 | 186.4 | 0.000953 | 0.00279 | BC |

__

**VII.a.v. B5 (pantothenate)**

Model: lmer(Overall_B5_Perc~Tissue+(1|Faimily),data=B5)

Note: We removed data for any tissue with n<3 species sampled for vitamin B5 (i.e. corresponding to “ROOTS” and “FLOWERS”).

Table i. ANOVA summary of linear mixed-effects model examining the effects of plant tissues on overall B5 percentage.

|  | Sum Sq | Mean Sq | NumDF | DenDF | F value | Pr(>F) |
| --- | --- | --- | --- | --- | --- | --- |
| Tissue | 5.06e-05 | 8.43e-06 | 6 | 163.69 | 3.7323 | 0.00166 |

Table j. Pairwise comparison evaluated with the emmeans function. Letters in ‘group’ column denote significantly different tissues.

| Tissue | emmean | SE | df | lower.CL | upper.CL | .group |
| --- | --- | --- | --- | --- | --- | --- |
| ROOTS_STORAGE | 0.000581 | 0.000718 | 186 | -0.000834 | 0.002 | AB |
| PHLOEM | 0.000852 | 0.000372 | 111.5 | 0.000115 | 0.00159 | A |
| SEEDS | 0.001104 | 0.000306 | 99.1 | 0.000497 | 0.00171 | A |
| FRUIT | 0.001929 | 0.000248 | 61.5 | 0.001433 | 0.00242 | AB |
| LEAVES | 0.002321 | 0.0003 | 99.7 | 0.001725 | 0.00292 | B |
| POLLEN | 0.002487 | 0.000505 | 131.7 | 0.001487 | 0.00349 | AB |
| STEMS | 0.002774 | 0.000932 | 186 | 0.000935 | 0.00461 | AB |

**VII.a.vi. B3 (niacin/nicotinate)**

Model: lmer(Overall_B3_Perc~Tissue+(1|Faimily),data=B3)

Note: We removed data for any tissue with n<3 species sampled for vitamin B3 “ROOTS_RHIZOM”).

Table k. ANOVA summary of linear mixed-effects model examining the effects of plant tissues on overall B3 percentage.

|  | Sum Sq | Mean Sq | NumDF | DenDF | F value | Pr(>F) |
| --- | --- | --- | --- | --- | --- | --- |
| Tissue | 0.0018 | 0.0003 | 7 | 228.19 | 17.788 | < 2.2e-16 |

Table L. Pairwise comparison evaluated with the emmeans function. Letters in ‘group’ column denote significantly different tissues.

| Tissue | emmean | SE | df | lower.CL | upper.CL | .group |
| --- | --- | --- | --- | --- | --- | --- |
| PHLOEM | 0.00353 | 0.001136 | 214.3 | 0.001295 | 0.00577 | AB |
| ROOTS_STORAGE | 0.00373 | 0.001438 | 250 | 0.000903 | 0.00657 | AB |
| FRUIT | 0.00386 | 0.000565 | 92.6 | 0.002738 | 0.00498 | A |
| SEEDS | 0.00426 | 0.000654 | 131.7 | 0.002964 | 0.00555 | A |
| FLOWERS | 0.00559 | 0.001644 | 245.5 | 0.002354 | 0.00883 | AB |
| STEMS | 0.00647 | 0.001565 | 247.6 | 0.003389 | 0.00955 | AB |
| LEAVES | 0.00722 | 0.000614 | 129 | 0.006008 | 0.00844 | B |
| POLLEN | 0.01586 | 0.001125 | 128.9 | 0.013636 | 0.01809 | C |

__

**VII.a.vii. B2 (riboflavin)**

Model: lmer(Overall_B2_Perc~Tissue+(1|Faimily),data=B2)

Note: We removed data for any tissue with n<3 species sampled for vitamin B2 (i.e. corresponding to “ROOTS_RHIZOM”).

Table m. ANOVA summary of linear mixed-effects model examining the effects of plant tissues on overall B2 percentage.

|  | Sum Sq | Mean Sq | NumDF | DenDF | F value | Pr(>F) |
| --- | --- | --- | --- | --- | --- | --- |
| Tissue | 6.37e-05 | 7.957e-06 | 8 | 222.72 | 36.199 | < 2.2e-16 |

Table n. Pairwise comparison evaluated with the emmeans function. Letters in ‘group’ column denote significantly different tissues.

| Tissue | emmean | SE | df | lower.CL | upper.CL | .group |
| --- | --- | --- | --- | --- | --- | --- |
| PHLOEM | 0.000016 | 1.24E-04 | 193.9 | -0.000228 | 0.00026 | A |
| SEEDS | 0.000323 | 8.67E-05 | 152.3 | 0.000152 | 0.000494 | AB |
| ROOTS_STORAGE | 0.000349 | 1.91E-04 | 255.9 | -0.000027 | 0.000726 | ABC |
| FRUIT | 0.000358 | 7.09E-05 | 95.7 | 0.000217 | 0.000499 | AB |
| FLOWERS | 0.000926 | 1.90E-04 | 254.3 | 0.000551 | 0.0013 | BCD |
| STEMS | 0.001008 | 1.95E-04 | 254.7 | 0.000624 | 0.001392 | CDE |
| ROOTS | 0.001452 | 2.92E-04 | 245.2 | 0.000877 | 0.002028 | DE |
| LEAVES | 0.001561 | 7.84E-05 | 132.2 | 0.001406 | 0.001716 | E |
| POLLEN | 0.001601 | 1.42E-04 | 131.2 | 0.001321 | 0.001881 | DE |

__

**VII.a.viii. B1 (thiamine)**

Model: lmer(Overall_B1_Perc~Tissue+(1|Faimily),data=B1)

Note: We removed data for any tissue with n<3 species sampled for vitamin B1 (i.e. corresponding to “ROOTS_RHIZOM”).

Table o. ANOVA summary of linear mixed-effects model examining the effects of plant tissues on overall B1 percentage.

|  | Sum Sq | Mean Sq | NumDF | DenDF | F value | Pr(>F) |
| --- | --- | --- | --- | --- | --- | --- |
| Tissue | 5.55e-06 | 7.926e-07 | 7 | 174.93 | 6.4622 | 8.866e-07 |

Table p. Pairwise comparison evaluated with the emmeans function. Letters in ‘group’ column denote significantly different tissues.

| Tissue | emmean | SE | df | lower.CL | upper.CL | .group |
| --- | --- | --- | --- | --- | --- | --- |
| ROOTS_STORAGE | 0.000127 | 1.33E-04 | 253 | -0.000134 | 0.000389 | A |
| PHLOEM | 0.000224 | 9.33E-05 | 162.5 | 0.00004 | 0.000408 | A |
| FRUIT | 0.000345 | 5.05E-05 | 89 | 0.000244 | 0.000445 | A |
| FLOWERS | 0.000402 | 1.41E-04 | 252.7 | 0.000125 | 0.000679 | AB |
| SEEDS | 0.00053 | 5.91E-05 | 122.7 | 0.000413 | 0.000647 | AB |
| STEMS | 0.000581 | 1.43E-04 | 250 | 0.000299 | 0.000863 | AB |
| LEAVES | 0.000632 | 5.56E-05 | 126.9 | 0.000522 | 0.000742 | B |
| POLLEN | 0.000898 | 1.41E-04 | 45.4 | 0.000614 | 0.001183 | B |

__

**VII.a.ix. Fiber**

Model: lmer(Overall_fiber_Perc~Tissue+(1|Faimily),data=fiber)

Note: We removed data for any tissue with n<3 species sampled for fiber (i.e. corresponding to “ROOTS_RHIZOM”, “ROOTS”, “SEEDS_pod” and “SEEDS_shell/coat”).

Table q. ANOVA summary of linear mixed-effects model examining the effects of plant tissues on overall neutral detergent fiber percentage.

|  | Sum Sq | Mean Sq | NumDF | DenDF | F value | Pr(>F) |
| --- | --- | --- | --- | --- | --- | --- |
| Tissue | 29727 | 4246.8 | 7 | 364.84 | 31.334 | < 2.2e-16 |

Table r. Pairwise comparison evaluated with the emmeans function. Letters in ‘group’ column denote significantly different tissues.

| Tissue | emmean | SE | df | lower.CL | upper.CL | .group |
| --- | --- | --- | --- | --- | --- | --- |
| POLLEN | 22.5 | 4.99 | 369 | 12.7 | 32.3 | A |
| FLOWERS | 31.7 | 2.98 | 393 | 25.8 | 37.6 | AB |
| SEEDS | 34.1 | 2.46 | 356 | 29.2 | 38.9 | AB |
| FRUIT | 40 | 1.7 | 208 | 36.7 | 43.4 | BC |
| LEAVES | 42.1 | 1.33 | 107 | 39.5 | 44.7 | C |
| UNRIPE_FRUIT | 42.4 | 4.76 | 380 | 33.1 | 51.8 | ABC |
| STEMS | 60.5 | 2.55 | 364 | 55.5 | 65.5 | D |
| WOOD | 76.5 | 3.45 | 394 | 69.7 | 83.3 | E |

__

**VII.b. Examining plant taxonomy and plant tissue effects on nutrient quantities**

The next set of statistical analyses were similar to those above, with the addition of plant family as a fixed effect and of a family * tissue interaction term. Since there was no random effect included, we used a linear regression model. To obtain a balanced design, we included subsets of our data – i.e. only those nutrients and tissues with at least modest, factorial, sampling across two or more plant families.

The results showed that plant family does have an effect on nitrogen content, but that tissue remains a highly significant variable in models of % nitrogen after accounting for family and tissue*family interaction effects.

**VII.b.i. Nitrogen** quantities in LEAVES, POLLEN, ROOTS, WOOD for the FABACEAE VS. PINACEAE plant families.

> mod<-lm(data$Percent.of.dry.weight~data$Tissue*data$Family)

> anova(mod)

Analysis of Variance Table

Response: data$Percent.of.dry.weight

                         Df  Sum Sq Mean Sq F value    Pr(>F)

data$Tissue               3 209.525  69.842 117.977 < 2.2e-16 ***

data$Family               2  36.695  18.348  30.993 3.341e-12 ***

data$Tissue:data$Family   5  33.603   6.721  11.352 1.840e-09 ***

Residuals               170 100.639   0.592

**VII.b.ii. Nitrogen** quantities in LEAVES, POLLEN, WOOD for the FABACEAE VS. PINACEAE VS. FAGACEAE VS. ROSACEAE VS. SALICACEAE VS. BETULACEAE VS. MYRTACEAE plant families.

> mod<-lm(data$Percent.of.dry.weight~data$Tissue*data$Family)

> anova_res <- anova(mod,test="F")

> anova_res

Analysis of Variance Table

Response: data$Percent.of.dry.weight

                         Df Sum Sq Mean Sq F value    Pr(>F)

data$Tissue               2 529.13 264.566 465.332 < 2.2e-16 ***

data$Family               6  55.11   9.185  16.154 1.039e-15 ***

data$Tissue:data$Family  12  72.85   6.071  10.678 < 2.2e-16 ***

Residuals               251 142.71   0.569

**VII.b.iii. Fiber** quantities in FRUIT, LEAVES for the MORACEAE, RUBIACEAE, ROSACEAE, EUPHORBIACEAE, SALICACEAE, PHYLLANTHACEAE plant families.

> mod<-lm(data$Percent.of.dry.weight~data$Tissue*data$Family)

> anova_res <- anova(mod,test="F")

> anova_res

Analysis of Variance Table

Response: data$Percent.of.dry.weight

                        Df Sum Sq Mean Sq F value Pr(>F)

data$Tissue              1    0.0   0.000  0.0000 0.9986

data$Family              4  392.0  98.005  0.8228 0.5151

data$Tissue:data$Family  4  639.2 159.794  1.3416 0.2634

Residuals               69 8218.5 119.108

**VII.c. Examining effects of plant taxonomy on B-vitamin content of seeds.**

We continue our attempts to understand how plant taxonomy shapes nutritive content. Here we utilize B-vitamin data for seeds, performing pairwise 2-tailed t-tests to assess variation in B-vitamins among plant families with n≥5 species measures.

**Bold-** significant after Benjamini-Hochberg correction (12 tests and false-discovery rate of 0.05)

*Italics* – significant after Bonferroni correction.

**VIII. Obligate symbionts vs. dietary nutrition**

We used generalized linear (GLM) models to assess whether nutrient data from insect diets predicted tendencies of insect families to harbor obligate symbionts. Response variables were binary (‘1’ = family had obligate symbiont; ‘0’ = family had no known obligate symbiont). Among our X-variables (% dry weight of fiber, nitrogen, or B-vitamins in the predominant food of the given insect families), we log-transformed B-vitamin data due to strong skew. Analyses for all B-vitamins were performed with and without data for blood feeding, helping to assess pattern robustness. Relevant p-values are highlighted in yellow; those significant are presented in bold font.

**__**

**VIII.a.i. B1 (thiamine) with Blood**

model2 <- glm(obligate_sym ~ log(B1),data = B1_with_blood_data, family = binomial())

summary(model2)

Call:

glm(formula = obligate_sym ~ log(B1), family = binomial(), data = B1_with_blood_data)

Coefficients:

Estimate Std. Error

(Intercept) -26.8427	3.4586

log(B1)	-3.1964	0.4381

z value Pr(>|z|)

(Intercept) -7.761 8.41e-15 ***

log(B1)	-7.297 2**.95e-13 *****

---

Signif. codes:

0 ‘***’ 0.001 ‘**’ 0.01 ‘*’ 0.05

‘.’ 0.1 ‘ ’ 1

(Dispersion parameter for binomial family taken to be 1)

Null deviance: 374.95 on 472 degrees of freedom

Residual deviance: 234.02 on 471 degrees of freedom

AIC: 238.02

Number of Fisher Scoring iterations: 7

__

**VIII.a.ii. B1 (thiamine) without Blood**

model2 <- glm(obligate_sym ~ log(B1),data = B1_without_blood_data, family = binomial())

summary(model2)

Call:

glm(formula = obligate_sym ~ log(B1), family = binomial(), data = B1_without_blood_data)

Coefficients:

Estimate Std. Error

(Intercept) -39.0668	4.4281

log(B1)	-4.7560	0.5626

z value Pr(>|z|)

(Intercept) -8.822	<2e-16 ***

log(B1)	-8.454	**<2e-16 *****

---

Signif. codes:

0 ‘***’ 0.001 ‘**’ 0.01 ‘*’ 0.05

‘.’ 0.1 ‘ ’ 1

(Dispersion parameter for binomial family taken to be 1)

Null deviance: 319.99 on 457 degrees of freedom

Residual deviance: 189.39 on 456 degrees of freedom

AIC: 193.39

Number of Fisher Scoring iterations: 7

__

**VIII.b.i. B2 (riboflavin) with Blood**

model2 <- glm(obligate_sym ~ log(B2),data = B2_with_blood_data, family = binomial())

summary(model2)

Call:

glm(formula = obligate_sym ~ log(B2), family = binomial(), data = B2_with_blood_data)

Coefficients:

Estimate Std. Error

(Intercept) -15.3249	1.4855

log(B2)	-1.7197	0.1889

z value Pr(>|z|)

(Intercept) -10.316	<2e-16 ***

log(B2)	-9.105	**<2e-16 *****

---

Signif. codes:

0 ‘***’ 0.001 ‘**’ 0.01 ‘*’ 0.05

‘.’ 0.1 ‘ ’ 1

(Dispersion parameter for binomial family taken to be 1)

Null deviance: 374.95 on 472 degrees of freedom

Residual deviance: 153.00 on 471 degrees of freedom

AIC: 157

Number of Fisher Scoring iterations: 6

__

**VIII.b.ii. B2 (riboflavin) without Blood**

model2 <- glm(obligate_sym ~ log(B2),data = B2_without_blood_data, family = binomial())

summary(model2)

Call:

glm(formula = obligate_sym ~ log(B2), family = binomial(), data = B2_without_blood_data)

Coefficients:

Estimate Std. Error

(Intercept) -15.9808	1.9139

log(B2)	-1.8112	0.2522

z value Pr(>|z|)

(Intercept) -8.350 < 2e-16 ***

log(B2)	-7.181 **6.91e-13 *****

---

Signif. codes:

0 ‘***’ 0.001 ‘**’ 0.01 ‘*’ 0.05

‘.’ 0.1 ‘ ’ 1

(Dispersion parameter for binomial family taken to be 1)

Null deviance: 319.99 on 457 degrees of freedom

Residual deviance: 140.80 on 456 degrees of freedom

AIC: 144.8

Number of Fisher Scoring iterations: 7

__

**VIII.c.i. B3 (niacin/nicotinate) with Blood**

model2 <- glm(obligate_sym ~ log(B3),data = B3_with_blood_data, family = binomial())

summary(model2)

Call:

glm(formula = obligate_sym ~ log(B3), family = binomial(), data = B3_with_blood_data)

Coefficients:

Estimate Std. Error

(Intercept) -23.9274	2.2612

log(B3)	-4.4043	0.4303

z value Pr(>|z|)

(Intercept) -10.58	<2e-16 ***

log(B3)	-10.23	**<2e-16 *****

---

Signif. codes:

0 ‘***’ 0.001 ‘**’ 0.01 ‘*’ 0.05

‘.’ 0.1 ‘ ’ 1

(Dispersion parameter for binomial family taken to be 1)

Null deviance: 374.95 on 472 degrees of freedom

Residual deviance: 153.41 on 471 degrees of freedom

AIC: 157.41

Number of Fisher Scoring iterations: 7

__

**VIII.c.ii. B3 (niacin/nicotinate) without Blood**

model2 <- glm(obligate_sym ~ log(B3),data = B3_without_blood_data, family = binomial())

summary(model2)

Call:

glm(formula = obligate_sym ~ log(B3), family = binomial(), data = B3_without_blood_data)

Coefficients:

Estimate Std. Error

(Intercept) -24.6701	2.4635

log(B3)	-4.5561	0.4733

z value Pr(>|z|)

(Intercept) -10.014	<2e-16 ***

log(B3)	-9.627	**<2e-16 *****

---

Signif. codes:

0 ‘***’ 0.001 ‘**’ 0.01 ‘*’ 0.05

‘.’ 0.1 ‘ ’ 1

(Dispersion parameter for binomial family taken to be 1)

Null deviance: 319.99 on 457 degrees of freedom

Residual deviance: 140.91 on 456 degrees of freedom

AIC: 144.91

Number of Fisher Scoring iterations: 7

__

**VIII.d.i. B5 (pantothenate) with Blood**

model2 <- glm(obligate_sym ~ log(B5),data = B5_with_blood_data, family = binomial())

summary(model2)

Call:

glm(formula = obligate_sym ~ log(B5), family = binomial(), data = B5_with_blood_data)

Coefficients:

Estimate Std. Error

(Intercept) -20.8691	2.0422

log(B5)	-3.0713	0.3234

z value Pr(>|z|)

(Intercept) -10.219	<2e-16 ***

log(B5)	-9.496	**<2e-16 *****

---

Signif. codes:

0 ‘***’ 0.001 ‘**’ 0.01 ‘*’ 0.05

‘.’ 0.1 ‘ ’ 1

(Dispersion parameter for binomial family taken to be 1)

Null deviance: 374.95 on 472 degrees of freedom

Residual deviance: 177.10 on 471 degrees of freedom

AIC: 181.1

Number of Fisher Scoring iterations: 6

__

**VIII.d.ii. B5 (pantothenate) without Blood**

model2 <- glm(obligate_sym ~ log(B5),data = B5_without_blood_data, family = binomial())

summary(model2)

Call:

glm(formula = obligate_sym ~ log(B5), family = binomial(), data = B5_without_blood_data)

Coefficients:

Estimate Std. Error

(Intercept) -26.3871	2.6264

log(B5)	-3.9686	0.4196

z value Pr(>|z|)

(Intercept) -10.047	<2e-16 ***

log(B5)	-9.458	**<2e-16 *****

---

Signif. codes:

0 ‘***’ 0.001 ‘**’ 0.01 ‘*’ 0.05

‘.’ 0.1 ‘ ’ 1

(Dispersion parameter for binomial family taken to be 1)

Null deviance: 319.99 on 457 degrees of freedom

Residual deviance: 136.62 on 456 degrees of freedom

AIC: 140.62

Number of Fisher Scoring iterations: 6

__

**VIII.e.i. B6 (pyridoxine) with Blood**

model2 <- glm(obligate_sym ~ log(B6),data = B6_with_blood_data, family = binomial())

summary(model2)

Call:

glm(formula = obligate_sym ~ log(B6), family = binomial(), data = B6_with_blood_data)

Coefficients:

Estimate Std. Error

(Intercept) -19.569	1.918

log(B6)	-2.414	0.254

z value Pr(>|z|)

(Intercept) -10.203	<2e-16 ***

log(B6)	-9.507	**<2e-16 *****

---

Signif. codes:

0 ‘***’ 0.001 ‘**’ 0.01 ‘*’ 0.05

‘.’ 0.1 ‘ ’ 1

(Dispersion parameter for binomial family taken to be 1)

Null deviance: 402.22 on 490 degrees of freedom

Residual deviance: 204.67 on 489 degrees of freedom

AIC: 208.67

Number of Fisher Scoring iterations: 6

__

**VIII.e.ii. B6 (pyridoxine) without Blood**

model2 <- glm(obligate_sym ~ log(B6),data = B6_without_blood_data, family = binomial())

summary(model2)

Call:

glm(formula = obligate_sym ~ log(B6), family = binomial(), data = B6_without_blood_data)

Coefficients:

Estimate Std. Error

(Intercept) -23.7058	2.3069

log(B6)	-2.9755	0.3061

z value Pr(>|z|)

(Intercept) -10.276	<2e-16 ***

log(B6)	-9.722	**<2e-16 *****

---

Signif. codes:

0 ‘***’ 0.001 ‘**’ 0.01 ‘*’ 0.05

‘.’ 0.1 ‘ ’ 1

(Dispersion parameter for binomial family taken to be 1)

Null deviance: 348.83 on 475 degrees of freedom

Residual deviance: 171.22 on 474 degrees of freedom

AIC: 175.22

Number of Fisher Scoring iterations: 6

__

**VIII.f.i. B7 (biotin) with Blood**

model2 <- glm(obligate_sym ~ log(B7),data = B7_with_blood_data, family = binomial())

summary(model2)

Call:

glm(formula = obligate_sym ~ log(B7), family = binomial(), data = B7_with_blood_data)

Coefficients:

Estimate Std. Error

(Intercept) -11.36945	0.89957

log(B7)	-0.83415	0.07265

z value Pr(>|z|)

(Intercept) -12.64	<2e-16 ***

log(B7)	-11.48	**<2e-16 *****

---

Signif. codes:

0 ‘***’ 0.001 ‘**’ 0.01 ‘*’ 0.05

‘.’ 0.1 ‘ ’ 1

(Dispersion parameter for binomial family taken to be 1)

Null deviance: 402.22 on 490 degrees of freedom

Residual deviance: 200.58 on 489 degrees of freedom

AIC: 204.58

Number of Fisher Scoring iterations: 6

__

**VIII.f.ii. B7 (biotin) without Blood**

model2 <- glm(obligate_sym ~ log(B7),data = B7_without_blood_data, family = binomial())

summary(model2)

Call:

glm(formula = obligate_sym ~ log(B7), family = binomial(), data = B7_without_blood_data)

Coefficients:

Estimate Std. Error

(Intercept) -11.44064	0.95177

log(B7)	-0.84108	0.07869

z value Pr(>|z|)

(Intercept) -12.02	<2e-16 ***

log(B7)	-10.69	**<2e-16 *****

---

Signif. codes:

0 ‘***’ 0.001 ‘**’ 0.01 ‘*’ 0.05

‘.’ 0.1 ‘ ’ 1

(Dispersion parameter for binomial family taken to be 1)

Null deviance: 348.83 on 475 degrees of freedom

Residual deviance: 188.75 on 474 degrees of freedom

AIC: 192.75

Number of Fisher Scoring iterations: 6

__

**VIII.g.i. B9 (folate) with Blood**

model2 <- glm(obligate_sym ~ log(B9),data = B9_with_blood_data, family = binomial())

summary(model2)

Call:

glm(formula = obligate_sym ~ log(B9), family = binomial(), data = B9_with_blood_data)

Coefficients:

Estimate Std. Error

(Intercept) -11.9040	1.4972

log(B9)	-1.0295	0.1583

z value Pr(>|z|)

(Intercept) -7.951 1.85e-15 ***

log(B9)	-6.504 **7.80e-11 *****

---

Signif. codes:

0 ‘***’ 0.001 ‘**’ 0.01 ‘*’ 0.05

‘.’ 0.1 ‘ ’ 1

(Dispersion parameter for binomial family taken to be 1)

Null deviance: 223.94 on 437 degrees of freedom

Residual deviance: 169.83 on 436 degrees of freedom

AIC: 173.83

Number of Fisher Scoring iterations: 6

__

**VIII.g.ii. B9 (folate) without Blood**

model2 <- glm(obligate_sym ~ log(B9),data = B9_without_blood_data, family = binomial())

summary(model2)

Call:

glm(formula = obligate_sym ~ log(B9), family = binomial(), data = B9_without_blood_data)

Coefficients:

Estimate Std. Error

(Intercept) -4.2842	2.4757

log(B9)	-0.1392	0.2914

z value Pr(>|z|)

(Intercept) -1.730	0.0835 .

log(B9)	-0.478	0.6329

---

Signif. codes:

0 ‘***’ 0.001 ‘**’ 0.01 ‘*’ 0.05

‘.’ 0.1 ‘ ’ 1

(Dispersion parameter for binomial family taken to be 1)

Null deviance: 148.87 on 422 degrees of freedom

Residual deviance: 148.64 on 421 degrees of freedom

AIC: 152.64

Number of Fisher Scoring iterations: 6

__

**VIII.h. Fiber**

model2 <- glm(obligate_sym ~ fiber,data = fiber_data, family = binomial())

summary(model2)

Call:

glm(formula = obligate_sym ~ fiber, family = binomial(), data = fiber_data)

Coefficients:

Estimate Std. Error

(Intercept) -2.78120	0.77963

fiber	0.02283	0.01515

z value Pr(>|z|)

(Intercept) -3.567 0.000361 ***

fiber	1.507 0.131750

---

Signif. codes:

0 ‘***’ 0.001 ‘**’ 0.01 ‘*’ 0.05

‘.’ 0.1 ‘ ’ 1

(Dispersion parameter for binomial family taken to be 1)

Null deviance: 100.794 on 117 degrees of freedom

Residual deviance: 98.662 on 116 degrees of freedom

AIC: 102.66

Number of Fisher Scoring iterations: 4

__

**VIII.i. Nitrogen**

model2 <- glm(obligate_sym ~ N,data = nitrogen_data, family = binomial())

summary(model2)

Call:

glm(formula = obligate_sym ~ N, family = binomial(), data = nitrogen_data)

Coefficients:

Estimate Std. Error

(Intercept) -0.40934	0.21070

N	-0.28331	0.04147

z value Pr(>|z|)

(Intercept) -1.943	0.052 .

N	-6.832 **8.36e-12 *****

---

Signif. codes:

0 ‘***’ 0.001 ‘**’ 0.01 ‘*’ 0.05

‘.’ 0.1 ‘ ’ 1

(Dispersion parameter for binomial family taken to be 1)

Null deviance: 429.70 on 559 degrees of freedom

Residual deviance: 366.21 on 558 degrees of freedom

AIC: 370.21

Number of Fisher Scoring iterations: 6

__

**IX. Symbiont metabolism/function vs. dietary nutrition – additional results and discussion**

**IX.a. Nitrogen recycling/fixation results - metabolism**

More limited than the amino acid- and B-vitamin-encoding metabolisms were those involving the recycling and fixation of nitrogen. Nitrogen fixation KOs were found in just six non-redundant obligate microbiomes in our analysis – one from a leafcutter ant fungal garden, two from the guts of wood-feeding termites, one from the gut of the Asian long-horned beetle, and two from newly acquired *Dactylopiibacterium* symbionts of phloem-feeding cochineal scale insects (Table S76 – [Zenodo](https://doi.org/10.5281/zenodo.19607748) repository).

Urease-comprising nitrogen recycling proteins were more common, being present across 22 non-redundant obligate microbiomes. We found no correlation between the number of unique urease-encoding KOs vs. % dietary nitrogen, even after removal of metagenomes and recently domesticated symbionts (Table S79 – [Zenodo](https://doi.org/10.5281/zenodo.19607748) repository). Urease genes were found in obligate microbiomes of omnivores, predators, and specialized consumers of blood, pollen, seeds, fruit, wood, and phloem sap (Table S76 – [Zenodo](https://doi.org/10.5281/zenodo.19607748) repository). While enriched among the microbiomes of wood-feeders, and somewhat common among seed-feeders, enrichment was less apparent among the other dietary guilds. The most notable paucity of N-recycling was seen among well-represented phloem sap-feeding microbiomes, given the low nitrogen content of their hosts’ diets. Rare production of urea and other uricolytic products in sap-feeders’ nitrogen-waste processing (e.g. Sasaki et al. 1990; Brodbeck et al. 1993; Crafts-Bandner 2002) makes this paucity less surprising.

**IX.b. Nitrogen recycling/fixation results - function**

While perhaps not common amongst the separately evolved symbioses of insects, nitrogen fixation activity has been inferred *in vivo* from many dozens of studies with the aid of acetylene reduction assays (Table S70 – [Zenodo](https://doi.org/10.5281/zenodo.19607748) repository). Indeed, of the 67 insect species with *in vivo* evidence for some microbial N-fixation, 61 were studied using this technique. Disproportionately represented amongst these are findings from termites, where N-fixation levels seem to correlate with nitrogen scarcity in the diet, being most pronounced for wood-feeders (Breznak 2000). We assessed evidence for host insect acquisition of symbiont-fixed nitrogen and found support in only nine species (Table S70 – [Zenodo](https://doi.org/10.5281/zenodo.19607748) repository), representing six origins of obligate symbiosis spanning wood wasps, fungus-growing ants, termites, and three beetle lineages (Täyasu et al. 1994; 1997; 2002; Pinto-Tomás et al. 2009; Thompson 2013; Ayayee et al. 2014; Ceja-Navarro et al. 2014; Mifsud et al. 2023). Also hosting nitrogen-fixers was a species of cranefly without a demonstrated obligate symbiosis (Kostina et al. 2020). Wood was the primary diet for larval hosts acquiring symbiont-fixed nitrogen, with exceptions for the cranefly (detritus) and leafcutter ants (eating fungus reared on excised leaves).

While more common than nitrogen fixation at the genetic level, insect acquisition of symbiont-recycled nitrogen was found for only seven insect species (Table S69 – [Zenodo](https://doi.org/10.5281/zenodo.19607748) repository). These spanned six separate origins of symbiosis distributed across three beetle lineages, a tephritid fruit fly (*Bactrocera oleae*), and two ants with separate symbiotic origins (Jurzitza 1970, 1972; Feldhaar et al. 2007; Ayayee et al. 2014; Ben-Yosef et al. 2014; Alonso-Pernas et al. 2017; Hu et al. 2018). A second tephritid (*Bactrocera dorsalis*) with a less-described symbiosis also acquires symbiont-recycled nitrogen (Ren et al. 2022). Diets of these hosts include pollen, wood, roots, and seeds, with at least occasional consumption of insect tissue by carpenter ants. The aforementioned tephritids eat fruit as larvae, but their symbioses may be relevant during adulthood, when they consume bird droppings and sap-feeders’ honeydew waste (Bateman 1972). These foods are also consumed by adult *Cephalotes* ants (Adams 1990; Cembrowski et al. 2015), hosts to a similar adult-active nitrogen-recycling symbiosis (Béchade et al. 2023).

**IX.c. Fiber breakdown results - metabolism**

While fixation and recycling may sometimes address nitrogen shortcomings, fiber catabolic function may shape carbon/energy budgets or digestive access to closely guarded, nutritious cell content (Wu et al. 2021). We found significantly positive correlations between the numbers of unique symbiont-encoded, fiber-catabolizing KOs vs. the neutral detergent fiber content of the diet (Tables S76; S79 – [Zenodo](https://doi.org/10.5281/zenodo.19607748) repository). Such relationships held for each cellulose, hemicellulose, pectin, and lignin catabolism. They suggest higher investment in the breakdown of recalcitrant carbon compounds when more carbon is such imprisoned.

While this fit the general expectation that symbioses have evolved to match dietary shortcomings – mirroring amino acid and B-vitamin trends above – several caveats warrant discussion. First, lignocellulose degradation is a complex process, with diverse solutions across microorganisms (Cragg et al. 2015; Andlar et al. 2018; Wertz & Béchade 2020). Our protein screening has undoubtedly omitted proteins of importance in these processes. And while insects do not encode capacities for nitrogen fixation, urease, or full pathways for B-vitamin or essential amino acid synthesis (but see Husnik & McCutcheon 2016; Ren et al. 2020), several encode endogenous or laterally transferred fiber catabolizing genes (Calderón-Cortés et al. 2012; Pauchet & Heckel 2013; Kirsch et al. 2014; Shelomi et al. 2016; Wybouw et al. 2016).

Second, among the primary diets used by insects with full obligate microbiome genomics, only five had direct fiber measures – fruit, pollen, leaves, seeds, and wood. To expand statistical power, we assumed 0% fiber content of phloem sap, xylem sap, and blood (Table S78 – [Zenodo](https://doi.org/10.5281/zenodo.19607748) repository). A related consideration stems from the absence of pectin data, and the fact that neutral detergent fiber (cellulose, lignin, and hemicellulose) comprises an imperfect proxy for pectin. Despite this, such fiber levels do predict the numbers of symbiont-encoding, pectin-degrading KOs.

A third consideration extends from the very low likelihood that some symbioses could ever be fiber-catabolizing due to their localization. Fiber catabolism is most expected for symbionts with some gut residence during host development, for those living in organs connecting directly to the gut (e.g. Salem et al. 2017; Reis et al. 2020), or for those that grow in fiber-rich, outside-of-body habitats during host development (e.g. Li et al. 2021). A large number of symbioses analyzed here are bacteriocyte-confined, checking none of these boxes. The absence of fiber-catabolizing genes in such symbionts was expected.

A fourth caveat is specific to lignin, as two of the six examined lignin-degrading enzymes possessed alternative functions, shaping purine, cysteine, and methionine metabolism or the synthesis of anti-fungals. Detection of these enzymes (K05810, K00433) made up all but two of the positive results across our lignin degradation dataset (Table S40 – [Zenodo](https://doi.org/10.5281/zenodo.19607748) repository), revealing potential confoundment.

Outlier datapoints comprise a final caveat. Included in this category are findings of pectinases in recently domesticated symbionts of phloem-feeding scale insects (Bustamante-Brito et al. 2019), and the gut microbiomes of pollen-feeding honeybees and cephalotine ants. These latter microbiomes further encoded more cellulose- and hemicellulose-degrading KOs than expected from our regression analysis.

**IX.d. Fiber breakdown results - function**

Findings from the experimental literature matched expectations from fiber-focused genome results (Table S68 – [Zenodo](https://doi.org/10.5281/zenodo.19607748) repository). *In vivo* evidence for fiber catabolism was obtained across 76 species from 21 insect families and seven orders. But amongst the 103 supportive publications we found direct evidence for fiber-derived carbon acquisition in only seven insect species – two termites, a cockroach, a scarab beetle, a cricket, a crane fly, and a caddisfly (Martoja 1962; Mauldin et al. 1972; Bignell 1977; Bayon & Mathelin 1980; Odelson & Breznak 1983; Sinsabaugh et al. 1985). These represented only two origins of obligate symbiosis, with the latter three cases being examples of understudied symbioses. Compared to lignin, pectin, and hemicellulose, cellulose was the most frequent fiber source for host-acquired carbon in papers attempting direct assessment (Table S68 – [Zenodo](https://doi.org/10.5281/zenodo.19607748) repository).

For several cockroaches and termites, a honeybee species, two beetles (Passalidae, Scarabaeidae), and two cricket species there was documented evidence of symbiont-produced short chain fatty acids (SCFAs), a potential boon to insect energy budgets (e.g. Breznak & Switzer 1986; Kaufman & Klug 1990; Cazemier et al. 1997; Zheng et al. 2017; Ceja-Navarro et al. 2019). Efforts that tracked SCFA origins suggested cellulose was the most common source amongst the four fiber categories (Table S68 – [Zenodo](https://doi.org/10.5281/zenodo.19607748) repository). Consistent with this was the higher incidence of *in vivo* symbiont cellulose catabolism (evidenced in n=42 insect species) compared to hemicellulose (n=33), pectin (n=24), or lignin (n-20). This equated to greater cellulase detection through enzymatic assays, transcriptomics, proteomics, or substrate/product measures. But the seemingly rarer breakdown of other fibers could partially reflect some research bias.

Across separately derived symbioses – clearly obligate or not – fiber catabolism was most often seen in host-microbe systems with gut chamber enlargement, slow gut transit, and large microbial biomass in extracellular gut environments. This syndrome was evidenced amongst a subset of the world’s scarab, cerambycid, and passalid beetles; weevils; termites and cockroaches; crane flies; caddisflies; corbiculate bees; and crickets. In holometabolous insects, lignocellulose and pectin-metabolizing symbioses appeared primarily relevant at the larval stages, with exceptions for the passalid beetle and corbiculate bee symbioses (Zheng et al. 2017; Ceja-Navarro et al. 2019).

It should be mentioned that some symbioses with important carbon-catabolic/energy function may not invest solely or primarily in the breakdown of the aforementioned plant fibers. Metabolized, instead, may be other abundant plant compounds including flavonoids, gamma-hydroxy acids, phenolamides, starches, and varying mono- and di-saccharides (de Fine Licht et al. 2010; Tanahashi et al. 2010; de Fine Licht & Biedermann 2012; Kešnerová et al. 2017; Li et al. 2017b; Lee et al. 2018; Huang et al. 2019; Toki & Aoki 2021). Fermentation of such substances could further enable host acquisition of energy-rich SCFAs.

__

**X. Amino acid content in varying insect foods**

**X.a. Analysis 1: Plant tissues only** – linear mixed effect model with % (dry weight) amino acid content as y-variable; plant tissue (for all tissues with n≥3) as a fixed effect, and plant family as a random effect.

**X.a.i. Alanine**

> full_lm_Arginine <- lmer(Arginine~Broad_food_category+(1|Family),data=AA_data_stats)

> anova(full_lm_Arginine)

> table <- emmeans(full_lm_Arginine, ~ Broad_food_category)

> multcomp::cld(table, Letters = LETTERS, alpha=0.05)

|  | Df | Sum Sq | Mean Sq | F value | Pr(>F) |
| --- | --- | --- | --- | --- | --- |
| Tissue | 9 | 50.781 | 5.6432 | 71.054 | < 2.2e-16 *** |

| Tissue | emmean | SE | df | lower.CL | upper.CL | .group |
| --- | --- | --- | --- | --- | --- | --- |
| PHLOEM | -0.07495 | 0.119 | 172.6 | -0.3097 | 0.16 | A |
| WOOD | -0.00957 | 0.143 | 171.5 | -0.2923 | 0.273 | B |
| NECTAR | 0.04634 | 0.0599 | 105 | -0.0724 | 0.165 | B |
| XYLEM | 0.05046 | 0.136 | 174 | -0.2174 | 0.318 | C |
| ROOT_storage | 0.33061 | 0.0852 | 160.9 | 0.1624 | 0.499 | D |
| FRUIT | 0.35134 | 0.058 | 72.8 | 0.2357 | 0.467 | D |
| SEEDS | 0.89108 | 0.0607 | 103.5 | 0.7707 | 1.012 | E |
| STEMS | 1.10942 | 0.173 | 174 | 0.768 | 1.451 | EF |
| LEAVES | 1.39785 | 0.0742 | 137.2 | 1.251 | 1.545 | F |
| POLLEN | 1.50107 | 0.0657 | 90.9 | 1.3705 | 1.632 | F |

__

**X.a.ii. Arginine**

|  | Df | Sum Sq | Mean Sq | F value | Pr(>F) |
| --- | --- | --- | --- | --- | --- |
| Tissue | 9 | 74.32 | 8.2578 | 43.736 | < 2.2e-16 *** |

| Tissue | emmean | SE | df | lower.CL | upper.CL | .group |
| --- | --- | --- | --- | --- | --- | --- |
| WOOD | -0.2208 | 0.226 | 179 | -0.6676 | 0.226 | A |
| PHLOEM | 0.0287 | 0.188 | 179 | -0.3429 | 0.4 | B |
| XYLEM | 0.0508 | 0.198 | 180 | -0.3392 | 0.441 | C |
| NECTAR | 0.1221 | 0.0996 | 111 | -0.0753 | 0.319 | D |
| FRUIT | 0.2796 | 0.0965 | 92 | 0.0879 | 0.471 | E |
| ROOT_storage | 0.6141 | 0.138 | 166 | 0.342 | 0.886 | F |
| STEMS | 1.0736 | 0.274 | 179 | 0.5328 | 1.614 | FG |
| LEAVES | 1.513 | 0.112 | 153 | 1.2914 | 1.735 | G |
| POLLEN | 1.6173 | 0.108 | 129 | 1.4041 | 1.83 | G |
| SEEDS | 1.6529 | 0.099 | 119 | 1.4568 | 1.849 | G |

**__**

**X.a.iii. Asparagine**

|  | Df | Sum Sq | Mean Sq | F value | Pr(>F) |
| --- | --- | --- | --- | --- | --- |
| Tissue | 2 | 0.38447 | 0.19224 | 5.3245 | 0.009199 ** |

| Tissue | emmean | SE | df | lower.CL | upper.CL | .group |
| --- | --- | --- | --- | --- | --- | --- |
| PHLOEM | -0.0986 | 0.132 | 39.2 | -0.3663 | 0.169 | A |
| XYLEM | -0.0225 | 0.133 | 37.4 | -0.2918 | 0.247 | B |
| NECTAR | 0.2376 | 0.0774 | 21.5 | 0.0769 | 0.398 | C |

**__**

**X.a.iv. Aspartate**

|  | Df | Sum Sq | Mean Sq | F value | Pr(>F) |
| --- | --- | --- | --- | --- | --- |
| Tissue | 9 | 168.71 | 18.745 | 35.009 | < 2.2e-16 *** |

| Tissue | emmean | SE | df | lower.CL | upper.CL | .group |
| --- | --- | --- | --- | --- | --- | --- |
| PHLOEM | -0.20916 | 0.321 | 169.1 | -0.844 | 0.425 | A |
| NECTAR | -0.00999 | 0.175 | 104 | -0.356 | 0.336 | B |
| XYLEM | 0.0442 | 0.606 | 146.4 | -1.153 | 1.241 | B |
| WOOD | 0.04768 | 0.386 | 168.2 | -0.714 | 0.809 | B |
| FRUIT | 1.03233 | 0.173 | 82.7 | 0.688 | 1.377 | C |
| ROOT_storage | 1.11856 | 0.238 | 155.1 | 0.648 | 1.589 | C |
| SEEDS | 1.94978 | 0.174 | 118.6 | 1.604 | 2.295 | D |
| LEAVES | 2.62603 | 0.209 | 148.2 | 2.213 | 3.04 | DE |
| POLLEN | 2.74598 | 0.187 | 130.4 | 2.376 | 3.116 | E |
| STEMS | 3.81091 | 0.47 | 170.4 | 2.884 | 4.738 | E |

**__**

**X.a.v. Cysteine**

|  | Df | Sum Sq | Mean Sq | F value | Pr(>F) |
| --- | --- | --- | --- | --- | --- |
| Tissue | 8 | 2.751 | 0.34387 | 39.455 | < 2.2e-16 *** |

| Tissue | emmean | SE | df | lower.CL | upper.CL | .group |
| --- | --- | --- | --- | --- | --- | --- |
| PHLOEM | -0.0051 | 0.0681 | 159.2 | -0.1396 | 0.1294 | A |
| NECTAR | 0.0113 | 0.0185 | 100 | -0.0253 | 0.0479 | A |
| XYLEM | 0.012 | 0.0491 | 159.9 | -0.0849 | 0.1089 | A |
| FRUIT | 0.0774 | 0.0182 | 58.5 | 0.041 | 0.1139 | B |
| ROOT_storage | 0.1451 | 0.027 | 152.3 | 0.0918 | 0.1985 | B |
| SEEDS | 0.2398 | 0.019 | 70 | 0.2019 | 0.2777 | C |
| STEMS | 0.2564 | 0.0557 | 161 | 0.1463 | 0.3665 | C |
| LEAVES | 0.2694 | 0.0232 | 117.1 | 0.2234 | 0.3154 | C |
| POLLEN | 0.3997 | 0.0205 | 37.9 | 0.3583 | 0.4412 | D |

**__**

**X.a.vi. Glutamine**

|  | Df | Sum Sq | Mean Sq | F value | Pr(>F) |
| --- | --- | --- | --- | --- | --- |
| Tissue | 2 | 1.6127 | 0.80635 | 3.2348 | 0.05058 |

| Tissue | emmean | SE | df | lower.CL | upper.CL | .group |
| --- | --- | --- | --- | --- | --- | --- |
| XYLEM | -0.0066 | 0.249 | 33.5 | -0.514 | 0.5 | A |
| PHLOEM | 0.0921 | 0.275 | 34.6 | -0.466 | 0.65 | A |
| NECTAR | 0.5455 | 0.125 | 18.1 | 0.282 | 0.809 | A |

**__**

**X.a.vii. Glutamate**

|  | Df | Sum Sq | Mean Sq | F value | Pr(>F) |
| --- | --- | --- | --- | --- | --- |
| Tissue | 9 | 279.74 | 31.082 | 29.354 | < 2.2e-16 *** |

| Tissue | emmean | SE | df | lower.CL | upper.CL | .group |
| --- | --- | --- | --- | --- | --- | --- |
| WOOD | -0.1101 | 0.535 | 170.4 | -1.167 | 0.947 | A |
| PHLOEM | -0.0811 | 0.445 | 170.6 | -0.96 | 0.798 | A |
| XYLEM | 0.0113 | 0.824 | 149.5 | -1.618 | 1.64 | A |
| NECTAR | 0.0515 | 0.235 | 102.6 | -0.414 | 0.517 | A |
| FRUIT | 1.1675 | 0.232 | 77.8 | 0.707 | 1.628 | B |
| ROOT_storage | 1.5125 | 0.325 | 155.9 | 0.87 | 2.155 | B |
| STEMS | 2.8762 | 0.649 | 170.6 | 1.595 | 4.157 | C |
| POLLEN | 3.047 | 0.254 | 118.5 | 2.543 | 3.551 | C |
| LEAVES | 3.2006 | 0.285 | 143.9 | 2.636 | 3.765 | C |
| SEEDS | 3.4835 | 0.236 | 113.8 | 3.016 | 3.951 | C |

**__**

**X.a.viii. Glycine**

|  | Df | Sum Sq | Mean Sq | F value | Pr(>F) |
| --- | --- | --- | --- | --- | --- |
| Tissue | 9 | 42.559 | 4.7288 | 64.062 | < 2.2e-16 *** |

| Tissue | emmean | SE | df | lower.CL | upper.CL | .group |
| --- | --- | --- | --- | --- | --- | --- |
| WOOD | -0.0255 | 0.136 | 163.2 | -0.2947 | 0.244 | A |
| PHLOEM | 0.0167 | 0.113 | 167.9 | -0.2067 | 0.24 | B |
| XYLEM | 0.0284 | 0.129 | 173.6 | -0.2265 | 0.283 | B |
| NECTAR | 0.037 | 0.0557 | 105.6 | -0.0735 | 0.148 | B |
| FRUIT | 0.289 | 0.0536 | 67.1 | 0.1821 | 0.396 | C |
| ROOT_storage | 0.3068 | 0.0804 | 162.3 | 0.148 | 0.465 | C |
| STEMS | 0.8872 | 0.165 | 174 | 0.5624 | 1.212 | D |
| SEEDS | 0.909 | 0.0568 | 93.3 | 0.7962 | 1.022 | D |
| LEAVES | 1.222 | 0.0697 | 130.7 | 1.0841 | 1.36 | DE |
| POLLEN | 1.3405 | 0.0614 | 70.8 | 1.2182 | 1.463 | E |

**__**

**X.a.ix. Histidine**

|  | Df | Sum Sq | Mean Sq | F value | Pr(>F) |
| --- | --- | --- | --- | --- | --- |
| Tissue | 9 | 12.027 | 1.3363 | 75.872 | < 2.2e-16 *** |

| Tissue | emmean | SE | df | lower.CL | upper.CL | .group |
| --- | --- | --- | --- | --- | --- | --- |
| PHLOEM | -0.04021 | 0.056 | 177.6 | -0.1507 | 0.0702 | A |
| NECTAR | 0.00996 | 0.0282 | 109.3 | -0.0459 | 0.0659 | B |
| XYLEM | 0.01461 | 0.0639 | 179 | -0.1115 | 0.1407 | B |
| WOOD | 0.02692 | 0.0674 | 176.4 | -0.1061 | 0.16 | B |
| FRUIT | 0.13698 | 0.0271 | 78.2 | 0.083 | 0.191 | C |
| ROOT_storage | 0.18176 | 0.0401 | 166.2 | 0.1025 | 0.261 | C |
| SEEDS | 0.4655 | 0.0282 | 104.8 | 0.4095 | 0.5215 | D |
| STEMS | 0.52158 | 0.0814 | 178.9 | 0.361 | 0.6822 | D |
| LEAVES | 0.54852 | 0.0324 | 141.9 | 0.4844 | 0.6127 | D |
| POLLEN | 0.75742 | 0.0309 | 94.9 | 0.696 | 0.8188 | E |

**__**

**X.a.x. Isoleucine**

|  | Df | Sum Sq | Mean Sq | F value | Pr(>F) |
| --- | --- | --- | --- | --- | --- |
| Tissue | 9 | 35.177 | 3.9085 | 65.734 | < 2.2e-16 *** |

| Tissue | emmean | SE | df | lower.CL | upper.CL | .group |
| --- | --- | --- | --- | --- | --- | --- |
| PHLOEM | -0.0576 | 0.105 | 181 | -0.2644 | 0.149 | A |
| WOOD | 0.0204 | 0.126 | 181 | -0.2284 | 0.269 | B |
| NECTAR | 0.0238 | 0.0545 | 111.5 | -0.0843 | 0.132 | BC |
| XYLEM | 0.0425 | 0.12 | 180.5 | -0.1935 | 0.279 | C |
| FRUIT | 0.229 | 0.0522 | 85.2 | 0.1252 | 0.333 | D |
| ROOT_storage | 0.3158 | 0.0761 | 166.8 | 0.1654 | 0.466 | D |
| SEEDS | 0.7772 | 0.0544 | 115.8 | 0.6695 | 0.885 | E |
| STEMS | 0.8099 | 0.152 | 180.7 | 0.5092 | 1.111 | EF |
| POLLEN | 1.1251 | 0.0593 | 120.6 | 1.0076 | 1.242 | F |
| LEAVES | 1.2664 | 0.0612 | 147.3 | 1.1455 | 1.387 | G |

**__**

**X.a.xi. Leucine**

|  | Df | Sum Sq | Mean Sq | F value | Pr(>F) |
| --- | --- | --- | --- | --- | --- |
| Tissue | 9 | 100.03 | 11.114 | 58.079 | < 2.2e-16 *** |

| Tissue | emmean | SE | df | lower.CL | upper.CL | .group |
| --- | --- | --- | --- | --- | --- | --- |
| PHLOEM | -0.1522 | 0.187 | 180.9 | -0.521 | 0.216 | A |
| WOOD | -0.0414 | 0.225 | 180.9 | -0.485 | 0.402 | B |
| NECTAR | 0.0527 | 0.096 | 111.3 | -0.138 | 0.243 | BC |
| XYLEM | 0.0843 | 0.213 | 180.8 | -0.336 | 0.505 | C |
| FRUIT | 0.3338 | 0.0917 | 82.6 | 0.151 | 0.516 | D |
| ROOT_storage | 0.4672 | 0.135 | 167.2 | 0.201 | 0.734 | D |
| STEMS | 1.3196 | 0.272 | 180.7 | 0.784 | 1.855 | E |
| SEEDS | 1.3673 | 0.096 | 112.9 | 1.177 | 1.557 | E |
| POLLEN | 1.9294 | 0.105 | 113 | 1.722 | 2.137 | F |
| LEAVES | 1.9686 | 0.108 | 144.7 | 1.755 | 2.183 | F |

**__**

**X.a.xii. Lysine**

|  | Df | Sum Sq | Mean Sq | F value | Pr(>F) |
| --- | --- | --- | --- | --- | --- |
| Tissue | 9 | 62.82 | 6.98 | 54.819 | < 2.2e-16 *** |

| Tissue | emmean | SE | df | lower.CL | upper.CL | .group |
| --- | --- | --- | --- | --- | --- | --- |
| PHLOEM | -0.1034 | 0.152 | 181 | -0.404 | 0.197 | A |
| WOOD | -0.0229 | 0.183 | 180.9 | -0.385 | 0.339 | A |
| NECTAR | 0.0101 | 0.0785 | 111.3 | -0.145 | 0.166 | A |
| XYLEM | 0.0407 | 0.174 | 180.8 | -0.303 | 0.384 | A |
| FRUIT | 0.3 | 0.075 | 82.8 | 0.151 | 0.449 | B |
| ROOT_storage | 0.3863 | 0.11 | 167.1 | 0.169 | 0.604 | B |
| SEEDS | 0.9462 | 0.0784 | 113.2 | 0.791 | 1.101 | C |
| STEMS | 1.3091 | 0.222 | 180.7 | 0.872 | 1.746 | CD |
| LEAVES | 1.3808 | 0.0884 | 144.9 | 1.206 | 1.556 | D |
| POLLEN | 1.6925 | 0.0856 | 113.7 | 1.523 | 1.862 | E |

**__**

**X.a.xiii. Methionine**

|  | Df | Sum Sq | Mean Sq | F value | Pr(>F) |
| --- | --- | --- | --- | --- | --- |
| Tissue | 9 | 7.4603 | 0.82892 | 81.467 | < 2.2e-16 *** |

| Tissue | emmean | SE | df | lower.CL | upper.CL | .group |
| --- | --- | --- | --- | --- | --- | --- |
| PHLOEM | -0.00978 | 0.0427 | 179.2 | -0.094 | 0.0745 | A |
| NECTAR | 0.01229 | 0.0216 | 110.1 | -0.0306 | 0.0552 | A |
| WOOD | 0.01332 | 0.0514 | 178.6 | -0.0881 | 0.1148 | A |
| XYLEM | 0.02392 | 0.0487 | 180 | -0.0722 | 0.12 | B |
| FRUIT | 0.07975 | 0.0206 | 78.5 | 0.0388 | 0.1207 | C |
| ROOT_storage | 0.11324 | 0.0307 | 167 | 0.0527 | 0.1738 | C |
| SEEDS | 0.27695 | 0.0216 | 107.7 | 0.2341 | 0.3198 | D |
| STEMS | 0.29758 | 0.0621 | 179.8 | 0.1751 | 0.42 | D |
| LEAVES | 0.3601 | 0.0248 | 144.4 | 0.311 | 0.4092 | D |
| POLLEN | 0.64157 | 0.0237 | 100.7 | 0.5946 | 0.6886 | E |

**__**

**X.a.xiv. Phenylalanine**

|  | Df | Sum Sq | Mean Sq | F value | Pr(>F) |
| --- | --- | --- | --- | --- | --- |
| Tissue | 9 | 34.455 | 3.8283 | 43.433 | < 2.2e-16 *** |

| Tissue | emmean | SE | df | lower.CL | upper.CL | .group |
| --- | --- | --- | --- | --- | --- | --- |
| PHLOEM | -0.0917 | 0.127 | 180 | -0.3422 | 0.159 | A |
| XYLEM | 0.0134 | 0.145 | 179.7 | -0.2724 | 0.299 | B |
| WOOD | 0.0295 | 0.153 | 179.9 | -0.2719 | 0.331 | C |
| NECTAR | 0.1696 | 0.0654 | 110.2 | 0.0399 | 0.299 | CD |
| FRUIT | 0.2124 | 0.0624 | 82.8 | 0.0882 | 0.337 | D |
| ROOT_storage | 0.3152 | 0.0918 | 166.3 | 0.1339 | 0.497 | D |
| STEMS | 0.8121 | 0.185 | 179.7 | 0.448 | 1.176 | E |
| SEEDS | 0.8844 | 0.0653 | 113 | 0.7551 | 1.014 | E |
| POLLEN | 1.1709 | 0.0713 | 113.7 | 1.0296 | 1.312 | F |
| LEAVES | 1.2441 | 0.0745 | 148.2 | 1.0968 | 1.391 | F |

**__**

**X.a.xv. Proline**

|  | Df | Sum Sq | Mean Sq | F value | Pr(>F) |
| --- | --- | --- | --- | --- | --- |
| Tissue | 9 | 158.5 | 17.611 | 53.757 | < 2.2e-16 *** |

| Tissue | emmean | SE | df | lower.CL | upper.CL | .group |
| --- | --- | --- | --- | --- | --- | --- |
| PHLOEM | -0.273 | 0.454 | 142 | -1.1714 | 0.625 | A |
| WOOD | 0.127 | 0.324 | 158 | -0.5126 | 0.766 | A |
| NECTAR | 0.243 | 0.175 | 117 | -0.103 | 0.589 | A |
| XYLEM | 0.251 | 0.311 | 162 | -0.3641 | 0.866 | B |
| ROOT_storage | 0.344 | 0.222 | 153 | -0.0946 | 0.783 | C |
| FRUIT | 0.464 | 0.178 | 101 | 0.1113 | 0.816 | C |
| STEMS | 0.828 | 0.405 | 165 | 0.028 | 1.628 | D |
| SEEDS | 0.943 | 0.171 | 124 | 0.6037 | 1.282 | D |
| LEAVES | 1.34 | 0.194 | 151 | 0.9559 | 1.724 | D |
| POLLEN | 3.497 | 0.177 | 143 | 3.148 | 3.846 | E |

**__**

**X.a.xvi. Serine**

|  | Df | Sum Sq | Mean Sq | F value | Pr(>F) |
| --- | --- | --- | --- | --- | --- |
| Tissue | 9 | 43.563 | 4.8403 | 69.251 | < 2.2e-16 *** |

| Tissue | emmean | SE | df | lower.CL | upper.CL | .group |
| --- | --- | --- | --- | --- | --- | --- |
| PHLOEM | -0.0568 | 0.111 | 171.6 | -0.2762 | 0.163 | A |
| WOOD | 0.0279 | 0.134 | 169.9 | -0.2364 | 0.292 | B |
| XYLEM | 0.0399 | 0.127 | 173.9 | -0.2104 | 0.29 | C |
| NECTAR | 0.0719 | 0.0556 | 105.1 | -0.0383 | 0.182 | D |
| ROOT_storage | 0.3083 | 0.0794 | 161.3 | 0.1515 | 0.465 | E |
| FRUIT | 0.345 | 0.0537 | 71.2 | 0.2378 | 0.452 | E |
| SEEDS | 0.938 | 0.0564 | 100.9 | 0.826 | 1.05 | F |
| STEMS | 0.9882 | 0.162 | 174 | 0.6691 | 1.307 | F |
| LEAVES | 1.0064 | 0.0691 | 135.3 | 0.8697 | 1.143 | F |
| POLLEN | 1.5047 | 0.0611 | 85.2 | 1.3833 | 1.626 | G |

**__**

**X.a.xvii. Threonine**

|  | Df | Sum Sq | Mean Sq | F value | Pr(>F) |
| --- | --- | --- | --- | --- | --- |
| Tissue | 9 | 32.957 | 3.6619 | 48.015 | < 2.2e-16 *** |

| Tissue | emmean | SE | df | lower.CL | upper.CL | .group |
| --- | --- | --- | --- | --- | --- | --- |
| PHLOEM | -0.0453 | 0.115 | 176.8 | -0.2729 | 0.182 | A |
| NECTAR | 0.019 | 0.0572 | 112 | -0.0943 | 0.132 | AB |
| XYLEM | 0.0413 | 0.132 | 180.8 | -0.2185 | 0.301 | B |
| WOOD | 0.0438 | 0.139 | 173.3 | -0.2306 | 0.318 | C |
| FRUIT | 0.2342 | 0.054 | 72.6 | 0.1265 | 0.342 | D |
| ROOT_storage | 0.3782 | 0.0822 | 169 | 0.2159 | 0.541 | D |
| SEEDS | 0.6619 | 0.0574 | 99.3 | 0.5479 | 0.776 | E |
| STEMS | 0.8185 | 0.168 | 181 | 0.4875 | 1.15 | EF |
| LEAVES | 1.0767 | 0.0654 | 133.8 | 0.9473 | 1.206 | F |
| POLLEN | 1.1952 | 0.0629 | 81.9 | 1.0702 | 1.32 | F |

**__**

**X.a.xviii. Tryptophan**

|  | Df | Sum Sq | Mean Sq | F value | Pr(>F) |
| --- | --- | --- | --- | --- | --- |
| Tissue | 6 | 1.6979 | 0.28299 | 50.365 | < 2.2e-16 *** |

| Tissue | emmean | SE | df | lower.CL | upper.CL | .group |
| --- | --- | --- | --- | --- | --- | --- |
| PHLOEM | -0.03282 | 0.0378 | 135.7 | -0.1076 | 0.042 | A |
| NECTAR | -0.00014 | 0.016 | 79.7 | -0.0321 | 0.0318 | A |
| FRUIT | 0.081614 | 0.0155 | 51.1 | 0.0504 | 0.1128 | B |
| ROOT_storage | 0.091963 | 0.0237 | 124.7 | 0.045 | 0.1389 | B |
| SEEDS | 0.207201 | 0.0167 | 69.1 | 0.1739 | 0.2405 | C |
| STEMS | 0.285485 | 0.0462 | 135.6 | 0.1942 | 0.3768 | CD |
| LEAVES | 0.334434 | 0.0188 | 94.9 | 0.297 | 0.3718 | D |

**__**

**X.a.xix. Tyrosine**

|  | Df | Sum Sq | Mean Sq | F value | Pr(>F) |
| --- | --- | --- | --- | --- | --- |
| Tissue | 9 | 17.792 | 1.9769 | 44.265 | < 2.2e-16 *** |

| Tissue | emmean | SE | df | lower.CL | upper.CL | .group |
| --- | --- | --- | --- | --- | --- | --- |
| PHLOEM | -0.06713 | 0.0899 | 176.7 | -0.2445 | 0.11 | A |
| WOOD | 0.00701 | 0.108 | 176.5 | -0.2064 | 0.22 | A |
| XYLEM | 0.03956 | 0.103 | 176.9 | -0.1628 | 0.242 | B |
| NECTAR | 0.05726 | 0.0458 | 108.6 | -0.0336 | 0.148 | B |
| FRUIT | 0.16158 | 0.0437 | 81.7 | 0.0745 | 0.249 | C |
| ROOT_storage | 0.1889 | 0.0647 | 164 | 0.0611 | 0.317 | C |
| STEMS | 0.54701 | 0.131 | 176.9 | 0.289 | 0.805 | D |
| SEEDS | 0.55439 | 0.0464 | 111 | 0.4625 | 0.646 | D |
| POLLEN | 0.82863 | 0.0501 | 104.7 | 0.7292 | 0.928 | E |
| LEAVES | 0.88787 | 0.0534 | 146.6 | 0.7823 | 0.993 | E |

**__**

**X.a.xx. Valine**

|  | Df | Sum Sq | Mean Sq | F value | Pr(>F) |
| --- | --- | --- | --- | --- | --- |
| Tissue | 9 | 51.852 | 5.7613 | 68.348 | < 2.2e-16 *** |

| Tissue | emmean | SE | df | lower.CL | upper.CL | .group |
| --- | --- | --- | --- | --- | --- | --- |
| PHLOEM | -0.05795 | 0.132 | 178 | -0.3179 | 0.202 | A |
| WOOD | 0.00419 | 0.15 | 181 | -0.2911 | 0.299 | B |
| NECTAR | 0.05109 | 0.0643 | 112 | -0.0763 | 0.178 | BC |
| XYLEM | 0.05881 | 0.13 | 181 | -0.1979 | 0.315 | C |
| FRUIT | 0.27269 | 0.0614 | 85 | 0.1506 | 0.395 | D |
| ROOT_storage | 0.37368 | 0.0901 | 167 | 0.1958 | 0.552 | D |
| SEEDS | 0.9624 | 0.0642 | 115 | 0.8353 | 1.09 | E |
| STEMS | 1.18584 | 0.181 | 181 | 0.8291 | 1.543 | EF |
| LEAVES | 1.41775 | 0.0723 | 147 | 1.2749 | 1.561 | F |
| POLLEN | 1.46485 | 0.0701 | 117 | 1.3261 | 1.604 | F |

__

**X.b. Analysis 2: Comparing amino acids across all insect foods. All tissues - linear model with % (dry weight) amino acid content as y-variable; food source (for all with n>3) as a fixed effect x-variable**

**X.b.i. Alanine**

lmA <-lm(Alanine~Broad_food_category,data=AA_data_stats)

anova(lmA)

table <- emmeans(lmA, ~Broad_food_category)

|  | Df | Sum Sq | Mean Sq | F value | Pr(>F) |
| --- | --- | --- | --- | --- | --- |
| Tissue | 12 | 624.92 | 52.077 | 102.65 | < 2.2e-16 *** |

| Tissue | emmean | SE | df | lower.CL | upper.CL | .group |
| --- | --- | --- | --- | --- | --- | --- |
| XYLEM | 0.000328 | 0.319 | 256 | -0.6269 | 0.628 | A |
| PHLOEM | 0.001886 | 0.269 | 256 | -0.5283 | 0.532 | B |
| WOOD | 0.033424 | 0.319 | 256 | -0.5939 | 0.661 | C |
| NECTAR | 0.041401 | 0.13 | 256 | -0.2147 | 0.297 | C |
| ROOT_storage | 0.33967 | 0.198 | 256 | -0.0494 | 0.729 | D |
| FRUIT | 0.381499 | 0.117 | 256 | 0.1509 | 0.612 | D |
| SEEDS | 0.927302 | 0.124 | 256 | 0.6831 | 1.171 | E |
| STEMS | 1.186587 | 0.411 | 256 | 0.3768 | 1.996 | EF |
| LEAVES | 1.391765 | 0.163 | 256 | 1.07 | 1.714 | F |
| POLLEN | 1.528193 | 0.122 | 256 | 1.2876 | 1.769 | F |
| MICROBES | 1.795937 | 0.269 | 256 | 1.2658 | 2.326 | F |
| ALGAE | 1.894348 | 0.411 | 256 | 1.0845 | 2.704 | F |
| ANIMAL_TISSUE | 3.990185 | 0.0834 | 256 | 3.826 | 4.154 | G |

**__**

**X.b.ii. Arginine**

|  | Df | Sum Sq | Mean Sq | F value | Pr(>F) |
| --- | --- | --- | --- | --- | --- |
| Tissue | 12 | 679.51 | 56.626 | 51.337 | < 2.2e-16 *** |

| Tissue | emmean | SE | df | lower.CL | upper.CL | .group |
| --- | --- | --- | --- | --- | --- | --- |
| XYLEM | 0.00191 | 0.429 | 275 | -0.8422 | 0.846 | A |
| PHLOEM | 0.00586 | 0.397 | 275 | -0.7756 | 0.787 | B |
| WOOD | 0.01614 | 0.47 | 275 | -0.9085 | 0.941 | B |
| NECTAR | 0.08786 | 0.192 | 275 | -0.2896 | 0.465 | C |
| FRUIT | 0.39118 | 0.17 | 275 | 0.0558 | 0.727 | D |
| ROOT_storage | 0.63316 | 0.291 | 275 | 0.0597 | 1.207 | E |
| ALGAE | 0.90442 | 0.606 | 275 | -0.2893 | 2.098 | EF |
| STEMS | 1.0323 | 0.606 | 275 | -0.1614 | 2.226 | EF |
| LEAVES | 1.45086 | 0.224 | 275 | 1.0101 | 1.892 | F |
| SEEDS | 1.63342 | 0.18 | 275 | 1.2788 | 1.988 | F |
| POLLEN | 1.73369 | 0.18 | 275 | 1.3791 | 2.088 | F |
| MICROBES | 1.89946 | 0.271 | 275 | 1.3656 | 2.433 | F |
| ANIMAL_TISSUE | 4.15622 | 0.119 | 275 | 3.9221 | 4.39 | G |

**__**

**X.b.iii. Asparagine**

|  | Df | Sum Sq | Mean Sq | F value | Pr(>F) |
| --- | --- | --- | --- | --- | --- |
| Tissue | 2 | .3044 | .15219 | 1.4415 | .2486 |

| Tissue | emmean | SE | df | lower.CL | upper.CL | .group |
| --- | --- | --- | --- | --- | --- | --- |
| Xylem | .00251 | 1.330 | 40 | -.2656 | .271 | A |
| Phloem | .04617 | .1230 | 40 | -.2020 | .294 | A |
| Nectar | .20735 | .0593 | 40 | .0874 | .327 | A |

**__**

**X.b.iv. Aspartate**

|  | Df | Sum Sq | Mean Sq | F value | Pr(>F) |
| --- | --- | --- | --- | --- | --- |
| Tissue | 12 | 1503.54 | 125.295 | 96.103 | < 2.2e-16 *** |

| Tissue | emmean | SE | df | lower.CL | upper.CL | .group |
| --- | --- | --- | --- | --- | --- | --- |
| XYLEM | 0.000443 | 0.807 | 233 | -1.59 | 1.591 | A |
| PHLOEM | 0.009821 | 0.432 | 233 | -0.84 | 0.86 | A |
| WOOD | 0.063112 | 0.511 | 233 | -0.943 | 1.069 | B |
| NECTAR | 0.064645 | 0.208 | 233 | -0.346 | 0.475 | B |
| ROOT_storage | 1.071546 | 0.317 | 233 | 0.448 | 1.695 | C |
| FRUIT | 1.274884 | 0.188 | 233 | 0.905 | 1.645 | C |
| ALGAE | 1.784783 | 0.659 | 233 | 0.486 | 3.084 | CD |
| SEEDS | 2.079188 | 0.199 | 233 | 1.688 | 2.471 | D |
| LEAVES | 2.545628 | 0.262 | 233 | 2.03 | 3.062 | D |
| MICROBES | 2.58544 | 0.432 | 233 | 1.735 | 3.436 | D |
| POLLEN | 2.872225 | 0.196 | 233 | 2.486 | 3.258 | DE |
| STEMS | 4.09316 | 0.659 | 233 | 2.794 | 5.392 | E |
| ANIMAL_TISSUE | 7.186319 | 0.157 | 233 | 6.877 | 7.495 | F |

**__**

**X.b.v. Cysteine**

|  | Df | Sum Sq | Mean Sq | F value | Pr(>F) |
| --- | --- | --- | --- | --- | --- |
| Tissue | 11 | 21.5223 | 1.95657 | 64.508 | < 2.2e-16 *** |

| Tissue | emmean | SE | df | lower.CL | upper.CL | .group |
| --- | --- | --- | --- | --- | --- | --- |
| XYLEM | 7.17E-05 | 0.0871 | 239 | -0.1715 | 0.1716 | A |
| PHLOEM | 2.75E-04 | 0.123 | 239 | -0.2423 | 0.2429 | A |
| NECTAR | 9.00E-03 | 0.0318 | 239 | -0.0536 | 0.0716 | A |
| FRUIT | 7.87E-02 | 0.0299 | 239 | 0.0199 | 0.1376 | B |
| ROOT_storage | 1.43E-01 | 0.0483 | 239 | 0.0475 | 0.2378 | B |
| SEEDS | 2.43E-01 | 0.0303 | 239 | 0.183 | 0.3024 | C |
| STEMS | 2.59E-01 | 0.101 | 239 | 0.0609 | 0.4571 | C |
| MICROBES | 2.63E-01 | 0.0711 | 239 | 0.1232 | 0.4033 | C |
| LEAVES | 2.67E-01 | 0.04 | 239 | 0.1885 | 0.3459 | C |
| POLLEN | 3.96E-01 | 0.0299 | 239 | 0.3376 | 0.4553 | D |
| ALGAE | 4.46E-01 | 0.101 | 239 | 0.2483 | 0.6445 | D |
| ANIMAL_TISSUE | 7.91E-01 | 0.0208 | 239 | 0.7495 | 0.8315 | E |

**__**

**X.b.vi. Glutamine**

|  | Df | Sum Sq | Mean Sq | F value | Pr(>F) |
| --- | --- | --- | --- | --- | --- |
| Tissue | 2 | 2.4906 | 1.24532 | 3.7534 | .0523 |

| Tissue | emmean | SE | df | lower.CL | upper.CL | .group |
| --- | --- | --- | --- | --- | --- | --- |
| Phloem | .0109 | .235 | 39 | -4.86 | .486 | A |
| Xylem | .0139 | .235 | 39 | -.462 | .490 | A |
| Nectar | .5514 | .105 | 39 | .339 | .764 | A |

**__**

**X.b.vii. Glutamate**

|  | Df | Sum Sq | Mean Sq | F value | Pr(>F) |
| --- | --- | --- | --- | --- | --- |
| Tissue | 12 | 3156.0 | 262.999 | 51.494 | < 2.2e-16 *** |

| Tissue | emmean | SE | df | lower.CL | upper.CL | .group |
| --- | --- | --- | --- | --- | --- | --- |
| XYLEM | 0.00181 | 1.6 | 253 | -3.145 | 3.149 | A |
| PHLOEM | 0.01768 | 0.854 | 253 | -1.665 | 1.7 | A |
| WOOD | 0.04759 | 1.01 | 253 | -1.943 | 2.038 | A |
| NECTAR | 0.05402 | 0.413 | 253 | -0.759 | 0.867 | A |
| FRUIT | 1.40334 | 0.372 | 253 | 0.672 | 2.135 | B |
| ROOT_storage | 1.61668 | 0.627 | 253 | 0.382 | 2.851 | B |
| ALGAE | 2.03273 | 1.3 | 253 | -0.537 | 4.602 | B |
| STEMS | 3.00857 | 1.3 | 253 | 0.439 | 5.578 | BC |
| POLLEN | 3.17679 | 0.388 | 253 | 2.413 | 3.94 | C |
| LEAVES | 3.24537 | 0.518 | 253 | 2.224 | 4.266 | C |
| SEEDS | 3.49525 | 0.393 | 253 | 2.72 | 4.27 | CD |
| MICROBES | 4.51188 | 0.854 | 253 | 2.83 | 6.194 | D |
| ANIMAL_TISSUE | 9.34835 | 0.265 | 253 | 8.827 | 9.869 | E |

**__**

**X.b.viii. Glycine**

|  | Df | Sum Sq | Mean Sq | F value | Pr(>F) |
| --- | --- | --- | --- | --- | --- |
| Tissue | 12 | 501.00 | 41.750 | 86.762 | < 2.2e-16 *** |

| Tissue | emmean | SE | df | lower.CL | upper.CL | .group |
| --- | --- | --- | --- | --- | --- | --- |
| XYLEM | 3.54E-04 | 0.31 | 257 | -0.6106 | 0.611 | A |
| PHLOEM | 4.40E-03 | 0.262 | 257 | -0.5119 | 0.521 | A |
| WOOD | 2.86E-02 | 0.31 | 257 | -0.5823 | 0.639 | B |
| NECTAR | 3.12E-02 | 0.127 | 257 | -0.2182 | 0.281 | B |
| FRUIT | 3.09E-01 | 0.114 | 257 | 0.0842 | 0.533 | C |
| ROOT_storage | 3.21E-01 | 0.192 | 257 | -0.0579 | 0.7 | C |
| SEEDS | 9.06E-01 | 0.121 | 257 | 0.6685 | 1.144 | D |
| STEMS | 9.28E-01 | 0.401 | 257 | 0.1394 | 1.717 | DE |
| MICROBES | 1.17E+00 | 0.262 | 257 | 0.6506 | 1.683 | DE |
| ALGAE | 1.17E+00 | 0.401 | 257 | 0.3858 | 1.963 | DE |
| LEAVES | 1.22E+00 | 0.159 | 257 | 0.9058 | 1.533 | E |
| POLLEN | 1.37E+00 | 0.119 | 257 | 1.1329 | 1.601 | E |
| ANIMAL_TISSUE | 3.555417 | 0.0806 | 257 | 3.3966 | 3.714 | F |

**__**

**X.b.ix. Histidine**

|  | Df | Sum Sq | Mean Sq | F value | Pr(>F) |
| --- | --- | --- | --- | --- | --- |
| Tissue | 12 | 142.196 | 12.8497 | 57.005 | < 2.2e-16 *** |

| Tissue | emmean | SE | df | lower.CL | upper.CL | .group |
| --- | --- | --- | --- | --- | --- | --- |
| XYLEM | 2.89E-04 | 0.204 | 275 | -0.40111 | 0.402 | A |
| PHLOEM | 1.77E-03 | 0.172 | 275 | -0.33748 | 0.341 | B |
| WOOD | 1.39E-02 | 0.204 | 275 | -0.38751 | 0.415 | BC |
| NECTAR | 1.76E-02 | 0.0832 | 275 | -0.14629 | 0.181 | C |
| FRUIT | 1.49E-01 | 0.074 | 275 | 0.00325 | 0.294 | D |
| ROOT_storage | 1.91E-01 | 0.126 | 275 | -0.0576 | 0.44 | DE |
| ALGAE | 3.80E-01 | 0.263 | 275 | -0.13861 | 0.898 | E |
| SEEDS | 4.89E-01 | 0.0782 | 275 | 0.33498 | 0.643 | E |
| STEMS | 5.35E-01 | 0.263 | 275 | 0.0167 | 1.053 | E |
| LEAVES | 5.53E-01 | 0.0972 | 275 | 0.362 | 0.745 | E |
| POLLEN | 7.62E-01 | 0.0782 | 275 | 0.60849 | 0.916 | F |
| MICROBES | 9.90E-01 | 0.118 | 275 | 0.75817 | 1.222 | G |
| ANIMAL_TISSUE | 1.844825 | 0.0513 | 275 | 1.74384 | 1.946 | H |

**__**

**X.b.x. Isoleucine**

|  | Df | Sum Sq | Mean Sq | F value | Pr(>F) |
| --- | --- | --- | --- | --- | --- |
| Tissue | 12 | 352.34 | 29.3620 | 69.947 | < 2.2e-16 *** |

| Tissue | emmean | SE | df | lower.CL | upper.CL | .group |
| --- | --- | --- | --- | --- | --- | --- |
| XYLEM | 4.23E-04 | 0.29 | 280 | -0.5699 | 0.571 | A |
| PHLOEM | 4.32E-03 | 0.245 | 280 | -0.4777 | 0.486 | A |
| WOOD | 1.87E-02 | 0.29 | 280 | -0.5517 | 0.589 | B |
| NECTAR | 2.10E-02 | 0.118 | 280 | -0.2119 | 0.254 | B |
| FRUIT | 2.84E-01 | 0.104 | 280 | 0.0799 | 0.488 | C |
| ROOT_storage | 3.42E-01 | 0.18 | 280 | -0.0114 | 0.696 | C |
| SEEDS | 8.14E-01 | 0.111 | 280 | 0.5955 | 1.033 | D |
| ALGAE | 8.58E-01 | 0.374 | 280 | 0.1219 | 1.595 | DE |
| STEMS | 8.65E-01 | 0.374 | 280 | 0.1286 | 1.601 | DE |
| POLLEN | 1.14E+00 | 0.111 | 280 | 0.923 | 1.36 | E |
| LEAVES | 1.26E+00 | 0.135 | 280 | 0.9936 | 1.525 | E |
| MICROBES | 1.58E+00 | 0.167 | 280 | 1.2539 | 1.913 | F |
| ANIMAL_TISSUE | 2.905788 | 0.0715 | 280 | 2.7649 | 3.047 | G |

**__**

**X.b.xi. Leucine**

|  | Df | Sum Sq | Mean Sq | F value | Pr(>F) |
| --- | --- | --- | --- | --- | --- |
| Tissue | 12 | 1039.63 | 86.636 | 67.372 | < 2.2e-16 *** |

| Tissue | emmean | SE | df | lower.CL | upper.CL | .group |
| --- | --- | --- | --- | --- | --- | --- |
| XYLEM | 2.69E-04 | 0.507 | 280 | -0.998 | 0.999 | A |
| PHLOEM | 4.85E-03 | 0.429 | 280 | -0.839 | 0.849 | A |
| NECTAR | 3.22E-02 | 0.207 | 280 | -0.375 | 0.44 | AB |
| WOOD | 3.92E-02 | 0.507 | 280 | -0.959 | 1.037 | B |
| FRUIT | 4.45E-01 | 0.182 | 280 | 0.088 | 0.803 | C |
| ROOT_storage | 4.62E-01 | 0.315 | 280 | -0.158 | 1.081 | C |
| STEMS | 1.32E+00 | 0.655 | 280 | 0.029 | 2.607 | D |
| SEEDS | 1.43E+00 | 0.194 | 280 | 1.051 | 1.816 | D |
| ALGAE | 1.69E+00 | 0.655 | 280 | 0.403 | 2.981 | D |
| LEAVES | 1.91E+00 | 0.236 | 280 | 1.449 | 2.38 | DE |
| POLLEN | 1.98E+00 | 0.194 | 280 | 1.601 | 2.366 | E |
| MICROBES | 2.56E+00 | 0.293 | 280 | 1.985 | 3.138 | E |
| ANIMAL_TISSUE | 4.970918 | 0.125 | 280 | 4.724 | 5.217 | F |

**__**

**X.b.xii. Lysine**

|  | Df | Sum Sq | Mean Sq | F value | Pr(>F) |
| --- | --- | --- | --- | --- | --- |
| Tissue | 12 | 1103.36 | 91.946 | 50.854 | < 2.2e-16 *** |

| Tissue | emmean | SE | df | lower.CL | upper.CL | .group |
| --- | --- | --- | --- | --- | --- | --- |
| XYLEM | 4.89E-04 | 0.602 | 280 | -1.1844 | 1.185 | A |
| PHLOEM | 5.15E-03 | 0.509 | 280 | -0.9963 | 1.007 | AB |
| WOOD | 2.03E-02 | 0.602 | 280 | -1.1645 | 1.205 | B |
| NECTAR | 2.18E-02 | 0.246 | 280 | -0.462 | 0.505 | B |
| FRUIT | 3.66E-01 | 0.216 | 280 | -0.0579 | 0.791 | C |
| ROOT_storage | 4.50E-01 | 0.373 | 280 | -0.2844 | 1.185 | C |
| ALGAE | 8.29E-01 | 0.777 | 280 | -0.7011 | 2.358 | D |
| SEEDS | 1.01E+00 | 0.231 | 280 | 0.5569 | 1.466 | DE |
| LEAVES | 1.40E+00 | 0.281 | 280 | 0.8431 | 1.948 | DE |
| STEMS | 1.42E+00 | 0.777 | 280 | -0.1137 | 2.946 | DE |
| POLLEN | 1.77E+00 | 0.231 | 280 | 1.3171 | 2.226 | E |
| MICROBES | 2.20E+00 | 0.348 | 280 | 1.5139 | 2.882 | E |
| ANIMAL_TISSUE | 4.992819 | 0.149 | 280 | 4.7002 | 5.285 | F |

**__**

**X.b.xiii. Methionine**

|  | Df | Sum Sq | Mean Sq | F value | Pr(>F) |
| --- | --- | --- | --- | --- | --- |
| Tissue | 12 | 128.439 | 10.7033 | 72.935 | < 2.2e-16 *** |

| Tissue | emmean | SE | df | lower.CL | upper.CL | .group |
| --- | --- | --- | --- | --- | --- | --- |
| XYLEM | 1.95E-04 | 0.171 | 276 | -0.3371 | 0.337 | A |
| PHLOEM | 4.66E-03 | 0.145 | 276 | -0.2804 | 0.29 | AB |
| NECTAR | 6.15E-03 | 0.0699 | 276 | -0.1315 | 0.144 | AB |
| WOOD | 7.16E-03 | 0.171 | 276 | -0.3301 | 0.344 | B |
| FRUIT | 8.96E-02 | 0.0613 | 276 | -0.0311 | 0.21 | C |
| ROOT_storage | 1.08E-01 | 0.106 | 276 | -0.1013 | 0.317 | C |
| SEEDS | 2.82E-01 | 0.0657 | 276 | 0.1528 | 0.412 | D |
| STEMS | 2.91E-01 | 0.221 | 276 | -0.1441 | 0.727 | DE |
| LEAVES | 3.50E-01 | 0.0817 | 276 | 0.1891 | 0.511 | DE |
| ALGAE | 4.73E-01 | 0.221 | 276 | 0.0371 | 0.908 | DE |
| MICROBES | 5.34E-01 | 0.0989 | 276 | 0.3396 | 0.729 | EF |
| POLLEN | 6.32E-01 | 0.0657 | 276 | 0.5028 | 0.762 | F |
| ANIMAL_TISSUE | 1.690742 | 0.0431 | 276 | 1.6059 | 1.776 | G |

**__**

**X.b.xiv. Phenylalanine**

|  | Df | Sum Sq | Mean Sq | F value | Pr(>F) |
| --- | --- | --- | --- | --- | --- |
| Tissue | 12 | 291.618 | 24.3015 | 67.706 | < 2.2e-16 *** |

| Tissue | emmean | SE | df | lower.CL | upper.CL | .group |
| --- | --- | --- | --- | --- | --- | --- |
| XYLEM | 3.47E-04 | 0.268 | 278 | -0.5271 | 0.528 | A |
| PHLOEM | 3.28E-03 | 0.226 | 278 | -0.4425 | 0.449 | B |
| WOOD | 2.49E-02 | 0.268 | 278 | -0.5025 | 0.552 | C |
| NECTAR | 1.72E-01 | 0.109 | 278 | -0.0434 | 0.387 | CD |
| FRUIT | 2.78E-01 | 0.0959 | 278 | 0.0891 | 0.467 | D |
| ROOT_storage | 3.04E-01 | 0.166 | 278 | -0.0231 | 0.631 | D |
| STEMS | 8.00E-01 | 0.346 | 278 | 0.1193 | 1.481 | E |
| ALGAE | 8.71E-01 | 0.346 | 278 | 0.1903 | 1.552 | EF |
| SEEDS | 9.25E-01 | 0.103 | 278 | 0.7231 | 1.128 | EF |
| POLLEN | 1.19E+00 | 0.103 | 278 | 0.9882 | 1.393 | F |
| LEAVES | 1.22E+00 | 0.128 | 278 | 0.9683 | 1.471 | F |
| MICROBES | 1.39E+00 | 0.155 | 278 | 1.0835 | 1.693 | F |
| ANIMAL_TISSUE | 2.728392 | 0.0666 | 278 | 2.5974 | 2.859 | G |

**__**

**X.b.xv. Proline**

|  | Df | Sum Sq | Mean Sq | F value | Pr(>F) |
| --- | --- | --- | --- | --- | --- |
| Tissue | 12 | 436.70 | 36.391 | 68.569 | < 2.2e-16 *** |

| Tissue | emmean | SE | df | lower.CL | upper.CL | .group |
| --- | --- | --- | --- | --- | --- | --- |
| XYLEM | 1.23E-03 | 0.326 | 247 | -0.64046 | 0.643 | A |
| PHLOEM | 9.83E-03 | 0.515 | 247 | -1.00479 | 1.024 | AB |
| WOOD | 3.58E-02 | 0.326 | 247 | -0.60594 | 0.677 | B |
| NECTAR | 1.67E-01 | 0.133 | 247 | -0.09538 | 0.429 | B |
| ROOT_storage | 3.05E-01 | 0.202 | 247 | -0.09301 | 0.703 | C |
| FRUIT | 3.59E-01 | 0.12 | 247 | 0.12359 | 0.595 | C |
| STEMS | 8.33E-01 | 0.421 | 247 | 0.00413 | 1.661 | D |
| ALGAE | 8.43E-01 | 0.421 | 247 | 0.01493 | 1.672 | D |
| MICROBES | 9.33E-01 | 0.275 | 247 | 0.39033 | 1.475 | D |
| SEEDS | 9.67E-01 | 0.127 | 247 | 0.71695 | 1.217 | D |
| LEAVES | 1.24E+00 | 0.167 | 247 | 0.90925 | 1.568 | D |
| ANIMAL_TISSUE | 2.94E+00 | 0.0877 | 247 | 2.76994 | 3.115 | E |
| POLLEN | 3.47586 | 0.125 | 247 | 3.22978 | 3.722 | F |

**__**

**X.b.xvi. Serine**

|  | Df | Sum Sq | Mean Sq | F value | Pr(>F) |
| --- | --- | --- | --- | --- | --- |
| Tissue | 12 | 280.854 | 23.4045 | 97.065 | < 2.2e-16 *** |

| Tissue | emmean | SE | df | lower.CL | upper.CL | .group |
| --- | --- | --- | --- | --- | --- | --- |
| XYLEM | 4.23E-04 | 0.22 | 256 | -0.432 | 0.433 | A |
| PHLOEM | 8.03E-03 | 0.186 | 256 | -0.3575 | 0.374 | B |
| WOOD | 2.52E-02 | 0.22 | 256 | -0.4073 | 0.458 | C |
| NECTAR | 7.33E-02 | 0.0897 | 256 | -0.1032 | 0.25 | C |
| ROOT_storage | 3.26E-01 | 0.136 | 256 | 0.0579 | 0.594 | D |
| FRUIT | 3.62E-01 | 0.0807 | 256 | 0.2032 | 0.521 | D |
| ALGAE | 9.72E-01 | 0.284 | 256 | 0.4137 | 1.53 | E |
| SEEDS | 9.83E-01 | 0.0855 | 256 | 0.8146 | 1.151 | EF |
| LEAVES | 9.99E-01 | 0.113 | 256 | 0.7774 | 1.221 | EF |
| STEMS | 1.03E+00 | 0.284 | 256 | 0.4669 | 1.583 | EF |
| MICROBES | 1.20E+00 | 0.186 | 256 | 0.8314 | 1.562 | EF |
| POLLEN | 1.52E+00 | 0.0842 | 256 | 1.3537 | 1.685 | F |
| ANIMAL_TISSUE | 2.760502 | 0.0575 | 256 | 2.6473 | 2.874 | G |

**__**

**X.b.xvii. Threonine**

|  | Df | Sum Sq | Mean Sq | F value | Pr(>F) |
| --- | --- | --- | --- | --- | --- |
| Tissue | 12 | 307.92 | 25.660 | 69.922 | < 2.2e-16 *** |

| Tissue | emmean | SE | df | lower.CL | upper.CL | .group |
| --- | --- | --- | --- | --- | --- | --- |
| XYLEM | 4.60E-04 | 0.271 | 280 | -0.5328 | 0.534 | A |
| PHLOEM | 4.34E-03 | 0.229 | 280 | -0.4464 | 0.455 | B |
| NECTAR | 1.76E-02 | 0.111 | 280 | -0.2001 | 0.235 | BC |
| WOOD | 3.27E-02 | 0.271 | 280 | -0.5006 | 0.566 | C |
| FRUIT | 2.63E-01 | 0.097 | 280 | 0.072 | 0.454 | D |
| ROOT_storage | 3.92E-01 | 0.168 | 280 | 0.0609 | 0.722 | D |
| SEEDS | 6.96E-01 | 0.104 | 280 | 0.4912 | 0.9 | E |
| STEMS | 8.80E-01 | 0.35 | 280 | 0.1916 | 1.569 | EF |
| ALGAE | 8.90E-01 | 0.35 | 280 | 0.2017 | 1.579 | EF |
| LEAVES | 1.07E+00 | 0.126 | 280 | 0.8204 | 1.318 | F |
| POLLEN | 1.20E+00 | 0.104 | 280 | 0.9989 | 1.408 | F |
| MICROBES | 1.60E+00 | 0.156 | 280 | 1.2899 | 1.906 | G |
| ANIMAL_TISSUE | 2.71137 | 0.0669 | 280 | 2.5797 | 2.843 | H |

**__**

**X.b.xviii. Tryptophan**

|  | Df | Sum Sq | Mean Sq | F value | Pr(>F) |
| --- | --- | --- | --- | --- | --- |
| Tissue | 9 | 22.2046 | 2.46717 | 55.305 | < 2.2e-16 *** |

| Tissue | emmean | SE | df | lower.CL | upper.CL | .group |
| --- | --- | --- | --- | --- | --- | --- |
| PHLOEM | 5.41E-04 | 0.0945 | 220 | -0.185615 | 0.1867 | A |
| NECTAR | 1.52E-03 | 0.0386 | 220 | -0.074475 | 0.0775 | A |
| FRUIT | 9.12E-02 | 0.0343 | 220 | 0.023664 | 0.1587 | B |
| ROOT_storage | 9.22E-02 | 0.061 | 220 | -0.027947 | 0.2124 | B |
| SEEDS | 2.18E-01 | 0.0368 | 220 | 0.145517 | 0.2904 | C |
| ALGAE | 2.41E-01 | 0.122 | 220 | 0.000519 | 0.4812 | CD |
| STEMS | 2.92E-01 | 0.122 | 220 | 0.05141 | 0.5321 | CD |
| LEAVES | 3.35E-01 | 0.045 | 220 | 0.246547 | 0.424 | D |
| MICROBES | 4.12E-01 | 0.0564 | 220 | 0.300769 | 0.5233 | D |
| ANIMAL_TISSUE | 7.96E-01 | 0.0252 | 220 | 0.746541 | 0.846 | E |

**__**

**X.b.xix. Tyrosine**

|  | Df | Sum Sq | Mean Sq | F value | Pr(>F) |
| --- | --- | --- | --- | --- | --- |
| Tissue | 12 | 304.684 | 23.290 | 78.113 | < 2.2e-16 *** |

| Tissue | emmean | SE | df | lower.CL | upper.CL | .group |
| --- | --- | --- | --- | --- | --- | --- |
| XYLEM | 9.06E-05 | 0.255 | 263 | -0.502 | 0.502 | A |
| PHLOEM | 2.68E-03 | 0.215 | 263 | -0.4216 | 0.427 | B |
| WOOD | 1.08E-02 | 0.255 | 263 | -0.4913 | 0.513 | C |
| NECTAR | 4.66E-02 | 0.104 | 263 | -0.1584 | 0.252 | C |
| ROOT_storage | 1.87E-01 | 0.158 | 263 | -0.1245 | 0.498 | D |
| FRUIT | 2.09E-01 | 0.0925 | 263 | 0.0273 | 0.392 | D |
| STEMS | 5.41E-01 | 0.329 | 263 | -0.1075 | 1.189 | E |
| SEEDS | 5.93E-01 | 0.0992 | 263 | 0.3973 | 0.788 | E |
| ALGAE | 6.93E-01 | 0.329 | 263 | 0.0451 | 1.341 | EF |
| MICROBES | 8.28E-01 | 0.215 | 263 | 0.4036 | 1.252 | EF |
| POLLEN | 8.43E-01 | 0.0978 | 263 | 0.6505 | 1.036 | F |
| LEAVES | 8.78E-01 | 0.124 | 263 | 0.6326 | 1.122 | F |
| ANIMAL_TISSUE | 2.70E+00 | 0.065 | 263 | 2.5696 | 2.825 | G |

**__**

**X.b.xx. Valine**

|  | Df | Sum Sq | Mean Sq | F value | Pr(>F) |
| --- | --- | --- | --- | --- | --- |
| Tissue | 12 | 462.80 | 38.567 | 78.357 | < 2.2e-16 *** |

| Tissue | emmean | SE | df | lower.CL | upper.CL | .group |
| --- | --- | --- | --- | --- | --- | --- |
| XYLEM | 4.58E-04 | 0.286 | 280 | -0.56334 | 0.564 | A |
| PHLOEM | 3.87E-03 | 0.286 | 280 | -0.55993 | 0.568 | B |
| WOOD | 2.94E-02 | 0.314 | 280 | -0.5882 | 0.647 | C |
| NECTAR | 3.66E-02 | 0.128 | 280 | -0.21554 | 0.289 | C |
| FRUIT | 3.39E-01 | 0.112 | 280 | 0.11768 | 0.56 | D |
| ROOT_storage | 3.86E-01 | 0.195 | 280 | 0.00294 | 0.769 | D |
| SEEDS | 9.84E-01 | 0.12 | 280 | 0.74763 | 1.221 | E |
| STEMS | 1.25E+00 | 0.405 | 280 | 0.45515 | 2.05 | EF |
| ALGAE | 1.37E+00 | 0.405 | 280 | 0.57477 | 2.169 | EF |
| LEAVES | 1.39E+00 | 0.146 | 280 | 1.10516 | 1.681 | F |
| POLLEN | 1.48E+00 | 0.12 | 280 | 1.24525 | 1.719 | F |
| MICROBES | 2.13E+00 | 0.181 | 280 | 1.77128 | 2.484 | G |
| ANIMAL_TISSUE | 3.34E+00 | 0.0775 | 280 | 3.18526 | 3.49 | H |

**XI. SUPPORTING INFORMATION DISCUSSION – An inordinate fondness for beetles?**

*XI.a. Diversity sustained by plausible symbiotic key innovations in curculionids*

With over 300 million years of symbiotic history in some hemipteran lineages (Li et al. 2017a), the reach of microbes’ nutritional services in the navigation of herbivory is staggering. In examining the >42 other independent origins of symbiosis, several other potentially ancient impacts, reaching thousands of species, warrant further discussion.

First are weevils, bark beetles, and ambrosia beetles from the Curculionidae. Common occurrence of bacteriocyte-confined symbionts – primarily *Nardonella* – assisting with cuticle building has been reported in some taxa (Salem & Kaltenpoth 2022). An unpublished pre-print suggests *Nardonella*-beetle associations evolved in the common ancestor of the Curculionidae and Brentidae (Zhang et al. 2017). The very sparse distributions of this symbiont in the Brentidae (3 out of 9 species), and in earlier-branching (5 out of 28 species) vs. crown group Curculionidae clades in that study suggests this single ancient origin hypothesis is tentative. High symbiont prevalence in some curculionid subfamilies, and previously documented cospeciation do still argue for a longstanding symbiosis, with one estimate placing its dawn at 125 MYA (Lefèvre et al. 2004; Conord et al. 2008). The retention of aromatic amino acid biosynthesis across *Nardonella* hosts, and in seed-feeding hosts with supplanting symbionts, argues for the continued importance of obligate symbiosis in weevils’ cuticle development (Toju et al. 2013; Vigneron et al. 2014).

While this symbiosis is not universal across the 76,761 curculionids, it is undoubtedly integral for many thousands of species. Missing, however, from this equation is a clear indication of the environmental pressure favoring the evolved/retained function – i.e. have those with symbiont-supported cuticles moved into a novel, inhospitable niche? To begin, the diverse diets among those with cuticle-building symbionts across beetles and beyond (e.g. predation, pollen, ominivory, stem-feeding, leaf-chewing) are often rich in aromatic amino acids (Table S85 – [Zenodo](https://doi.org/10.5281/zenodo.19607748) repository). So any consistent, novel niche is not yet clearly defined through diet. And while thicker cuticles may be integral for withstanding predation or desiccation (Engl et al. 2018; Kanyile et al. 2022), there is no indication that these pressures have been higher for hosts with cuticle-building symbioses. Defining the symbiont-driven cuticle-building service as a key innovation will, thus, require future study.

Beyond these relationships lie those between fungi and the ambrosia/bark beetles – also from the Curculionidae. Hailing from the subfamilies Scolytinae and Platypodinae, which possess a combined 7275 species (https://www.barkbeetles.info/amer_classification.php), these wood-exploiting beetles surely benefit from the nitrogen-concentrating, amino acid synthesizing, and digestive abilities of their fungi. But further work is needed to comprehend both the sustained diversity and the factors favoring origins/retention of their mycangial symbioses (Ayres et al. 2000; Hulcr & Stelinski 2017; Six & Klepzig 2021).

*XI.b. Diversity-sustaining symbiotic key innovations in other beetles?*

The highly diverse Chrysomelidae is estimated to have spawned three origins of obligate symbiosis (Kirsch et al. 2025). Symbionts are found in lineages that have lost host-encoded pectinases, one class of plant cell wall degrading enzymes (PCWDEs). Across one origin in the ancestor of the Cassidinae, Eumolpinae, and Spilopyrinae clade, symbiont-encoded pectinase function is mostly conserved amidst these leaf- and root-chewing taxa. Symbionts of the latter two subfamilies also encode aromatic amino acid biosynthesis pathways, suggestive of cuticle building. In a separately derived symbiosis within the stem gall-inducing Sagrinae, symbionts encode this latter function plus biosynthetic capacities for the synthesis of some B-vitamins and essential amino acids. Within the third symbiotic lineage – i.e. the reed beetle Donaciinae subfamily – essential amino acid synthesis, and pectinase activity of an obligate symbiont are hypothesized to support larval phloem feeding and adult leaf chewing (Reis et al. 2020).

Symbiosis seems non-ubiquitous within most of these subfamilies (e.g. Mann & Crowson 1983; Kirsch et al. 2025), and it is not clear if obligate symbionts exist in other taxa within the chrysomelid family. Just how many of the 13,560 described chrysomelids are sustained through symbiosis, thus, remains unknown (see main manuscript for discussion of potentially higher species richness). Also uncertain are the drivers of host-encoded pectinase loss. Are there benefits to outsourcing fiber digestion to symbionts? Or were losses, unfolding for other reasons, most readily fixed by symbiosis? Furthermore, beyond the phloem-feeding reed beetles (Kleinschmidt and Kölsch 2011), just how have symbionts altered habitat or feeding niche?

The absence of certain PCWDEs from beetle genomes has been noted as a correlate of symbiosis beyond the chrysomelids (McKenna et al. 2019; Kirsch et al. 2025). Haling from a lineage with some symbiont reliance, and without many laterally transferred PCWDEs, are 36,127 accepted species from the Scarabaeidae. Using a recently published phylogeny of the Scarabaeidae, root-, leaf-, rotting wood-, and detritus-feeding scarabs formed a sister relationship with those consuming dung (Dietz et al. 2023). Studies of symbionts in this group have elucidated function, transmission, and conservation (Bayon & Mathelin 1980; Andert et al. 2010; Huang et al. 2010; Estes et al. 2013; Schwab et al. 2016; Parker et al. 2021). But symbioses with such fermentative gut microbes have waxed and waned (e.g. Ebert et al. 2021). The diversity of the consumed diets, and the lack of obligate symbioses in many root-, rotting wood, leaf-, and detritus-feeders from other insect taxa (Table S1; Table S3 – [Zenodo](https://doi.org/10.5281/zenodo.19607748) repository) makes it difficult to know whether symbionts opened access to an inhospitable niche, or if some aspect of beetle biology – beyond a lack of genomic PCWDEs – made them more pre-disposed toward quick-fix symbiotic solutions.

Providing an exception to the enrichment of symbiosis in beetles without PCWDEs are the Cerambycidae and the above-described Curculionidae. Cerambycids hail from the hyper-diverse Phytophaga – a group with rapid net diversification rates that also includes chrysomelids and curulionids (McKenna et al. 2019). Despite some conflicting claims, symbiotic integration in cerambycids seems to include sporadic instances of mycetocytes/bacteriocytes across the Cerambycinae and Lamiinae. Mycetocyte symbioses are prevalent in the basal grade (Song et al. 2025) of Lepturinae, Necydalinae, and Spondylidinae, suggesting an ancestral reliance on obligate symbionts. Also common are well-studied symbioses with populous extracellular gut microbes in the Lamiinae (Linsley 1959; Geib et al. 2008; Calderon & Berkov 2010; Grünwald et al. 2010; Ayayee et al. 2014; Scully et al. 2014; Kishigami et al. 2023; Sasakura et al. 2024).

While distinct types of integration suggest separate origins – or at least major reinventions – of symbiosis, it is not clear how many cerambycids lack obligate symbionts. Intriguingly, one study revealed mycetocyte loss in lepturine cerambycids transitioning to inner bark and root feeding, though it did not determine the potential for reliance on extracellular gut microbes (Sasakura et al. 2024). The ancestral feeding habit of mycetocyte-harboring lepturines is argued to be brown-rot affected sapwood. Would such microbe-conditioned food leave a void necessitating nutritional symbionts? While this question remains unanswered, larval consumption of wood at varying life/decay stages across the cerambycids (Linsley 1959) suggests occasional need for digestion, nitrogen fixation or recycling, and nutrient provisioning as demonstrated for some of their extracellular gut symbionts (Geib et al. 2008; Ayayee et al. 2014; Scully et al. 2014). Separately, sustained fitness of symbiont-cured lepturines on a nutritious artificial diet helps to suggest nutritive roles of mycetocyte-confined symbionts (Sasakura et al. 2024). With 160 million years of evolution, and the likelihood of an ancestral mycetocyte symbiosis, nutritional symbiosis may comprise a far-reaching series of symbiotic key innovations for many of the world’s 35,288 cerambycids.

*XI.c. Diversity-sustaining nutritional services in small subsets of the world’s palinivores and detritivores – outlines of symbiont-based key innovations?*

We end our discussion of symbiont-supported diversity in insects with plant- and plant-adjacent diets through discussion of gregarious, subsocial, and eusocial insects, where symbioses are not always expected. Certainly diverse amongst these are symbiotic cockroaches and termites (order Blattodea), with over 8000 species (Table S3 – [Zenodo](https://doi.org/10.5281/zenodo.19607748) repository). Hosting several types of symbioses reinvented since their common ancestor >263 MYA (Bandi et al. 1995; Ohkuma et al. 2009; Andert et al. 2008; Dietrich et al. 2014; Legendre et al. 2015; Mikaelyan et al. 2015), obligate microbiomes in this lineage seem to support a range of diets including detritus (not typically symbiotic), wood in varying stages of decay, soil, and even fresh plant tissue (Collins 1983).

While there are phases where detritus and wood are comprised by substantial fractions of nutritious microbial biomass and microbe-conditioned tissue (Cummins 1974; France 2011; Harrop-Archibald et al. 2016), detrital nitrogen content may evolve into more recalcitrant, humic forms (Rice 1982; Knicker & Lüdemann 1995; Ji & Brune 2005; Allison 2006). So one reason detritivores and soil feeders vary in their reliance on symbiosis could stem from variation in the stage and form in which detritus and, subsequent, soil organic matter are consumed. Another could stem from varying gut physiologies (Šustr et al. 2014).

A further consideration might be that derived physiologies or behaviors made it easier to evolve and maintain highly integrated symbioses in a subset of the world’s insects. In detritivorous cockroaches, coprophagy was proposed as a pre-condition to the evolution of more specialized, mutualistic symbioses, which gave way to those in eusocial termites (Nalepa et al. 2001). By eating feces, cockroaches re-consume microbes that have passaged through the gut. This likely promotes partner fidelity, helping to better align the interests of hosts and their microbes (Foster & Wenseleers 2006; Salem et al. 2015). Did this lower the barrier for evolving more integrated, mutualistic symbiosis? More specifically, if selfish microbe ‘behaviors’ were more readily weeded out, is it possible that lowered costs lessened thresholds for nutrient scarcity required to favor obligate symbiosis?

Another important question is whether the relationship with bacteriocyte-housed *Blattabacterium* started as a coprophagy-sustained/jump-started symbiosis with extracellular gut bacteria. After this origin, there is no clear place for coprophagy in supporting a sustained bacteriocyte symbiosis. Indeed, hemipteran shifts to nutritious diets (e.g. mesophyll) have repeatedly enabled loss of bacteriocyte-contained symbioses (e.g. Phylloxera, Typhlocybinae). The maintenance of *Blattabacterium* despite consistent histories of detritivory (Bandi et al. 1995; Kinjo et al. 2018) suggests nutritive and physiological mysteries have yet to be uncovered.

While coprophagous behaviors are seen in gregarious, non-social insects, some cockroaches have evolved subsociality, while their descendant termites are fully eusocial. Prior hypotheses have linked the origins of both symbiosis and eusociality (Nalepa 2020). And, indeed, social living could align the interests of hosts and microbes through a range of extending transmission mechanisms (Onchuru et al. 2018; Rose et al. 2023). Did eusociality separately pave the path for the evolution of obligate nutritional symbioses in the ants and bees? Did it help to better integrate symbioses between extracellular microbes and the wood roaches and termites? And might this explain the retention of socially transmitted gut symbioses in termites that have moved away from wood feeding? Or might, again, misunderstood aspects of diet or cost-minimizing physiologies, prove more influential?

Like the above-described curious case of detritus, it is surprising that some symbiont-reliant, eusocial hymenopterans feed extensively on pollen. Precedent for shortfalls in one or two specific amino acids in the pollen of some plants (Weiner et al. 2010; Génissel et al. 2002) would seem not to explain provisioning of up to seven essential amino acids by ancient, specialized extracellular gut microbes of corbiculate bees and ants (Hu et al. 2018; Zheng et al. 2019). Endogenous means for pollen digestion in insects (e.g. González et al. 2022), raise further questions on the needs for symbionts’ pollen wall-digesting services (Kešnerová et al. 2017; but see Human & Nicolson 2003). Derived eusociality in the symbiont-reliant corbiculate bee clade (Meliponini, Apini, Bombini; Mee & Baribeau 2023), returns us further to the question of whether social/gregarious behaviors help to lower the bar in evolving reliance on symbiosis.

While the diversity of corbiculate bees approaches only 698 species (Cameron et al. 2007; Nogueira 2023), an even larger group of eusocial insects with occasional pollen feeding comes from across the Hymenoptera, within the ants (Cook & Davidson 2006; Cembrowski et al. 2015). In this family (Formicidae), nitrogen-recycling symbioses have been characterized in groups feeding low on the food chain (Russell et al. 2017). The Camponotini (carpenter ants) makes up the greatest diversity among these taxa with over 1850 species. *Dolichoderus* ants and those from the *Cephalotes/Procryptocerus* clade round out this guild, with an additional >290 species. With imprecise knowledge on nutritive content, correlations between diet (i.e. insect honeydew, plant extrafloral nectar, and pollen) vs. nutritional symbioses (Billen & Buschinger 2000; Davidson et al. 2003; Cook & Davidson 2006; Russell et al. 2017; Hansen et al. 2020) remain harder to explain in these ants than in a range of other insects with more precisely defined, nutrient-poor diets. Furthermore, with ominivory being frequently seen in symbiont-reliant carpenter ants, one does wonder if the rules for evolving and maintaining obligate symbiosis – i.e. the needs of the basic insect body plan, and the evolvability of stable obligate symbiosis – go out the window for social insects.

**XII. COMPARISONS TO CORNWALLIS ET AL. 2023**

**Sample sizes for B-vitamin measures across plant tissues and other insect foods in the present study vs. that of Cornwallis et al. 2023.** Our sample sizes are generally larger for plant tissues, but also for some non-plant foods. Blue to white to red shading is used as a heatmap to illustrate sample size. Note that our sample sizes refer to the number of species with data for the given tissue. Those of Cornwallis et al. indicate total number of measurements across the tissue, regardless of species identity (i.e. includes some resampling of conspecifics). *Data for detritus from Cornwallis et al. lacks natural leaf-litter substrate typically defined as such (e.g. present study, and literature beyond).

**Sample sizes for nitrogen/protein measures across plant tissues and other insect foods in the present study vs. that of Cornwallis et al. 2023.** Note that Cornwallis et al. focused on total %protein, whereas our study used measures of total nitrogen (as a % of total dry weight). Our sample sizes are generally larger for plant tissues, but for non-plant based foods, we used data ranges from one or more papers from each category. In those cases, we indicate the availability of %nitrogen estimates with an “x”. We use blue to white to red shading as a heatmap for illustrating sample size. Note that our sample sizes refer to the number of species with data for the given tissue. Those of Cornwallis et al. indicate total number of measurements across the tissue, regardless of species identity (i.e. includes some resampling of conspecifics).

**XIII. REFERENCES CITED**

Adams, E. S. (1990). Interaction between the ants *Zacryptocerus maculatus* and *Azteca trigona*: interspecific parasitization of information. *Biotropica*, *22*(2), 200–206.

Allison, S. D. (2006). Brown ground: a soil carbon analogue for the green world hypothesis? *The American Naturalist*, *167*(5), 619–627.

Alonso-Pernas, P., Bartram, S., Arias-Cordero, E. M., Novoselov, A. L., Halty-deLeon, L., Shao, Y., & Boland, W. (2017). *In vivo* isotopic labeling of symbiotic bacteria involved in cellulose degradation and nitrogen recycling within the gut of the Forest cockchafer (*Melolontha hippocastani*). *Frontiers in Microbiology*, *8*, 1970.

Anbutsu, H., Moriyama, M., Nikoh, N., Hosokawa, T., Futahashi, R., Tanahashi, M., Meng, X.-Y., Kuriwada, T., Mori, N., Oshima, K., Hattori, M., Fujie, M., Satoh, N., Maeda, T., Shigenobu, S., Koga, R., & Fukatsu, T. (2017). Small genome symbiont underlies cuticle hardness in beetles. *Proceedings of the National Academy of Sciences of the United States of America*, *114*(40), E8382–E8391.

Andert, J., Geissinger, O., & Brune, A. (2008). Peptidic soil components are a major dietary resource for the humivorous larvae of *Pachnoda* spp. (Coleoptera: Scarabaeidae). *Journal of Insect Physiology*, *54*(1), 105–113.

Andert, J., Marten, A., Brandl, R., & Brune, A. (2010). Inter- and intraspecific comparison of the bacterial assemblages in the hindgut of humivorous scarab beetle larvae (*Pachnoda* spp.). *FEMS Microbiology Ecology*, *74*(2), 439–449.

Andlar, M., Rezić, T., Marđetko, N., Kracher, D., Ludwig, R., & Šantek, B. (2018). Lignocellulose degradation: an overview of fungi and fungal enzymes involved in lignocellulose degradation. *Engineering in Life Sciences*, *18*(11), 768–778.

An, Y., Braga, M. P., Garcia, S. L., Grudzinska-Sterno, M., & Hambäck, P. A. (2023). Host phylogeny structures the gut bacterial community within *Galerucella* leaf beetles. *Microbial Ecology*, *86*(4), 2477–2487.

Ayayee, P., Rosa, C., Ferry, J. G., Felton, G., Saunders, M., & Hoover, K. (2014). Gut microbes contribute to nitrogen provisioning in a wood-feeding cerambycid. *Environmental Entomology*, *43*(4), 903–912.

Ayres, M. P., Wilkens, R. T., Ruel, J. J., Lombardero, M. J., & Vallery, E. (2000). Nitrogen budgets of phloem-feeding bark beetles with and without symbiotic fungi. *Ecology*, *81*(8), 2198–2210.

Bandi, C., Sironi, M., Damiani, G., Magrassi, L., Nalepa, C. A., Laudani, U., & Sacchi, L. (1995). The establishment of intracellular symbiosis in an ancestor of cockroaches and termites. [*Proceedings of the Royal Society of London, Series B, Biological Sciences*,](http://paperpile.com/b/C5m4ez/MNxJ) *259*(1356), 293–299.

Bateman, M. A. (1972). The ecology of fruit flies. *Annual Review of Entomology*, *17*(1), 493–518.

Bayon, C., & Mathelin, J. (1980). Carbohydrate fermentation and by-product absorption studied with labelled cellulose in *Oryctes nasicornis* larvae (Coleoptera:Scarabaeidae). *Journal of Insect Physiology*, *26*(12), 833–840.

Béchade, B., Cabuslay, C. S., Hu, Y., Mendonca, C. M., Hassanpour, B., Lin, J. Y., Su, Y., Fiers, V. J., Anandarajan, D., Lu, R., Olson, C. J., Duplais, C., Rosen, G. L., Moreau, C. S., Aristilde, L., Wertz, J. T., & Russell, J. A. (2023). Physiological and evolutionary contexts of a new symbiotic species from the nitrogen-recycling gut community of turtle ants. *The ISME Journal*, *17*(10), 1751–1764.

Beilin, L. J., Knight, G. J., Munro-Faure, A. D., & Anderson, J. (1966). The sodium, potassium, and water contents of red blood cells of healthy human adults. *The Journal of Clinical Investigation*, *45*(11), 1817–1825.

Ben-Yosef, M., Pasternak, Z., Jurkevitch, E., & Yuval, B. (2014). Symbiotic bacteria enable olive flies (*Bactrocera oleae*) to exploit intractable sources of nitrogen. *Journal of Evolutionary Biology* *27*(12), 2695–2705.

Bignell, D. E. (1977). An experimental study of cellulose and hemicellulose degradation in the alimentary canal of the American cockroach. *Canadian Journal of Zoology*, *55*(3), 579–589.

Billen, J., & Buschinger, A. (2000). Morphology and ultrastructure of a specialized bacterial pouch in the digestive tract of *Tetraponera* ants (Formicidae, Pseudomyrmecinae). *Arthropod Structure & Development*, *29*(3), 259–266.

Breznak, J. A. (2000). Ecology of prokaryotic microbes in the guts of wood- and litter-feeding termites. In: Abe, T., Bignell, D.E., Higashi, M. (Eds.), *Termites: Evolution, Sociality, Symbioses, Ecology* (pp. 209–231). Springer Netherlands.

Breznak, J. A., & Switzer, J. M. (1986). Acetate synthesis from H_2_ plus CO_2_ by termite gut microbes. *Applied and Environmental Microbiology*, *52*(4), 623–630.

Brodbeck, B. V., Mizell, R. F., III, & Andersen, P. C. (1993). Physiological and behavioral adaptations of three species of leafhoppers in response to the dilute nutrient content of xylem fluid. *Journal of Insect Physiology*, *39*(1), 73–81.

Brunetti, M., Magoga, G., Gionechetti, F., De Biase, A., & Montagna, M. (2021). Does diet breadth affect the complexity of the phytophagous insect microbiota? The case study of Chrysomelidae. *Environmental Microbiology* *24*(8):3565-3579.

Bustamante-Brito, R., Vera-Ponce de León, A., Rosenblueth, M., Martínez-Romero, J. C., & Martínez-Romero, E. (2019). Metatranscriptomic analysis of the bacterial symbiont *Dactylopiibacterium carminicum* from the Carmine Cochineal *Dactylopius coccus* (Hemiptera: Coccoidea: Dactylopiidae). *Life*, *9*(1), 4.

Calderon, O., & Berkov, A. (2012). Midgut and fat body bacteriocytes in neotropical cerambycid beetles (Coleoptera: Cerambycidae). *Environmental Entomology*, *41*(1), 108–117.

Calderón-Cortés, N., Quesada, M., Watanabe, H., Cano-Camacho, H., & Oyama, K. (2012). Endogenous plant cell wall digestion: a key mechanism in insect evolution. *Annual Review of Ecology, Evolution, and Systematics*, *43*(1), 45–71.

Cameron, S. A., Hines, H. M., & Williams, P. H. (2007). A comprehensive phylogeny of the bumble bees (Bombus). *Biological Journal of the Linnean Society*, *91*(1), 161–188.

Cazemier, A. E., den Camp, H. J. M. O., Hackstein, J. H. P., & Vogels, G. D. (1997). Fibre digestion in arthropods. *Comparative Biochemistry and Physiology. A, Comparative Physiology*, *118*(1), 101–109.

Ceja-Navarro, J. A., Karaoz, U., Bill, M., Hao, Z., White, R. A., 3rd, Arellano, A., Ramanculova, L., Filley, T. R., Berry, T. D., Conrad, M. E., Blackwell, M., Nicora, C. D., Kim, Y.-M., Reardon, P. N., Lipton, M. S., Adkins, J. N., Pett-Ridge, J., & Brodie, E. L. (2019). Gut anatomical properties and microbial functional assembly promote lignocellulose deconstruction and colony subsistence of a wood-feeding beetle. *Nature Microbiology*, *4*(5), 864–875.

Ceja-Navarro, J. A., Nguyen, N. H., Karaoz, U., Gross, S. R., Herman, D. J., Andersen, G. L., Bruns, T. D., Pett-Ridge, J., Blackwell, M., & Brodie, E. L. (2014). Compartmentalized microbial composition, oxygen gradients and nitrogen fixation in the gut *of Odontotaenius disjunctus*. *The ISME Journal*, *8*(1), 6–18.

Cembrowski, A. R., Reurink, G., Arcila Hernandez, L. M., Sanders, J. G., Youngerman, E., & Frederickson, M. E. (2015). Sporadic pollen consumption among tropical ants. *Insectes Sociaux*, *62*(3), 379–382.

Chalcoff, V. R., Aizen, M. A., & Galetto, L. (2006). Nectar concentration and composition of 26 species from the temperate forest of South America. *Annals of Botany*, *97*(3), 413–421.

Collins, N. M. (1983). The utilization of nitrogen resources by termites (Isoptera). In J. A. Lee, S. McNeill, & I. H. Rorison (Eds.), *Nitrogen as an Ecological Factor* (pp. 381–412). Blackwell Scientific Publications.

Conord, C., Despres, L., Vallier, A., Balmand, S., Miquel, C., Zundel, S., Lemperiere, G., & Heddi, A. (2008). Long-term evolutionary stability of bacterial endosymbiosis in Curculionoidea: additional evidence of symbiont replacement in the Dryophthoridae family. *Molecular Biology and Evolution*, *25*(5), 859–868.

Consuegra, J., Grenier, T., Akherraz, H., Rahioui, I., Gervais, H., da Silva, P., & Leulier, F. (2020a). Metabolic cooperation among commensal bacteria supports Drosophila juvenile growth under nutritional stress. *iScience*, *23*(6), 101232.

Consuegra, J., Grenier, T., Baa-Puyoulet, P., Rahioui, I., Akherraz, H., Gervais, H., Parisot, N., da Silva, P., Charles, H., Calevro, F., & Leulier, F. (2020b). *Drosophila*-associated bacteria differentially shape the nutritional requirements of their host during juvenile growth. *PLoS Biology*, *18*(3), e3000681.

Cook, S. C., & Davidson, D. W. (2006). Nutritional and functional biology of exudate‐feeding ants. *Entomologia Experimentalis et Applicata*, *118*(1), 1–10.

Cornwallis, C. K., van ’t Padje, A., Ellers, J., Klein, M., Jackson, R., Kiers, E. T., West, S. A., & Henry, L. M. (2023). Symbioses shape feeding niches and diversification across insects. *Nature Ecology & Evolution 7*, 1022–1044.

Crafts-Brandner, S. J. (2002). Plant nitrogen status rapidly alters amino acid metabolism and excretion in *Bemisia tabaci*. *Journal of Insect Physiology*, *48*(1), 33–41.

Cragg, S. M., Beckham, G. T., Bruce, N. C., Bugg, T. D. H., Distel, D. L., Dupree, P., Etxabe, A. G., Goodell, B. S., Jellison, J., McGeehan, J. E., McQueen-Mason, S. J., Schnorr, K., Walton, P. H., Watts, J. E. M., & Zimmer, M. (2015). Lignocellulose degradation mechanisms across the tree of life. *Current Opinion in Chemical Biology*, *29*, 108–119.

Cummins, K. W. (1974). Structure and function of stream ecosystems. *Bioscience*, *24*(11), 631–641.

Dadd, R. H. (1985). Chapter 8: Nutrition: Organisms. In G. A. Kerkut & L. I. Gilbert (Eds.), *Comprehensive insect physiology, biochemistry, and pharmacology, vol. 4* (pp. 313-390). Pergamon Press Inc, New York.

Davidson, D. W., Cook, S. C., Snelling, R. R., & Chua, T. H. (2003). Explaining the abundance of ants in lowland tropical rainforest canopies. *Science*, *300*(5621), 969–972.

De Cock, M., Virgilio, M., Vandamme, P., Bourtzis, K., De Meyer, M., & Willems, A. (2020). Comparative microbiomics of tephritid frugivorous pests (Diptera: Tephritidae) from the field: a tale of high variability across and within species. *Frontiers in Microbiology*, *11*, 1890.

De Fine Licht, H. H., & Biedermann, P. H. W. (2012). Patterns of functional enzyme activity in fungus farming ambrosia beetles. *Frontiers in Zoology*, *9*(1), 13.

De Fine Licht, H. H., Schiøtt, M., Mueller, U. G., & Boomsma, J. J. (2010). Evolutionary transitions in enzyme activity of ant fungus gardens. *Evolution* *64*(7), 2055–2069.

Dietrich, C., Köhler, T., & Brune, A. (2014). The cockroach origin of the termite gut microbiota: patterns in bacterial community structure reflect major evolutionary events. *Applied and Environmental Microbiology*, *80*(7), 2261–2269.

Dietz, L., Seidel, M., Eberle, J., Misof, B., Pacheco, T. L., Podsiadlowski, L., Ranasinghe, S., Gunter, N. L., Niehuis, O., Mayer, C., & Ahrens, D. (2023). A transcriptome‐based phylogeny of Scarabaeoidea confirms the sister group relationship of dung beetles and phytophagous pleurostict scarabs (Coleoptera). *Systematic Entomology* *48*(4), 672-686.

Douglas, A. E. (2021). *The Symbiotic Habit*. Princeton University Press.

Duplais, C., Sarou-Kanian, V., Massiot, D., Hassan, A., Perrone, B., Estevez, Y., Wertz, J. T., Martineau, E., Farjon, J., Giraudeau, P., & Moreau, C. S. (2021). Gut bacteria are essential for normal cuticle development in herbivorous turtle ants. *Nature Communications*, *12*(1), 676.

Ebert, K. M., Arnold, W. G., Ebert, P. R., & Merritt, D. J. (2021). Hindgut microbiota reflects different digestive strategies in dung beetles (Coleoptera: Scarabaeidae: Scarabaeinae). *Applied and Environmental Microbiology*, *87*(5), e02100-20.

Engl, T., Eberl, N., Gorse, C., Krüger, T., Schmidt, T. H. P., Plarre, R., Adler, C., & Kaltenpoth, M. (2018). Ancient symbiosis confers desiccation resistance to stored grain pest beetles. *Molecular Ecology*, *27*(8), 2095–2108.

Estes, A. M., Hearn, D. J., Snell-Rood, E. C., Feindler, M., Feeser, K., Abebe, T., Dunning Hotopp, J. C., & Moczek, A. P. (2013). Brood ball-mediated transmission of microbiome members in the dung beetle, *Onthophagus taurus* (Coleoptera: Scarabaeidae). *PLoS One*, *8*(11), e79061.

Feldhaar, H., Straka, J., Krischke, M., Berthold, K., Stoll, S., Mueller, M. J., & Gross, R. (2007). Nutritional upgrading for omnivorous carpenter ants by the endosymbiont *Blochmannia*. *BMC Biology*, *5*, 48.

Fisher, R. M., Henry, L. M., Cornwallis, C. K., Kiers, E. T., & West, S. A. (2017). The evolution of host-symbiont dependence. *Nature Communications*, *8*, 15973.

Foster, K. R., & Wenseleers, T. (2006). A general model for the evolution of mutualisms. *Journal of Evolutionary Biology*, *19*(4), 1283–1293.

France, R. (2011). Leaves as “crackers”, biofilm as “peanut butter”: exploratory use of stable isotopes as evidence for microbial pathways in detrital food webs. *Oceanological and Hydrobiological Studies*, *40*(4), 110–115.

Fukumori, K., Oguchi, K., Ikeda, H., Shinohara, T., Tanahashi, M., Moriyama, M., Koga, R., & Fukatsu, T. (2022). Evolutionary dynamics of host organs for microbial symbiosis in tortoise leaf beetles (Coleoptera: Chrysomelidae: Cassidinae). *mBio*, *13*(1), e0369121.

Gall, R., Landolt, W., Schleppi, P., Michellod, V., & Bucher, J. B. (2002). Water content and bark thickness of Norway spruce (*Picea abies*) stems: phloem water capacitance and xylem sap flow. *Tree Physiology*, *22*(9), 613–623.

García-Lozano, M., Henzler, C., Porras, M. Á. G., Pons, I., Berasategui, A., Lanz, C., Budde, H., Oguchi, K., Matsuura, Y., Pauchet, Y., Goffredi, S., Fukatsu, T., Windsor, D., & Salem, H. (2024). Paleocene origin of a streamlined digestive symbiosis in leaf beetles. *Current Biology*, *34*(8), 1621–1634.

GBIF Secretariat: GBIF Backbone Taxonomy. https://doi.org/10.15468/39omei Accessed August 1, 2023

Geib, S. M., Filley, T. R., Hatcher, P. G., Hoover, K., Carlson, J. E., Jimenez-Gasco, M. del M., Nakagawa-Izumi, A., Sleighter, R. L., & Tien, M. (2008). Lignin degradation in wood-feeding insects. *Proceedings of the National Academy of Sciences of the United States of America*, *105*(35), 12932–12937.

Genissel, A., Aupinel, P., Bressac, C., Tasei, J.-N., & Chevrier, C. (2002). Influence of pollen origin on performance of *Bombus terrestris* micro-colonies. *Entomologia Experimentalis et Applicata*, *104*(2-3), 329–336.

Gollan, T., Schurr, U., & Schulze, E.-D. (1992). Stomatal response to drying soil in relation to changes in the xylem sap composition of *Helianthus annuus*. I. The concentration of cations, anions, amino acids in, and pH of, the xylem sap. *Plant, Cell & Environment*, *15*(5), 551–559.

González, E., Bianchi, F. J. J. A., Wizorek, S., Schumacher, M., & Entling, M. H. (2022). Variation between plant species in pollen digestion rates in the green lacewing *Chrysoperla carnea*. *Entomologia Experimentalis et Applicata*, *170*(12), 1049–1054.

Gordon, E. R. L., McFrederick, Q., & Weirauch, C. (2016). Phylogenetic Evidence for Ancient and Persistent Environmental Symbiont Reacquisition in Largidae (Hemiptera: Heteroptera). *Applied and Environmental Microbiology*, *82*(24), 7123–7133.

Grünwald, S., Pilhofer, M., & Höll, W. (2010). Microbial associations in gut systems of wood- and bark-inhabiting longhorned beetles [Coleoptera: Cerambycidae]. *Systematic and Applied Microbiology*, *33*(1), 25–34.

Hammer, T. J. (2024). Why do hosts malfunction without microbes? Missing benefits versus evolutionary addiction. *Trends in Microbiology*, *32*(2), 132–141.

Hansen, A. K., Pers, D., & Russell, J. A. (2020). Chapter Five - Symbiotic solutions to nitrogen limitation and amino acid imbalance in insect diets. In K. M. Oliver & J. A. Russell (Eds.), *Advances in Insect Physiology* 58: *Mechanisms underlying microbial symbiosis* (pp. 161-205). Academic Press.

Harrop-Archibald, H., Didham, R. K., Standish, R. J., Tibbett, M., & Hobbs, R. J. (2016). Mechanisms linking fungal conditioning of leaf litter to detritivore feeding activity. *Soil Biology & Biochemistry*, *93*, 119–130.

Hosokawa, T., & Fukatsu, T. (2010). *Nardonella* endosymbiont in the West Indian sweet potato weevil *Euscepes postfasciatus* (Coleoptera: Curculionidae). *Applied Entomology and Zoology*, *45*(1), 115–120.

Huang, S.-W., Zhang, H.-Y., Marshall, S., & Jackson, T. A. (2010). The scarab gut: a potential bioreactor for bio‐fuel production. *Insect Science*, *17*(3), 175–183.

Hu, Y., Sanders, J. G., Łukasik, P., D’Amelio, C. L., Millar, J. S., Vann, D. R., Lan, Y., Newton, J. A., Schotanus, M., Kronauer, D. J. C., Pierce, N. E., Moreau, C. S., Wertz, J. T., Engel, P., & Russell, J. A. (2018). Herbivorous turtle ants obtain essential nutrients from a conserved nitrogen-recycling gut microbiome. *Nature Communications, 9*, 964.

Huang, X., Huang, Y., Zhang, J., Lu, F., Wei, J., & Jiang, M. (2016). The symbiotic bacteria *Nardonella* in rice water weevil (Coleoptera: Curculionidae): diversity, density, and associations with host reproduction. *Annals of the Entomological Society of America*, *109*(3), 415–423.

Huang, Y.-T., Skelton, J., & Hulcr, J. (2019). Multiple evolutionary origins lead to diversity in the metabolic profiles of ambrosia fungi. *Fungal Ecology*, *38*, 80–88.

Hulcr, J., Barnes, I., De Beer, Z. W., Duong, T. A., Gazis, R., Johnson, A. J., Jusino, M. A., Kasson, M. T., Li, Y., Lynch, S., Mayers, C., Musvuugwa, T., Roets, F., Seltmann, K. C., Six, D., Vanderpool, D., & Villari, C. (2020). Bark beetle mycobiome: collaboratively defined research priorities on a widespread insect-fungus symbiosis. *Symbiosis 81*(2), 101–113.

Hulcr, J., & Stelinski, L. L. (2017). The ambrosia symbiosis: from evolutionary ecology to practical management. *Annual Review of Entomology*, *62*(1), 285–303.

Human, H., H Human, & Nicolson, S. W. (2003). Digestion of maize and sunflower pollen by the spotted maize beetle *Astylus atromaculatus* (Melyridae): is there a role for osmotic shock? *Journal of Insect Physiology* *49*(7), 633–643.

Husnik, F., & McCutcheon, J. P. (2016). Repeated replacement of an intrabacterial symbiont in the tripartite nested mealybug symbiosis. *Proceedings of the National Academy of Sciences of the United States of America*, *113*(37), E5416–E5424.

Jensen, K. H., Savage, J. A., & Holbrook, N. M. (2013). Optimal concentration for sugar transport in plants. *Journal of the Royal Society, Interface*, *10*(83), 20130055.

Jing, T.-Z., Qi, F.-H., & Wang, Z.-Y. (2020). Most dominant roles of insect gut bacteria: digestion, detoxification, or essential nutrient provision? *Microbiome*, *8*(1), 38.

Ji, R., & Brune, A. (2005). Digestion of peptidic residues in humic substances by an alkali-stable and humic-acid-tolerant proteolytic activity in the gut of soil-feeding termites. *Soil Biology & Biochemistry*, *37*(9), 1648–1655.

Jurzitza, G. (1970). Über isolierung, kultur und taxonomie einiger anobiidensymbionten (Insecta, Coleoptera). *Archives of Microbiology*, *72*(3), 203–222.

Jurzitza, G. (1972). Rasterelektronenmikroskopische untersuchungen über die strukturen der oberfläche von anobiideneiern (Coleoptera) und über die verteilung der endosymbionten auf den eischalen. *Forma et Functio*, *5*, 75–88.

Kanyile, S. N., Engl, T., & Kaltenpoth, M. (2022). Nutritional symbionts enhance structural defence against predation and fungal infection in a grain pest beetle. *The Journal of Experimental Biology*, *225*(1), jeb243593.

Kaufman M. G., & Klug, M. J. (1990). Microbial community metabolism in the digestive tract of crickets: (Orthoptera:Gryllidae): Implications for omnivorous insects. In R. Lésel (Ed.), *Microbiology in Poecilotherms* (pp. 69–72). Elsevier.

Kešnerová, L., Mars, R. A. T., Ellegaard, K. M., Troilo, M., Sauer, U., & Engel, P. (2017). Disentangling metabolic functions of bacteria in the honey bee gut. *PLoS Biology*, *15*(12), e2003467.

Kinjo, Y., Bourguignon, T., Tong, K. J., Kuwahara, H., Lim, S. J., Yoon, K. B., Shigenobu, S., Park, Y. C., Nalepa, C. A., Hongoh, Y., Ohkuma, M., Lo, N., & Tokuda, G. (2018). Parallel and gradual genome erosion in the *Blattabacterium* endosymbionts of *Mastotermes darwiniensis* and *Cryptocercus* wood roaches. *Genome Biology and Evolution*, *10*(6), 1622–1630.

Kirsch, R., Gramzow, L., Theißen, G., Siegfried, B. D., Ffrench-Constant, R. H., Heckel, D. G., & Pauchet, Y. (2014). Horizontal gene transfer and functional diversification of plant cell wall degrading polygalacturonases: key events in the evolution of herbivory in beetles. *Insect Biochemistry and Molecular Biology*, *52*, 33–50.

Kirsch, R., Okamura, Y., García-Lozano, M., Weiss, B., Keller, J., Vogel, H., Fukumori, K., Fukatsu, T., Konstantinov, A. S., Montagna, M., Moseyko, A. G., Riley, E. G., Slipinski, A., Vencl, F. V., Windsor, D. M., Salem, H., Kaltenpoth, M., & Pauchet, Y. (2025). Symbiosis and horizontal gene transfer promote herbivory in the megadiverse leaf beetles. *Current Biology*, *35*(3), 640–654.

Kishigami, M., Matsuoka, F., Maeno, A., Yamagishi, S., Abe, H., & Toki, W. (2023). Yeast associated with flower longicorn beetle *Leptura ochraceofasciata* (Cerambycidae: Lepturinae), with implication for its function in symbiosis. *PLoS One*, *18*(3), 1–17.

Kleinschmidt, B., & Kölsch, G. (2011). Adopting bacteria in order to adapt to water-how Reed beetles colonized the wetlands (Coleoptera, Chrysomelidae, Donaciinae). *Insects*, *2*(4), 540–554.

Knicker, H., & Lüdemann, H.-D. (1995). N-15 and C-13 CPMAS and solution NMR studies of N-15 enriched plant material during 600 days of microbial degradation. *Organic Geochemistry*, *23*(4), 329–341.

Kostina, N. V., Chernysheva, A. N., Vecherskii, M. V., & Kuznetsova, T. A. (2020). Microbial nitrogen fixation in the intestine of Tipulidae *Tipula maxima* larvae. *Biology Bulletin of the Russian Academy of Sciences*, *47*(1), 35–39.

Kostovcik, M., Bateman, C. C., Kolarik, M., Stelinski, L. L., Jordal, B. H., & Hulcr, J. (2015). The ambrosia symbiosis is specific in some species and promiscuous in others: evidence from community pyrosequencing. *The ISME Journal*, *9*(1), 126–138.

Kwong, W. K., Medina, L. A., Koch, H., Sing, K.-W., Soh, E. J. Y., Ascher, J. S., Jaffé, R., & Moran, N. A. (2017). Dynamic microbiome evolution in social bees. *Science Advances*, *3*(3), e1600513.

Labandeira, C. (2014). Why did terrestrial insect diversity not increase during the angiosperm radiation? Mid-Mesozoic, plant-associated insect lineages harbor clues. In P. Pontarotti (Ed.), *Evolutionary Biology: Genome evolution, speciation, coevolution and origin of life* (pp. 261–299). Springer International Publishing.

Lee, F. J., Miller, K. I., McKinlay, J. B., & Newton, I. L. G. (2018). Differential carbohydrate utilization and organic acid production by honey bee symbionts. *FEMS Microbiology Ecology*, *94*(8), fiy113.

Lefèvre, C., Charles, H., Vallier, A., Delobel, B., Farrell, B., & Heddi, A. (2004). Endosymbiont phylogenesis in the Dryophthoridae weevils: evidence for bacterial replacement. *Molecular Biology and Evolution*, *21*(6), 965–973.

Legendre, F., Nel, A., Svenson, G. J., Robillard, T., Pellens, R., & Grandcolas, P. (2015). Phylogeny of Dictyoptera: dating the origin of cockroaches, praying mantises and termites with molecular data and controlled fossil evidence. *PLoS One*, *10*(7), e0130127.

Li, H., Leavengood, J. M., Jr, Chapman, E. G., Burkhardt, D., Song, F., Jiang, P., Liu, J., Zhou, X., & Cai, W. (2017a). Mitochondrial phylogenomics of Hemiptera reveals adaptive innovations driving the diversification of true bugs. [*Proceedings of the Royal Society of London, Series B, Biological Sciences*,](http://paperpile.com/b/C5m4ez/MNxJ) *284*(1862), 20171223.

Li, H., Yelle, D. J., Li, C., Yang, M., Ke, J., Zhang, R., Liu, Y., Zhu, N., Liang, S., Mo, X., Ralph, J., Currie, C. R., & Mo, J. (2017b). Lignocellulose pretreatment in a fungus-cultivating termite. *Proceedings of the National Academy of Sciences of the United States of America, 114*(18), 4709–4714.

Li, H., Young, S. E., Poulsen, M., & Currie, C. R. (2021). Symbiont-mediated digestion of plant biomass in fungus-farming insects. *Annual Review of Entomology*, *66*(1), 297–316.

Linsley, E. G. (1959). Ecology of Cerambycidae. *Annual Review of Entomology*, *4*(1), 99–138.

Lintunen, A., Paljakka, T., Jyske, T., Peltoniemi, M., Sterck, F., von Arx, G., Cochard, H., Copini, P., Caldeira, M. C., Delzon, S., Gebauer, R., Grönlund, L., Kiorapostolou, N., Lechthaler, S., Lobo-do-Vale, R., Peters, R. L., Petit, G., Prendin, A. L., Salmon, Y., … Hölttä, T. (2016). Osmolality and non-structural carbohydrate composition in the secondary phloem of trees across a latitudinal gradient in Europe. *Frontiers in Plant Science*, *7*, 726.

Ma, M., Tu, C., Luo, J., Lu, M., Zhang, S., & Xu, L. (2021). Metabolic and immunological effects of gut microbiota in leaf beetles at the local and systemic levels. *Integrative Zoology*, *16*(3), 313–323.

Mann, J. S., & Crowson, R. A. (1983). On the occurrence of mid-gut caeca, and organs of symbiont transmission, in leaf-beetles (Coleoptera: Chrysomelidae). *The Coleopterists’ Bulletin*, *37*(1), 1–15.

Martoja, R. (1962). Assimilation of cellulose and intestinal microorganisms in *Gryllus bimaculatus* De Geer (Insecta, Orthoptera, Grylloidea). *Comptes Rendus Hebdomadaires des Seances de l’Academie des Sciences*, *254*, 3040–3042.

Mason, C. J., Auth, J., & Geib, S. M. (2023). Gut bacterial population and community dynamics following adult emergence in pest tephritid fruit flies. *Scientific Reports*, *13*(1), 13723.

Mauldin, J. K., Smythe, R. V., & Baxter, C. C. (1972). Cellulose catabolism and lipid synthesis by the subterranean termite, *Coptotermes formosanus*. *Insect Biochemistry*, *2*(6), 209–217.

Mazzon, L., Piscedda, A., Simonato, M., Martinez-Sañudo, I., Squartini, A., & Girolami, V. (2008). Presence of specific symbiotic bacteria in flies of the subfamily Tephritinae (Diptera Tephritidae) and their phylogenetic relationships: proposal of “*Candidatus* Stammerula tephritidis.” *International Journal of Systematic and Evolutionary Microbiology*, *58*(Pt 6), 1277–1287.

McKenna, D. D., Shin, S., Ahrens, D., Balke, M., Beza-Beza, C., Clarke, D. J., Donath, A., Escalona, H. E., Friedrich, F., Letsch, H., Liu, S., Maddison, D., Mayer, C., Misof, B., Murin, P. J., Niehuis, O., Peters, R. S., Podsiadlowski, L., Pohl, H., … Beutel, R. G. (2019). The evolution and genomic basis of beetle diversity. *Proceedings of the National Academy of Sciences of the United States of America*, *116*(49), 24729–24737.

Mee, L., & Barribeau, S. M. (2023). Influence of social lifestyles on host-microbe symbioses in the bees. *Ecology and Evolution*, *13*(11), e10679.

Mifsud, I. E. J., Akana, P. R., Bytnerowicz, T. A., Davis, S. R., & Menge, D. N. L. (2023). Nitrogen fixation in the stag beetle, *Ceruchus piceus* (Coleoptera: Lucanidae): could insects contribute more to ecosystem nitrogen budgets than previously thought? *Environmental Entomology*, *52*(4), 618–626.

Mikaelyan, A., Dietrich, C., Köhler, T., Poulsen, M., Sillam-Dussès, D., & Brune, A. (2015). Diet is the primary determinant of bacterial community structure in the guts of higher termites. *Molecular Ecology*, *24*(20), 5284–5295.

Morera-Margarit, P., Bulgarelli, D., Pope, T. W., Graham, R. I., Mitchell, C., & Karley, A. J. (2019). The bacterial community associated with adult vine weevil (*Otiorhynchus sulcatus*) in UK populations growing on strawberry is dominated by *Candidatus* Nardonella. *Entomologia Experimentalis et Applicata*, *167*(3), 186–196.

Nalepa, C. A. (2020). Origin of mutualism between termites and flagellated gut protists: transition from horizontal to vertical transmission. *Frontiers in Ecology and Evolution*, *8*, 14.

Nalepa, C. A., Bignell, D. E., & Bandi, C. (2001). Detritivory, coprophagy, and the evolution of digestive mutualisms in Dictyoptera. *Insectes Sociaux*, *48*(3), 194–201.

[Nikoh, N., Hosokawa, T., & Moriyama, M. (2014). Evolutionary origin of insect–*Wolbachia* nutritional mutualism. *Proceedings of the*](http://paperpile.com/b/C5m4ez/JcPN) *National Academy of Sciences of the United States of America 111*(28), 10257-10262.

Onchuru, T. O., Javier Martinez, A., Ingham, C. S., & Kaltenpoth, M. (2018). Transmission of mutualistic bacteria in social and gregarious insects. *Current Opinion in Insect Science*, *28*, 50–58.

Nogueira, D. S. (2023). Overview of stingless bees in Brazil (Hymenoptera: Apidae: Meliponini). *EntomoBrasilis*, *16: e1041*.

Odelson, D. A., & Breznak, J. A. (1983). Volatile fatty acid production by the hindgut microbiota of xylophagous termites. *Applied and Environmental Microbiology*, *45*(5), 1602–1613.

Ohkuma, M., Noda, S., Hongoh, Y., Nalepa, C. A., & Inoue, T. (2009). Inheritance and diversification of symbiotic trichonymphid flagellates from a common ancestor of termites and the cockroach *Cryptocercus*. *Proceedings. Biological Sciences*, *276*(1655), 239–245.

Paljakka, T., Rissanen, K., Vanhatalo, A., Salmon, Y., Jyske, T., Prisle, N. L., Linnakoski, R., Lin, J. J., Laakso, T., Kasanen, R., Bäck, J., & Hölttä, T. (2020). Is decreased xylem sap surface tension associated with embolism and loss of xylem hydraulic conductivity in pathogen-infected Norway spruce saplings? *Frontiers in Plant Science*, *11*, 1090.

Parker, E. S., Moczek, A. P., & Macagno, A. L. M. (2021). Reciprocal microbiome transplants differentially rescue fitness in two syntopic dung beetle sister species (Scarabaeidae: *Onthophagus*). *Ecological Entomology*, *46*(4), 946–954.

Pauchet, Y., & Heckel, D. G. (2013). The genome of the mustard leaf beetle encodes two active xylanases originally acquired from bacteria through horizontal gene transfer. *Proceedings. Biological Sciences*, *280*(1763), 20131021.

Pinto-Tomás, A. A., Anderson, M. A., Suen, G., Stevenson, D. M., Chu, F. S. T., Cleland, W. W., Weimer, P. J., & Currie, C. R. (2009). Symbiotic nitrogen fixation in the fungus gardens of leaf-cutter ants. *Science*, *326*(5956), 1120–1123.

Rainford, J. L., Hofreiter, M., Nicholson, D. B., & Mayhew, P. J. (2014). Phylogenetic distribution of extant richness suggests metamorphosis is a key innovation driving diversification in insects. *PLoS One*, *9*(9), e109085.

R Core Team. (2020). R: A language and environment for statistical computing. R Foundation for Statistical Computing.

Rainford, J. L., & Mayhew, P. J. (2015). Diet evolution and clade richness in Hexapoda: a phylogenetic study of higher taxa. *The American Naturalist*, *186*(6), 777–791.

Rao, P. G., & Mallikarjuna, K. (1979). Physiological changes associated with gall formation in *Pongamia pinnata*, *Diospyros melanoxylon* *and Argeria chosiana*. *Proceedings of the Indian Academy of Sciences. Section B, Biological Sciences*, *88*(5), 397–400.

Reis, F., Kirsch, R., Pauchet, Y., Bauer, E., Bilz, L. C., Fukumori, K., Fukatsu, T., Kölsch, G., & Kaltenpoth, M. (2020). Bacterial symbionts support larval sap feeding and adult folivory in (semi-)aquatic reed beetles. *Nature Communications*, *11*(1), 2964.

Ren, F.-R., Sun, X., Wang, T.-Y., Yao, Y.-L., Huang, Y.-Z., Zhang, X., & Luan, J.-B. (2020). Biotin provisioning by horizontally transferred genes from bacteria confers animal fitness benefits. *The ISME Journal*, *14*(10), 2542–2553.

Ren, X., Guo, R., Akami, M., & Niu, C. (2022). Nitrogen acquisition strategies mediated by insect symbionts: a review of their mechanisms, methodologies, and case studies. *Insects*, *13*(1), 84.

Rice, D. L. (1982). The detritus nitrogen problem: new observations and perspectives from organic geochemistry. *Marine Ecology Progress Series*, *9*(2), 153–162.

Rose, C., Lund, M. B., Søgård, A. M., Busck, M. M., Bechsgaard, J. S., Schramm, A., & Bilde, T. (2023). Social transmission of bacterial symbionts homogenizes the microbiome within and across generations of group-living spiders. *ISME Communications*, *3*(1), 60.

Russell, J. A., Oliver, K. M., & Hansen, A. K. (2017). Band-aids for *Buchnera* and B vitamins for all. *Molecular Ecology*, *26*(8), 2199–2203.

Salem, H., Bauer, E., Kirsch, R., Berasategui, A., Cripps, M., Weiss, B., Koga, R., Fukumori, K., Vogel, H., Fukatsu, T., & Kaltenpoth, M. (2017). Drastic genome reduction in an herbivore’s pectinolytic symbiont. *Cell* *171*(7), 1520–1531.

Salem, H., Florez, L., Gerardo, N., & Kaltenpoth, M. (2015). An out-of-body experience: the extracellular dimension for the transmission of mutualistic bacteria in insects. [*Proceedings of the Royal Society of London, Series B, Biological Sciences*,](http://paperpile.com/b/C5m4ez/MNxJ) *282*(1804), 20142957.

Salem, H., & Kaltenpoth, M. (2022). Beetle-bacterial symbioses: endless forms most functional. *Annual Review of Entomology*, *67*(1), 201–219.

Sasaki, T., Aoki, T., Hayashi, H., & Ishikawa, H. (1990). Amino acid composition of the honeydew of symbiotic and aposymbiotic pea aphids *Acyrthosiphon pisum*. *Journal of Insect Physiology*, *36*(1), 35–40.

Sasakura, Y., Yuzawa, N., Yamasako, J., Mori, K., Horie, T., & Nonaka, M. (2024). Environment-mediated vertical transmission fostered uncoupled phylogenetic relationships between longicorn beetles and their symbionts. *Zoological Science*, *41*(4), 363–376.

Scharf, M. E., & Peterson, B. F. (2021). A century of synergy in termite symbiosis research: linking the past with new genomic insights. *Annual Review of Entomology*, *66*(1), 23–43.

Schwab, D. B., Riggs, H. E., Newton, I. L. G., & Moczek, A. P. (2016). Developmental and ecological benefits of the maternally transmitted mcrobiota in a dung beetle. *The American Naturalist*, *188*(6), 679–692.

Scully, E. D., Geib, S. M., Carlson, J. E., Tien, M., McKenna, D., & Hoover, K. (2014). Functional genomics and microbiome profiling of the Asian longhorned beetle (*Anoplophora glabripennis*) reveal insights into the digestive physiology and nutritional ecology of wood feeding beetles. In *BMC Genomics* *15*(1), 1096.

Serbus, L. R., Rodriguez, B. G., Sharmin, Z., Momtaz, A. J. M. Z., & Christensen, S. (2017). Predictive genomic analyses inform the basis for vitamin metabolism and provisioning in bacteria-arthropod endosymbioses. *G3*, *7*(6), 1887–1898.

Shelef, O., Helman, Y., Friedman, A.-L.-L., Behar, A., & Rachmilevitch, S. (2013). Tri-party underground symbiosis between a weevil, bacteria and a desert plant. *PLoS One*, *8*(11), e76588.

Shelomi, M., Danchin, E. G. J., Heckel, D., Wipfler, B., Bradler, S., Zhou, X., & Pauchet, Y. (2016). Horizontal gene transfer of pectinases from bacteria preceded the diversification of stick and leaf insects. *Scientific Reports*, *6*, 26388.

Sinsabaugh, R. L., Linkins, A. E., & Benfield, E. F. (1985). Cellulose digestion and assimilation by three leaf-shredding aquatic insects. *Ecology*, *66*(5), 1464–1471.

Six, D. L. (2012). Ecological and evolutionary determinants of bark beetle -fungus symbioses. *Insects*, *3*(1), 339–366.

Six, D. L., & Klepzig, K. D. (2021). Context dependency in bark beetle-fungus mutualisms revisited: assessing potential shifts in interaction outcomes against varied genetic, ecological, and evolutionary backgrounds. *Frontiers in Microbiology*, *12*, 682187.

Snyder, A. K., & Rio, R. V. M. (2015). ``*Wigglesworthia morsitans*’' folate (vitamin B9) biosynthesis contributes to tsetse host fitness. *Applied and Environmental Microbiology*, *81*(16), 5375–5386.

Song, N., Wang, M., Zhai, Q., & Zhang, H. (2025). Insights into the phylogeny of longhorn beetles from phylogenomic data. *Zoological Journal of the Linnean Society*, *203*(1), zlae174.

Šustr, V., Stingl, U., & Brune, A. (2014). Microprofiles of oxygen, redox potential, and pH, and microbial fermentation products in the highly alkaline gut of the saprophagous larva of *Penthetria holosericea* (Diptera: Bibionidae). *Journal of Insect Physiology*, *67*, 64–69.

Tanahashi, M., Kubota, K., Matsushita, N., & Togashi, K. (2010). Discovery of mycangia and the associated xylose-fermenting yeasts in stag beetles (Coleoptera: Lucanidae). *Sciences & Nature*, *97*(3), 311–317.

Tayasu, I., Abe, T., Eggleton, P., & Bignell, D. E. (1997). Nitrogen and carbon isotope ratios in termites: an indicator of trophic habit along the gradient from wood‐feeding to soil‐feeding. *Ecological Entomology*, *22*(3), 343–351.

Täyasu, I., Sugimoto, A., Wada, E., & Abe, T. (1994). Xylophagous termites depending on atmospheric nitrogen. *Sciences & Nature*, *81*(5), 229–231.

Thompson, B. M. (2013). *Community Ecology and* Sirex noctilio*: Interactions with Microbial Symbionts and Native Insects*. University of Maryland, College Park.

Toju, H., Tanabe, A. S., Notsu, Y., Sota, T., & Fukatsu, T. (2013). Diversification of endosymbiosis: replacements, co-speciation and promiscuity of bacteriocyte symbionts in weevils. *The ISME Journal*, *7*(7), 1378–1390.

Toki, W., & Aoki, D. (2021). Nutritional resources of the yeast symbiont cultivated by the lizard beetle *Doubledaya bucculenta* in bamboos. *Scientific Reports*, *11*(1), 19208.

Vanderpool, D., Bracewell, R. R., & McCutcheon, J. P. (2018). Know your farmer: Ancient origins and multiple independent domestications of ambrosia beetle fungal cultivars. *Molecular Ecology*, *27*(8), 2077–2094.

Vigneron, A., Masson, F., Vallier, A., Balmand, S., Rey, M., Vincent-Monégat, C., Aksoy, E., Aubailly-Giraud, E., Zaidman-Rémy, A., & Heddi, A. (2014). Insects recycle endosymbionts when the benefit is over. *Current Biology*, *24*(19), 2267–2273.

Vitello, D. J., Ripper, R. M., Fettiplace, M. R., Weinberg, G. L., & Vitello, J. M. (2015). Blood density is nearly equal to water density: a validation study of the gravimetric method of measuring intraoperative blood loss. *Journal of Veterinary Medicine*, *2015*, 152730.

Wang, Y., Brożek, J., & Dai, W. (2020). Comparative morphology of the mouthparts in three predatory stink bugs (Heteroptera: Asopinae) reveals feeding specialization of stylets and sensilla*. Insects, 11*(11), 762.

Weiner, C. N., Hilpert, A., Werner, M., Linsenmair, K. E., & Blüthgen, N. (2010). Pollen amino acids and flower specialisation in solitary bees. *Apidologie*, *41*(4), 476–487.

Wertz, J. T., & Béchade, B. (2020). Chapter three - Symbiont-mediated degradation of dietary carbon sources in social herbivorous insects. In K. M. Oliver & J. A. Russell (Eds.), *Advances in Insect Physiology* 58: *Mechanisms underlying microbial symbiosis* (pp. 63-109). Academic Press.

White, J. A., Richards, N. K., Laugraud, A., Saeed, A., Curry, M. M., & McNeill, M. R. (2015). Endosymbiotic candidates for parasitoid defense in exotic and native New Zealand weevils. *Microbial Ecology*, *70*(1), 274–286.

Wu, W., Qiao, J., Xiao, X., Kong, L., Dong, J., & Zhang, H. (2021). *In vitro* and *in vivo* digestion comparison of bee pollen with or without wall-disruption. *Journal of the Science of Food and Agriculture*, *101*(7), 2744–2755.

Wybouw, N., Pauchet, Y., Heckel, D. G., & Van Leeuwen, T. (2016). Horizontal gene transfer contributes to the evolution of arthropod herbivory. *Genome Biology and Evolution*, *8*(6), 1785–1801.

Zhang, G., Browne, P., Zhen, G., Johnston, A., Cadillo-Quiroz, H., & Franz, N. (2017). Endosymbiont diversity and evolution across weevil tree of life. *bioRxiv: The Preprint Server for Biology*.

Zheng, H., Perreau, J., Powell, J. E., Han, B., Zhang, Z., Kwong, W. K., Tringe, S. G., & Moran, N. A. (2019). Division of labor in honey bee gut microbiota for plant polysaccharide digestion. *Proceedings of the National Academy of Sciences of the United States of America*, *116*(51), 25909–25916.

Zheng, H., Powell, J. E., Steele, M. I., Dietrich, C., & Moran, N. A. (2017). Honeybee gut microbiota promotes host weight gain via bacterial metabolism and hormonal signaling. *Proceedings of the National Academy of Sciences of the United States of America*, *114*(18), 4775–4780.
